# Inverting External Asymmetric Induction via Selective Energy Transfer Catalysis: A Strategy to β‐Chiral Phosphonate Antipodes

**DOI:** 10.1002/anie.201911651

**Published:** 2019-11-26

**Authors:** Carina Onneken, Kathrin Bussmann, Ryan Gilmour

**Affiliations:** ^1^ Organisch Chemisches Institut Westfälische Wilhelms-Universität Münster Corrensstraße 40 48149 Münster Germany

**Keywords:** catalysis, energy transfer, hydrogenation, organophotocatalysis, stereodivergence

## Abstract

Enantiodivergent, catalytic reduction of activated alkenes relays stereochemical information encoded in the antipodal chiral catalysts to the pro‐chiral substrate. Although powerful, the strategy remains vulnerable to costs and availability of sourcing both catalyst enantiomers. Herein, a stereodivergent hydrogenation of α,β‐unsaturated phosphonates is disclosed using a single enantiomer of the catalyst. This enables generation of the *R*‐ or *S*‐configured β‐chiral phosphonate with equal and opposite selectivity. Enantiodivergence is regulated at the substrate level through the development of a facile *E* → *Z* isomerisation. This has been enabled for the first time by selective energy transfer catalysis using anthracene as an inexpensive organic photosensitiser. Synthetically valuable in its own right, this process enables subsequent Rh^I^‐mediated stereospecific hydrogenation to generate both enantiomers of the product using only the *S*‐catalyst (up to 99:1 and 3:97 *e.r*.). This strategy out‐competes the selectivities observed with the *E*‐substrate and the *R*‐catalyst.

## Graphical abstract

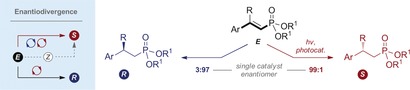

A defining feature of external asymmetric induction is that the degree and direction of stereoselectivity may be regulated at the catalyst/reagent level.^1^ This general premise enables achiral substrates to be processed to one of two chiral antipodes by design under the auspices of catalyst control.^2^ Expansive and transformative, this strategy continues to profit from the breadth and diversity of chiral pool entities available for inclusion in catalyst scaffolds.^3^ Although essential for life, homochirality^4^ intrinsically limits external asymmetric induction due to the deficiency of unnatural biomolecules (e.g. l‐sugars and d‐amino acids). This vulnerability often has a negative impact on enantiodivergent synthesis^5^ due to limited availability/higher costs of one chiral stereodirecting element. It follows that conceptual paradigms in which a second, synergistic external stimulus may be harnessed to enable enantiodivergence would be valuable and thereby mitigate the current reliance on an enantiomeric catalyst pair.^6^ Scenarios including elevated temperature^7^ or mechanical stress^8^ may be considered, but the operational simplicity of light irradiation prompted an investigation of energy transfer‐based stimuli.^9^, ^10^, ^11^ Given the value of alkenes as pro‐chiral substrates in asymmetric catalysis, and the renaissance of positional^12^ and geometric isomerisation^13^ enabled by selective energy transfer,^14^ an isomerisation/stereospecific reduction sequence would provide a useful platform to validate the working hypothesis. To that end, α,β‐unsaturated phosphonates were explored due to their prominence in the pharmaceutical and agrochemical sectors, biochemical significance and venerable history in synthesis (Scheme 1).^15^, ^16^ Herein, the first photocatalytic *E* → *Z* isomerisation of α,β‐unsaturated phosphonates is disclosed using an inexpensive organocatalyst. Merging this with a stereospecific, Rh^I^‐mediated hydrogenation^17^ results in an enantiodivergent paradigm to access the *R*‐ or *S*‐configured β‐chiral phosphonate. Equal and opposite selectivity can be attained using only one catalyst enantiomer.

**Scheme 1:**
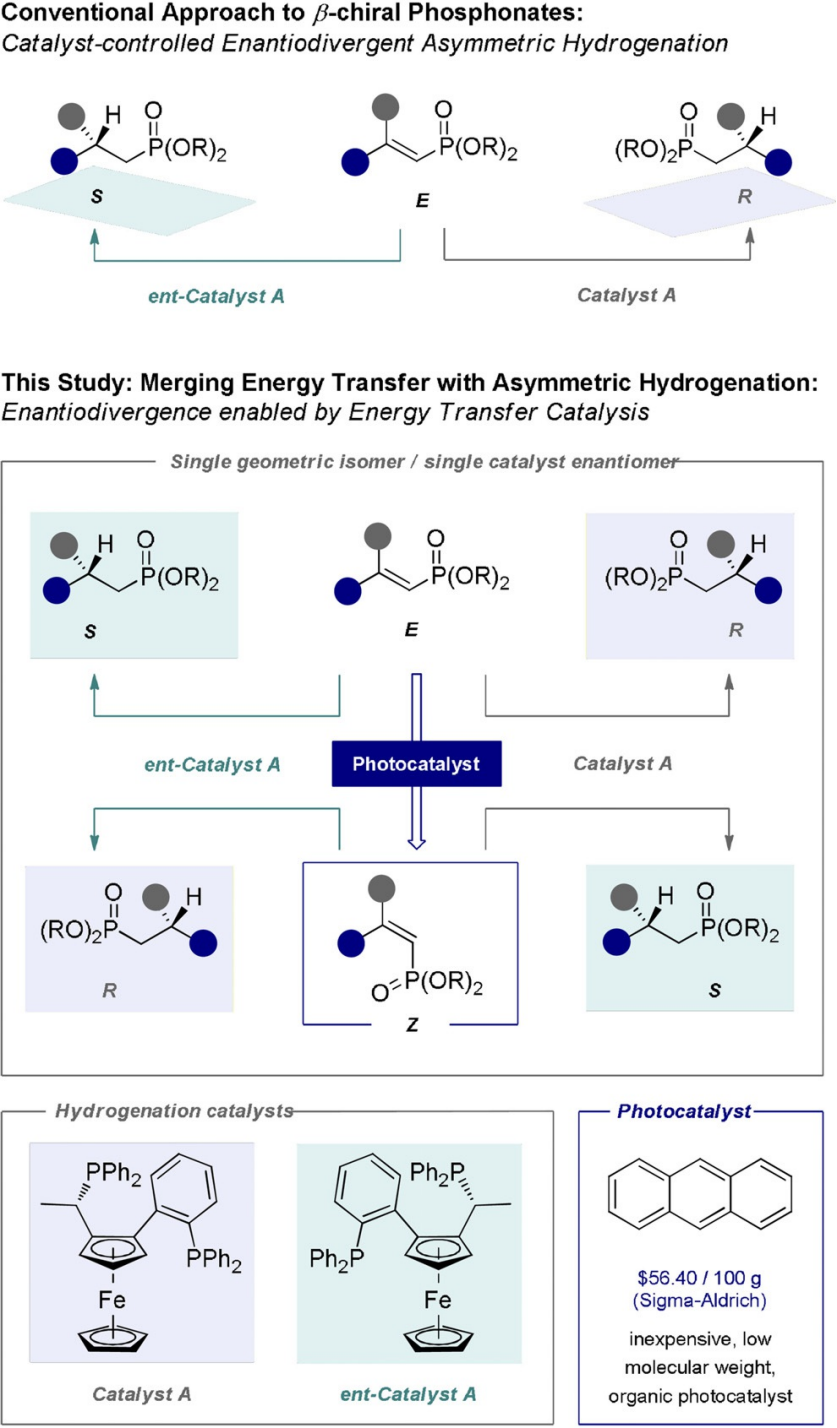
Top: External asymmetric induction approach to β‐chiral phosphonates requiring two enantiomeric catalysts. Bottom: An enantiodivergent platform based on photocatalytic *E*→*Z* isomerisation to generate the *R*‐ or *S*‐configured product using the same catalyst enantiomer.

To establish conditions for the geometrical isomerisation, the conversion of ***E*** **‐1** to ***Z*** **‐1** was investigated as a model reaction, due to the ease with which the substrate can be prepared selectively. It was envisaged that selective energy transfer from a simple organic photosensitiser would facilitate isomerisation of the *E*‐isomer.^18^ In contrast, 1,3‐allylic strain (A^1,3^)^19^ in the product *Z*‐isomer would inhibit re‐excitation thereby endowing the transformation with the desired directionality (Table 1, top). Due to the closely similar absorption spectra of the isomers at ca. 220–270 nm, attention was focused on the more attractive UV‐visible region (ca. 365 nm and above). Excitation in this range is highly compatible with a diverse array of common small molecule photocatalysts (see Supporting Information).^20^ A concise screen conducted in acetonitrile quickly eliminated Ir(ppy)_3_ (450 nm), (−)‐riboflavin, and benzil (both 402 nm) due to poor selectivities (*Z*:*E* 13:87, 66:34 and 25:75, respectively (Table 1, entries 1–3). Only thioxanthone showed a promising 86:14 *Z*:*E* ratio (entry 4), but since this process is part of a stereodivergent sequence, a more efficient catalyst was required. Switching to benzophenone (at 365 nm) preserved the selectivity (entry 5), but the best *Z*:*E* ratio was observed with anthracene (92:08, entry 6).

**Table 1:** Optimisation of the *E* → *Z* isomerisation of vinyl phosphonate ***E*** **‐1**.^[a]^ 
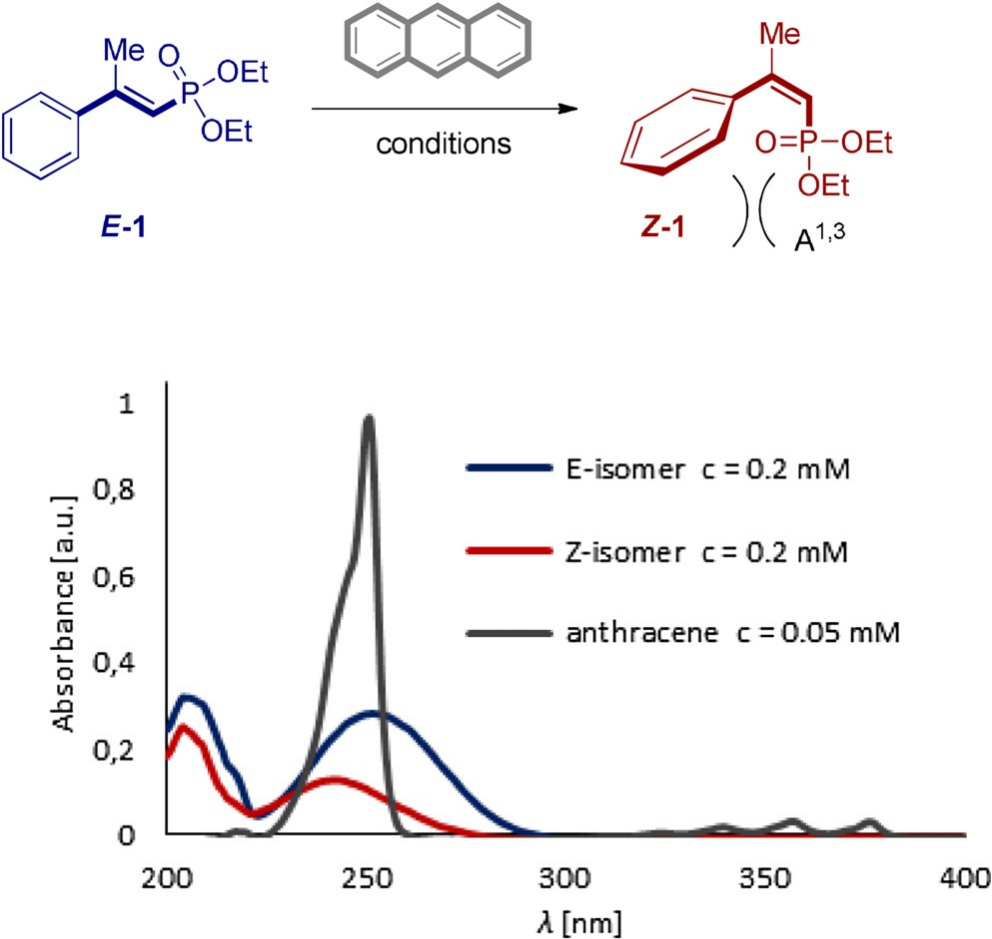

Entry	Photocatalyst	Irradiation wavelength [nm]	Isolated yield [%]	*Z*:*E* ratio^[b]^
1	Ir(ppy)_3_	450	quant.	13:87
2	(−)‐riboflavin	402	quant.	66:34
3	benzil	402	98	25:75
4	thioxanthone	402	94	86:14
5	benzophenone	365	quant.	86:14
6	anthracene	365	quant.	92:08

[a] All reactions were performed on a 0.1 mmol scale using 5 mol % catalyst in 1.5 mL MeCN at ambient temperature for 18 h. [b] Determined by ^1^H NMR and confirmed by ^31^P NMR spectroscopy.

To investigate the scope and limitations of this method, a series of *E*‐vinyl phosphonates modified at R^1^, R^2^ and the aryl ring fragments were investigated under the optimised conditions (Figure 1). Gratifyingly, *para*‐substitution was well tolerated (***Z*** **‐1**–**Z**‐**7**) with selectivities up to *Z*:*E* 94:6 having been observed. The *meta*‐bromo product ***Z*** **‐8** was also generated with a high degree of selectivity (*Z*:*E* 91:09) and, like ***Z*** **‐4**, provides a versatile building block for subsequent derivatisation via cross coupling. Variation in R^2^ had no impact on the reaction efficiency or selectivity as is evident from ***Z*** **‐9** and ***Z*** **‐10** (92:8 and 90:10, respectively). Importantly, highly electron‐rich aryl systems such as those found in ***Z*** **‐11** and ***Z*** **‐12** were found to compromise selectivity. This is likely a consequence of contributing resonance forms partially facilitating bond rotation and relaxation to the starting *E*‐isomer.

**Figure 1:**
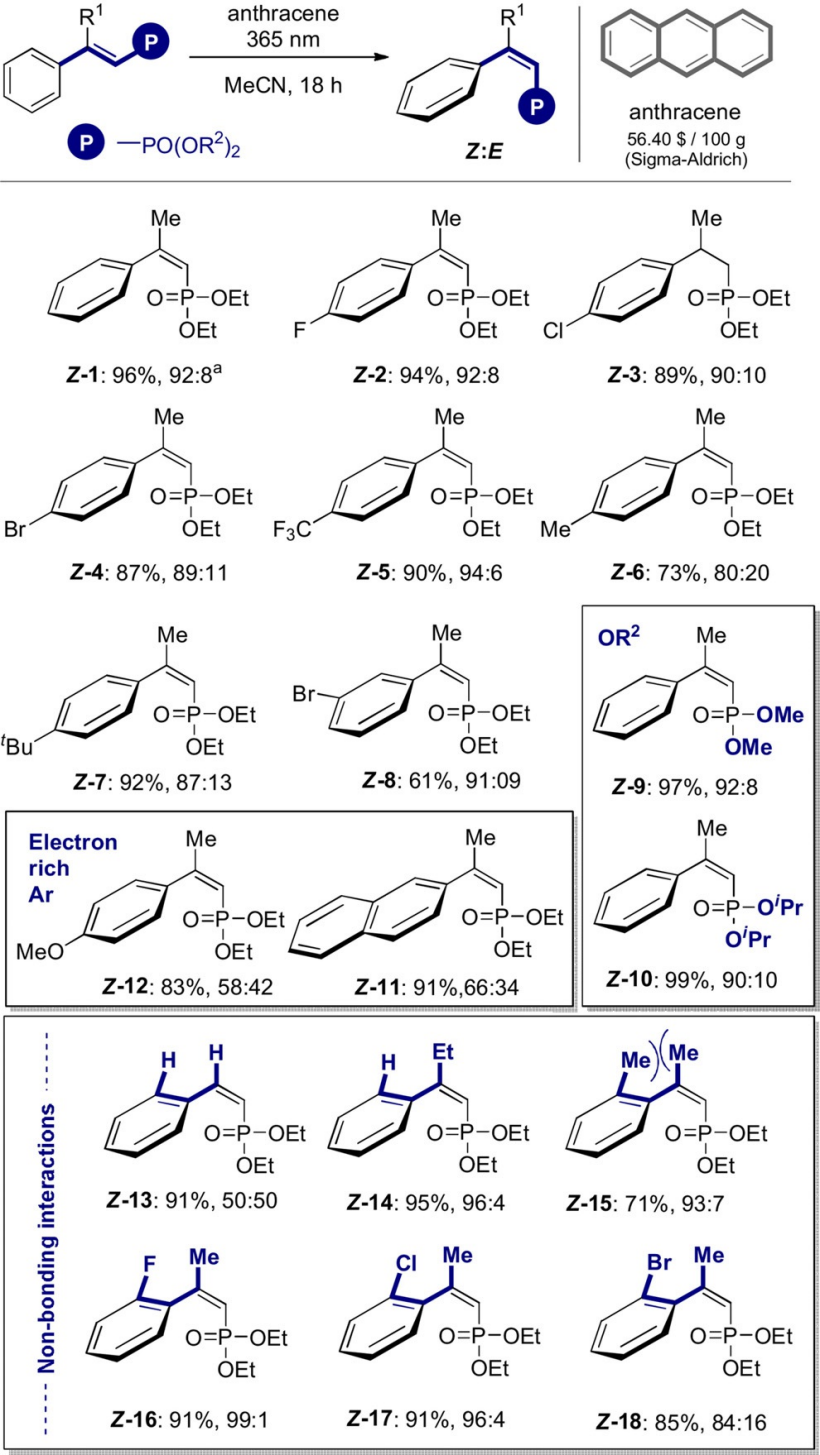
Development of the *E* → *Z* isomerisation of vinyl phosphonates via selective energy transfer catalysis using anthracene. Reactions performed on a 0.1 mmol scale. ^a^Scale up: 95 %, 92:8 *Z*:*E* (0.4 mmol) and 97 %, 83:17 *Z*:*E* (1.0 mmol). For full details see the Supporting Information.

To interrogate the influence of R^1^ in inhibiting re‐conjugation of the chromophore, ***Z*** **‐13** was prepared as a control. Although of no significance for the subsequent reduction, this substrate illustrates the structural importance of R^1^ (photostationary composition 1:1). By extension, augmenting the size of R^1^ from Me to Et caused a small increase in *Z*:*E* ratio from 92:8 to 96:4 (***Z*** **‐14**). The *ortho*‐methyl derivative also furnished synthetically useful levels of selectivity (93:7, ***Z*** **‐15**). Indeed, the value of *ortho*‐substitution is evident from a screen of the halogens (***Z*** **‐16**, **17** and **18**, up to 99:1 *Z*:*E*). Having established a robust protocol to facilitate the geometrical *E* → *Z* isomerisation of vinyl phosphonates via energy transfer catalysis, the reaction was then coupled to a stereospecific hydrogenation (Figure 2). For that purpose, both the *E*‐ and *Z*‐activated alkenes were exposed to Rh^I^‐catalysed hydrogenation conditions using a single enantiomer of (*Sc,Sp*)‐Walphos.^21^

**Figure 2:**
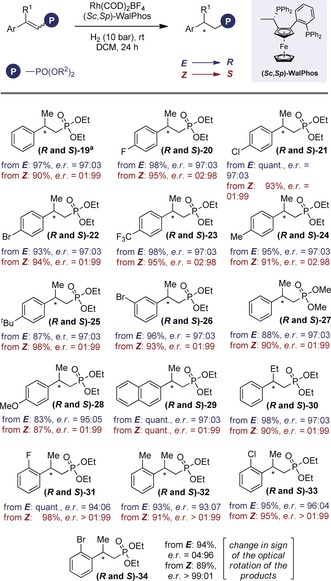
Stereospecific reduction of *E*‐ and *Z*‐activated alkenes. Reactions performed on a 0.1 mmol scale. ^a^Scale up to 1.0 mmol from *E* (99 %, 97:03 *e.r*.); from *Z* (96 %, 01:99 *e.r*.). For full details see the Supporting Information.

To our delight, the reaction proved to be highly stereospecific delivering ***R*** **‐** or ***S*** **‐19** with equal and opposite selectivity [*R*:*S* 97:03 *e.r*. versus 01:99 *e.r*., from ***E*** **‐1** and **Z‐1**, respectively]. This trend proved to be general, resulting in a highly stereospecific reduction, where enantioselectivity encoded at the catalyst level could be inverted by geometrical isomerisation (Figure 3). In the case of the *para*‐substituted derivatives **20**–**25**, the *E*‐substrates furnished the *R*‐products in 97:03 *e.r*. and the *Z*‐substrates led to the opposite *S*‐antipode in up to 01:99 *e.r*. This stereospecificity was mirrored by the *meta*‐bromo derivative ***R*** **‐** and ***S*** **‐26** (97:03 and 01:99, respectively). Modifying R^2^ was extremely well tolerated (**27**) as was introduction of an electron rich *para*‐OMe substituent (**28**) or extended π‐system (**29**). Switching R^1^ from Me to Et had no impact on selectivity (**30**); *R*:*S* 97:03 *e.r*. versus 01:99 *e.r*.) and the introduction of *ortho*‐substituents **31**–**33** was equally well tolerated (up to >99:1 from the *Z*‐alkene). Interestingly, the sign of the optical rotation observed with the *o*‐Br derivative **34** were inverted, again with reduction of the Z‐isomer out‐competing the *E*‐substrate (>01:99).

**Figure 3:**
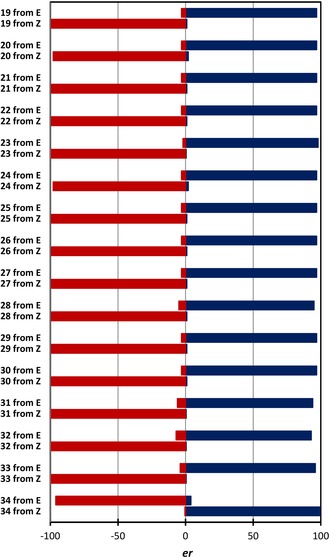
Graphical representation of the degree and direction of stereoselectivity.

Since *Z*‐configured substrates generally gave higher enantioselectivities, the reaction with the opposite catalyst antipode was performed (Figure 4, top). Geometrical isomerisation constitutes a practical advantage enabling both stereoisomers to be prepared in an optically pure manner (99:1 and 1:99 *e.r*.). Furthermore, the reaction could be executed in a one‐pot fashion from the *E*‐isomer as is demonstrated in Figure 4 (bottom). In conclusion, an enabling approach to enantiodivergence is disclosed that is predicated on efficient geometrical isomerisation of vinyl phosphonates via selective energy transfer catalysis using anthracene (up to *Z*:*E* 99:1). Although valuable in its own right, this reaction provides a stimulus prior to stereospecific hydrogenation allowing both product enantiomers to be generated from a single optically active catalyst (up to 99:1 *e.r*.). This additional dimension to external asymmetric induction may prove to be expansive, particularly where the opposite catalyst enantiomer is prohibitively expensive or is unobtainable.

**Figure 4:**
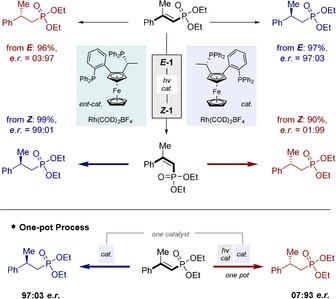
Top: Exploring both catalysts and alkene geometries. Bottom: A one‐pot, stereodivergent synthesis.

## Experimental Section

Full details are provided in the Supporting Information.

## Conflict of interest

The authors declare no conflict of interest.

## Supporting information

As a service to our authors and readers, this journal provides supporting information supplied by the authors. Such materials are peer reviewed and may be re‐organized for online delivery, but are not copy‐edited or typeset. Technical support issues arising from supporting information (other than missing files) should be addressed to the authors.

Supplementary
