# Supplementary material for: Inverting External Asymmetric Induction via Selective Energy Transfer Catalysis: A Strategy to β‐Chiral Phosphonate Antipodes
Source: Angew Chem Int Ed Engl. 2019 Nov 26;59(1):330–4. doi: 10.1002/anie.201911651 (PMC6972697; doi:10.1002/anie.201911651)
Supplement: Supplementary file 1 — Supplementary [file ANIE-59-330-s001.pdf]

Angewandte Chemie
Eine Zeitschrift der Gesellschaft Deutscher Chemiker

Supporting Information

Inverting External Asymmetric Induction via Selective Energy Transfer Catalysis: A Strategy to β-Chiral Phosphonate Antipodes

*Carina Onneken, Kathrin Bussmann, and Ryan Gilmour**

anie_201911651_sm_miscellaneous_information.pdf

Supporting Information

General Information	1
Experimental Section	3
HPLC traces	37
NMR Spectra of Key Compouds	55
References	138

General Information

All chemicals were purchased as reagent grade and used without further purification. Solvents for purification (extraction and chromatography) were purchased as technical grade and distilled on the rotary evaporator prior to use. For column chromatography SiO2 (40-63 µm for Flash-Chromatography, VWR Chemicals) was used as stationary phase. Analytical thin layer chromatography (TLC) was performed on aluminium foil pre-coated with SiO2-60 F254 (Merck) and visualised with a UV-lamp (254 nm) and KMnO4 or CAM solution. Concentration in vacuo was performed at ~10 mbar and 40 °C, drying at ~10-2 mbar and room temperature. NMR spectra were measured by the NMR service of the Organisch-Chemisches Institut, Westfälische Wilhelms-Universität Münster on a Bruker BZH 200/52, Bruker AV300, Bruker AV400, Agilent DD2 500 or an Agilent DD2 600 spectrometer at room temperature. The chemical shifts are referenced to the residual solvent peak as internal standard. Spectra of other nuclides as ^{13}C and ^{31}P are referenced according to the proton resonance of TMS as the primary reference for the unified chemical shift scale. The resonance multiplicity is abbreviated as: s (singlet), d (doublet), t (triplet), q (quadruplet), p (pentet), sext (sextet), hept (heptet), m (multiplet) and b (broad). Assignments of unknown compounds are based on DEPT, COSY (HH), HMBC, HSQC and NOESY spectra. Alkene configuration is assigned based on coupling constants and NOESY spectra. IR spectra were recorded on a PerkinElmer 100 FT-IR spectrometer, selected absorption bands are reported in wavenumbers (cm^{-1}) and intensities are reported as: w (weak), m (medium), s (strong) and b (broad). High-resolution mass spectra (HR-ESI) were measured by the MS service of the Organisch-Chemisches Institut, Westfälische Wilhelms-Universität Münster. Optical rotations were measured on a JASCO P2000 polarimeter. Enantiomeric ratios were determined on an Agilent Infinity 1260 HPLC system using a diode array detector (DAD). The chiral stationary phase and the eluent ratio of *n*-hexane and *i*-propanol is given specifically for each compound. The column temperature measured 25 to 35 °C. UV/vis absorption spectra were measured on an *Agilent Cary 60 UV-Vis Spectrophotometer*, baseline correction was performed with the corresponding solvent. Isomerisation reactions were performed utilizing a UVA LED (365 nm, emission spectrum: Figure S1), a *Winger WEPUV3-S2 UV Power LED Star* (402 nm, emission spectrum: Figure S2) *and a Winger WEPRB3-S1 Power LED Star royalblue* (450 nm, emission spectrum: Figure S3). The distance between the reaction vessels and the UV-lamp was set at approximately 0.5 cm for all reactions. Hydrogenation reactions were performed in a Berghof High Pressure Reactor using hydrogen gas.

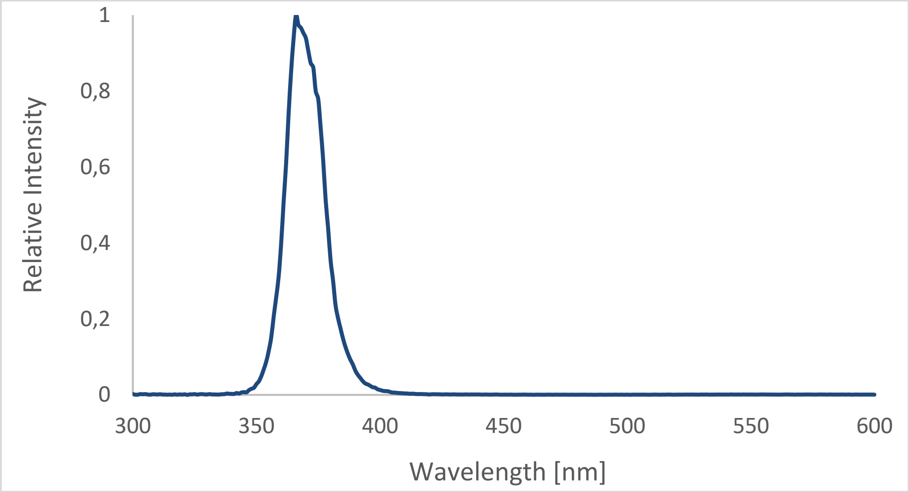

Figure S1: Emission spectrum of the utilised UVA LED (365 nm).

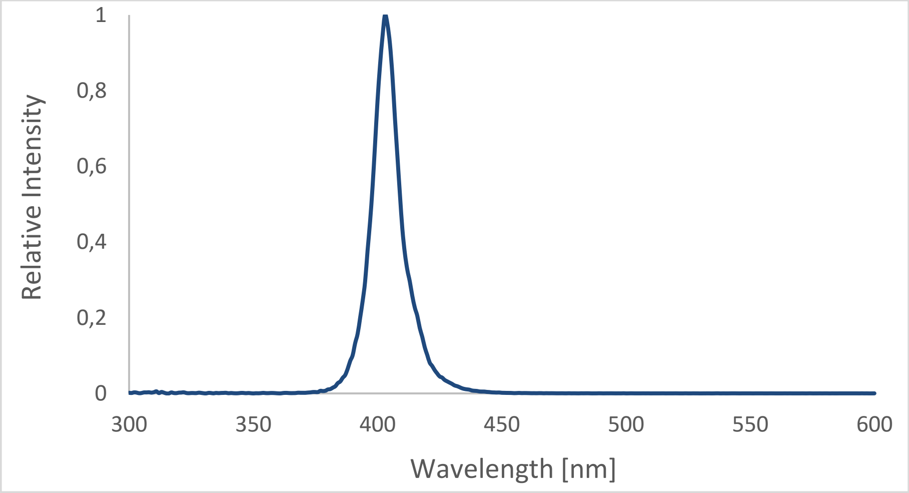

Figure S2: Emission spectrum of the utilised Winger WEPUV3-S2 UV Power LED Star (402 nm).

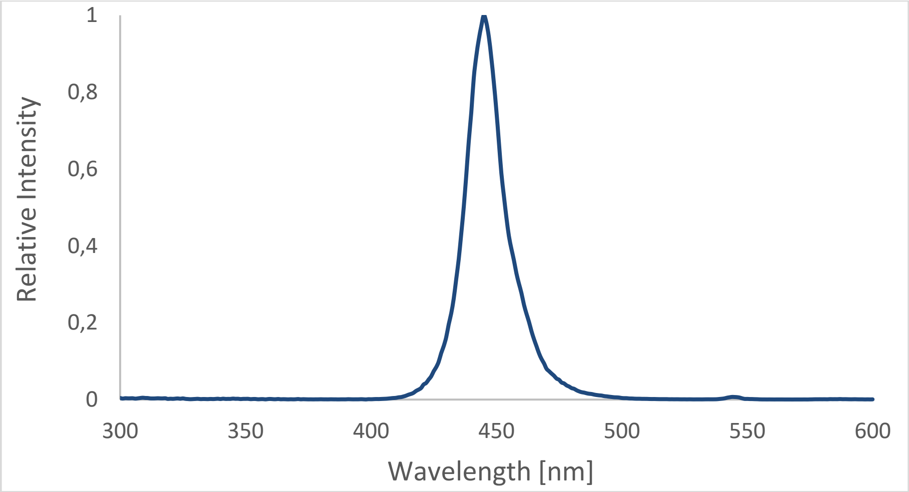

Figure S3: Emission spectrum of the utilised Winger WEPRB3-S1 Power LED Star royalblue (450 nm).

Experimental Section

Procedures and Analytical Data

General Procedure A for the Synthesis of *E*-Vinylphosphonates

In a flame-dried schlenk tube under argon atmosphere sodium hydride (60% in mineral oil) was dissolved in dry tetrahydrofuran (10 mL) at 0 °C. Tetraalkyl methylenediphosphonate was added dropwise and the solution was stirred under argon atmosphere at 0 °C for 1 h. The specified acetophenone derivative was added via syringe and the solution was heated at 60 °C and stirred for 3 days. After the mixture was cooled to room temperature, water (30 mL) and ethyl acetate (40 mL) were added. The layers were separated and the aqueous layer was extracted with ethyl acetate (3 x 20 mL). The combined organic layers were dried over anhydrous magnesium sulfate and the solvent was removed in vacuo. Purification by column chromatography yielded the *E*-vinylphosphonates.

Diethyl (*E*)-(2-phenylprop-1-en-1-yl)phosphonate (*E*-1):

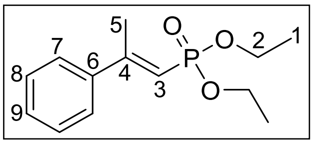

Prepared according to **general procedure A** from acetophenone (1.00 mL, 8.57 mmol, 1.00 eq.) and tetraethyl methylenediphosphonate (2.70 mL, 10.9 mmol, 1.27 eq.) with sodium hydride (60% in mineral oil, 0.50 g, 12.5 mmol, 1.46 eq.) in 66 h. Purification by column chromatography (SiO_2, *n*-pentane/ethyl acetate: 6/4) yielded *E*-vinylphosphonate ***E*-1** as yellow oil (0.87 g, 3.42 mmol, 40%).

$\mathbf{R_f}$ = 0.25 (SiO_2, *n*-pentane/ethyl acetate: 3/7); **^{1}H NMR** (600 MHz, $CDCl_3$): δ = 7.48 - 7.44 (m, 2H, H7), 7.38 - 7.33 (m, 3H, H8, H9), 5.89 (dd, *J* = 16.5, 1.1 Hz, 1H, H3), 4.12 (dq, *J* = 7.9, 7.1 Hz, 4H, H2), 2.50 (dd, *J* = 3.3, 1.1 Hz, 3H, H5), 1.35 (t, *J* = 7.1 Hz, 6H, H1) ppm; **^{13}C NMR** (151 MHz, $CDCl_3$): δ = 158.3 (d, J_{CP} = 8.0 Hz, C4), 141.9 (d, J_{CP} = 23.6 Hz, C6), 129.2 (C8/C9), 128.6 (C8/C9), 126.1 (C7), 113.7 (d, J_{CP} = 190.2 Hz, C3), 61.6 (d, J_{CP} = 5.6 Hz, C2), 19.4 (d, J_{CP} = 7.0 Hz, C5), 16.5 (d, J_{CP} = 6.4 Hz, C1) ppm; **^{31}P NMR** (243 MHz, $CDCl_3$): δ = 18.14 ppm; **IR (ATR):** $\tilde{\nu}$ = 3474 (w), 2982 (w), 2906 (w), 1608 (m), 1574 (w), 1495 (w), 1444 (m), 1391 (w), 1325 (w), 1244 (m), 1163 (w), 1098 (w), 1050 (s), 1024 (s), 957 (s), 823 (m), 790 (m), 753 (m), 696 (m) cm^{-1}; **HR-ESI-MS**: *m/z*: 277.0969 ($[M+Na]^+$, calcd. for $C_{13}H_{19}NaO_3P^+$: 277.0964), 531.2039 ($[M_2+Na]^+$, cald. for $C_{26}H_{38}NaO_6P_2^+$: 531.2036); analytical data in agreement with literature.[1]

Diethyl (*E*)-(2-(4-fluorophenyl)prop-1-en-1-yl)phosphonate (*E*-2):

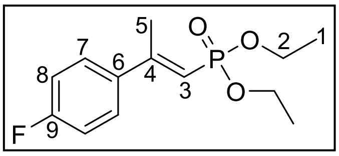

Prepared according to **general procedure A** from 4'-fluoroacetophenone (0.18 mL, 1.48 mmol, 1.00 eq.) and tetraethyl methylenediphosphonate (0.48 mL, 1.95 mmol, 1.32 eq.) with sodium hydride (60% in mineral oil, 0.10 g, 2.50 mmol, 1.69 eq.) in 65 h. Purification by column chromatography (SiO_2, cyclohexane/ethyl acetate: 4/1) yielded *E*-vinylphosphonate ***E*-2** as yellow oil (251 mg, 0.92 mmol, 62%).

R_f = 0.47 (SiO_2, ethyl acetate); **^{1}H NMR** (500 MHz, $CDCl_3$): δ = 7.46 - 7.39 (m, 2H, H7), 7.04 - 6.98 (m, 2H, H8), 5.82 (ddd, *J* = 16.1, 1.2, 0.7 Hz, 1H, H3), 4.10 (dqd, *J* = 7.7, 7.1, 0.6 Hz, 4H, H2), 2.45 (dt, *J* = 3.3, 0.8 Hz, 3H, H5), 1.32 (tt, *J* = 7.0, 0.6 Hz, 6H, H1) ppm; **^{13}C NMR** (126 MHz, $CDCl_3$): δ = 163.4 (d, J_{CF} = 249.2 Hz, C9), 156.9 (d, J_{CP} = 8.3 Hz, C4), 137.9 (dd, J_{CP} = 24.0, J_{CF} = 3.4 Hz, C6), 127.9 (d, J_{CF} = 8.3 Hz, C7), 115.5 (d, J_{CF} = 21.5 Hz, C8), 113.6 (dd, J_{CP} = 191.0, J_{CF} = 1.4 Hz, C3), 61.6 (d, J_{CP} = 5.7 Hz, C2), 19.4 (d, J_{CP} = 7.0 Hz, C5), 16.5 (d, J_{CP} = 6.5 Hz, C1) ppm; **^{19}F NMR** (282 MHz, $CDCl_3$): δ = -112.34 (tt, *J* = 8.4, 5.3 Hz) ppm; **^{31}P NMR** (202 MHz, $CDCl_3$): δ = 17.85 (ddtq, *J* = 15.8, 11.7, 7.9, 3.7 Hz) ppm; **IR (ATR):** $\tilde{\nu}$ = 3457 (w), 2983 (w), 2917 (w), 2850 (w), 1601 (m), 1509 (m), 1444 (w), 1392 (w), 1323 (w), 1236 (s), 1163 (m), 1098 (w), 1051 (s), 1024 (s), 957 (s), 810 (s), 745 (w), 718 (w) cm^{-1}; **HR-ESI-MS**: *m/z*: 295.0879 ($[M+Na]^+$, calcd. for $C_{13}H_{18}FNaO_3P^+$: 295.0870), 567.1847 ($[M_2+Na]^+$, cald. for $C_{26}H_{36}F_2NaO_6P_2^+$: 567.1847); analytical data in agreement with literature.[1]

Diethyl (*E*)-(2-(4-chlorophenyl)prop-1-en-1-yl)phosphonate (*E*-3):

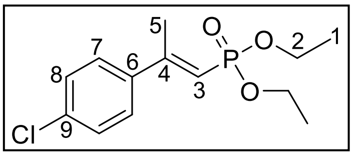

Prepared according to **general procedure A** from 4'-chloroacetophenone (0.19 mL, 1.49 mmol, 1.00 eq.) and tetraethyl methylenediphosphonate (0.48 mL, 1.95 mmol, 1.31 eq.) with sodium hydride (60% in mineral oil, 0.10 g, 2.50 mmol, 1.68 eq.) in 64 h. Purification by column chromatography (SiO_2, *n*-pentane/ethyl acetate: 4/1) yielded *E*-vinylphosphonate ***E*-3** as yellow oil (210 mg, 0.73 mmol, 49%).

R_f = 0.31 (SiO_2, *n*-penatne/ethyl acetate: 1/1); **^{1}H NMR** (500 MHz, $CDCl_3$): δ = 7.40 - 7.35 (m, 2H, H8), 7.33 - 7.28 (m, 2H, H7), 5.86 (dd, *J* = 16.1, 0.9 Hz, 1H, H3), 4.10 (p, *J* = 7.1 Hz, 4H, H2), 2.45 (dd, *J* = 3.3, 0.8 Hz, 3H, H5), 1.33 (t, *J* = 7.1 Hz, 6H, H1) ppm; **^{13}C NMR** (126 MHz, $CDCl_3$): δ = 156.7 (d, J_{CP} = 8.3 Hz, C4), 140.2 (d, J_{CP} = 24.0 Hz, C6), 135.2 (C9), 128.7 (d, J_{CP} = 0.7 Hz, C7), 127.4 (C8), 114.2 (d, J_{CP} = 190.8 Hz, C3), 61.7 (d, J_{CP} = 5.6 Hz, C2), 19.2 (d, J_{CP} = 6.9 Hz, C5), 16.5 (d, J_{CP} = 6.4 Hz, C1) ppm; **^{31}P NMR** (202 MHz, $CDCl_3$): δ = 17.61 ppm; **IR (ATR):** $\tilde{\nu}$ = 3477 (w), 2983 (w), 2906 (w), 1610 (m), 1592 (w), 1489 (m), 1443 (w), 1392 (w), 1321 (w), 1246 (s), 1163 (w), 1094 (m), 1051 (s), 1025 (s), 958 (s), 812 (s), 767 (m), 742 (w), 662 (m) cm^{-1}; **HR-ESI-MS**: *m/z*: 311.0576 ($[M+Na]^+$, calcd. for $C_{13}H_{18}ClNaO_3P^+$:

311.0574), 599.1256 ([M_2+Na]$^+$, cald. for $C_{26}H_{36}Cl_2NaO_6P_2^+$: 599.1256); analytical data in agreement with literature.[1]

Diethyl (*E*)-(2-(4-bromophenyl)prop-1-en-1-yl)phosphonate (*E*-4):

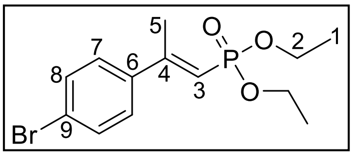

Prepared according to **general procedure A** from 4'-bromoacetophenone (298 mg, 1.51 mmol, 1.00 eq.) and tetraethyl methylenediphosphonate (0.48 mL, 1.95 mmol, 1.29 eq.) with sodium hydride (60% in mineral oil, 0.10 g, 2.50 mmol, 1.67 eq.) in 64 h. Purification by column chromatography (SiO_2, *n*-pentane/ethyl acetate: 4/1) yielded *E*-vinylphosphonate ***E*-4** as yellow oil (186 mg, 0.56 mmol, 37%).

R$_f$ = 0.54 (SiO_2, ethyl acetate); **^{1}H NMR** (500 MHz, $CDCl_3$): δ = 7.49 - 7.45 (m, 2H, H7), 7.34 - 7.30 (m, 2H, H8), 5.87 (dq, *J* = 15.9, 1.1 Hz, 1H, H3), 4.11 (dq, *J* = 7.8, 7.1 Hz, 4H, H2), 2.45 (dd, *J* = 3.3, 1.1 Hz, 3H, H5), 1.34 (td, *J* = 7.1, 0.5 Hz, 6H, H1) ppm; **^{13}C NMR** (126 MHz, $CDCl_3$): δ = 156.8 (d, J_{CP} = 8.2 Hz, C4), 140.8 (d, J_{CP} = 24.1 Hz, C6), 131.7 (d, J_{CP} = 0.7 Hz, C7), 127.7 (C8), 123.5 (C9), 114.3 (d, J_{CP} = 190.7 Hz, C3), 61.7 (d, J_{CP} = 5.6 Hz, C2), 19.2 (d, J_{CP} = 7.0 Hz, C5), 16.5 (d, J_{CP} = 6.3 Hz, C1) ppm; **^{31}P NMR** (202 MHz, $CDCl_3$): δ = 17.57 (dddp, *J* = 15.8, 11.7, 8.0, 4.2 Hz) ppm; **IR (ATR):** $\tilde{\nu}$ = 3456 (w), 2982 (w), 2908 (w), 1609 (m), 1585 (w), 1486 (m), 1442 (w), 1394 (m), 1320 (w), 1246 (s), 1163 (w), 1097 (m), 1050 (s), 1024 (s), 959 (s), 808 (s), 761 (m) cm^{-1}; **HR-ESI-MS**: *m/z*: 355.0086 ([*M*+Na]$^+$, calcd. for $C_{13}H_{18}BrNaO_3P^+$: 355.0069), 689.0243 ([M_2+Na]$^+$, cald. for $C_{26}H_{36}Br_2NaO_6P_2^+$: 689.0226); analytical data in agreement with literature.[1]

Diethyl (*E*)-(2-(4-(trifluoromethyl)phenyl)prop-1-en-1-yl)phosphonate (*E*-5):

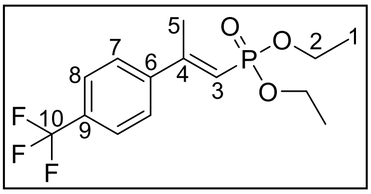

Prepared according to **general procedure A** from 4'-trifluoromethylacetophenone (282 mg, 1.50 mmol, 1.00 eq.) and tetraethyl methylenediphosphonate (0.48 mL, 1.95 mmol, 1.30 eq.) with sodium hydride (60% in mineral oil, 0.10 g, 2.50 mmol, 1.67 eq.) in 67 h. Purification by column chromatography (SiO_2, cyclohexane/ethyl acetate: 7/3) yielded *E*-vinylphosphonate ***E*-5** as yellow oil (119 mg, 0.37 mmol, 25%).

R$_f$ = 0.53 (SiO_2, ethyl acetate); **^{1}H NMR** (500 MHz, $CDCl_3$): δ = 7.61 (d, *J* = 8.7 Hz, 2H, H8), 7.54 (d, *J* = 8.2 Hz, 2H, H7), 5.92 (dq, *J* = 15.7, 1.1 Hz, 1H, H3), 4.13 (dq, *J* = 8.1, 7.0 Hz, 4H, H2), 2.50 (dd, *J* = 3.3, 1.1 Hz, 3H, H5), 1.35 (t, *J* = 7.1 Hz, 6H, H1) ppm; **^{13}C NMR** (126 MHz, $CDCl_3$): δ = 156.6 (d, J_{CP} = 8.2 Hz, C4), 145.5 (d, J_{CP} = 24.0, C6), 131.1 (q, J_{CF} = 32.7 Hz, C9), 126.5 (C7), 125.6 (q, J_{CF} = 3.8 Hz, C8), 124.0 (q, J_{CF} = 272.1 Hz, C10), 116.1 (d, J_{CP} = 190.3 Hz, C3), 61.8 (d, J_{CP} = 5.6 Hz, C2), 19.4 (d, J_{CP} = 6.9 Hz, C5), 16.5 (d, J_{CP} = 6.4 Hz, C1) ppm; **^{19}F NMR** (470 MHz, $CDCl_3$): δ = -62.78 ppm; **^{31}P NMR** (202 MHz, $CDCl_3$):

δ = 16.95 (ddtq, *J* = 15.8, 11.7, 7.9, 4.2, 3.7 Hz) ppm; **IR (ATR):** $\tilde{\nu}$ = 3506 (w), 2983 (w), 2917 (w), 2850 (w), 1615 (w), 1572 (w), 1445 (w), 1409 (w), 1393 (w), 1322 (s), 1248 (m), 1166 (m), 1119 (s), 1080 (w), 1052 (s), 1025 (s), 959 (s), 859 (w), 825 (m), 732 (w) cm^{-1}; **HR-ESI-MS**: *m/z*: 345.0843 ([*M*+Na]$^+$, calcd. for $C_{14}H_{18}F_3NaO_3P^+$: 345.0838), 667.1794 ([M_2+Na]$^+$, cald. for $C_{28}H_{36}F_6NaO_6P_2^+$: 667.1784); analytical data in agreement with literature.[1]

Diethyl (*E*)-(2-(4-tolyl)prop-1-en-1-yl)phosphonate (*E*-6):

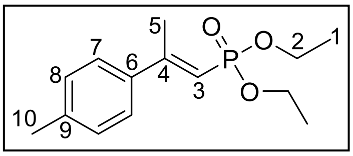

Prepared according to **general procedure A** from 4'-methylacetophenone (0.20 mL, 1.49 mmol, 1.00 eq.) and tetraethyl methylenediphosphonate (0.48 mL, 1.95 mmol, 1.31 eq.) with sodium hydride (60% in mineral oil, 0.10 g, 2.50 mmol, 1.68 eq.) in 64 h. Purification by column chromatography (SiO_2, *n*-pentane/ethyl acetate: 85/15) yielded *E*-vinylphosphonate ***E*-6** as yellow oil (211 mg, 0.79 mmol, 53%).

R_f = 0.32 (SiO_2, *n*-penatne/ethyl acetate: 1/1); **^{1}H NMR** (500 MHz, $CDCl_3$): δ = 7.39 - 7.35 (m, 2H, H8), 7.18 - 7.14 (m, 2H, H7), 5.87 (dq, *J* = 16.6, 0.9 Hz, 1H, H3), 4.11 (dq, *J* = 7.9, 7.1 Hz, 4H, H2), 2.47 (dd, *J* = 3.2, 1.0 Hz, 3H, H5), 2.35 (s, 3H, H10), 1.36 - 1.32 (m, 6H, H1) ppm; **^{13}C NMR** (126 MHz, $CDCl_3$): δ = 158.1 (d, J_{CP} = 8.1 Hz, C4), 139.4 (C9), 138.9 (d, J_{CP} = 23.7 Hz, C6), 129.3 (d, J_{CP} = 0.8 Hz, C7), 126.0 (C8), 112.5 (d, J_{CP} = 190.6 Hz, C3), 61.5 (d, J_{CP} = 5.6 Hz, C2), 21.3 (C10), 19.3 (d, J_{CP} = 7.1 Hz, C5), 16.5 (d, J_{CP} = 6.5 Hz, C1) ppm; **^{31}P NMR** (202 MHz, $CDCl_3$): δ = 18.55 (ddtq, *J* = 15.8, 11.6, 7.9, 3.8 Hz) ppm; **IR (ATR):** $\tilde{\nu}$ = 3448 (w), 2981 (w), 2920 (w), 1606 (m), 1567 (w), 1513 (w), 1443 (w), 1391 (w), 1322 (w), 1246 (s), 1164 (w), 1097 (w), 1051 (s), 1025 (s), 957 (s), 831 (m), 806 (s), 746 (w) cm^{-1}; **HR-ESI-MS**: *m/z*: 291.1121 ([*M*+Na]$^+$, calcd. for $C_{14}H_{21}NaO_3P^+$: 291.1121), 559.2341 ([M_2+Na]$^+$, cald. for $C_{28}H_{42}NaO_6P_2^+$: 559.2349); analytical data in agreement with literature.[1]

Diethyl (*E*)-(2-(4-(*tert*-butyl)phenyl)prop-1-en-1-yl)phosphonate (*E*-7):

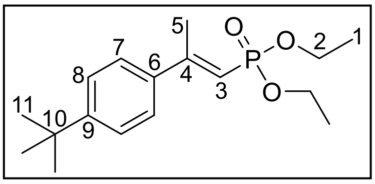

Prepared according to **general procedure A** from 4'-*tert*-butylacetophenone (0.27 mL, 1.48 mmol, 1.00 eq.) and tetraethyl methylenediphosphonate (0.48 mL, 1.95 mmol, 1.32 eq.) with sodium hydride (60% in mineral oil, 0.10 g, 2.50 mmol, 1.69 eq.) in 67 h. Purification by column chromatography (SiO_2, cyclohexane/ethyl acetate: 9/1) yielded *E*-vinylphosphonate ***E*-7** as yellow oil (151 mg, 0.49 mmol, 33%).

R_f = 0.63 (SiO_2, ethyl acetate); **^{1}H NMR** (500 MHz, $CDCl_3$): δ = 7.44 - 7.41 (m, 2H, H8), 7.40 - 7.34 (m, 2H, H7), 5.90 (dq, *J* = 16.6, 1.1 Hz, 1H, H3), 4.11 (dq, *J* = 8.0, 7.1 Hz, 4H, H2), 2.49 (dd, *J* = 3.3, 1.0 Hz, 3H, H5), 1.37 - 1.30 (m, 15H, H1, H11) ppm; **^{13}C NMR** (126 MHz, $CDCl_3$): δ = 158.0 (d, J_{CP} = 8.0 Hz, C4), 152.6

(C9), 138.8 (d, J_{CP} = 23.7 Hz, C6), 125.9 (C8), 125.5 (d, J_{CP} = 0.4 Hz, C7), 112.6 (d, J_{CP} = 190.4 Hz, C3), 61.5 (d, J_{CP} = 5.5 Hz, C2), 34.8 (C10), 31.3 (C11), 19.2 (d, J_{CP} = 7.1 Hz, C5), 16.5 (d, J_{CP} = 6.5 Hz, C1) ppm; **^{31}P NMR** (202 MHz, $CDCl_3$): δ = 18.57 (ddtq, *J* = 15.8, 11.6, 8.0, 4.4, 3.8 Hz) ppm; **IR (ATR):** $\tilde{\nu}$ = 3457 (w), 2963 (m), 2905 (w), 2869 (w), 1607 (m), 1557 (w), 1509 (w), 1443 (w), 1393 (w), 1364 (w), 1323 (w), 1247 (s), 1163 (w), 1097 (w), 1051 (s), 1025 (s), 956 (s), 820 (s), 740 (w), 670 (w) cm^{-1}; **HR-ESI-MS**: *m/z*: 333.1594 ([*M*+Na]$^+$, calcd. for $C_{17}H_{27}NaO_3P^+$: 333.1590), 643.3299 ([*M*$_2$+Na]$^+$, cald. for $C_{34}H_{54}NaO_6P_2^+$: 643.3288).

Diethyl (*E*)-(2-(3-bromophenyl)prop-1-en-1-yl)phosphonate (*E*-8):

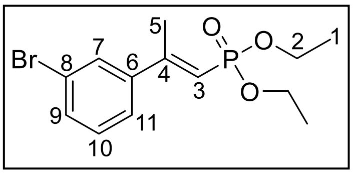

Prepared according to **general procedure A** from 3′-bromoacetophenone (0.46 mL, 3.47 mmol, 1.00 eq.) and tetraethyl methylenediphosphonate (1.04 mL, 4.53 mmol, 1.31 eq.) with sodium hydride (60% in mineral oil, 234 g, 5.85 mmol, 1.69 eq.) in 65 h. Purification by column chromatography (SiO_2, cyclohexane/ethyl acetate: 9/1) yielded *E*-vinylphosphonate ***E*-8** as yellow oil (678 mg, 2.04 mmol, 59%).

R$_f$ = 0.34 (SiO_2, ethyl acetate); **^{1}H NMR** (600 MHz, $CDCl_3$): δ = 7.59 (t, *J* = 1.9 Hz, 1H, H7), 7.48 (ddd, *J* = 7.9, 1.9, 1.0 Hz, 1H, H9), 7.38 (ddd, *J* = 7.8, 1.8, 1.0 Hz, 1H, H11), 7.24 (t, *J* = 7.9 Hz, 1H, H10), 5.88 (dt, *J* = 15.9, 1.1 Hz, 1H, H3), 4.13 (p, *J* = 7.1 Hz, 4H, H2), 2.47 (dd, *J* = 3.3, 1.0 Hz, 3H, H5), 1.36 (t, *J* = 7.0 Hz, 6H, H1) ppm; **^{13}C NMR** (151 MHz, $CDCl_3$): δ = 156.6 (d, J_{CP} = 8.3 Hz, C4), 144.1 (d, J_{CP} = 23.9 Hz, C6), 132.1 (C9), 130.2 (C10), 129.3 (C7), 124.8 (C11), 122.8 (d, J_{CP} = 1.1 Hz, C8), 115.1 (d, J_{CP} = 190.3 Hz, C3), 61.8 (d, J_{CP} = 5.6 Hz, C2), 19.4 (d, J_{CP} = 6.9 Hz, C5), 16.5 (d, J_{CP} = 6.5 Hz, C1) ppm; **^{31}P NMR** (243 MHz, $CDCl_3$): δ = 17.25 (ddq, *J* = 15.8, 8.0, 3.9 Hz) ppm; **IR (ATR):** $\tilde{\nu}$ = 3475 (b), 2982 (w), 2929 (w), 1721 (w), 1611 (w), 1591 (w), 1558 (w), 1477 (w), 1443 (w), 1392 (w), 1368 (w), 1319 (w), 1299 (w), 1243 (m), 1163 (w), 1097 (w), 1048 (s), 1019 (s), 954 (s), 884 (w), 931 (s), 779 (s), 746 (m), 688 (m), 669 (m) cm^{-1}; **HR-ESI-MS**: *m/z*: 355.0077 ([*M*+Na]$^+$, calcd. for $C_{13}H_{18}BrNaO_3P^+$: 355.0069), 689.0251 ([*M*$_2$+Na]$^+$, cald. for $C_{26}H_{36}Br_2NaO_6P_2^+$: 689.0226).

Dimethyl (*E*)-(2-phenylprop-1-en-1-yl)phosphonate (*E*-9):

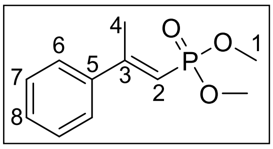

Prepared according to **general procedure A** from acetophenone (0.17 mL, 1.46 mmol, 1.00 eq.) and tetramethyl methylene-diphosphonate (0.45 g, 1.94 mmol, 1.33 eq.) with sodium hydride (60% in mineral oil, 0.10 g, 2.50 mmol, 1.71 eq.) in 68 h. Purification by column chromatography (SiO_2, cyclohexane/ethyl acetate: 3/2) yielded *E*-vinylphosphonate ***E*-9** as orange oil (136 mg, 0.60 mmol, 41%).

$\mathbf{R_f}$ = 0.49 (SiO_2, ethyl acetate); **^{1}H NMR** (600 MHz, $CDCl_3$): δ = 7.47 - 7.43 (m, 2H, H6), 7.36 - 7.33 (m, 3H, H7, H8), 5.85 (dq, J = 16.8, 1.1 Hz, 1H, H2), 3.75 (d, J = 11.2 Hz, 6H, H1), 2.49 (dd, J = 3.3, 1.1 Hz, 3H, H4) ppm; **^{13}C NMR** (151 MHz, $CDCl_3$): δ = 159.3 (d, J_{CP} = 8.1 Hz, C3), 141.7 (d, J_{CP} = 23.8 Hz, C5), 129.4 (C8), 128.6 (C7), 126.1 (C6), 112.1 (d, J_{CP} = 191.0 Hz, C2), 52.20 (d, J_{CP} = 5.5 Hz, C1), 19.4 (d, J_{CP} = 7.2 Hz, C4) ppm; **^{31}P NMR** (243 MHz, $CDCl_3$): δ = 20.95 ppm; **IR (ATR):** $\tilde{\nu}$ = 3457 (w), 2951 (w), 2849 (w), 1609 (m), 1574 (w), 1495 (w), 1445 (w), 1380 (w), 1326 (w), 1246 (m), 1182 (m), 1024 (s), 980 (m), 843 (m), 815 (s), 753 (m), 695 (m) cm^{-1}; **HR-ESI-MS**: *m/z*: 249.0651 ([M+Na]$^+$, calcd. for $C_{11}H_{15}NaO_3P^+$: 249.0651), 475.1407 ([M_2+Na]$^+$, cald. for $C_{22}H_{30}NaO_6P_2^+$: 475.1410).

Diisopropyl (*E*)-(2-phenylprop-1-en-1-yl)phosphonate (*E*-10):

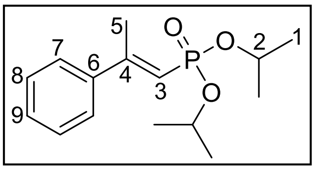

Prepared according to **general procedure A** from acetophenone (0.17 mL, 1.46 mmol, 1.00 eq.) and tetraisopropyl methylenediphosphonate (0.62 mL, 1.95 mmol, 1.34 eq.) with sodium hydride (60% in mineral oil, 0.10 g, 2.50 mmol, 1.71 eq.) in 66 h. Purification by column chromatography (SiO_2, cyclohexane/ethyl acetate: 7/3) yielded *E*-vinylphosphonate ***E*-10** as orange oil (154 mg, 0.55 mmol, 38%).

$\mathbf{R_f}$ = 0.27 (SiO_2, cyclohexane/ethyl acetate: 1/1); **^{1}H NMR** (500 MHz, $CDCl_3$): δ = 7.47 - 7.43 (m, 2H, H7), 7.38 - 7.32 (m, 3H, H8, H9), 5.91 (dq, J = 16.4, 1.0 Hz, 1H, H3), 4.71 (dhept, J = 8.2, 6.2 Hz, 2H, H2), 2.49 (dd, J = 3.3, 1.1 Hz, 3H, H5), 1.34 (dd, J = 15.2, 6.2 Hz, 12H, H1) ppm; **^{13}C NMR** (126 MHz, $CDCl_3$): δ = 157.1 (d, J_{CP} = 8.0 Hz, C4), 142.2 (d, J_{CP} = 23.7 Hz, C6), 129.1 (C9), 128.6 (d, J_{CP} = 0.7 Hz, C7), 126.1 (C8), 115.5 (d, J_{CP} = 190.7 Hz, C3), 70.2 (d, J_{CP} = 5.7 Hz, C2), 24.2 (dd, J_{CP} = 10.9, 4.3 Hz, C1), 19.3 (d, J_{CP} = 6.9 Hz, C5) ppm; **^{31}P NMR** (202 MHz, $CDCl_3$): δ = 15.93 (dtd, J = 16.3, 8.2, 3.8 Hz) ppm; **IR (ATR):** $\tilde{\nu}$ = 3456 (w), 2978 (w), 2933 (w), 1607 (w), 1575 (w), 1495 (w), 1445 (w), 1385 (w), 1374 (w), 1324 (w), 1245 (m), 1178 (w), 1141 (w), 1107 (m), 976 (s), 886 (m), 814 (m), 753 (m), 695 (m) cm^{-1}; **HR-ESI-MS**: *m/z*: 305.1288 ([M+Na]$^+$, calcd. for $C_{15}H_{23}NaO_3P^+$: 305.1277), 587.2675 ([M_2+Na]$^+$, cald. for $C_{30}H_{46}NaO_6P_2^+$: 587.2662); analytical data in agreement with literature.[2]

Diethyl (*E*)-(2-(naphthalen-2-yl)prop-1-en-1-yl)phosphonate (*E*-11):

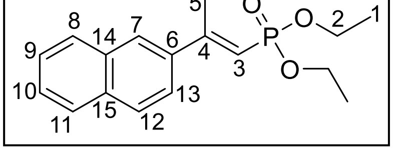

Prepared according to **general procedure A** from 2-acetonaphtone (255 mg, 1.50 mmol, 1.00 eq.) and tetraethyl methylenediphosphonate (0.48 mL, 1.95 mmol, 1.30 eq.) with sodium hydride (60% in mineral oil, 0.10 g, 2.50 mmol, 1.67 eq.) in

67 h. Purification by column chromatography (SiO_2, cyclohexane/ethyl acetate: 4/1) yielded *E*-vinylphosphonate ***E*-11** as orange oil (153 mg, 0.50 mmol, 33%).

R_f = 0.67 (SiO_2, ethyl acetate); **^{1}H NMR** (600 MHz, $CDCl_3$): δ = 7.93 (d, *J* = 1.6 Hz, 1H, H7), 7.85 (dd, *J* = 5.6, 4.1 Hz, 1H, H8), 7.84 - 7.81 (m, 2H, H11, H12), 7.60 (dd, *J* = 8.6, 1.9 Hz, 1H, H13), 7.52 - 7.47 (m, 2H, H9, H10), 6.06 (dd, *J* = 16.3, 1.0 Hz, 1H, H3), 4.16 (p, *J* = 7.1 Hz, 4H, H2), 2.62 (dd, *J* = 3.2, 1.0 Hz, 3H, H5), 1.38 (t, *J* = 7.1 Hz, 6H, H1) ppm; **^{13}C NMR** (151 MHz, $CDCl_3$): δ = 157.9 (d, J_{CP} = 8.1 Hz, C4), 139.0 (d, J_{CP} = 23.7 Hz, C6), 133.7 (C15), 133.2 (C14), 128.7 (C8), 128.3 (C12), 127.7 (C11), 126.9 (C10), 126.7 (C9), 125.7 (C7), 123.7 (C13), 114.1 (d, J_{CP} = 190.6 Hz, C3), 61.7 (d, J_{CP} = 5.6 Hz, C2), 19.4 (d, J_{CP} = 7.0 Hz, C5), 16.6 (d, J_{CP} = 6.6 Hz, C1) ppm; **^{31}P NMR** (243 MHz, $CDCl_3$): δ = 18.25 ppm; **IR (ATR):** $\tilde{\nu}$ = 3477 (w), 3056 (w), 2981 (w), 2926 (w), 2852 (w), 1607 (m), 1574 (w), 1505 (w), 1442 (w), 1389 (w), 1367 (w), 1349 (w), 1316 (w), 1240 (s), 1163 (w), 1131 (w), 1097 (w), 1050 (s), 1024 (s), 957 (s), 882 (m), 813 (s), 748 (m) cm^{-1}; **HR-ESI-MS**: *m/z*: 327.1117 ([*M*+Na]$^+$, calcd. for $C_{17}H_{21}NaO_3P^+$: 327.1121), 631.2350 ([M_2+Na]$^+$, cald. for $C_{34}H_{42}NaO_6P_2^+$: 631.2349); analytical data in agreement with literature.[1]

Diethyl (*E*)-(2-(4-methoxyphenyl)prop-1-en-1-yl)phosphonate (*E*-12):

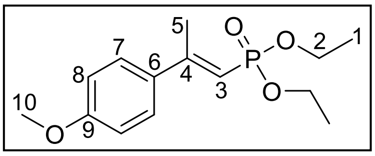

Prepared according to **general procedure A** from 4'-methoxyacetophenone (225 mg, 1.50 mmol, 1.00 eq.) and tetraethyl methylenediphosphonate (0.48 mL, 1.95 mmol, 1.30 eq.) with sodium hydride (60% in mineral oil, 0.10 g, 2.50 mmol, 1.67 eq.) in 67 h. Purification by column chromatography (SiO_2, cyclohexane/ethyl acetate: 4/1) yielded *E*-vinylphosphonate ***E*-12** as orange oil (174 mg, 0.61 mmol, 41%).

R_f = 0.55 (SiO_2, ethyl acetate); **^{1}H NMR** (500 MHz, $CDCl_3$): δ = 7.44 - 7.40 (m, 2H, H7), 6.88 - 6.84 (m, 2H, H8), 5.83 (dd, *J* = 16.4, 1.0 Hz, 1H, H3), 4.10 (dq, *J* = 7.9, 7.1 Hz, 4H, H2), 3.80 (s, 3H, H10), 2.46 (dd, *J* = 3.2, 0.9 Hz, 3H, H5), 1.33 (t, *J* = 7.1 Hz, 6H, H1) ppm; **^{13}C NMR** (126 MHz, $CDCl_3$): δ = 160.6 (C9), 157.4 (d, J_{CP} = 8.2 Hz, C4), 133.9 (d, J_{CP} = 23.9 Hz, C6), 127.4 (C7), 113.9 (C8), 111.3 (d, J_{CP} = 191.7 Hz, C3), 61.5 (d, J_{CP} = 5.5 Hz, C2), 55.4, 19.1 (d, J_{CP} = 7.1 Hz, C5), 16.5 (d, J_{CP} = 6.4 Hz, C1) ppm; **^{31}P NMR** (202 MHz, $CDCl_3$): δ = 18.88 ppm; **IR (ATR):** $\tilde{\nu}$ = 3447 (w), 2981 (w), 2907 (w), 2840 (w), 1602 (s), 1571 (w), 1513 (s), 1442 (m), 1418 (w), 1391 (w), 1327 (w), 1291 (m), 1245 (s), 1182 (m), 1164 (m), 1097 (w), 1050 (s), 1025 (s), 957 (s), 822 (s), 805 (s), 744 (w), 714 (w) cm^{-1}; **HR-ESI-MS**: *m/z*: 307.1079 ([*M*+Na]$^+$, calcd. for $C_{14}H_{21}NaO_4P^+$: 307.1070), 591.2266 ([M_2+Na]$^+$, cald. for $C_{28}H_{42}NaO_8P_2^+$: 591.2247); analytical data in agreement with literature.[1]

Diethyl (*E*)-styrylphosphonate (*E*-13):

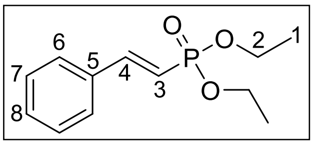

Prepared according to **general procedure A** from benzaldehyde (0.15 mL, 1.50 mmol, 1.00 eq.) and tetraethyl methylenediphosphonate (0.48 mL, 2.50 mmol, 1.67 eq.) with sodium hydride (60% in mineral oil, 0.10 g, 1.95 mmol, 1.30 eq.) in 63 h. Purification by column chromatography (SiO_2, cyclohexane/ethyl acetate: 1/9) yielded *E*-vinylphosphonate ***E*-13** as yellow oil (288 mg, 1.20 mmol, 80%).

R_f = 0.59 (SiO_2, ethyl acetate); **^{1}H NMR** (500 MHz, $CDCl_3$): δ = 7.55 - 7.45 (m, 3H, H4, H6), 7.41 - 7.34 (m, 3H, H7, H8), 6.25 (t, *J* = 17.6 Hz, 1H, H3), 4.18 - 4.07 (m, 4H, H2), 1.34 (td, *J* = 7.1, 0.6 Hz, 6H, H1) ppm; **^{13}C NMR** (126 MHz, $CDCl_3$): δ = 148.8 (d, J_{CP} = 6.7 Hz, C4), 135.0 (d, J_{CP} = 23.2 Hz, C5), 130.3 (C8), 129.0 (d, J_{CP} = 0.9 Hz, C7), 127.8 (d, J_{CP} = 0.9 Hz, C6), 114.1 (dd, J_{CP} = 191.2, 1.0 Hz, C3), 61.9 (d, J_{CP} = 5.5 Hz, C2), 16.5 (d, J_{CP} = 6.4 Hz, C1) ppm; **^{31}P NMR** (202 MHz, $CDCl_3$): δ = 19.46 (ddt, *J* = 22.6, 17.7, 8.1 Hz) ppm; **IR (ATR):** $\tilde{\nu}$ = 3476 (w), 1982 (w), 2909 (w), 2852 (w), 1616 (m), 1577 (w), 1449 (w), 1392 (w), 1243 (m), 1197 (w), 1163 (w), 1097 (w), 1051 (s), 1023 (s), 960 (s), 857 (m), 828 (m), 790 (m), 743 (m), 691 (m) cm^{-1}; **HR-ESI-MS**: *m/z*: 263.0814 ([*M*+Na]$^+$, calcd. for $C_{12}H_{17}NaO_3P^+$: 263.0808), 503.1729 ([M_2+Na]$^+$, cald. for $C_{24}H_{34}NaO_6P_2^+$: 503.1723); analytical data in agreement with literature.[3]

Diethyl (*E*)-(2-phenylbut-1-en-1-yl)phosphonate (*E*-14):

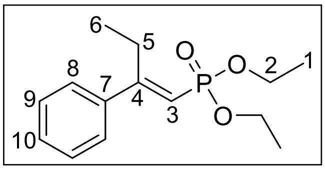

Prepared according to **general procedure A** from propiophenone (0.20 mL, 1.50 mmol, 1.00 eq.) and tetraethyl methylenediphosphonate (0.48 mL, 1.95 mmol, 1.30 eq.) with sodium hydride (60% in mineral oil, 0.10 g, 2.50 mmol, 1.67 eq.) in 66 h. Purification by column chromatography (SiO_2, cyclohexane/ethyl acetate: 6/4) yielded *E*-vinylphosphonate ***E*-14** as yellow oil (158 mg, 0.59 mmol, 39%).

R_f = 0.50 (SiO_2, ethyl acetate); **^{1}H NMR** (500 MHz, $CDCl_3$): δ = 7.42 - 7.38 (m, 2H, H8), 7.38 - 7.33 (m, 3H, H9, H10), 5.74 (d, *J* = 17.2 Hz, 1H, H3), 4.12 (p, *J* = 7.1 Hz, 4H, H2), 3.00 (qd, *J* = 7.4, 2.1 Hz, 2H, H5), 1.34 (t, *J* = 7.1 Hz, 6H, H1), 1.02 (t, *J* = 7.5 Hz, 3H, H6) ppm; **^{13}C NMR** (126 MHz, $CDCl_3$): δ = 165.0 (d, J_{CP} = 8.7 Hz, C4), 140.8 (d, J_{CP} = 23.8 Hz, C7), 129.0 (C9/C10), 128.6 (C9/C10), 126.65 (C8), 113.4 (d, J_{CP} = 189.8 Hz, C3), 61.6 (d, J_{CP} = 5.6 Hz, C2), 26.0 (d, J_{CP} = 6.8 Hz, C5), 16.5 (d, J_{CP} = 6.5 Hz, C1), 13.6 (d, J_{CP} = 2.3 Hz, C6) ppm; **^{31}P NMR** (202 MHz, $CDCl_3$): δ = 17.86 (dp, *J* = 15.8, 7.7 Hz) ppm; **IR (ATR):** $\tilde{\nu}$ = 3503 (w), 2978 (w), 2934 (w), 1606 (m), 1574 (w), 1495 (w), 1444 (w), 1391 (w), 1306 (w), 1241 (s), 1163 (w), 1097 (w), 1050 (s), 1024 (s), 958 (s), 835 (m), 788 (m), 760 (m), 698 (m) cm^{-1}; **HR-ESI-MS**: *m/z*: 291.1128 ([*M*+Na]$^+$, calcd. for $C_{14}H_{21}NaO_3P^+$: 291.1121), 559.2362 ([M_2+Na]$^+$, cald. for $C_{28}H_{42}NaO_6P_2^+$: 559.2349); analytical data in agreement with literature.[1]

Diethyl (*E*)-(2-(2-tolyl)prop-1-en-1-yl)phosphonate (*E*-15):

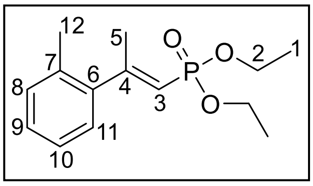

Prepared according to **general procedure A** from 2'-methylacetophenone (1.30 mL, 9.88 mmol, 3.06 eq.) and tetraethyl methylenediphosphonate (0.80 mL, 3.23 mmol, 1.00 eq.) with sodium hydride (60% in mineral oil, 0.17 g, 4.25 mmol, 1.32 eq.) in tetrahydrofuran (20 mL) in 67 h. Purification by column chromatography (SiO_2, cyclohexane/ethyl acetate: 9/1) yielded *E*-vinylphosphonate ***E*-15** as red oil (433 mg, 1.61 mmol, 50%).

R_f = 0.65 (SiO_2, ethyl acetate); **^{1}H NMR** (600 MHz, $CDCl_3$): δ = 7.21 - 7.13 (m, 3H, H8, H9, H10), 7.06 (dd, *J* = 7.3, 1.4 Hz, 1H, H11), 5.50 (dq, *J* = 18.7, 1.2 Hz, 1H, H3), 4.13 (dqd, *J* = 7.8, 7.1, 0.7 Hz, 4H, H2), 2.37 (dd, *J* = 3.3, 1.2 Hz, 3H, H5), 2.29 (s, 3H, H12), 1.35 (td, *J* = 7.0, 0.4 Hz, 6H, H1) ppm; **^{13}C NMR** (151 MHz, $CDCl_3$): δ = 161.3 (d, J_{CP} = 6.8 Hz, C4), 144.3 (d, J_{CP} = 23.4 Hz, C6), 133.5 (d, J_{CP} = 0.9 Hz, C7), 130.5 (C8), 127.8 (C9), 126.8 (d, J_{CP} = 1.4 Hz, C11), 125.9 (C10), 116.4 (d, J_{CP} = 184.1 Hz, C3), 61.5 (d, J_{CP} = 5.5 Hz, C2), 22.0 (d, J_{CP} = 6.6 Hz (C5)), 19.7 (C12), 16.5 (d, J_{CP} = 6.4 Hz, C1) ppm; **^{31}P NMR** (243 MHz, $CDCl_3$): δ = 17.00 ppm; **IR (ATR):** $\tilde{\nu}$ = 3456 (w), 2981 (w), 2910 (w), 1622 (w), 1487 (w), 1443 (w), 1391 (w), 1313 (w), 1247 (s), 1163 (w), 1097 (w), 1051 (s), 1025 (s), 957 (s), 833 (m), 794 (m), 749 (m) cm^{-1}; **HR-ESI-MS**: *m/z*: 291.1147 ($[M+Na]^+$, calcd. for $C_{14}H_{21}NaO_3P^+$: 291.1121), 559.2362 ($[M_2+Na]^+$, cald. for $C_{28}H_{42}NaO_6P_2^+$: 559.2349); analytical data in agreement with literature.[1]

Diethyl (*E*)-(2-(2-fluorophenyl)prop-1-en-1-yl)phosphonate (*E*-16):

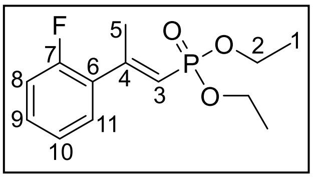

Prepared according to **general procedure A** from 2'-fluoroacetophenone (0.18 mL, 1.48 mmol, 1.00 eq.) and tetraethyl methylenediphosphonate (0.48 mL, 1.95 mmol, 1.32 eq.) with sodium hydride (60% in mineral oil, 0.10 g, 2.50 mmol, 1.69 eq.) in 68 h. Purification by column chromatography (SiO_2, cyclohexane/ethyl acetate: 4/1) yielded *E*-vinylphosphonate ***E*-16** as orange oil (229 mg, 0.84 mmol, 57%).

R_f = 0.51 (SiO_2, ethyl acetate); **^{1}H NMR** (600 MHz, $CDCl_3$): δ = 7.31 - 7.24 (m, 2H, H9, H11), 7.11 (td, *J* = 7.6, 1.2 Hz, 1H, H10), 7.05 (ddd, *J* = 11.0, 8.2, 1.1 Hz, 1H, H8), 5.77 (dd, *J* = 17.0, 1.2 Hz, 1H, H3), 4.15 - 4.10 (m, 4H, H2), 2.46 (dt, *J* = 3.3, 1.4 Hz, 3H, H5), 1.35 (t, *J* = 7.1 Hz, 6H, H1) ppm; **^{13}C NMR** (151 MHz, $CDCl_3$): δ = 159.4 (d, J_{CF} = 248.6 Hz, C7), 154.9 (dd, J_{CP} = 8.6, J_{CF} = 1.1 Hz, C4), 131.1 (dd, J_{CP} = 24.5, J_{CF} = 13.3 Hz, C6), 130.2 (d, J_{CP} = 8.5 Hz, C9), 129.1 (dd, J_{CF} = 3.7, J_{CP} = 1.1 Hz, C11), 124.3 (d, J_{CF} = 3.6 Hz, C10), 117.9 (dd, J_{CP} = 186.7, J_{CF} = 2.9 Hz, C3), 116.2 (d, J_{CF} = 22.6 Hz, C8), 61.7 (d, J_{CP} = 5.6 Hz, C2), 20.8 (dd, J_{CP} = 6.9, J_{CF} = 3.6 Hz, C5), 16.5 (d, J_{CP} = 6.4 Hz, C1) ppm; **^{19}F NMR** (564 MHz, $CDCl_3$): δ = -114.47 (dddd, *J* = 12.9, 7.2, 3.5, 1.8 Hz) ppm; **^{31}P NMR** (243 MHz, $CDCl_3$): δ = 16.69 (dddp, *J* = 15.9, 11.7, 7.9, 4.2 Hz) ppm; **IR (ATR):** $\tilde{\nu}$ = 3457 (w), 2982 (w), 2908 (w), 1615 (w), 1575 (w), 1488 (m), 1446 (m), 1392 (w), 1323 (w), 1247 (s), 1206 (w), 1163 (w), 1112 (w), 1098 (w), 1051 (s), 1024 (s), 960 (s),

836 (m), 805 (m), 759 (m) cm^{-1}; **HR-ESI-MS**: *m/z*: 295.0869 ([*M*+Na]$^+$, calcd. for $C_{13}H_{18}FNaO_3P^+$: 295.0870), 567.1853 ([*M₂*+Na]$^+$, cald. for $C_{26}H_{36}F_2NaO_6P_2^+$: 567.1847).

Diethyl (*E*)-(2-(2-chlorophenyl)prop-1-en-1-yl)phosphonate (*E*-17):

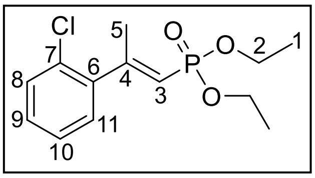

Prepared according to **general procedure A** from 2'-chloroacetophenone (0.20 mL, 1.54 mmol, 1.00 eq.) and tetraethyl methylenediphosphonate (0.48 mL, 1.95 mmol, 1.27 eq.) with sodium hydride (60% in mineral oil, 0.10 g, 2.50 mmol, 1.62 eq.) in 65 h. Purification by column chromatography (SiO_2, cyclohexane/ethyl acetate: 7/3) yielded *E*-vinylphosphonate ***E*-17** as orange oil (258 mg, 0.89 mmol, 58%).

R$_f$ = 0.46 (SiO_2, ethyl acetate); **^{1}H NMR** (600 MHz, $CDCl_3$): δ = 7.38 - 7.34 (m, 1H, H8), 7.25 - 7.21 (m, 2H, H9, H10), 7.18 - 7.14 (m, 1H, H11), 5.58 (dq, *J* = 17.6, 1.2 Hz, 1H, H3), 4.16 - 4.10 (m, 4H, H2), 2.42 (dd, *J* = 3.4, 1.2 Hz, 3H, H5), 1.35 (td, *J* = 7.1, 0.5 Hz, 6H, H1) ppm; **^{13}C NMR** (151 MHz, $CDCl_3$): δ = 158.6 (d, J_{CP} = 8.0 Hz, C4), 143.1 (d, J_{CP} = 24.5 Hz, C6), 130.9 (d, J_{CP} = 1.4 Hz, C7), 129.9 (C8), 129.2 (C9), 128.7 (d, J_{CP} = 1.7 Hz, C11), 127.0 (C10), 118.0 (d, J_{CP} = 183.8 Hz, C3), 61.7 (d, J_{CP} = 5.5 Hz, C2), 21.4 (d, J_{CP} = 6.5 Hz, C5), 16.5 (d, J_{CP} = 6.4 Hz, C1) ppm; **^{31}P NMR** (243 MHz, $CDCl_3$): δ = 16.31 (ddqt, *J* = 15.2, 11.4, 7.5, 3.4 Hz) ppm; **IR (ATR):** $\tilde{\nu}$ = 3467 (w), 2982 (w), 2907 (w), 1624 (w), 1590 (w), 1565 (w), 1471 (w), 1429 (w), 1392 (w), 1316 (w), 1246 (s), 1163 (w), 1129 (w), 1096 (w), 1023 (s), 959 (s), 837 (m), 822 (m), 793 (w), 756 (m), 682 (w) cm^{-1}; **HR-ESI-MS**: *m/z*: 311.0588 ([*M*+Na]$^+$, calcd. for $C_{13}H_{18}ClNaO_3P^+$: 311.0574), 599.1261 ([*M₂*+Na]$^+$, cald. for $C_{26}H_{36}Cl_2NaO_6P_2^+$: 599.1256).

Diethyl (*E*)-(2-(2-bromophenyl)prop-1-en-1-yl)phosphonate (*E*-18):

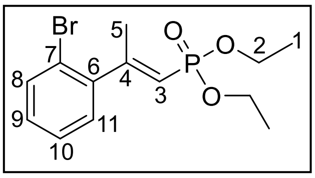

Prepared according to **general procedure A** from 2'-bromoacetophenone (0.20 mL, 1.48 mmol, 1.00 eq.) and tetraethyl methylenediphosphonate (0.48 mL, 1.95 mmol, 1.32 eq.) with sodium hydride (60% in mineral oil, 0.10 g, 2.50 mmol, 1.69 eq.) in 65 h. Purification by column chromatography (SiO_2, cyclohexane/ethyl acetate: 7/3) yielded *E*-vinylphosphonate ***E*-18** as orange oil (309 mg, 0.93 mmol, 63%).

R$_f$ = 0.46 (SiO_2, ethyl acetate); **^{1}H NMR** (600 MHz, $CDCl_3$): δ = 7.56 - 7.54 (m, 1H, H8), 7.28 (td, *J* = 7.5, 1.2 Hz, 1H, H10), 7.17 - 7.13 (m, 2H, H9, H11), 5.55 (dq, *J* = 17.6, 1.2 Hz, 1H, H3), 4.14 (dq, *J* = 8.0, 7.1 Hz, 4H, H2), 2.41 (dd, *J* = 3.4, 1.3 Hz, 3H, H5), 1.36 (td, *J* = 7.1, 0.5 Hz, 6H, H1) ppm; **^{13}C NMR** (151 MHz, $CDCl_3$): δ = 160.0 (d, J_{CP} = 7.9 Hz, C4), 145.1 (d, J_{CP} = 24.6 Hz, C6), 133.1 (C8), 129.3 (C9), 128.6 (d, J_{CP} = 1.7 Hz, C11), 127.6 (C10), 120.3 (d, J_{CP} = 1.6 Hz, C7), 117.9 (d, J_{CP} = 183.4 Hz, C3), 61.7 (d, J_{CP} = 5.5 Hz, C2), 21.6 (d, J_{CP} = 6.3 Hz, C5), 16.5 (d, J_{CP} = 6.5 Hz, C1) ppm; **^{31}P NMR** (243 MHz, $CDCl_3$):

δ = 16.28 (dddq, J = 23.5, 11.6, 7.9, 4.0 Hz) ppm; **IR (ATR):** $\tilde{\nu}$ = 3475 (w), 2982 (w), 2907 (w), 1624 (w), 1561 (w), 1467 (w), 1425 (w), 1392 (w), 1315 (w), 1246 (s), 1163 (w), 1097 (w), 1051 (s), 1023 (s), 958 (s), 837 (m), 821 (m), 792 (w), 754 (m), 661 (w) cm^{-1}; **HR-ESI-MS**: *m/z*: 355.0074 ($[M+Na]^+$, calcd. for $C_{13}H_{18}BrNaO_3P^+$: 355.0069), 689.0236 ($[M_2+Na]^+$, cald. for $C_{26}H_{36}Br_2NaO_6P_2^+$: 689.0226).

General procedure B for the Isomerisation of *E*-Vinylphosphonates

The specified *E*-vinylphosphonate (0.10 mmol, 1.00 eq.) and anthracene (0.9 mg, 0.005 mmol, 5 mol%) were dissolved in acetonitrile (1.5 mL) and the solution was stirred under UV light irradiation at 365 nm at ambient temperature for 18 h. After removal of the solvent, *E*- and *Z*-isomer were isolated by column chromatography. Yields were determined by mass recovery; *Z*:*E* ratios were determined by integration of peaks in the ^{31}P NMR spectrum and confirmed by integration the olefinic proton peaks in the ^{1}H NMR spectrum of both isomers.

Diethyl (*Z*)-(2-phenylprop-1-en-1-yl)phosphonate (*Z*-1):

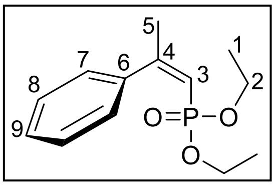

According to **general procedure B**, ***E*-1** (25.0 mg, 0.10 mmol) was converted to ***Z*-1**. Purification by column chromatography (SiO_2, ethyl acetate) yielded a yellow oil (24.9 mg, quant., ***Z*-1**: ***E*-1** = 92:8).

R_f = 0.21 (SiO_2, *n*-pentane/ethyl acetate: 3/7); **^{1}H NMR** (600 MHz, $CDCl_3$): δ = 7.40 - 7.37 (m, 2H, H7), 7.36 - 7.32 (m, 2H, H8), 7.32 - 7.29 (m, 1H, H9), 5.72 (dq, J = 17.3, 1.4 Hz, 1H, H3), 3.88 - 3.72 (m, 4H, H2), 2.23 (dd, J = 1.3, 1.1 Hz, 3H, H5), 1.07 (t, J = 7.0, 0.5 Hz, 6H, H1) ppm; **^{13}C NMR** (151 MHz, $CDCl_3$): δ = 159.1 (d, J_{CP} = 4.6 Hz, C4), 140.7 (d, J_{CP} = 7.5 Hz, C6), 128.4 (C9), 128.0 (C8), 127.4 (d, J_{CP} = 1.7 Hz, C7), 115.0 (d, J_{CP} = 191.6 Hz, C3), 61.4 (d, J_{CP} = 6.0 Hz, C2), 28.5 (d, J_{CP} = 23.1 Hz, C5), 16.2 (d, J_{CP} = 6.8 Hz, C1) ppm; **^{31}P NMR** (243 MHz, $CDCl_3$): δ = 16.13 ppm; **IR (ATR):** $\tilde{\nu}$ = 3456 (w), 2980 (w), 2908 (w), 1671 (w), 1599 (w), 1494 (w), 1441 (w), 1391 (w), 1237 (s), 1190 (w), 1163 (w), 1098 (w), 1052 (s), 1027 (s), 959 (s), 853 (m), 790 (m), 765 (m), 701 (m) cm^{-1}; **HR-ESI-MS**: *m/z*: 277.0970 ($[M+Na]^+$, calcd. for $C_{13}H_{19}NaO_3P^+$: 277.0964), 531.2032 ($[M_2+Na]^+$, cald. for $C_{26}H_{38}NaO_6P_2^+$: 531.2036); analytical data in agreement with literature.[4]

A representative example of the reaction was repeated on a 1 mmol scale:

E-vinylphosphonate **1** (254.6 mg, 1.00 mmol, 1.00 eq.) and anthracene (8.9 mg, 0.05 mmol, 5 mol%) were dissolved in acetonitrile (10 mL) and the solution was stirred under UV light irradiation at 365 nm at ambient temperature for 18 h. The solvent was removed *in vacuo* and purification by column chromatography (SiO_2, ethyl acetate) yielded a yellow oil (2246.5 mg, 97%, ***Z*-1**: ***E*-1** = 83:17).

Diethyl (*Z*)-(2-(4-fluorophenyl)prop-1-en-1-yl)phosphonate (*Z*-2):

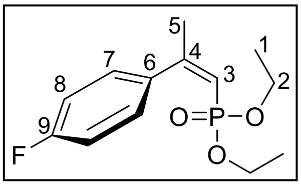

According to **general procedure B**, ***E*-2** (27.2 mg, 0.10 mmol) was converted to ***Z*-2**. Purification by column chromatography (SiO_2, cyclohexane/ethyl acetate: 1/9) yielded a yellow oil (25.6 mg, 94%, ***Z*-2**: ***E*-2** = 92:8).

R$_f$ = 0.43 (SiO_2, ethyl acetate); **^{1}H NMR** (500 MHz, $CDCl_3$): δ = 7.38 (ddd, *J* = 8.3, 5.3, 2.6 Hz, 2H, H7), 7.06 - 6.99 (m, 2H, H8), 5.71 (dq, *J* = 16.9, 1.3 Hz, 1H, H3), 3.90 - 3.75 (m, 4H, H2), 2.23 - 2.18 (m, 3H, H5), 1.13 - 1.07 (m, 6H, H1) ppm; **^{13}CNMR** (126 MHz, $CDCl_3$): δ = 162.8 (d, J_{CF} = 247.6 Hz, C9), 157.9 (d, J_{CP} = 4.4 Hz, C4), 136.5 (dd, J_{CP} = 7.6, J_{CF} = 3.4 Hz, C6), 129.4 (dd, J_{CF} = 8.2, J_{CP} = 1.8 Hz, C7), 115.3 (d, J_{CP} = 191.2 Hz, C3), 115.0 (d, J_{CF} = 21.5 Hz, C8), 61.5 (d, J_{CP} = 6.0 Hz, C2), 28.5 (d, J_{CP} = 22.9 Hz, C5), 16.2 (d, J_{CP} = 6.7 Hz, C1) ppm; **^{19}F-NMR** (470 MHz, $CDCl_3$): δ = -113.40 (tt, *J* = 8.7, 5.4 Hz) ppm; **^{31}P-NMR** (202 MHz, $CDCl_3$): δ = 15.86 (dp, *J* = 15.6, 7.7 Hz) ppm; **IR (ATR):** $\tilde{\nu}$ = 3457 (w), 2982 (w), 2908 (w), 1602 (m), 1508 (s), 1479 (w), 1441 (w), 1392 (w), 1375 (w), 1345 (w), 1227 (s), 1192 (w), 1162 (m), 1098 (w), 1051 (s), 1026 (s), 958 (s), 842 (s), 818 (m), 785 (m), 741 (w), 730 (w), 666 (m) cm^{-1}; **HR-ESI-MS**: *m/z*: 295.0878 ([*M*+Na]$^+$, calcd. for $C_{13}H_{18}FNaO_3P^+$: 295.0870), 567.1855 ([M_2+Na]$^+$, cald. for $C_{26}H_{36}F_2NaO_6P_2^+$: 567.1847).

Diethyl (*Z*)-(2-(4-chlorophenyl)prop-1-en-1-yl)phosphonate (*Z*-3):

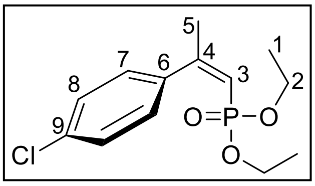

According to **general procedure B**, ***E*-3** (28.9 mg, 0.10 mmol) was converted to ***Z*-3**. Purification by column chromatography (SiO_2, *n*-pentane/ethyl acetate: 15/85) yielded a yellow oil (25.8 mg, 89%, ***Z*-3**:***E*-3** = 90:10).

R$_f$ = 0.21 (SiO_2, *n*-penatne/ethyl acetate: 1/1); **^{1}H NMR** (500 MHz, $CDCl_3$): δ = 7.35 - 7.29 (m, 4H, H7, H8), 5.73 (dq, *J* = 16.8, 1.4 Hz, 1H, H3), 3.91 - 3.76 (m, 4H, H2), 2.22 - 2.18 (m, 3H, H5), 1.11 (t, *J* = 7.1 Hz, 6H, H1) ppm; **^{13}C NMR** (126 MHz, $CDCl_3$): δ = 157.7 (d, J_{CP} = 4.3 Hz, C4), 139.0 (d, J_{CP} = 7.6 Hz, C6), 134.4 (C9), 128.9 (d, J_{CP} = 1.9 Hz, C7), 128.2 (C8), 115.7 (d, J_{CP} = 191.0 Hz, C3), 61.5 (d, J_{CP} = 6.0 Hz, C2), 28.3 (d, J_{CP} = 22.9 Hz, C5), 16.2 (d, J_{CP} = 6.7 Hz, C1) ppm; **^{31}P NMR** (202 MHz, $CDCl_3$): δ = 15.60 (dp, *J* = 15.7, 7.7 Hz) ppm; **IR (ATR):** $\tilde{\nu}$ = 3466 (w), 2981 (w), 2908 (w), 1619 (w), 1594 (w), 1490 (m), 1440 (w), 1393 (w), 1237 (s), 1163 (w), 1091 (m), 1051 (s), 1026 (s), 959 (s), 837 (s), 791 (m), 756 (m) cm^{-1}; **HR-ESI-MS**: *m/z*: 311.0577 ([*M*+Na]$^+$, calcd. for $C_{13}H_{18}ClNaO_3P^+$: 311.0574), 599.1247 ([M_2+Na]$^+$, cald. for $C_{26}H_{36}Cl_2NaO_6P_2^+$: 599.1256).

Diethyl (*Z*)-(2-(4-bromophenyl)prop-1-en-1-yl)phosphonate (*Z*-4):

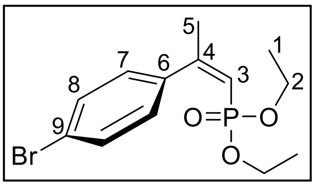

According to **general procedure B**, ***E*-4** (33.3 mg, 0.10 mmol) was converted to ***Z*-4**. Purification by column chromatography (SiO_2, *n*-pentane/ethyl acetate: 1/9) yielded a yellow oil (29.0 mg, 87%, ***Z*-4**:***E*-4** = 89:11).

R$_f$ = 0.49 (SiO_2, ethyl acetate); **^{1}H NMR** (600 MHz, $CDCl_3$): δ = 7.48 - 7.44 (m, 2H, H8), 7.28 - 7.24 (m, 2H, H7), 5.72 (dd, *J* = 16.8, 1.5 Hz, 1H, H3), 3.91 - 3.76 (m, 4H, H2), 2.19 - 2.18 (m, 3H, H5), 1.10 (t, *J* = 7.1 Hz, 6H, H1) ppm; **^{13}C NMR** (151 MHz, $CDCl_3$): δ = 157.7 (d, J_{CP} = 4.2 Hz, C4), 139.5 (d, J_{CP} = 7.6 Hz, C6), 131.2 (C8), 129.2 (d, J_{CP} = 1.7 Hz, C7), 122.5 (C9), 115.7 (d, J_{CP} = 191.1 Hz, C3), 61.5 (d, J_{CP} = 6.1 Hz, C2), 28.3 (d, J_{CP} = 22.8 Hz, C5), 16.2 (d, J_{CP} = 6.9 Hz, C1) ppm; **^{31}P NMR** (243 MHz, $CDCl_3$): δ = 15.55 (dp, *J* = 15.6, 7.7 Hz) ppm; **IR (ATR):** $\tilde{\nu}$ = 3456 (w), 2979 (w), 2908 (w), 1616 (w), 1588 (w), 1487 (m), 1440 (w), 1392 (w), 1238 (s), 1163 (w), 1097 (w), 1076 (m), 1050 (s), 1026 (s), 1008 (s), 958 (s), 833 (m), 793 (m), 746 (m) cm^{-1}; **HR-ESI-MS**: *m/z*: 355.0068 ([*M*+Na]$^+$, calcd. for $C_{13}H_{18}BrNaO_3P^+$: 355.0069), 689.0234 ([*M$_2$*+Na]$^+$, cald. for $C_{26}H_{36}Br_2NaO_6P_2^+$: 689.0226).

Diethyl (*Z*)-(2-(4-(trifluoromethyl)phenyl)prop-1-en-1-yl)phosphonate (*Z*-5):

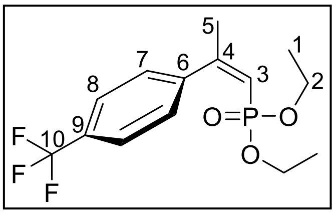

According to **general procedure B**, ***E*-5** (32.2 mg, 0.10 mmol) was converted to ***Z*-5**. Purification by column chromatography (SiO_2, cyclohexane/ethyl acetate: 1/19) yielded a yellow oil (28.9 mg, 90%, ***Z*-5**:***E*-5** = 94:6).

R$_f$ = 0.45 (SiO_2, ethyl acetate); **^{1}H NMR** (600 MHz, $CDCl_3$): δ = 7.60 (d, *J* = 8.5 Hz, 2H, H8), 7.48 (d, *J* = 8.0 Hz, 2H, H7), 5.79 (dq, *J* = 16.8, 1.4 Hz, 1H, H3), 3.91 - 3.74 (m, 4H, H2), 2.23 - 2.22 (m, 3H, H5), 1.08 (t, *J* = 7.1 Hz, 6H, H1); **^{13}C NMR** (151 MHz, $CDCl_3$): δ = 157.4 (d, J_{CP} = 4.1 Hz, C4), 144.4 (dq, J_{CP} = 7.6, J_{CF} = 1.4 Hz, C6), 130.4 (q, J_{CF} = 32.6 Hz, C9), 127.9 (d, J_{CP} = 1.8 Hz, C7), 125.0 (q, J_{CF} = 3.7 Hz, C8), 124.1 (q, J_{CF} = 271.8 Hz, C10), 116.6 (d, J_{CP} = 191.0 Hz, C3), 61.6 (d, J_{CP} = 6.1 Hz, C2), 28.4 (d, J_{CP} = 22.7 Hz, C5), 16.1 (d, J_{CP} = 6.7 Hz, C1) ppm; **^{19}F NMR** (564 MHz, $CDCl_3$): δ = -62.77 ppm; **^{31}P NMR** (243 MHz, $CDCl_3$): δ = 15.05 (dp, *J* = 15.6, 7.7 Hz) ppm; **IR (ATR):** $\tilde{\nu}$ = 3457 (w), 2920 (m), 2851 (w), 1613 (w), 1571 (w), 1443 (w), 1405 (w), 1325 (s), 1242 (m), 1166 (m), 1126 (s), 1078 (m), 1054 (s), 1029 (s), 963 (m), 848 (m), 798 (w), 738 (w), 715 (w) cm^{-1}; **HR-ESI-MS**: *m/z*: 345.0835 ([*M*+Na]$^+$, calcd. for $C_{14}H_{18}F_3NaO_3P^+$: 345.0838), 667.1774 ([*M$_2$*+Na]$^+$, cald. for $C_{28}H_{36}F_6NaO_6P_2^+$: 667.1784).

Diethyl (*Z*)-(2-(4-tolyl)prop-1-en-1-yl)phosphonate (*Z*-6):

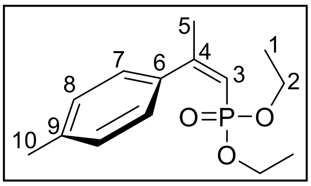

According to **general procedure B**, ***E*-6** (26.8 mg, 0.10 mmol) was converted to ***Z*-6**. Purification by column chromatography (SiO_2, ethyl acetate) yielded a yellow oil (19.6 mg, 73%, ***Z*-6**:***E*-6** = 80:20).

R$_f$ = 0.49 (SiO_2, ethyl acetate:); **^{1}H NMR** (500 MHz, $CDCl_3$): δ = 7.31 - 7.28 (m, 2H, H7), 7.17 - 7.13 (m, 2H, H8), 5.68 (dq, *J* = 17.3, 1.4 Hz, 1H, H3), 3.90 - 3.74 (m, 4H, H2), 2.33 (s, 3H, H10), 2.21 (t, *J* = 1.2 Hz, 3H, H5), 1.09 (t, *J* = 7.1 Hz, 6H, H1) ppm; **^{13}C NMR** (126 MHz, $CDCl_3$): δ = 159.2 (d, J_{CP} = 4.7 Hz, C4), 138.3 (C9), 137.6 (d, J_{CP} = 7.4 Hz, C6), 128.7 (C8), 127.4 (d, J_{CP} = 1.7 Hz, C7), 114.3 (d, J_{CP} = 191.3 Hz, C3), 61.4 (d, J_{CP} = 6.0 Hz, C2), 28.5 (d, J_{CP} = 23.2 Hz, C5), 21.3 (C10), 16.2 (d, J_{CP} = 6.8 Hz, C1) ppm; **^{31}P NMR** (202 MHz, $CDCl_3$): δ = 16.42 ppm; **IR (ATR):** $\tilde{\nu}$ = 3460 (w), 2981 (w), 2926 (w), 1611 (w), 1512 (w), 1441 (w), 1391 (w), 1237 (s), 1187 (w), 1163 (w), 1098 (w), 1052 (s), 1027 (s), 957 (s), 854 (m), 826 (m), 783 (m), 742 (w) cm^{-1}; **HR-ESI-MS**: *m/z*: 291.1124 ([*M*+Na]$^+$, calcd. for $C_{14}H_{21}NaO_3P^+$: 291.1121), 559.2351 ([M_2+Na]$^+$, cald. for $C_{28}H_{42}NaO_6P_2^+$: 559.2349).

Diethyl (*Z*)-(2-(4-(*tert*-butyl)phenyl)prop-1-en-1-yl)phosphonate (*Z*-7):

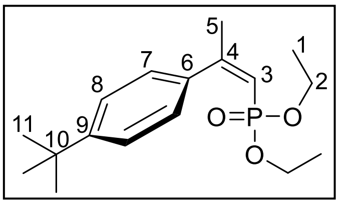

According to **general procedure B**, ***E*-7** (31.0 mg, 0.10 mmol) was converted to ***Z*-7**. Purification by column chromatography (SiO_2, cyclohexane/ethyl acetate: 1/4) yielded a yellow oil (28.6 mg, 92%, ***Z*-7**:***E*-7** = 87:13).

R$_f$ = 0.52 (SiO_2, ethyl acetate); **^{1}H NMR** (600 MHz, $CDCl_3$): δ = 7.38 - 7.32 (m, 4H, H7, H8), 5.68 (dq, *J* = 17.4, 1.4 Hz, 1H, H3), 3.86 - 3.71 (m, 4H, H2), 2.23 - 2.21 (m, 3H, H5), 1.29 (s, 9H, H11), 1.03 (td, *J* = 7.1, 0.6 Hz, 6H, H1) ppm; **^{13}C NMR** (151 MHz, $CDCl_3$): δ = 159.0 (d, J_{CP} = 4.5 Hz, C4), 151.6 (C9), 137.6 (d, J_{CP} = 7.4 Hz, C6), 127.3 (d, J_{CP} = 1.7 Hz, C7), 124.9 (C8), 114.4 (d, J_{CP} = 191.7 Hz, C3), 61.4 (d, J_{CP} = 6.0 Hz, C2), 34.7 (C10), 31.4 (C11), 28.3 (d, J_{CP} = 23.3 Hz, C5), 16.1 (d, J_{CP} = 7.0 Hz, C1) ppm; **^{31}P NMR** (243 MHz, $CDCl_3$): δ = 16.45 (dt, *J* = 17.3, 7.5 Hz) ppm; **IR (ATR):** $\tilde{\nu}$ = 3457 (w), 2963 (w), 2869 (w), 1617 (w), 1511 (w), 1464 (w), 1441 (w), 1392 (w), 1364 (w), 1342 (w), 1237 (m), 1202 (w), 1162 (w), 1113 (w), 1098 (w), 1052 (s), 1025 (s), 956 (s), 838 (m), 789 (m), 756 (w) cm^{-1}; **HR-ESI-MS**: *m/z*: 333.1593 ([*M*+Na]$^+$, calcd. for $C_{17}H_{27}NaO_3P^+$: 333.1590), 643.3293 ([M_2+Na]$^+$, cald. for $C_{34}H_{54}NaO_6P_2^+$: 643.3288).

Diethyl (*Z*)-(2-(3-bromophenyl)prop-1-en-1-yl)phosphonate (*Z*-8):

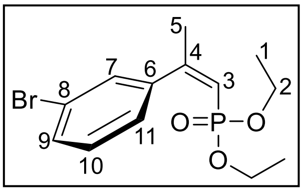

According to **general procedure B**, ***E*-8** (33.3 mg, 0.10 mmol) was converted to ***Z*-8**. Purification by column chromatography (SiO_2, ethyl acetate) yielded a yellow oil (20.4 mg, 61%, ***Z*-8**:***E*-8** = 91:9).

R$_f$ = 0.29 (SiO_2, ethyl acetate); **^{1}H NMR** (500 MHz, $CDCl_3$): δ = 7.51 (t, *J* = 1.8 Hz, 1H, H11), 7.45 (ddd, *J* = 8.0, 2.0, 1.0 Hz, 1H, H9), 7.33 (ddd, *J* = 7.7, 1.7, 1.1 Hz, 1H, H7), 7.23 (t, *J* = 7.8 Hz, 1H, H10), 5.76 (dq, *J* = 16.9, 1.5 Hz, 1H, H3), 3.95 – 3.77 (m, 4H, H2), 2.22 – 2.20 (m, 3H, H5), 1.13 (td, *J* = 7.1, 0.6 Hz, 6H, H1) ppm; **^{13}C NMR** (126 MHz, $CDCl_3$): δ = 157.3 (d, J_{CP} = 4.0 Hz, C4), 142.7 (d, J_{CP} = 7.6 Hz, C6), 131.3 (C9), 130.4 (d, J_{CP} = 1.8 Hz, C11), 129.7 (C10), 126.2 (d, J_{CP} = 1.9 Hz, C7), 122.1 (C8), 116.3 (d, J_{CP} = 191.3 Hz, C3), 61.6 (d, J_{CP} = 6.0 Hz, C2), 28.4 (d, J_{CP} = 22.8 Hz, C5), 16.3 (d, J_{CP} = 6.9 Hz, C1) ppm; **^{31}P NMR** (202 MHz, $CDCl_3$): δ = 15.34 (dp, *J* = 15.7, 7.8 Hz) ppm; **IR (ATR):** $\tilde{\nu}$ = 3467 (b), 2980 (w), 2906 (w), 1622 (w), 1592 (w), 1559 (w), 1473 (w), 1410 (w), 1392 (w), 1373 (w), 1336 (w), 1234 (m), 1192 (w), 1163 (w), 1097 (w), 1049 (s), 1021 (s), 956 (s), 853 (m), 795 (m), 739 (w), 696 (m), 661 (w) cm^{-1}; **HR-ESI-MS**: *m/z*: 355.0072 ([*M*+Na]$^+$, calcd. for $C_{13}H_{18}BrNaO_3P^+$: 355.0069), 689.0249 ([M_2+Na]$^+$, cald. for $C_{26}H_{36}Br_2NaO_6P_2^+$: 689.0226).

Dimethyl (*Z*)-(2-phenylprop-1-en-1-yl)phosphonate (*Z*-9):

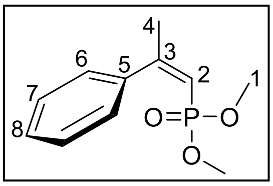

According to **general procedure B**, ***E*-9** (22.6 mg, 0.10 mmol) was converted to ***Z*-9**. Purification by column chromatography (SiO_2, *n* pentane/ethyl acetate: 1/9) yielded a yellow oil (22.0 mg, 97%, ***Z*-9**:***E*-9** = 92:8).

R$_f$ = 0.43 (SiO_2, ethyl acetate); **^{1}H NMR** (600 MHz, $CDCl_3$): δ = 7.38 - 7.33 (m, 4H, H6, H7), 7.32 - 7.29 (m, 1H, H8), 5.70 (dd, *J* = 17.6, 1.3 Hz, 1H, H2), 3.42 (dd, *J* = 11.1, 0.4 Hz, 6H, H1), 2.23 (ddd, *J* = 1.5, 1.0, 0.5 Hz, 3H, H4) ppm; **^{13}C NMR** (151 MHz, $CDCl_3$): δ = 159.9 (d, J_{CP} = 4.8 Hz, C3), 140.5 (d, J_{CP} = 7.6 Hz, C5), 128.5 (C8), 128.1 (C7), 127.2 (d, J_{CP} = 1.7 Hz, C6), 113.8 (d, J_{CP} = 192.2 Hz, C2), 52.0 (d, J_{CP} = 6.1 Hz, C1), 28.5 (d, J_{CP} = 23.1 Hz, C4) ppm; **^{31}P NMR** (243 MHz, $CDCl_3$): δ = 18.87 ppm; **IR (ATR):** $\tilde{\nu}$ = 3475 (w), 2951 (w), 2850 (w), 1616 (w), 1599 (w), 1494 (w), 1441 (w), 1374 (w), 1341 (w), 1237 (m), 1182 (w), 1023 (s), 922 (w), 864 (m), 803 (s), 764 (m), 747 (m), 699 (s) cm^{-1}; **HR-ESI-MS**: *m/z*: 249.0663 ([*M*+Na]$^+$, calcd. for $C_{11}H_{15}NaO_3P^+$: 249.0651), 475.1412 ([M_2+Na]$^+$, cald. for $C_{22}H_{30}NaO_6P_2^+$: 475.1410).

Diisopropyl (*Z*)-(2-phenylprop-1-en-1-yl)phosphonate (*Z*-10):

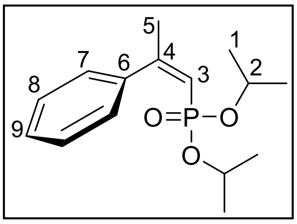

According to **general procedure B**, ***E*-10** (28.2 mg, 0.10 mmol) was converted to ***Z*-10**. Purification by column chromatography (SiO_2, cyclohexane/ethyl acetate: 1/9) yielded an orange oil (28.0 mg, 99%, ***Z*-10**:***E*-10** = 90:10).

R$_f$ = 0.55 (SiO_2, ethyl acetate); **^{1}H NMR** (600 MHz, $CDCl_3$): δ = 7.42 - 7.39 (m, 2H, H7), 7.34 - 7.30 (m, 2H, H8), 7.30 - 7.26 (m, 1H, H9), 5.71 (dq, *J* = 17.3, 1.3 Hz, 1H, H3), 4.47 (ddt, *J* = 12.4, 7.6, 6.2 Hz, 2H, H2), 2.23 - 2.19 (m, 3H, H5), 1.16 (d, *J* = 6.2 Hz, 6H, H1), 1.04 (d, *J* = 6.2 Hz, 6H, H1’) ppm; **^{13}C NMR** (151 MHz, $CDCl_3$): δ = 158.2 (d, J_{CP} = 4.2 Hz, C4), 140.8 (d, J_{CP} = 7.4 Hz, C6), 128.3 (C9), 127.9 (C8), 127.7 (d, J_{CP} = 1.7 Hz, C7), 116.5 (d, J_{CP} = 193.0 Hz, C3), 70.2 (d, J_{CP} = 6.4 Hz, C2), 28.6 (d, J_{CP} = 23.1 Hz, C5), 24.0 (d, J_{CP} = 4.0 Hz, C1), 23.7 (d, J_{CP} = 5.2 Hz, C1’) ppm; **^{31}PNMR** (243 MHz, $CDCl_3$): δ = 13.96 (dt, *J* = 16.4, 7.8 Hz) ppm; **IR (ATR):** $\tilde{\nu}$ = 3449 (w), 2977 (w), 2922 (w), 2851 (w), 1617 (w), 1599 (w), 1494 (w), 1442 (w), 1385 (w), 1373 (w), 1236 (m), 1177 (w), 1141 (w), 1107 (m), 1072 (w), 978 (s), 886 (m), 841 (w), 805 (w), 766 (m), 699 (m) cm^{-1}; **HR-ESI-MS**: *m/z*: 305.1286 ([*M*+Na]$^+$, calcd. for $C_{15}H_{23}NaO_3P^+$: 305.1277), 587.2676 ([*M$_2$*+Na]$^+$, cald. for $C_{30}H_{46}NaO_6P_2^+$: 587.2662); analytical data in agreement with literature.[4]

Diethyl (*Z*)-(2-(naphthalen-2-yl)prop-1-en-1-yl)phosphonate (*Z*-11):

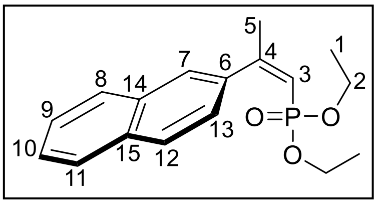

According to **general procedure B**, ***E*-11** (30.4 mg, 0.10 mmol) was converted to ***Z*-11**. Purification by column chromatography (SiO_2, cyclohexane/ethyl acetate: 1/9) yielded an orange oil (27.6 mg, 91%, ***Z*-11**:***E*-11** = 66:34).

R$_f$ = 0.63 (SiO_2, ethyl acetate); **^{1}H NMR** (300 MHz, $CDCl_3$): δ = 7.91 (d, *J* = 1.9 Hz, 1H, H7), 7.88 - 7.78 (m, 3H, H8, H11, H12), 7.53 - 7.43 (m, 3H, H9, H10, H13), 5.83 (dp, *J* = 17.3, 1.4 Hz, 1H, H3), 3.93 - 3.64 (m, 4H, H2), 2.34 – 2.30 (m, 3H, H5), 1.00 (t, *J* = 7.1 Hz, 6H, H1) ppm; **^{13}C NMR** (151 MHz, $CDCl_3$): δ = 159.1 (d, J_{CP} = 4.4 Hz, C4), 138.0 (d, J_{CP} = 7.6 Hz, C6), 133.2 (C15), 132.9 (C14), 128.4 (C8), 127.7 (C11/C12), 127.6 (C11/C12), 126.8 (d, J_{CP} = 2.0 Hz, C7), 126.5 (C9/C10), 126.4 (C9/C10), 125.3 (d, J_{CP} = 1.6 Hz, C13), 115.4 (d, J_{CP} = 191.9 Hz, C3), 61.5 (d, J_{CP} = 6.1 Hz, C2), 28.5 (d, J_{CP} = 23.1 Hz, C5), 16.1 (d, J_{CP} = 6.6 Hz, C1) ppm; **^{31}P NMR** (243 MHz, $CDCl_3$): δ = 16.21 ppm; **IR (ATR):** $\tilde{\nu}$ = 3456 (w), 3056 (w), 2981 (w), 2926 (w), 2853 (w), 1615 (w), 1598 (w), 1504 (w), 1439 (w), 1390 (w), 1323 (w), 1234 (s), 1163 (w), 1129 (w), 1097 (w), 1052 (s), 1026 (s), 958 (s), 874 (w), 842 (w), 822 (m), 784 (m), 750 (m) cm^{-1}; **HR-ESI-MS**: *m/z*: 327.1127 ([*M*+Na]$^+$, calcd. for $C_{17}H_{21}NaO_3P^+$: 327.1121), 631.2359 ([*M$_2$*+Na]$^+$, cald. for $C_{34}H_{42}NaO_6P_2^+$: 631.2349).

Diethyl (*Z*)-(2-(4-methoxyphenyl)prop-1-en-1-yl)phosphonate (*Z*-12):

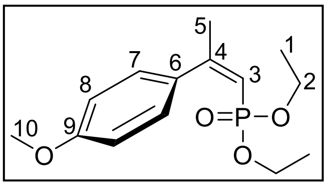

According to **general procedure B**, ***E*-12** (28.4 mg, 0.10 mmol) was converted to ***Z*-12**. Purification by column chromatography (SiO_2, ethyl acetate) yielded a yellow oil (23.7 mg, 83%, ***Z*-12**:***E*-12** = 58:42).

R$_f$ = 0.48 (SiO_2, ethyl acetate); **^{1}H NMR** (600 MHz, $CDCl_3$): δ = 7.41 - 7.37 (m, 2H, H7), 6.89 - 6.85 (m, 2H, H8), 5.65 (dq, *J* = 17.0, 1.4 Hz, 1H, H3), 3.90 - 3.79 (m, 7H, H2, H10), 2.22 (dd, *J* = 1.4, 0.9 Hz, 3H, H5), 1.11 (td, *J* = 7.0, 0.5 Hz, 6H, H1) ppm; **^{13}C NMR** (151 MHz, $CDCl_3$): δ = 160.0 (C9), 158.8 (d, J_{CP} = 4.7 Hz, C4), 132.8 (d, J_{CP} = 7.6 Hz, C6), 129.1 (d, J_{CP} = 1.7 Hz, C7), 113.7 (d, J_{CP} = 191.3 Hz, C3)), 113.4 (C8), 61.5 (d, J_{CP} = 6.0 Hz, C2), 55.4 (C10), 28.3 (d, J_{CP} = 23.2 Hz, C5), 16.2 (d, J_{CP} = 6.7 Hz, C1) ppm; **^{31}P NMR** (243 MHz, $CDCl_3$): δ = 16.72 (dt, *J* = 14.9, 6.7 Hz) ppm; **IR (ATR):** $\tilde{\nu}$ = 3449 (w), 2957 (w), 2918 (m), 2850 (w), 1608 (m), 1511 (m), 1462 (w), 1443 (w), 1376 (w), 1292 (w), 1252 (s), 1180 (m), 1095 (m), 1027 (s), 959 (m), 837 (m), 798 (s), 666 (w) cm^{-1}; **HR-ESI-MS**: *m/z*: 307.1073 ([*M*+Na]$^+$, calcd. for $C_{14}H_{21}NaO_4P^+$: 307.1070), 591.2241 ([M_2+Na]$^+$, cald. for $C_{28}H_{42}NaO_8P_2^+$: 591.2247).

Diethyl (*Z*)-styrylphosphonate (*Z*-13):

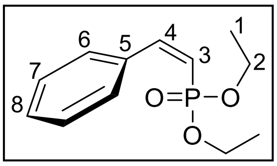

According to **general procedure B**, ***E*-13** (24.0 mg, 0.10 mmol) was converted to ***Z*-13**. Purification by column chromatography (SiO_2, *n*-pentane/ethyl acetate: 15/85) yielded a yellow oil (21.9 mg, 91%, ***Z*-13**:***E*-13** = 50:50).

R$_f$ = 0.59 (SiO_2, ethyl acetate); **^{1}H NMR** (600 MHz, $CDCl_3$): δ = 7.69 - 7.64 (m, 2H, H6), 7.40 - 7.31 (m, 3H, H7, H8), 7.30 - 7.19 (m, 1H, H4), 5.79 (dd, *J* = 15.5, 14.2 Hz, 1H, H3), 4.02 - 3.92 (m, 4H, H2), 1.16 (td, *J* = 7.1, 0.5 Hz, 6H, H1) ppm; **^{13}C NMR** (151 MHz, $CDCl_3$): δ = 148.5 (d, J_{CP} = 2.0 Hz, C4), 135.4 (d, J_{CP} = 8.9 Hz, C5), 129.7 (d, J_{CP} = 1.8 Hz, C6), 129.4 (C7/C8), 128.2 (C7/C8), 116.7 (d, J_{CP} = 185.4 Hz, C3), 61.8 (d, J_{CP} = 5.9 Hz, C2), 16.2 (d, J_{CP} = 6.6 Hz, C1) ppm; **^{31}P NMR** (243 MHz, $CDCl_3$): δ = 15.95 ppm; analytical data in agreement with literature.[5]

Diethyl (*Z*)-(2-phenylbut-1-en-1-yl)phosphonate (*Z*-14):

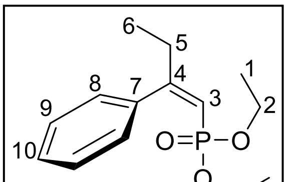

According to **general procedure B**, ***E*-14** (26.8 mg, 0.10 mmol) was converted to ***Z*-14**. Purification by column chromatography (SiO_2, *n*-pentane/ethyl acetate: 1/9) yielded a yellow oil (25.4 mg, 95%, ***Z*-14**:***E*-14** = 96:4).

R$_f$ = 0.48 (SiO_2, ethyl acetate); **^{1}H NMR** (500 MHz, $CDCl_3$): δ = 7.35 - 7.26 (m, 5H, H8, H9, H10), 5.68 (dt, *J* = 17.4, 1.5 Hz, 1H, H3), 3.88 - 3.67 (m, 4H, H2), 2.48 (qdd, *J* = 7.3, 1.5, 1.0 Hz, 2H, H5), 1.09 - 1.01 (m, 9H, H1, H6) ppm; **^{13}C NMR** (126 MHz, $CDCl_3$): δ = 164.7 (d, J_{CP} = 4.1 Hz,

C4), 140.3 (d, J_{CP} = 7.8 Hz, C7), 128.1 (C10), 127.9 (C9), 127.6 (d, J_{CP} = 1.8 Hz, C8), 113.1 (d, J_{CP} = 192.4 Hz, C3), 61.4 (d, J_{CP} = 6.1 Hz, C2), 34.4 (d, J_{CP} = 21.4 Hz, C5), 16.2 (d, J_{CP} = 6.7 Hz, C1), 12.2 (C6) ppm; **^{31}P NMR** (202 MHz, $CDCl_3$): δ = 17.08 (dp, *J* = 15.5, 7.2 Hz) ppm; **IR (ATR):** $\tilde{\nu}$ = 3457 (w), 2975 (w), 2924 (w), 2852 (w), 1619 (w), 1598 (w), 1457 (w), 1443 (w), 1391 (w), 1237 (s), 1162 (w), 1097 (w), 1052 (s), 1025 (s), 959 (s), 858 (w), 829 (w), 773 (m), 701 (m) cm^{-1}; **HR-ESI-MS**: *m/z*: 291.1126 ([*M*+Na]$^+$, calcd. for $C_{14}H_{21}NaO_3P^+$: 291.1121), 559.2345 ([M_2+Na]$^+$, cald. for $C_{28}H_{42}NaO_6P_2^+$: 559.2349); analytical data in agreement with literature.[4]

Diethyl (*Z*)-(2-(2-tolyl)prop-1-en-1-yl)phosphonate (*Z*-15):

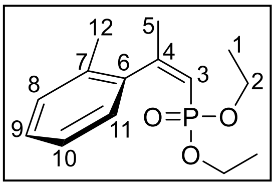

According to **general procedure B**, ***E*-15** (26.8 mg, 0.10 mmol) was converted to ***Z*-15**. Purification by column chromatography (SiO_2, ethyl acetate) yielded a yellow oil (19.1 mg, 71%, ***Z*-15**:***E*-15** = 93:7).

R_f = 0.64 (SiO_2, ethyl acetate); **^{1}H NMR** (600 MHz, $CDCl_3$): δ = 7.18 - 7.12 (m, 3H, H8, H9, H10), 7.06 (dt, *J* = 6.5, 1.5 Hz, 1H, H11), 5.81 (dq, *J* = 19.1, 1.5 Hz, 1H, H3), 3.79 (dp, *J* = 10.1, 7.2 Hz, 2H, H2), 3.73 - 3.53 (m, 2H, H2'), 2.26 (s, 3H, H12), 2.12 – 2.11 (m, 3H, H5), 1.08 (t, *J* = 7.1 Hz, 6H, H1) ppm; **^{13}C NMR** (151 MHz, $CDCl_3$): δ = 159.4 (d, J_{CP} = 4.7 Hz, C4), 140.8 (d, J_{CP} = 7.3 Hz, C6), 134.1 (d, J_{CP} = 1.5 Hz, C7), 129.9 (C8/C9/C10), 127.6 (C8/C9/C10), 127.2 (d, J_{CP} = 2.2 Hz, C11), 125.5 (C8/C9/C10), 116.4 (d, J_{CP} = 193.5 Hz, C3), 61.2 (d, J_{CP} = 6.1 Hz, C2), 28.5 (d, J_{CP} = 23.7 Hz, C5), 19.3 (C12), 16.2 (d, J_{CP} = 6.5 Hz, C1) ppm; **^{31}P NMR** (243 MHz, $CDCl_3$): δ = 15.51 ppm; **IR (ATR):** $\tilde{\nu}$ = 3449 (w), 2979 (w), 2909 (w), 1627 (w), 1600 (w), 1488 (w), 1440 (w), 1391 (w), 1369 (w), 1335 (w), 1239 (s), 1163 (w), 1098 (w), 1053 (s), 1027 (s), 959 (s), 855 (m), 803 (m), 785 (m), 762 (s), 727 (m), 698 (w) cm^{-1}; **HR-ESI-MS**: *m/z*: 291.1135 ([*M*+Na]$^+$, calcd. for $C_{14}H_{21}NaO_3P^+$: 291.1121), 559.2355 ([M_2+Na]$^+$, cald. for $C_{28}H_{42}NaO_6P_2^+$: 559.2349).

Diethyl (*Z*)-(2-(2-fluorophenyl)prop-1-en-1-yl)phosphonate (*Z*-16):

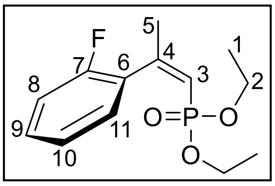

According to **general procedure B**, ***E*-16** (27.2 mg, 0.10 mmol) was converted to ***Z*-16**. Purification by column chromatography (SiO_2, cyclohexane/ethyl acetate: 1/9) yielded a yellow oil (24.8 mg, quant., ***Z*-16**:***E*-16** = 99:1).

R_f = 0.49 (SiO_2, ethyl acetate); **^{1}H NMR** (600 MHz, $CDCl_3$): δ = 7.33 - 7.25 (m, 2H, H9, H11), 7.12 (tt, *J* = 7.5, 0.9 Hz, 1H, H10), 7.04 (dd, *J* = 10.2, 8.2 Hz, 1H, H8), 5.84 (dq, *J* = 17.3, 1.2 Hz, 1H, H3), 3.91 - 3.76 (m, 4H, H2), 2.20 (s, 3H, H5), 1.12 (t, *J* = 7.1 Hz, 6H, H1) ppm; **^{13}C NMR** (151 MHz, $CDCl_3$): δ = 158.9 (dd, J_{CF} = 246.3, J_{CP} = 1.7 Hz, C7), 154.2 (d, J_{CP} = 3.4 Hz, C4), 130.1 (dd, J_{CF} = 3.8, J_{CP} = 2.0 Hz, C11), 129.8 (d, J_{CF} = 8.1 Hz, C9), 128.5 (dd, J_{CF} = 16.1, J_{CP} = 7.6 Hz, C6), 123.8 (d, J_{CF} = 3.5 Hz, C10), 117.8 (d, J_{CP} = 190.7 Hz, C3), 115.4 (d, J_{CF} = 21.7 Hz, C8), 61.4 (d, J_{CP} = 6.1 Hz, C2), 27.8

(d, J_{CP} = 22.5 Hz, C5), 16.2 (d, J_{CP} = 6.6 Hz, C1) ppm; **^{19}F NMR** (564 MHz, $CDCl_3$): δ = -115.54 (ddd, J = 10.1, 7.2, 5.5 Hz) ppm; **^{31}P NMR** (243 MHz, $CDCl_3$): δ = 14.78 ppm; **IR (ATR):** $\tilde{\nu}$ = 3475 (w), 2983 (w), 2908 (w), 1732 (w), 1630 (w), 1609 (w), 1576 (w), 1489 (m), 1444 (m), 1392 (w), 1372 (w), 1339 (w), 1239 (s), 1223 (s), 1182 (w), 1163 (w), 1105 (w), 1052 (s), 1025 (s), 961 (s), 853 (m), 830 (m), 813 (m), 787 (m), 760 (s), 697 (w) cm^{-1}; **HR-ESI-MS**: *m/z*: 295.0879 ([*M*+Na]$^+$, calcd. for $C_{13}H_{18}FNaO_3P^+$: 295.0870), 567.1851 ([M_2+Na]$^+$, cald. for $C_{26}H_{36}F_2NaO_6P_2^+$: 567.1847).

Diethyl (*Z*)-(2-(2-chlorophenyl)prop-1-en-1-yl)phosphonate (*Z*-17):

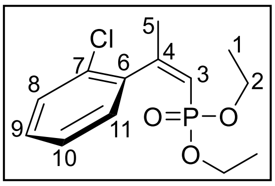

According to **general procedure B**, ***E*-17** (28.9 mg, 0.10 mmol) was converted to ***Z*-17**. Purification by column chromatography (SiO_2, cyclohexane/ethyl acetate: 1/9) yielded a yellow oil (26.3 mg, 91%, ***Z*-17**:***E*-17** = 96:4).

R_f = 0.33 (SiO_2, ethyl acetate); **^{1}H NMR** (600 MHz, $CDCl_3$): δ = 7.35 (dd, J = 7.4, 2.1 Hz, 1H, H8), 7.25 - 7.19 (m, 3H, H9, H10, H11), 5.83 (dd, J = 17.8, 1.4 Hz, 1H, H3), 3.85 (s, 4H, H2), 2.18 (s, 3H, H5), 1.11 (s, 6H, H1) ppm; **^{13}C NMR** (151 MHz, $CDCl_3$): δ = 157.1 (d, J_{CP} = 3.5 Hz, C4), 139.8 (d, J_{CP} = 7.6 Hz, C6), 131.2 (d, J_{CP} = 2.1 Hz, C7), 129.5 (d, J_{CP} = 2.0 Hz, C11), 129.3 (C8), 129.1 (C9/C10), 126.6 (C9/C10), 117.5 (d, J_{CP} = 191.2 Hz, C3), 61.4 (d, J_{CP} = 6.1 Hz, C2), 27.4 (d, J_{CP} = 22.4 Hz, C5), 16.2 (d, J_{CP} = 6.7 Hz, C1) ppm; **^{31}P NMR** (243 MHz, $CDCl_3$): δ = 14.69 ppm; **IR (ATR):** $\tilde{\nu}$ = 3449 (w), 2981 (w), 2917 (w), 2851 (w), 1629 (w), 1592 (s), 1472 (w), 1429 (w), 1392 (w), 1370 (w), 1337 (w), 1240 (m), 1163 (w), 1053 (s), 1026 (s), 959 (s), 853 (w), 790 (m), 753 (m), 669 (w) cm^{-1}; **HR-ESI-MS**: *m/z*: 311.0575 ([*M*+Na]$^+$, calcd. for $C_{13}H_{18}ClNaO_3P^+$: 311.0574), 599.1257 ([M_2+Na]$^+$, cald. for $C_{26}H_{36}Cl_2NaO_6P_2^+$: 599.1256).

Diethyl (*Z*)-(2-(2-bromophenyl)prop-1-en-1-yl)phosphonate (*Z*-18):

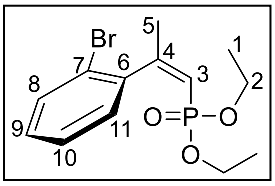

According to **general procedure B**, ***E*-18** (33.3 mg, 0.10 mmol) was converted to ***Z*-18**. Purification by column chromatography (SiO_2, ethyl acetate) yielded a yellow oil (28.4 mg, 85%, ***Z*-18**:***E*-18** = 84:16).

R_f = 0.34 (SiO_2, ethyl acetate); **^{1}H NMR** (500 MHz, $CDCl_3$): δ = 7.55 - 7.51 (m, 1H, H8), 7.28 (td, J = 7.5, 1.2 Hz, 1H, H10), 7.20 (dd, J = 7.6, 1.7 Hz, 1H, H11), 7.13 (ddd, J = 8.0, 7.4, 1.8 Hz, 1H, H9), 5.81 (dq, J = 17.7, 1.4 Hz, 1H, H3), 3.78 (d, J = 108.0 Hz, 4H, H2), 2.18 – 2.17 (m, 3H, H5), 1.11 (d, J = 39.2 Hz, 6H, H1) ppm; **^{13}C NMR** (126 MHz, $CDCl_3$): δ = 158.2 (d, J_{CP} = 3.4 Hz, C4), 141.8 (d, J_{CP} = 7.6 Hz, C6), 132.5 (C8), 129.4 (d, J_{CP} = 2.1 Hz, C11), 129.2 (C9), 127.1 (C10), 120.7 (d, J_{CP} = 2.2 Hz, C7), 117.3 (d, J_{CP} = 191.7 Hz, C3), 61.4 (d, J_{CP} = 5.0 Hz, C2), 27.5 (d, J_{CP} = 22.6 Hz, C5), 16.2 (d, J_{CP} = 6.7 Hz, C1) ppm; **^{31}P NMR** (243 MHz, $CDCl_3$): δ = 14.53 ppm; **IR (ATR):** $\tilde{\nu}$ = 3408 (w), 2978 (w), 2917 (w), 2850 (w), 1627 (w), 1589 (w), 1468 (w), 1427 (w), 1391 (w), 1369 (w), 1337 (w), 1240 (m), 1163 (w), 1098

(w), 1053 (s), 1024 (s), 961 (s), 853 (w), 821 (w), 790 (m), 759 (m), 655 (w) cm^{-1}; **HR-ESI-MS**: *m/z*: 355.0068 ([*M*+Na]$^+$, calcd. for $C_{13}H_{18}BrNaO_3P^+$: 355.0069), 689.0234 ([M_2+Na]$^+$, cald. for $C_{26}H_{36}Br_2NaO_6P_2^+$: 689.0226).

General Procedure C for the Hydrogenation of Vinylphosphonates

In a glovebox, $Rh(COD)_2BF_4$ (1.3 mg, 0.0032 mmol, 3.2 mol%) and (S_C,S_P)-WalPhos (2.3 mg, 0.0035 mmol, 3.5 mol%) were added to a vial and dissolved in DCM (1 mL). After stirring for 15 min, the specified vinylphosphonate (0.10 mmol, 1.00 eq.) was added and the vial was transferred to an autoclave. The autoclave was charged with H_2 (10 bar) and the solution was stirred at room temperature for 24 h. After carefully releasing the pressure and evaporation of the solvent, *n*-pentane (3 mL) was added and filtration through a plug of silica or a glass microfiber filter with subsequent elution with *n*-pentane (2 x 2 mL) yielded the products as clear oils. The enantiomeric ratios were determined by HPLC analysis using a chiral stationary phase.

Diethyl (*R*/*S*)-(2-phenylpropyl)phosphonate ((*R*/*S*)-19):

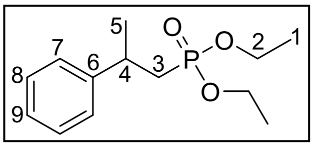

According to **general procedure C**, hydrogention of ***E*-1** (25.4 mg, 0.100 mmol) afforded **(+)-19** as colourless oil (24.8 mg, 0.097 mmol, 97%, e.r. = 97:03); hydrogention of ***Z*-1** (25.4 mg, 0.100 mmol) afforded **(-)-19** as colourless oil (23.0 mg, 0.090 mmol, 90%, e.r. = 01:99). The enantiomeric ratios were determined by HPLC analysis using a Daicel Chiralcel OJ-H (0.46 cm * 25 cm) column and *n*-hexane/*i*-propanol (98/2, 1.0 mL/min) as the eluent with detection at 210 nm.

Hydrogention of ***E*-1**: t_R = 13.05 min (minor enantiomer), 14.03 min (major enantiomer); **ODR** (CHCl3, c 1.0): $[\alpha]_D^{25}$ = +17.3°; hydrogention of ***Z*-1**: t_R = 12.90 min (major enantiomer), 14.14 min (minor enantiomer); **ODR** (CHCl3, c 1.0): $[\alpha]_D^{25}$ = -22.4°.

^{1}H NMR (400 MHz, $CDCl_3$): δ = 7.33 - 7.26 (m, 2H, H8), 7.24 - 7.15 (m, 3H, H7, H9), 4.05 - 3.83 (m, 4H, H2), 3.20 (dq, *J* = 11.1, 7.0 Hz, 1H, H4), 2.18 - 1.95 (m, 2H, H3), 1.38 (d, *J* = 6.9 Hz, 3H, H5), 1.22 (dt, *J* = 16.4, 7.0 Hz, 6H, H1) ppm; **^{13}C NMR** (101 MHz, $CDCl_3$): δ = 146.8 (d, J_{CP} = 11.9 Hz, C6), 128.6 (C8), 126.8 (C7), 126.5 (C9), 61.5 (dd, J_{CP} = 17.2, 6.4 Hz, C2), 34.8 (d, J_{CP} = 3.5 Hz, C4), 34.4 (d, J_{CP} = 138.4 Hz, C3), 23.6 (d, J_{CP} = 9.5 Hz, C5), 16.4 (dd, J_{CP} = 6.1, 1.6 Hz, C1) ppm; **^{31}P NMR** (162 MHz, $CDCl_3$): δ = 30.25 ppm; **IR (ATR):** $\tilde{\nu}$ = 3480 (b), 3029 (w), 2981 (w), 2932 (w), 2907 (w), 1649 (b), 1604 (w), 1495 (w), 1479 (w), 1454 (w), 1392 (w), 1368 (w), 1288 (w), 1240 (m), 1163 (w), 1097 (w), 1052 (s), 1022 (s), 955 (s), 855 (w), 830 (w), 781 (m), 762 (m), 699 (s) cm^{-1}; **HR-ESI-MS**: *m/z*: 279.1134 ([*M*+Na]$^+$, calcd. for

$C_{13}H_{21}NaO_3P^+$: 279.1121), 535.2356 ([M_2+Na]$^+$, cald. for $C_{26}H_{42}NaO_6P_2^+$: 535.2349); analytical data in agreement with literature.[1]

As a representative example the reaction was repeated on a 1 mmol scale:

In a glovebox, $Rh(COD)_2BF_4$ (13.0 mg, 0.032 mmol, 3.2 mol%) and (S_C,S_P)-WalPhos (23.0 mg, 0.035 mmol, 3.5 mol%) were added to a flask and dissolved in DCM (10 mL). After stirring for 15 min, vinylphosphonate ***E*-1** or ***Z*-1** (254.3 mg, 1.00 mmol, 1.00 eq.) was added and the flask was transferred to an autoclave. The autoclave was charged with H_2 (10 bar) and the solution was stirred at room temperature for 24 h. After carefully releasing the pressure and evaporation of the solvent, *n*-pentane (5 mL) was added and filtration through a glass microfiber filter with subsequent elution with *n*-pentane (2 x 5 mL) yielded the products **(+)-19** (252.7 mg, 0.99 mmol, 99%, e.r. = 97:03) and **(-)-19** (246.5 mg, 0.96 mmol, 96%, e.r. = 01:99) as colourless oils. The enantiomeric ratios were determined by HPLC analysis using a Daicel Chiralcel OJ-H (0.46 cm * 25 cm) column and *n*-hexane/*i*-propanol (98/2, 1.0 mL/min) as the eluent with detection at 210 nm.
Hydrogention of ***E*-1**: t_R = 11.75 min (minor enantiomer), 13.69 min (major enantiomer); hydrogention of ***Z*-1**: t_R = 12.58 min (major enantiomer), 15.40 min (minor enantiomer).

As a representative example the reaction was repeated using the opposite catalyst enantiomer

According to **general procedure C**, using (R_C,R_P)-WalPhos instead of (S_C,S_P)-WalPhos, hydrogention of ***E*-1** (25.4 mg, 0.100 mmol) afforded **(-)-19** as colourless oil (24.6 mg, 0.096 mmol, 96%, e.r. = 03:97); hydrogention of ***Z*-1** (25.4 mg, 0.100 mmol) afforded **(+)-19** as colourless oil (25.4 mg, 0.099 mmol, 99%, e.r. = 99:01). The enantiomeric ratios were determined by HPLC analysis using a Daicel Chiralcel OJ-H (0.46 cm * 25 cm) column and *n*-hexane/*i*-propanol (98/2, 1.0 mL/min) as the eluent with detection at 210 nm.
Hydrogention of ***E*-1**: t_R = 11.05 min (major enantiomer), 13.58 min (minor enantiomer); **ODR** (CHCl3, c 1.0): $[\alpha]_D^{27}$ = -19.4°; hydrogention of ***Z*-1**: t_R = 11.50 min (minor enantiomer), 13.48 min (major enantiomer); **ODR** (CHCl3, c 1.0): $[\alpha]_D^{29}$ = +19.7°.

Diethyl (*R*/*S*)-(2-(4-fluorophenyl)propyl)phosphonate ((*R*/*S*)-20):

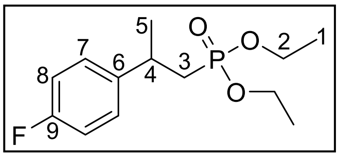

According to **general procedure C**, hydrogention of ***E*-2** (27.2 mg, 0.100 mmol) afforded **(+)-20** as colourless oil (26.8 mg, 0.098 mmol, 98%, e.r. = 97:03); hydrogention of ***Z*-2** (27.2 mg, 0.100 mmol) afforded **(-)-20** as colourless oil (26.0 mg, 0.095 mmol, 95%, e.r. = 02:98). The enantiomeric ratios were

determined by HPLC analysis using a Daicel Chiralcel OJ-H (0.46 cm * 25 cm) column and *n*-hexane/*i*-propanol (98/2, 1.0 mL/min) as the eluent with detection at 210 nm.

Hydrogention of ***E*-2**: t_R = 10.75 min (minor enantiomer), 11.57 min (major enantiomer); **ODR** (CHCl3, c 1.0): $[\alpha]_D^{27}$ = +10.2°; hydrogention of ***Z*-2**: t_R = 10.00 min (major enantiomer), 10.92 min (minor enantiomer); **ODR** (CHCl3, c 1.0): $[\alpha]_D^{27}$ = -19.0°.

^{1}H NMR (500 MHz, $CDCl_3$): δ = 7.18 (dd, *J* = 8.6, 5.4 Hz, 2H, H7), 6.97 (t, *J* = 8.6 Hz, 2H, H8), 4.06 - 3.85 (m, 4H, H2), 3.20 (s, 1H, H4), 2.15 - 1.95 (m, 2H, H2), 1.36 (d, *J* = 6.6 Hz, 3H, H5), 1.22 (dt, *J* = 21.8, 6.7 Hz, 6H, H1) ppm; **^{13}C NMR** (126 MHz, $CDCl_3$): δ = 161.5 (d, J_{CF} = 244.2 Hz, C9), 142.4 (C6), 128.2 (d, J_{CF} = 7.8 Hz, C7), 115.3 (d, J_{CF} = 21.2 Hz, C8), 61.5 (d, J_{CP} = 16.1 Hz, C2), 34.9 (d, J_{CP} = 141.0 Hz, C3), 34.2 (C4), 23.9 (d, J_{CP} = 7.2 Hz, C5), 16.5 (C1) ppm; **^{19}F NMR** (282 MHz, $CDCl_3$): δ = -116.87 ppm; **^{31}P NMR** (121 MHz, $CDCl_3$): δ = 29.90 ppm; **IR (ATR):** $\tilde{\nu}$ = 3650 (b), 3476 (b), 1604 (w), 1510 (s), 1480 (w), 1456 (w), 1393 (w), 1287 (w), 1222 (s), 1160 (m), 1098 (w), 1052 (s), 1023 (s), 955 (s), 832 (s), 777 (m), 737 (w), 713 (w), 685 (w) cm^{-1}; **HR-ESI-MS**: *m/z*: 297.1045 ($[M+Na]^+$, calcd. for $C_{13}H_{20}FNaO_3P^+$: 297.1026), 571.2173 ($[M_2+Na]^+$, cald. for $C_{26}H_{40}F_2NaO_6P_2^+$: 571.2160); analytical data in agreement with literature.[1]

Diethyl (*R*/*S*)-(2-(4-chlorophenyl)propyl)phosphonate ((*R*/*S*)-21):

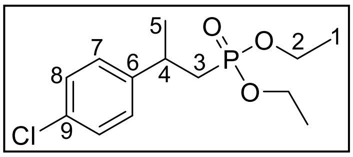

According to **general procedure C**, hydrogention of ***E*-3** (29.0 mg, 0.100 mmol) afforded **(+)-21** as colourless oil (29.1 mg, 0.100 mmol, quant., e.r. = 97:03); hydrogention of ***Z*-3** (28.9 mg, 0.100 mmol) afforded **(-)-21** as colourless oil (27.0 mg, 0.093 mmol, 93%, e.r. = 01:99). The enantiomeric ratios were determined by HPLC analysis using a Daicel Chiralcel OJ-H (0.46 cm * 25 cm) column and *n*-hexane/*i*-propanol (99.5/0.5, 0.5 mL/min) as the eluent with detection at 230 nm.

Hydrogention of ***E*-3**: t_R = 29.16 min (minor enantiomer), 30.60 min (major enantiomer); **ODR** (CHCl3, c 1.0): $[\alpha]_D^{26}$ = +23.1°; hydrogention of ***Z*-3**: t_R = 24.62 min (major enantiomer), 26.47 min (minor enantiomer); **ODR** (CHCl3, c 1.0): $[\alpha]_D^{27}$ = -26.1°.

^{1}H NMR (500 MHz, $CDCl_3$): δ = 7.29 - 7.25 (m, 2H, H8), 7.18 - 7.14 (m, 2H, H7), 4.06 - 3.88 (m, 4H, H2), 3.20 (dt, *J* = 17.8, 6.7 Hz, 1H, H4), 2.13 - 1.95 (m, 2H, H3), 1.37 (d, *J* = 6.9 Hz, 3H, H5), 1.24 (dt, *J* = 20.7, 7.0 Hz, 6H, H1) ppm; **^{13}C NMR** (126 MHz, $CDCl_3$): δ = 145.2 (d, J_{CP} = 11.0 Hz, C6), 132.1 (C9), 128.7 (C8), 128.2 (C7), 61.5 (dd, J_{CP} = 14.4, 5.8 Hz, C2), 34.5 (d, J_{CP} = 138.9 Hz, C3), 34.3 (d, J_{CP} = 2.6 Hz, C4), 23.7 (d, J_{CP} = 9.8 Hz, C5), 16.5 (C1) ppm; **^{31}P NMR** (202 MHz, $CDCl_3$): δ = 29.74 ppm; **IR (ATR):** $\tilde{\nu}$ = 3445 (b), 2981 (w), 2931 (w), 2908 (w), 1647 (b), 1493 (w), 1456 (w), 1411 (w), 1392 (w), 1368 (w), 1297 (w), 1227 (m), 1163 (w), 1095 (m), 1052 (s), 1023 (s), 1013 (s), 956 (s), 825 (m), 759 (w), 718 (w), 657 (m) cm^{-1};

HR-ESI-MS: *m/z*: 313.0738 ([*M*+Na]$^+$, calcd. for $C_{13}H_{20}ClNaO_3P^+$: 313.0731), 603.1569 ([M_2+Na]$^+$, cald. for $C_{26}H_{40}Cl_2NaO_6P_2^+$: 603.1577); analytical data in agreement with literature.[1]

Diethyl (*R*/*S*)-(2-(4-bromophenyl)propyl)phosphonate ((*R*/*S*)-22):

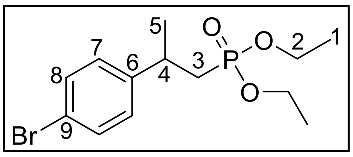

According to **general procedure C**, hydrogention of ***E*-4** (33.3 mg, 0.100 mmol) afforded **(+)-22** as colourless oil (31.2 mg, 0.093 mmol, 93%, e.r. = 97:03); hydrogention of ***Z*-4** (33.3 mg, 0.100 mmol) afforded **(-)-22** as colourless oil (31.6 mg, 0.094 mmol, 94%, e.r. = 01:99). The enantiomeric ratios were determined by HPLC analysis using a Daicel Chiralcel OJ-H (0.46 cm * 25 cm) column and *n*-hexane/*i*-propanol (99.5/0.5, 1 mL/min) as the eluent with detection at 230 nm.

Hydrogention of ***E*-4**: t_R = 20.01 min (minor enantiomer), 21.05 min (major enantiomer); **ODR** (CHCl3, c 1.0): $[\alpha]_D^{27}$ = +22.7°; hydrogention of ***Z*-4**: t_R = 19.60 min (major enantiomer), 21.48 min (minor enantiomer); **ODR** (CHCl3, c 1.0): $[\alpha]_D^{25}$ = -25.3°.

^{1}H NMR (500 MHz, $CDCl_3$): δ = 7.43 - 7.39 (m, 2H, H8), 7.12 - 7.08 (m, 2H, H7), 4.04 - 3.86 (m, 4H, H2), 3.17 (dq, *J* = 11.2, 7.0 Hz, 1H, H4), 2.02 (dqd, *J* = 18.0, 15.3, 7.1 Hz, 2H, H3), 1.35 (d, *J* = 6.9 Hz, 3H, H5), 1.22 (dt, *J* = 20.4, 7.0 Hz, 6H, H1) ppm; **^{13}C NMR** (126 MHz, $CDCl_3$): δ = 145.7 (d, J_{CP} = 11.3 Hz, C6), 131.6 (C8), 128.6 (C7), 120.2 (C9), 61.5 (dd, J_{CP} = 14.1, 6.4 Hz, C2), 34.4 (d, J_{CP} = 3.3 Hz, C4), 34.4 (d, J_{CP} = 138.8 Hz, C3), 23.6 (d, J_{CP} = 9.9 Hz, C5), 16.5 (dd, J_{CP} = 6.1, 3.8 Hz, C1) ppm; **^{31}P NMR** (202 MHz, $CDCl_3$): δ = 29.61 ppm; **IR (ATR):** $\tilde{\nu}$ = 3454 (b), 2980 (w), 2932 (w), 2907 (w), 1647 (b), 1592 (w), 1489 (w), 1456 (w), 1408 (w), 1392 (w), 1368 (w), 1297 (w), 1227 (m), 1163 (w), 1096 (w), 1051 (s), 1023 (s), 1008 (s), 956 (s), 822 (m), 751 (w), 715 (w) cm^{-1}; **HR-ESI-MS**: *m/z*: 357.0229 ([*M*+Na]$^+$, calcd. for $C_{13}H_{20}BrNaO_3P^+$: 357.0226), 693.0534 ([M_2+Na]$^+$, cald. for $C_{26}H_{40}Br_2NaO_6P_2^+$: 693.0539); analytical data in agreement with literature.[1]

Diethyl (*R*/*S*)-(2-(4-(trifluoromethyl)phenyl)propyl)phosphonate ((*R*/*S*)-23):

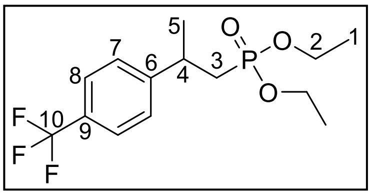

According to **general procedure C**, hydrogention of ***E*-5** (32.2 mg, 0.10 mmol) afforded **(+)-23** as colourless oil (32.0 mg, 0.099 mmol, 99%, e.r. = 98:02); hydrogention of ***Z*-5** (32.2 mg, 0.10 mmol) afforded **(-)-23** as colourless oil (32.4 mg, 0.10 mmol, quant., e.r. > 01:99). The enantiomeric ratios were determined by HPLC analysis using a Daicel Chiralcel OJ-H (0.46 cm * 25 cm) column and *n*-hexane/*i*-propanol (99.2/0.8, 0.4 mL/min) as the eluent with detection at 210 nm.

Hydrogention of ***E*-5**: t_R = 46.65 min (minor enantiomer), 49.01 min (major enantiomer); **ODR** (CHCl3, c 1.0): $[\alpha]_D^{27}$ = +17.1°; hydrogention of ***Z*-5**: t_R = 43.65 min (major enantiomer), 47.60 min (minor enantiomer); **ODR** (CHCl3, c 1.0): $[\alpha]_D^{27}$ = -21.3°.

^{1}H NMR (600 MHz, $CDCl_3$): δ = 7.55 (d, *J* = 8.0 Hz, 2H, H8), 7.34 (d, *J* = 8.1 Hz, 2H, H7), 4.03 - 3.86 (m, 4H, H2), 3.27 (dq, *J* = 11.2, 7.0 Hz, 1H, H4), 2.06 (dqd, *J* = 18.2, 15.3, 7.2 Hz, 2H, H3), 1.39 (dd, *J* = 6.9, 0.8 Hz, 3H, H5), 1.20 (dt, *J* = 30.5, 7.1 Hz, 6H, H1) ppm; **^{13}C NMR** (151 MHz, $CDCl_3$): δ = 150.7 (dd, J_{CP} = 11.0, 1.3 Hz, C6), 128.9 (q, J_{CF} = 32.3 Hz, C9), 127.3 (C7), 125.6 (q, J_{CF} = 3.8 Hz, C8), 124.3 (q, J_{CF} = 271.9 Hz, C10), 61.6 (dd, J_{CP} = 9.6, 6.6 Hz, C2), 34.9 (d, J_{CP} = 3.7 Hz, C4), 34.2 (d, J_{CP} = 139.6 Hz, C3), 23.6 (d, J_{CP} = 10.5 Hz, C5), 16.4 (dd, J_{CP} = 8.2, 6.2 Hz, C1) ppm; **^{19}F NMR** (564 MHz, $CDCl_3$): δ = -62.48 ppm; **^{31}P NMR** (243 MHz, $CDCl_3$): δ = 29.26 ppm; **IR (ATR):** $\tilde{\nu}$ = 3455 (b), 2983 (w), 2935 (w), 2908 (w), 1619 (w), 1456 (w), 1422 (w), 1393 (w), 1369 (w), 1325 (s), 1295 (w), 1162 (m), 1119 (s), 1067 (s), 1052 (s), 1016 (s), 957 (s), 835 (m), 794 (m), 776 (w), 718 (w) cm^{-1}; **HR-ESI-MS**: *m/z*: 347.1011 ($[M+Na]^+$, calcd. for $C_{14}H_{20}F_3NaO_3P^+$: 347.0994), 671.2117 ($[M_2+Na]^+$, cald. for $C_{28}H_{40}F_6NaO_6P_2^+$: 671.2097); analytical data in agreement with literature.[1]

Diethyl (*R*/*S*)-(2-(4-tolyl)propyl)phosphonate ((*R*/*S*)-24):

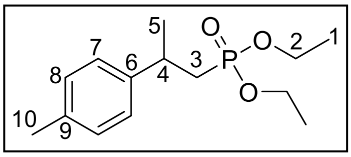

According to **general procedure C**, hydrogention of ***E*-6** (26.8 mg, 0.10 mmol) afforded **(+)-24** as colourless oil (25.7 mg, 0.095 mmol, 95%, e.r. = 97:03); hydrogention of ***Z*-6** (26.8 mg, 0.10 mmol) afforded **(-)-24** as colourless oil (24.5 mg, 0.091 mmol, 91%, e.r. = 02:98). The enantiomeric ratios were determined by HPLC analysis using a Daicel Chiralpak AS-H (0.46 cm * 25 cm) column and *n*-hexane/*i*-propanol (97/3, 1.0 mL/min) as the eluent with detection at 220 nm.

Hydrogention of ***E*-6**: t_R = 18.98 min (major enantiomer), 21.94 min (minor enantiomer); **ODR** (CHCl3, c 1.0): $[\alpha]_D^{27}$ = +19.3°; hydrogention of ***Z*-6**: t_R = 19.19 min (minor enantiomer), 21.31 min (major enantiomer); **ODR** (CHCl3, c 1.0): $[\alpha]_D^{24}$ = -24.0°.

^{1}H NMR (500 MHz, $CDCl_3$): δ = 7.13 - 7.08 (m, 4H, H7, H8), 4.04 - 3.87 (m, 4H, H2), 3.22 - 3.12 (m, 1H, H4), 2.31 (s, 3H, H10), 2.13 - 1.96 (m, 2H H3), 1.37 (d, *J* = 7.0 Hz, 3H, H5), 1.23 (dt, *J* = 18.6, 7.1 Hz, 6H, H1) ppm; **^{13}C NMR** (126 MHz, $CDCl_3$): δ = 143.9 (d, J_{CP} = 12.5 Hz, C6), 136.0 (C9), 129.3 (C8), 126.6 (C7), 61.4 (dd, J_{CP} = 23.1, 6.5 Hz, C2), 34.5 (d, J_{CP} = 138.0 Hz, C3), 34.4 (d, J_{CP} = 3.6 Hz, C4), 23.6 (d, J_{CP} = 9.0 Hz, C5), 21.1 (C10), 16.5 (dd, J_{CP} = 6.2, 2.6 Hz, C1) ppm; **^{31}P NMR** (202 MHz, $CDCl_3$): δ = 30.31 (dddd, *J* = 26.1, 18.4, 11.0, 7.7 Hz) ppm; **IR (ATR):** $\tilde{\nu}$ = 3477 (b), 2980 (w), 2929 (w), 1636 (b), 1516 (w), 1456 (w), 1392 (w), 1285 (w), 1240 (m), 1163 (w), 1097 (w), 1052 (s), 1021 (s), 955 (s), 816 (m), 778 (m), 735 (w), 719 (w) cm^{-1}; **HR-ESI-MS**: *m/z*: 293.1293 ($[M+Na]^+$, calcd. for $C_{14}H_{23}NaO_3P^+$: 293.1277), 563.2683 ($[M_2+Na]^+$, cald. for $C_{28}H_{46}NaO_6P_2^+$: 563.2662); analytical data in agreement with literature.[1]

Diethyl (*R*/*S*)-(2-(4-(*tert*-butyl)phenyl)propyl)phosphonate ((*R*/*S*)-25):

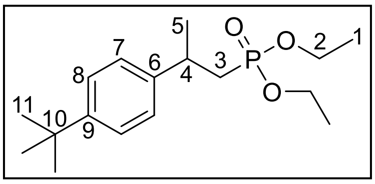

According to **general procedure C**, hydrogention of ***E*-7** (31.0 mg, 0.10 mmol) afforded **(+)-25** as colourless oil (27.2 mg, 0.087 mmol, 87%, e.r. = 97:03); hydrogention of ***Z*-7** (31.0 mg, 0.10 mmol) afforded **(-)-25** as colourless oil (30.6 mg, 0.098 mmol, 98%, e.r. = 01:99). The enantiomeric ratios were determined by HPLC analysis using a ReproSil Chiral OM (0.46 cm * 25 cm) column and *n*-hexane/*i*-propanol (98/2, 1.0 mL/min) as the eluent with detection at 230 nm.

Hydrogention of ***E*-7**: t_R = 8.99 min (major enantiomer), 9.91 min (minor enantiomer); **ODR** (CHCl3, c 1.0): $[\alpha]_D^{26}$ = +20.6°; hydrogention of ***Z*-7**: t_R = 8.98 min (minor enantiomer), 9.87 min (major enantiomer); **ODR** (CHCl3, c 1.0): $[\alpha]_D^{27}$ = -22.2°.

^{1}H NMR (500 MHz, $CDCl_3$): δ = 7.33 - 7.29 (m, 2H, H8), 7.18 - 7.14 (m, 2H, H7), 4.05 - 3.83 (m, 4H, H2), 3.19 (dq, *J* = 11.1, 7.0 Hz, 1H, H4), 2.16 - 1.97 (m, 2H, H3), 1.38 (d, *J* = 6.9 Hz, 3H, H5), 1.30 (s, 9H, H11), 1.21 (dt, *J* = 24.3, 7.0 Hz, 6H, H1) ppm; **^{13}C NMR** (126 MHz, $CDCl_3$): δ = 149.3 (C9), 143.7 (d, J_{CP} = 12.0 Hz, C6), 126.4 (C7), 125.5 (C8), 61.4 (dd, J_{CP} = 24.9, 6.4 Hz, C2), 34.5 (d, J_{CP} = 137.8 Hz, C3), 34.5 (C10), 34.3 (d, J_{CP} = 3.5 Hz, C4), 31.5 (C11), 23.6 (d, J_{CP} = 9.5 Hz, C5), 16.4 (dd, J_{CP} = 6.3, 2.8 Hz, C1) ppm; **^{31}P NMR** (202 MHz, $CDCl_3$): δ = 30.35 ppm; **IR (ATR):** $\tilde{\nu}$ = 3485 (b), 2963 (m), 2906 (w), 2871 (w), 1716 (w), 1648 (b), 1511 (w), 1458 (w), 1393 (w), 1366 (w), 1241 (m), 1163 (w), 1113 (w), 1098 (w), 1053 (s), 1023 (s), 956 (s), 828 (m), 785 (m), 748 (w), 719 (w), 668 (w) cm^{-1}; **HR-ESI-MS**: *m/z*: 335.1773 ($[M+Na]^+$, calcd. for $C_{17}H_{29}NaO_3P^+$: 335.1747), 647.3625 ($[M_2+Na]^+$, cald. for $C_{34}H_{58}NaO_6P_2^+$: 647.3601).

Diethyl (*R*/*S*)-(2-(3-bromophenyl)propyl)phosphonate ((*R*/*S*)-26):

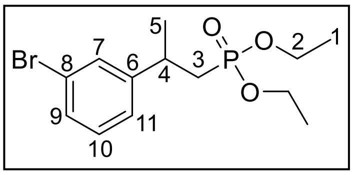

According to **general procedure C**, hydrogention of ***E*-8** (33.3 mg, 0.10 mmol) afforded **(+)-26** as colourless oil (32.1 mg, 0.096 mmol, 96%, e.r. = 97:03); hydrogention of ***Z*-8** (33.3 mg, 0.10 mmol) afforded **(-)-26** as colourless oil (31.1 mg, 0.093 mmol, 93%, e.r. = 01:99). The enantiomeric ratios were determined by HPLC analysis using a Daicel Chiralcel OJ-H (0.46 cm * 25 cm) column and *n*-hexane/*i*-propanol (95/5, 1.0 mL/min) as the eluent with detection at 220 nm.

Hydrogention of ***E*-8**: t_R = 5.59 min (minor enantiomer), 6.38 min (major enantiomer); **ODR** (CHCl3, c 1.0): $[\alpha]_D^{29}$ = +20.4°; hydrogention of ***Z*-8**: t_R = 5.56 min (major enantiomer), 6.43 min (minor enantiomer); **ODR** (CHCl3, c 1.0): $[\alpha]_D^{29}$ = -24.7°.

R_f = XXX (SiO_2, ethyl acetate); **^{1}H NMR** (500 MHz, $CDCl_3$): δ = 7.36 (q, *J* = 1.3 Hz, 1H, H7), 7.34 – 7.30 (m, 1H, H9), 7.18 – 7.14 (m, 2H, H10, H11), 4.05 – 3.87 (m, 4H, H2), 3.17 (dh, *J* = 11.2, 7.0 Hz, 1H, H4), 2.13 – 1.94 (m, 2H, H3), 1.37 (d, *J* = 6.8 Hz, 3H, H5), 1.23 (dt, *J* = 17.6, 7.0 Hz, 6H, H1) ppm; **^{13}C NMR** (126 MHz,

$CDCl_3$): δ = 149.1 (d, J_{CP} = 11.5 Hz, C6), 130.2 (C10), 130.0 (C7), 129.6 (C9), 125.6 (C11), 122.6 (C8), 61.6 (dd, J_{CP} = 13.4, 6.6 Hz, C2), 34.7 (d, J_{CP} = 3.6 Hz, C4), 34.3 (d, J_{CP} = 139.3 Hz, C3), 23.6 (d, J_{CP} = 9.9 Hz, C5), 16.5 (dd, J_{CP} = 6.2, 2.6 Hz. C1) ppm; **^{31}P NMR** (202 MHz, $CDCl_3$): δ = 29.46 ppm; **IR (ATR):** $\tilde{\nu}$ = 3658 (b), 3475 (b), 2979 (w), 2930 (w), 1906 (w), 1727 (b), 1594 (w), 1568 (w), 1477 (w), 1455 (w), 1428 (w), 1392 (w), 1368 (w), 1342 (w), 1241 (m), 1164 (w), 1097 (w), 1052 (s), 1022 (s), 997 (m), 955 (s), 880 (w), 855 (w), 782 (m), 724 (w), 694 (m), 666 (w) cm^{-1}; **HR-ESI-MS**: *m/z*: 359.0204 ([*M*+Na]$^+$, calcd. for $C_{13}H_{18}BrNaO_3P^+$: 359.0205), 693.0559 ([M_2+Na]$^+$, cald. for $C_{26}H_{36}Br_2NaO_6P_2^+$: 693.0539).

Dimethyl (*R*/*S*)-(2-phenylpropyl)phosphonate ((*R*/*S*)-27):

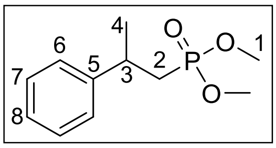

According to **general procedure C**, hydrogention of ***E*-9** (22.6 mg, 0.10 mmol) afforded **(+)-27** as colourless oil (20.0 mg, 0.088 mmol, 88%, e.r. = 97:03); hydrogention of ***Z*-9** (22.6 mg, 0.10 mmol) afforded **(-)-27** as colourless oil (20.5 mg, 0.090 mmol, 90%, e.r. = 01:99). The enantiomeric ratios were determined by HPLC analysis using a ReproSil Chiral OM (0.46 cm * 25 cm) column and *n*-hexane/*i*-propanol (97/3, 1.0 mL/min) as the eluent with detection at 210 nm.

Hydrogention of ***E*-9**: t_R = 22.53 min (minor enantiomer), 25.07 min (major enantiomer); **ODR** (CHCl3, c 0.93): $[\alpha]_D^{28}$ = +22.7°; hydrogention of ***Z*-9**: t_R = 22.63 min (major enantiomer), 25.57 min (minor enantiomer); **ODR** (CHCl3, c 0.74): $[\alpha]_D^{28}$ = -19.5°.

^{1}H NMR (500 MHz, $CDCl_3$): δ = 7.30 (t, *J* = 7.7 Hz, 2H, H7), 7.24 - 7.18 (m, 3H, H6, H8), 3.59 (ddd, *J* = 39.2, 10.8, 0.7 Hz, 6H, H1), 3.20 (dq, *J* = 11.2, 7.0 Hz, 1H, H3), 2.15 - 2.00 (m, 2H, H2), 1.38 (dt, *J* = 6.9, 0.7 Hz, 3H, H4) ppm; **^{13}C NMR** (126 MHz, $CDCl_3$): δ = 146.6 (d, J_{CP} = 12.0 Hz, C5), 128.7 (C7), 126.8 (C6), 126.6 (C8), 52.2 (dd, J_{CP} = 32.1, 6.6 Hz, C1), 34.7 (d, J_{CP} = 3.5 Hz, C5), 33.5 (d, J_{CP} = 138.3 Hz, C2), 23.6 (d, J_{CP} = 9.6 Hz, C4) ppm; **^{31}P NMR** (162 MHz, $CDCl_3$): δ = 32.91 ppm; **IR (ATR):** $\tilde{\nu}$ = 3471 (b), 3029 (w), 2957 (w), 2851 (w), 1637 (b), 1604 (w), 1495 (w), 1454 (w), 1406 (w)1378 (w), 1358 (w), 1287 (w), 1241 (m), 1183 (w), 1053 (s), 1023 (s), 914 (w), 841 (m), 799 (s), 763 (m), 721 (w), 699 (s) cm^{-1}; **HR-ESI-MS**: *m/z*: 251.0820 ([*M*+Na]$^+$, calcd. for $C_{11}H_{17}NaO_3P^+$: 251.0808), 479.1738 ([M_2+Na]$^+$, cald. for $C_{22}H_{34}NaO_6P_2^+$: 479.1723); analytical data in agreement with literature.[6]

Diethyl (*R*/*S*)-(2-(4-methoxyphenyl)propyl)phosphonate ((*R*/*S*)-28):

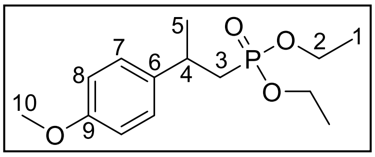

According to **general procedure C**, hydrogention of ***E*-12** (28.4 mg, 0.10 mmol) afforded **(+)-28** as colourless oil (23.7 mg, 0. 83 mmol, 83%, e.r. = 95:05); hydrogention of ***Z*-12** (28.4 mg, 0.10 mmol) afforded **(-)-28** as colourless oil (24.8 mg, 0. 87 mmol, 87%, e.r. = 01:99). The enantiomeric ratios were

determined by HPLC analysis using a Daicel Chiralcel OJ-H (0.46 cm * 25 cm) column and *n*-hexane/*i*-propanol (98/2, 1.0 mL/min) as the eluent with detection at 220 nm.

Hydrogention of ***E*-12**: t_R = 18.65 min (minor enantiomer), 20.52 min (major enantiomer); **ODR** (CHCl3, c 1.0): $[\alpha]_D^{29}$ = +21.3°; hydrogention of ***Z*-12**: t_R = 18.44 min (major enantiomer), 20.90 min (minor enantiomer); **ODR** (CHCl3, c 0.75): $[\alpha]_D^{27}$ = -19.7°.

^{1}H NMR (400 MHz, $CDCl_3$): δ = 7.14 (d, *J* = 8.6 Hz, 2H, H7), 6.84 (d, *J* = 8.5 Hz, 2H, H8), 4.05 – 3.87 (m, 4H, H2), 3.78 (s, 3H, H10), 3.17 (dq, *J* = 13.4, 6.8 Hz, 1H, H4), 2.16 – 1.93 (m, 2H, H3), 1.36 (d, *J* = 6.8 Hz, 3H, H5), 1.24 (dt, *J* = 16.3, 6.9 Hz, 6H, H1) ppm; **^{13}C NMR** (101 MHz, $CDCl_3$): δ = 158.2 (C9), 139.0 (d, J_{CP} = 11.9 Hz, C6), 127.7 (C7), 114.0 (C8), 61.5 (dd, J_{CP} = 18.4, 6.0 Hz, C2), 55.4 (C10), 34.8 (d, J_{CP} = 140.8 Hz, C3), 34.0 (d, J_{CP} = 1.9 Hz, C4), 23.8 (d, J_{CP} = 9.0 Hz, C5), 16.5 (d, J_{CP} = 5.4 Hz, C1) ppm; **^{31}P NMR** (162 MHz, $CDCl_3$): δ = 30.31 ppm; **IR (ATR):** $\tilde{\nu}$ = 3478 (b), 2981 (w), 2934 (w), 2907 (w), 2837 (w), 2302 (w), 1611 (w), 1584 (w), 1513 (s), 1456 (w), 1392 (w), 1293 (w), 1244 (s), 1179 (m), 1098 (w), 1051 (s), 1023 (s), 955 (s), 828 (s), 807 (m), 776 (m), 737 (w), 711 (w), 684 (w) cm^{-1}; **HR-ESI-MS**: *m*/*z*: 309.1237 ($[M+Na]^+$, calcd. for $C_{14}H_{23}NaO_4P^+$: 309.1226), 595.2574 ($[M_2+Na]^+$, cald. for $C_{28}H_{46}NaO_8P_2^+$: 595.2560); analytical data in agreement with literature.[1]

Diethyl (*R*/*S*)-(2-(naphthalen-2-yl)propyl)phosphonate ((*R*/*S*)-29):

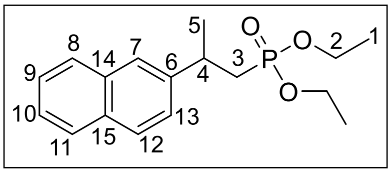

According to **general procedure C**, hydrogention of ***E*-11** (30.3 mg, 0.10 mmol) afforded **(+)-29** as colourless oil (30.4 mg, 0.10 mmol, quant., e.r. = 97:03); hydrogention of ***Z*-11** (30.5 mg, 0.10 mmol) afforded **(-)-29** as colourless oil (30.6 mg, 0.10 mmol, quant, e.r. = 01:99). The enantiomeric ratios were determined by HPLC analysis using a Daicel Chiralcel OJ-H (0.46 cm * 25 cm) column and *n*-hexane/*i*-propanol (98/2, 1.0 mL/min) as the eluent with detection at 230 nm.

Hydrogention of ***E*-11**: t_R = 22.67 min (minor enantiomer), 27.04 min (major enantiomer); **ODR** (CHCl3, c 1.0): $[\alpha]_D^{25}$ = +21.1°; hydrogention of ***Z*-11**: t_R = 22.62 min (major enantiomer), 27.74 min (minor enantiomer); **ODR** (CHCl3, c 1.0): $[\alpha]_D^{27}$ = -28.0°.

^{1}H NMR (600 MHz, $CDCl_3$): δ = 7.81 - 7.77 (m, 3H), 7.66 (d, *J* = 1.8 Hz, 1H, H7), 7.47 - 7.40 (m, 2H), 7.37 (dd, *J* = 8.5, 1.8 Hz, 1H, H13), 4.06 - 3.85 (m, 4H, H2), 3.40 (dq, *J* = 11.1, 6.9 Hz, 1H, H4), 2.26 - 2.07 (m, 2H, H3), 1.48 (d, *J* = 6.9 Hz, 3H, H5), 1.23 (t, *J* = 7.1 Hz, 3H, H1), 1.16 (t, *J* = 7.1 Hz, 3H, H1') ppm; **^{13}C NMR** (151 MHz, $CDCl_3$): δ = 144.2 (d, J_{CP} = 12.2 Hz, C6), 133.7 (C14), 132.4 (C15), 128.3, 127.7 (C7), 127.7, 126.1, 125.5 (C10), 125.4 (C13), 125.0 (C7), 61.5 (dd, J_{CP} = 19.2, 6.6 Hz, C2), 34.9 (d, J_{CP} = 3.1 Hz, C4) 34.4 (d, J_{CP} = 138.0 Hz, C3), 23.5 (d, J_{CP} = 9.2 Hz, C5), 16.4 (t, J_{CP} = 6.6 Hz, C1) ppm; **^{31}P NMR** (202 MHz, $CDCl_3$): δ = 30.13 ppm; **IR (ATR):** $\tilde{\nu}$ = 3476 (b), 3053 (w), 2979 (m), 2932 (w), 2904 (w), 2349 (w), 2309

(w), 1634 (w), 1601 (w), 1508 (w), 1478 (w), 1455 (w), 1391 (w), 1228 (m), 1163 (w), 1127 (w), 1097 (w), 1052 (s), 1020 (s), 951 (s), 892 (w), 858 (m), 818 (s), 778 (m), 747 (s), 701 (m) cm^{-1}; **HR-ESI-MS**: *m/z*: 329.1292 ($[M+Na]^+$, calcd. for $C_{17}H_{23}NaO_3P^+$: 329.1277), 635.2679 ($[M_2+Na]^+$, cald. for $C_{34}H_{46}NaO_6P_2^+$: 635.2662); analytical data in agreement with literature.[1]

Diethyl (*R*/*S*)-(2-phenylbutyl)phosphonate ((*R*/*S*)-30):

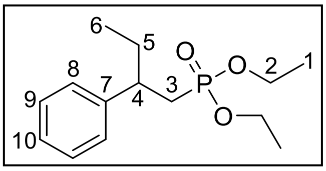

According to **general procedure C**, hydrogention of ***E*-14** (26.8 mg, 0.10 mmol) afforded **(+)-30** as colourless oil (26.5 mg, 0.098 mmol, 98%, e.r. = 97:03); hydrogention of ***Z*-14** (26.7 mg, 0.10 mmol) afforded **(-)-30** as colourless oil (24.2 mg, 0.090 mmol, 90%, e.r. = 01:99). The enantiomeric ratios were determined by HPLC analysis using a Daicel Chiralcel OJ-H (0.46 cm * 25 cm) column and *n*-hexane/*i*-propanol (98/2, 1.0 mL/min) as the eluent with detection at 210 nm.
Hydrogention of ***E*-14**: t_R = 8.10 min (minor enantiomer), 9.68 min (major enantiomer); **ODR** (CHCl3, c 1.0): $[\alpha]_D^{27}$ = +8.0°; hydrogention of ***Z*-14**: t_R = 8.03 min (major enantiomer), 9.75 min (minor enantiomer); **ODR** (CHCl3, c 1.0): $[\alpha]_D^{27}$ = -13.2°.
R_f = 0.50 (SiO_2, ethyl acetate); **^{1}H NMR** (400 MHz, $CDCl_3$): δ = 7.33 - 7.27 (m, 2H, H9), 7.24 - 7.17 (m, 3H, H8, H10), 4.03 - 3.73 (m, 4H, H2), 2.99 - 2.87 (m, 1H, H4), 2.21 - 2.03 (m, 2H, H3), 1.86 (dtd, *J* = 14.6, 7.3, 5.0 Hz, 1H, H5), 1.64 (ddq, *J* = 14.5, 9.8, 7.3 Hz, 1H, H5'), 1.18 (dt, *J* = 25.1, 7.0 Hz, 6H, H1), 0.77 (t, *J* = 7.3 Hz, 3H, H6) ppm; **^{13}C NMR** (126 MHz, $CDCl_3$): δ = 144.5 (d, J_{CP} = 7.8 Hz, C7), 128.4 (C9), 127.7 (C8), 126.5 (C10), 61.4 (dd, J_{CP} = 18.6, 6.0 Hz, C2), 42.0 (d, J_{CP} = 2.9 Hz, C4), 33.0 (d, J_{CP} = 139.0 Hz, C3), 30.9 (d, J_{CP} = 12.5 Hz, C5), 16.4 (dd, J_{CP} = 6.0, 2.5 Hz, C1), 11.9 (C6) ppm; **^{31}P NMR** (162 MHz, $CDCl_3$): δ = 30.57 ppm; **IR (ATR):** $\tilde{\nu}$ = 3479 (b), 3063 (w), 3029 (w), 2973 (w), 2932 (w), 2875 (w), 2301 (w), 1643 (b), 1604 (w), 1495 (w), 1455 (w), 1392 (w), 1368 (w), 1292 (w), 1241 (m), 1163 (w), 1098 (w), 1054 (s), 1023 (s), 955 (s), 872 (w), 853 (w), 803 (m), 756 (m), 699 (s) cm^{-1}; **HR-ESI-MS**: *m/z*: 293.1290 ($[M+Na]^+$, calcd. for $C_{14}H_{23}NaO_3P^+$: 293.1277), 563.2670 ($[M_2+Na]^+$, cald. for $C_{28}H_{46}NaO_6P_2^+$: 563.2662); analytical data in agreement with literature.[1]

Diethyl (*R*/*S*)-(2-(2-fluorophenyl)propyl)phosphonate ((*R*/*S*)-31):

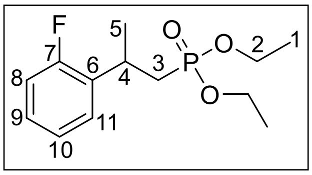

According to **general procedure C**, hydrogention of ***E*-16** (27.2 mg, 0.10 mmol) afforded **(+)-31** as colourless oil (27.4 mg, 0.100 mmol, quant, e.r. = 94:06); hydrogention of ***Z*-16** (27.2 mg, 0.10 mmol) afforded **(-)-31** as colourless oil (26.9 mg, 0.098 mmol, 98%, e.r. > 01:99). The enantiomeric ratios were determined by HPLC analysis using a Daicel Chiralcel OJ-H (0.46 cm * 25 cm) column and *n*-hexane/*i*-propanol (99/1, 1.0 mL/min) as the eluent with detection at 254 nm.

Hydrogention of ***E*-16**: t_R = 10.60 min (minor enantiomer), 11.65 min (major enantiomer); **ODR** (CHCl3, c 1.0): $[\alpha]_D^{26}$ = +18.4°; hydrogention of ***Z*-16**: t_R = 10.37 min (only enantiomer); **ODR** (CHCl3, c 1.0): $[\alpha]_D^{26}$ = -15.0°.

^{1}H NMR (500 MHz, $CDCl_3$): δ = 7.22 (td, *J* = 7.6, 1.8 Hz, 1H, H11), 7.17 (dddd, *J* = 8.2, 7.1, 5.2, 1.7 Hz, 1H, H9), 7.07 (td, *J* = 7.5, 1.3 Hz, 1H, H10), 6.99 (ddd, *J* = 10.8, 8.1, 1.2 Hz, 1H, H8), 4.05 - 3.90 (m, 4H, H2), 3.46 (dq, *J* = 11.5, 7.0 Hz, 1H, H4), 2.19 (ddd, *J* = 18.3, 15.3, 6.6 Hz, 1H, H3), 2.06 (ddd, *J* = 18.0, 15.3, 7.7 Hz, 1H, H3'), 1.40 (d, *J* = 7.0 Hz, 3H, H5), 1.22 (td, *J* = 7.0, 2.6 Hz, 6H, H1) ppm; **^{13}C NMR** (126 MHz, $CDCl_3$): δ = 160.8 (d, J_{CF} = 245.6 Hz, C7), 133.1 (dd, J_{CP} = 13.8, 11.4 Hz, C6), 128.5 (d, J_{CF} = 5.2 Hz, C11), 128.0 (d, J_{CF} = 8.4 Hz, C9), 124.3 (d, J_{CF} = 3.5 Hz, C10), 115.7 (d, J_{CF} = 22.5 Hz, C8), 61.5 (dd, J_{CP} = 9.0, 6.4 Hz, C2), 32.8 (d, J_{CP} = 139.3 Hz, C3), 29.2 (dd, J_{CP} = 3.6, J_{CF} = 1.9 Hz, C4), 22.1 (dd, J_{CP} = 9.8, J_{CF} = 1.4 Hz, C5), 16.4 (dd, J_{CP} = 6.2, 1.6 Hz, C1) ppm; **^{19}F NMR** (282 MHz, $CDCl_3$): δ = -117.98 ppm; **^{31}P NMR** (202 MHz, $CDCl_3$): δ = 29.86 ppm; **IR (ATR):** $\tilde{\nu}$ = 3478 (b), 2981 (w), 2934 (w), 1717 (w), 1616 (w), 1584 (w), 1492 (m), 1453 (w), 1392 (w), 1368 (w), 1230 (m), 1052 (s), 1022 (s), 957 (s), 854 (w), 823 (m), 805 (w), 755 (s) cm^{-1}; **HR-ESI-MS**: *m/z*: 297.1047 ($[M+Na]^+$, calcd. for $C_{13}H_{20}FNaO_3P^+$: 297.1026), 571.2185 ($[M_2+Na]^+$, cald. for $C_{26}H_{40}F_2NaO_6P_2^+$: 571.2160).

Diethyl (*R*/*S*)-(2-(2-tolyl)propyl)phosphonate ((*R*/*S*)-32):

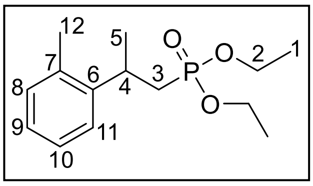

According to **general procedure C**, hydrogention of ***E*-15** (26.8 mg, 0.10 mmol) afforded **(+)-32** as colourless oil (25.2 mg, 0.093 mmol, 93%, e.r. = 93:07); hydrogention of ***Z*-15** (26.8 mg, 0.10 mmol) afforded **(-)-32** as colourless oil (24.6 mg, 0.091 mmol, 91%, e.r. > 01:99). The enantiomeric ratios were determined by HPLC analysis using a Daicel Chiralcel OJ-H (0.46 cm * 25 cm) column and *n*-hexane/*i*-propanol (98/2, 1.0 mL/min) as the eluent with detection at 210 nm.

Hydrogention of ***E*-15**: t_R = 9.55 min (minor enantiomer), 18.32 min (major enantiomer); **ODR** (CHCl3, c 1.0): $[\alpha]_D^{27}$ = +13.9°; hydrogention of ***Z*-15**: t_R = 9.58 min (only enantiomer); **ODR** (CHCl3, c 1.0): $[\alpha]_D^{27}$ = -15.7°.

^{1}H NMR (500 MHz, $CDCl_3$): δ = 7.21 - 7.15 (m, 2H, H10, H11), 7.12 (dd, *J* = 7.4, 1.4 Hz, 1H, H8), 7.08 (ddd, *J* = 7.8, 6.1, 2.1 Hz, 1H, H9), 4.07 - 3.86 (m, 4H, H2), 3.55 - 3.43 (m, 1H, H4), 2.37 (s, 3H, H12), 2.19 - 1.98 (m, 2H, H3), 1.35 (d, *J* = 6.7 Hz, 3H, H5), 1.23 (dt, *J* = 13.3, 6.6 Hz, 6H, H1) ppm; **^{13}C NMR** (126 MHz, $CDCl_3$): δ = 145.0 (d, J_{CP} = 10.0 Hz, C6), 135.1 (C7), 130.5 (C8), 126.4 (C10), 126.1 (C9), 125.3 (C11), 61.5 (d, J_{CP} = 16.2 Hz, C2), 34.1 (d, J_{CP} = 136.7 Hz, C3), 29.5 (C4), 23.0 (d, J_{CP} = 6.4 Hz, C5), 19.6 (C12), 16.5 (d, J_{CP} = 3.0 Hz, C1) ppm; **^{31}P NMR** (162 MHz, $CDCl_3$): δ = 30.58 ppm; **IR (ATR):** $\tilde{\nu}$ = 3476 (b), 2974 (w), 2929 (w), 1651 (b), 1605 (w), 1491 (w), 1456 (w), 1392 (w), 1287 (w), 1231 (m), 1163 (w), 1097 (w), 1050 (s), 1022 (s), 955 (s), 854 (w), 831 (w), 810 (w), 758 (s), 727 (m) cm^{-1}; **HR-ESI-MS**: *m/z*:

293.1293 ([M+Na]$^+$, calcd. for $C_{14}H_{23}NaO_3P^+$: 293.1277), 563.2681 ([M_2+Na]$^+$, cald. for $C_{28}H_{46}NaO_6P_2^+$: 563.2662); analytical data in agreement with literature.[1]

Diethyl (*R*/*S*)-(2-(2-chlorophenyl)propyl)phosphonate ((*R*/*S*)-33):

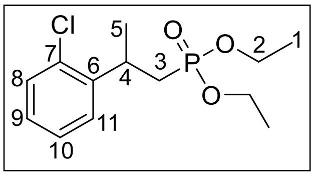

According to **general procedure C**, hydrogention of ***E*-17** (28.9 mg, 0.10 mmol) afforded **(+)-33** as colourless oil (27.5 mg, 0.095 mmol, 95%, e.r. = 96:04); hydrogention of ***Z*-17** (28.9 mg, 0.10 mmol) afforded **(-)-33** as colourless oil (27.5 mg, 0.095 mmol, 95%, e.r. > 01:99). The enantiomeric ratios were determined by HPLC analysis using a Daicel Chiralcel OJ-H (0.46 cm * 25 cm) column and *n*-hexane/*i*-propanol (97/3, 1.0 mL/min) as the eluent with detection at 230 nm.

Hydrogention of ***E*-17**: t_R = 8.53 min (minor enantiomer), 13.40 min (major enantiomer); **ODR** (CHCl3, c 1.0): $[\alpha]_D^{27}$ = +0.2°; hydrogention of ***Z*-17**: t_R = 8.41 min (major enantiomer), 13.82 min (minor enantiomer); **ODR** (CHCl3, c 1.0): $[\alpha]_D^{27}$ = -0.7°.

^{1}H NMR (500 MHz, $CDCl_3$): δ = 7.33 (dd, J = 7.9, 1.3 Hz, 1H, H8), 7.26 (dd, J = 7.8, 1.9 Hz, 1H, H11), 7.22 (ddd, J = 7.5, 6.9, 1.3 Hz, 1H, H10), 7.13 (ddd, J = 8.0, 7.1, 1.9 Hz, 1H, H9), 4.08 - 3.96 (m, 4H, H2), 3.77 - 3.67 (m, 1H, H4), 2.17 (ddd, J = 18.7, 15.4, 5.5 Hz, 1H, H3), 2.00 (ddd, J = 17.9, 15.3, 8.6 Hz, 1H, H3'), 1.39 (d, J = 7.0 Hz, 3H, H5), 1.25 (dt, J = 15.9, 7.0 Hz, 6H, H1) ppm; **^{13}C NMR** (126 MHz, $CDCl_3$): δ = 143.8 (d, J_{CP} = 13.2 Hz, C6), 133.3 (C7), 129.8 (C8), 127.6 (C9), 127.5 (C11), 127.2 (C10), 61.6 (dd, J_{CP} = 16.0, 6.5 Hz, C2), 33.0 (d, J_{CP} = 139.0 Hz, C3), 30.9 (d, J_{CP} = 3.1 Hz, C4), 21.9 (d, J_{CP} = 7.5 Hz, C5), 16.5 (t, J_{CP} = 6.4 Hz, C1) ppm; **^{31}P NMR** (202 MHz, $CDCl_3$): δ = 29.62 ppm; **IR (ATR):** $\tilde{\nu}$ = 3474 (b), 2980 (w), 2934 (w), 2907 (w), 1640 (b), 1572 (w), 1476 (w), 1442 (w), 1392 (w), 1368 (w), 1281 (w), 1246 (m), 1163 (w), 1127 (w), 1098 (w), 1022 (s), 956 (s), 833 (w), 787 (m), 753 (s), 731 (w), 684 (m), 674 (w) cm^{-1}; **HR-ESI-MS**: m/z: 313.0741 ([M+Na]$^+$, calcd. for $C_{13}H_{20}ClNaO_3P^+$: 313.0731), 603.1588 ([M_2+Na]$^+$, cald. for $C_{26}H_{40}Cl_2NaO_6P_2^+$: 603.1577).

Diethyl (*R*/*S*)-(2-(2-bromophenyl)propyl)phosphonate ((*R*/*S*)-34:

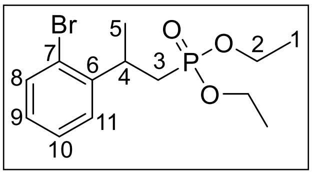

According to **general procedure C**, hydrogention of ***E*-18** (33.3 mg, 0.10 mmol) afforded **(-)-34** as colourless oil (31.5 mg, 0.094 mmol, 94%, e.r. = 96:04); hydrogention of ***Z*-18** (33.3 mg, 0.10 mmol) afforded **(+)-34** as colourless oil (31.5 mg, 0.089 mmol, 89%, e.r. > 01:99). The enantiomeric ratios were determined by HPLC analysis using a Daicel Chiralcel OJ-H (0.46 cm * 25 cm) column and *n*-hexane/*i*-propanol (95/5, 1.0 mL/min) as the eluent with detection at 230 nm.

Hydrogention of ***E*-18**: t_R = 6.89 min (minor enantiomer), 13.57 min (major enantiomer); **ODR** (CHCl3, c 1.0): $[\alpha]_D^{27}$ = -5.3°; hydrogention of ***Z*-18**: t_R = 6.93 min (only enantiomer); **ODR** (CHCl3, c 1.0): $[\alpha]_D^{28}$ = +5.4°.

^{1}H NMR (500 MHz, $CDCl_3$): δ = 7.52 (dd, *J* = 8.0, 1.2 Hz, 1H, H8), 7.29 - 7.26 (m, 2H, H10), 7.24 (dd, *J* = 7.8, 2.2 Hz, 1H, H11), 7.05 (ddd, *J* = 8.0, 6.8, 2.2 Hz, 1H, H9), 4.08 - 3.98 (m, 4H, H2), 3.70 (dddd, *J* = 12.3, 8.8, 7.0, 5.4 Hz, 1H, H4), 2.16 (ddd, *J* = 18.8, 15.3, 5.4 Hz, 1H, H3), 1.99 (ddd, *J* = 17.8, 15.3, 8.7 Hz, 1H, H3'), 1.38 (d, *J* = 6.9 Hz, 3H, H5), 1.26 (dt, *J* = 19.0, 7.1 Hz, 6H, H1) ppm; **^{13}C NMR** (126 MHz, $CDCl_3$): δ = 145.4 (d, J_{CP} = 13.5 Hz, C6), 133.1 (C8), 127.9 (C9), 127.9 (C10), 127.4 (C11), 124.0 (C7), 61.6 (dd, J_{CP} = 18.0, 6.6 Hz, C2), 33.5 (d, J_{CP} = 3.0 Hz, C4), 33.1 (d, J_{CP} = 138.9 Hz, C3), 22.1 (d, J_{CP} = 7.2 Hz, C5), 16.5 (dd, J_{CP} = 8.2, 6.1 Hz, C1) ppm; **^{31}P NMR** (202 MHz, $CDCl_3$): δ = 29.44 ppm; **IR (ATR):** $\tilde{\nu}$ = 3469 (b), 3060 (w), 2980 (w), 2980 (w), 2932 (w), 2906 (w), 1647 (b), 1591 (w), 1568 (w), 1472 (w), 1440 (w), 1392 (w), 1368 (w), 1245 (m), 1163 (w), 1097 (w), 1052 (s), 1019 (s), 955 (s), 833 (w), 806 (w), 784 (m), 753 (s), 726 (w) cm^{-1}; **HR-ESI-MS**: *m/z*: 357.0228 ($[M+Na]^+$, calcd. for $C_{13}H_{20}BrNaO_3P^+$: 357.0226), 693.0550 ($[M_2+Na]^+$, cald. for $C_{26}H_{40}Br_2NaO_6P_2^+$: 693.0539).

Diisopropyl (*R*/*S*)-(2-phenylpropyl)phosphonate ((*R*/*S*)-35):

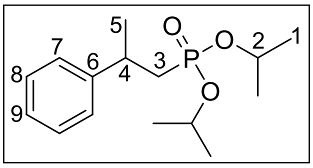

According to **general procedure C**, hydrogention of ***E*-10** (2802 mg, 0.10 mmol) afforded **(+)-35** as colourless oil (28.5 mg, 0.100 mmol, quant.); hydrogention of ***Z*-10** (28.2 mg, 0.10 mmol) afforded **(-)-35** as colourless oil (28.5 mg, 0.100 mmol, quant.). The enantiomeric ratios could not be determined by HPLC analysis due to decomposition of the products during the analysis.

Hydrogention of ***E*-10**: **ODR** (CHCl3, c 1.0): $[\alpha]_D^{28}$ = +17.5°.

Hydrogention of ***Z*-10**: **ODR** (CHCl3, c 1.0): $[\alpha]_D^{27}$ = -20.0°.

^{1}H NMR (500 MHz, $CDCl_3$): δ = 7.31 - 7.27 (m, 2H, H8), 7.23 - 7.17 (m, 3H, H7, H9), 4.64 (tdd, *J* = 13.6, 9.8, 6.2 Hz, 2H, H2), 3.20 (tq, *J* = 14.1, 7.1 Hz, 1H, H4), 2.12 - 1.94 (m, 2H, H3), 1.39 (d, *J* = 7.0 Hz, 3H, H5), 1.28 (dd, *J* = 6.1, 4.5 Hz, 6H, H1), 1.23 (dd, *J* = 17.8, 6.1 Hz, 6H, H1') ppm; **^{13}C NMR** (126 MHz, $CDCl_3$): δ = 147.2 (d, J_{CP} = 12.9 Hz, C6), 128.6 (C8), 126.8 (C7), 126.4 (C9), 70.0 (dd, J_{CP} = 10.0, 6.2 Hz, C2), 35.8 (d, J_{CP} = 139.5 Hz, C3), 35.0 (d, J_{CP} = 3.4 Hz, C4), 24.1 (ddd, J_{CP} = 14.5, 6.9, 3.9 Hz, C1), 23.5 (d, J_{CP} = 8.0 Hz, C5) ppm; **^{31}P NMR** (162 MHz, $CDCl_3$): δ = 28.23 ppm; **IR (ATR):** $\tilde{\nu}$ = 3454 (b), 3029 (w), 2978 (w), 2934 (w), 2876 (w), 1604 (w), 1495 (w) 1455 (w), 1385 (w), 1374 (w), 1245 (m), 1226 (m), 1177 (w), 1141 (w), 1107 (m), 1005 (s), 974 (s), 896 (m), 886 (m), 805 (w), 760 (m), 699 (s) cm^{-1}; **HR-ESI-MS**: *m/z*: 307.1449 ($[M+Na]^+$, calcd. for $C_{15}H_{25}NaO_3P^+$: 307.1434), 591.2989 ($[M_2+Na]^+$, cald. for $C_{30}H_{50}NaO_6P_2^+$: 591.2975).

“One-pot” Isomerisation and Hydrogenation of Vinylphosphonate *E*-1

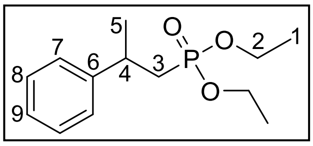

E-vinylphosphonate ***E*-1** (25.4 mg, 0.10 mmol, 1.00 eq.) and anthracene (0.9 mg, 0.005 mmol, 5 mol%) were dissolved in acetonitrile (1.5 mL) and the solution was stirred under UV light irradiation at 365 nm at ambient temperature for 18 h. The solution was filtered through a syringe filter (PTFE, 0.2 µm), added to a vial and the solvent was evaporated. In a glovebox, a pre-stirred solution of $Rh(COD)_2BF_4$ (1.3 mg, 0.0032 mmol, 3.2 mol%) and (S_C,S_P)-WalPhos (2.3 mg, 0.0035 mmol, 3.5 mol%) in DCM (1 mL) was added and the vial was transferred to an autoclave. The autoclave was charged with H_2 (10 bar) and the solution was stirred at room temperature for 24 h. After carefully releasing the pressure and evaporation of the solvent, *n*-pentane (3 mL) was added and filtration through a glass microfiber filter with subsequent elution with *n*-pentane (2 x 2 mL) yielded the product **(-)-19** as clear oil (22.3 mg, 0.087 mmol, 87%, e.r. = 07:93). The enantiomeric ratio was determined by HPLC analysis using a Daicel Chiralcel OJ-H (0.46 cm * 25 cm) column and *n*-hexane/*i*-propanol (98/2, 1.0 mL/min) as the eluent with detection at 210 nm: t_R = 11.63 min (major enantiomer), 13.67 min (minor enantiomer); **ODR** (CHCl3, c 0.75): $[\alpha]_D^{26}$ = -12.5°.

Catalyst Screening for the *E* → Z Isomerisation of Vinylphosphonates

Vinylphosphonate ***E*-1** (25.4 mg, 0.10 mmol, 1.00 eq.) and the specified catalyst (0.005 mmol, 5 mol%) were dissolved in acetonitrile (1.5 mL) and the solution was stirred under UV or visible light irradiation at the given wavelength at ambient temperature for 18 h. After removal of the solvent, ***E*-1** and ***Z*-1** were isolated by column chromatography (SiO_2, ethyl acetate). Yields were determined by mass recovery; *Z*:*E* ratios were determined by integration of peaks in the ^{31}P NMR spectrum and confirmed by integration the olefinic proton peaks in the ^{1}H NMR spectrum of both isomers.

Table S1: Catalyst screening for the *E* → Z isomerisation of vinylphosphonates.

entry	catalyst	irradiation wavelength/ nm	isolated yield/ %	*Z*:*E* ratio
**1**	$Ir(ppy)_3$	450	quant.	13:87
**2**	(-)-riboflavin	402	quant.	66:34
**3**	benzil	402	98	25:75
**4**	thioxanthone	402	94	86:14
**5**	benzophenone	365	quant.	86:14
**6**	anthracene	365	quant.	92:08

Reaction Optimisation for the *E* → Z Isomerisation of Vinylphosphonates

Vinylphosphonate ***E*-1** (25.4 mg, 0.10 mmol, 1.00 eq.) and anthracene (0.9 mg, 0.005 mmol, 5 mol%) were dissolved in the specified solvent (1.5 mL) and the solution was stirred under UV light irradiation at 365 nm at ambient temperature. After removal of the solvent, ***E*-1** and ***Z*-1** were isolated by column chromatography (SiO_2, ethyl acetate). Yields were determined by mass recovery; *Z*:*E* ratios were determined by integration of peaks in the ^{31}P NMR spectrum and confirmed by integration the olefinic proton peaks in the ^{1}H NMR spectrum of both isomers.

Table S2: Reaction optimisation for the *E* → Z isomerisation of vinylphosphonates.

entry	solvent	irradiation time/ h	atmosphere	isolated yield/ %	*Z*:*E* ratio
**1**	acetonitrile	18	air	quant.	92:08
**2**	cyclohexane	18	air	quant.	38:62
**3** [a]	dichloromethane	18	air	n.d.	84:16
**4**	toluene	18	air	quant.	68:32
**5**	acetonitrile	3	air	98	66:34
**6** [a]	acetonitrile	24	air	n.d.	90:10
**7**	acetonitrile	18	oxygen	94	83:17
**8**	acetonitrile	18	argon	84	91:09

a) *Z*:*E* ratio determined from crude reaction mixture.

Control Experiments for the *E* → Z Isomerisation of Vinylphosphonates

According to **general procedure B**, control experiments with vinylphosphonate ***E*-1** (25.4 mg, 0.10 mmol, 1.00 eq.) and anthracene (0.9 mg, 0.005 mmol, 5 mol%) were performed in the dark, without catalyst, and in the dark without catalyst. ***E*-1** and ***Z*-1** were isolated by column chromatography (SiO_2, ethyl acetate). Yields were determined by mass recovery; *Z*:*E* ratios were determined by integration of peaks in the ^{31}P NMR spectrum and confirmed by integration the olefinic proton peaks in the ^{1}H NMR spectrum of both isomers.

Table S3: Control experiments for the *E* → Z isomerisation of vinylphosphonates.

entry	catalyst	irradiation wavelength/ nm	isolated yield/ %	*Z*:*E* ratio
**1**	anthracene	-	96	0:100
**2**	-	365	quant.	02:98
**3**	-	-	quant.	0:100

Verification of the Photostationary State

According to **general procedure B**, control experiments with vinylphosphonates ***E*-** and ***Z*-4** (33.3 mg, 0.10 mmol, 1.00 eq.) and anthracene (0.9 mg, 0.005 mmol, 5 mol%) were performed. ***E*-4** and ***Z*-4** were isolated by column chromatography (SiO_2, ethyl acetate). Yields were determined by mass recovery; *Z*:*E* ratios were determined by integration of peaks in the ^{31}P NMR spectrum and confirmed by integration the olefinic proton peaks in the ^{1}H NMR spectrum of both isomers.

Table S4: Isomerisation of vinylphosphonates *E*- and *Z*-4.

entry	starting geometry	isolated yield/ %	*Z*:*E* ratio
**1**	***E*** only	87	89:11
**2**	***Z*** only	85	90:10
**3**	***E*:*Z*** 1:1	85	90:10

The *Z*:*E* ratios resulting from exposure of vinylphosphonate ***Z*-4** or a 1:1 mixture of ***E*-** and ***Z*-4** to the standard isomerisation conditions verify that the obtained *Z*:*E* ratios represent photostationary state compositions.

HPLC traces

Diethyl (2-phenylpropyl)phosphonate (19):

HPLC trace: racemic sample: **19**

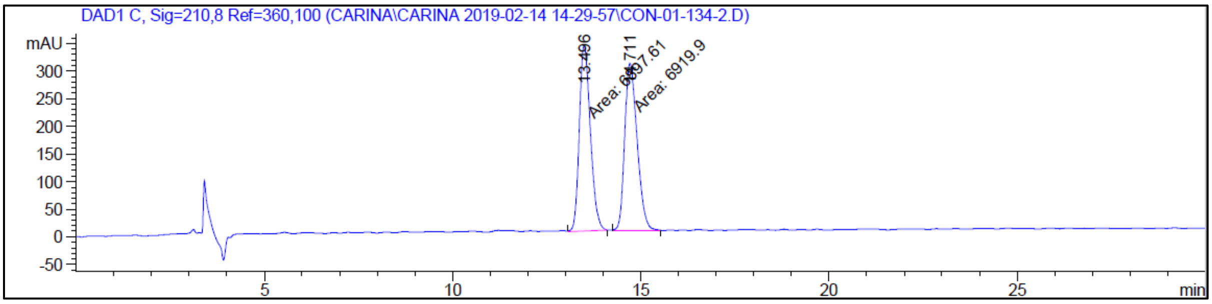

Peak #	RetTime [min]	Type	Width [min]	Area [mAU*s]	Height [mAU]	Area %
1	13.496	MM	0.3417	6897.60693	336.47971	49.9193
2	14.711	MM	0.3816	6919.90283	302.26364	50.0807

HPLC trace: Hydrogenation of the *E*-isomer: **(+)-19**

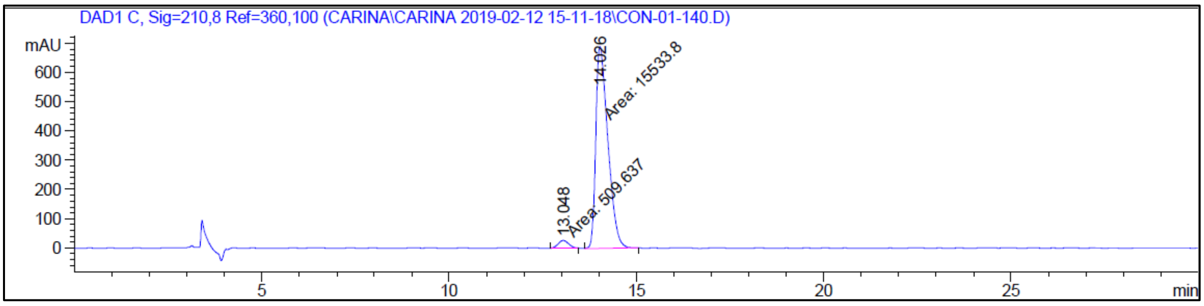

Peak #	RetTime [min]	Type	Width [min]	Area [mAU*s]	Height [mAU]	Area %
1	13.048	MM	0.3221	509.63739	26.37405	3.1766
2	14.026	MM	0.3756	1.55338e4	689.20227	96.8234

HPLC trace: Hydrogenation of the *Z*-isomer: **(-)-19**

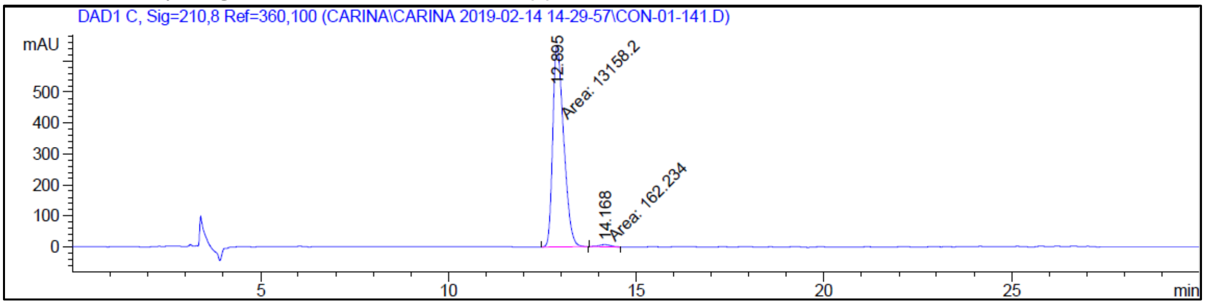

Peak #	RetTime [min]	Type	Width [min]	Area [mAU*s]	Height [mAU]	Area %
1	12.895	MM	0.3376	1.31582e4	649.64325	98.7821
2	14.168	MM	0.3887	162.23425	6.95633	1.2179

Hydrogenation on a 1.0 mmol scale

Diethyl (2-phenylpropyl)phosphonate (19):

HPLC trace: Hydrogenation of the *E*-isomer: **(+)-19**

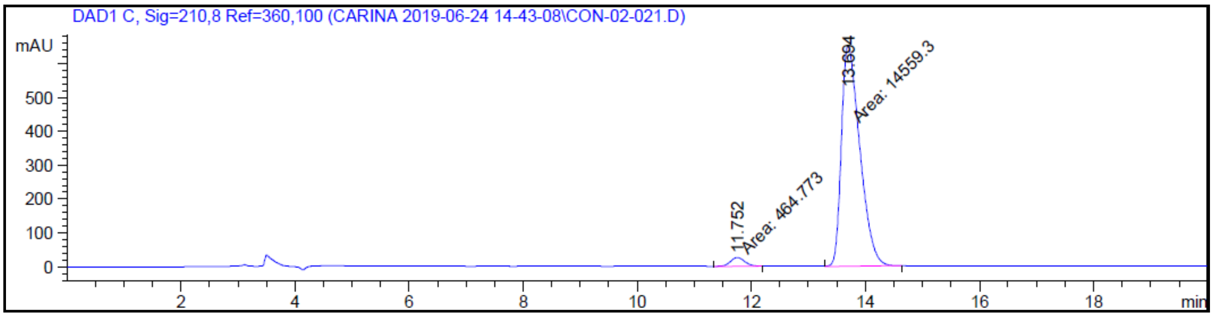

Peak #	RetTime [min]	Type	Width [min]	Area [mAU*s]	Height [mAU]	Area %
1	11.752	MM	0.2957	464.77316	26.19628	3.0935
2	13.694	MM	0.3743	1.45593e4	648.36182	96.9065

HPLC trace: Hydrogenation of the *Z*-isomer: **(-)-19**

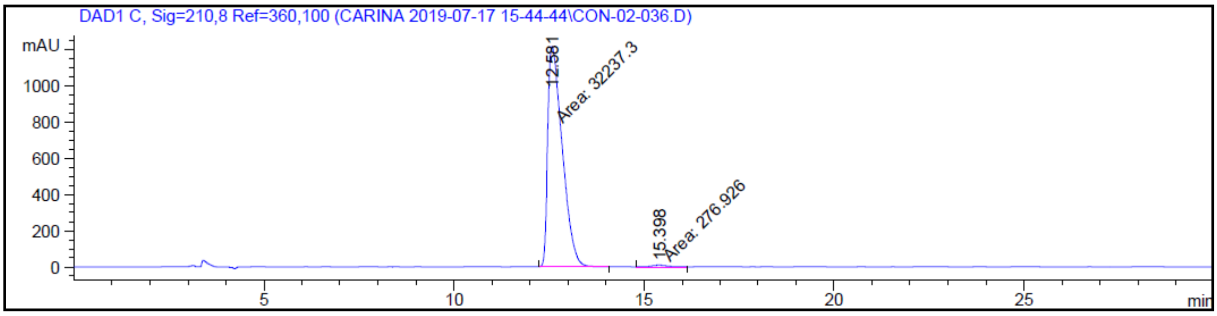

Peak #	RetTime [min]	Type	Width [min]	Area [mAU*s]	Height [mAU]	Area %
1	12.581	MM	0.4416	3.22373e4	1216.56042	99.1483
2	15.398	MM	0.4983	276.92630	9.26319	0.8517

Hydrogenation using the opposite catalyst enantiomer

Diethyl (2-phenylpropyl)phosphonate (19):

HPLC trace: Hydrogenation of the *E*-isomer using (R_c,R_p)-Walphos: **(-)-19**

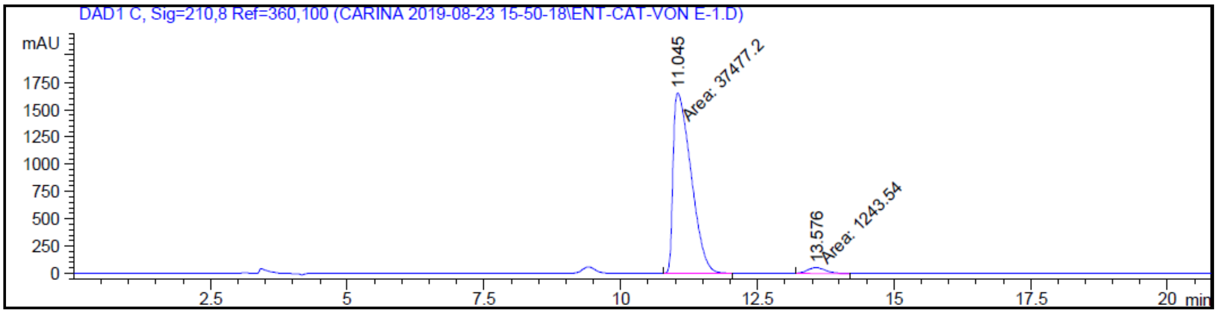

Peak #	RetTime [min]	Type	Width [min]	Area [mAU*s]	Height [mAU]	Area %
1	11.045	MM	0.3780	3.74772e4	1652.40442	96.7884
2	13.576	MM	0.3840	1243.53992	53.97715	3.2116

HPLC trace: Hydrogenation of the *Z*-isomer using (R_c,R_p)-Walphos: **(+)-19**

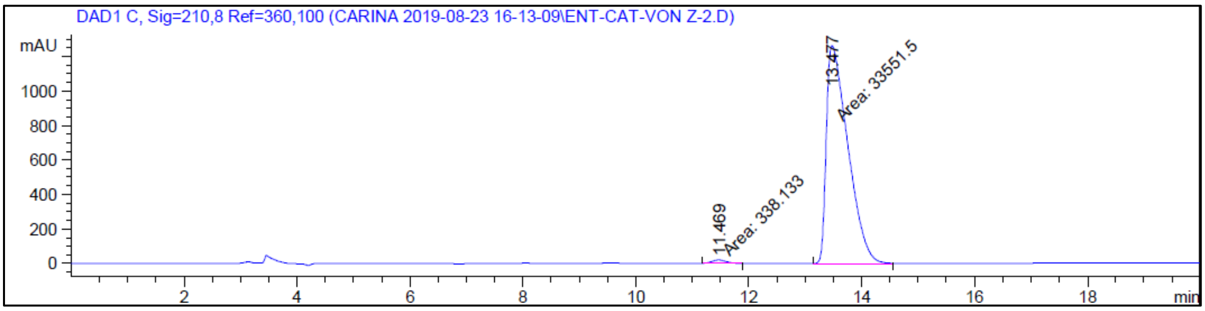

Peak #	RetTime [min]	Type	Width [min]	Area [mAU*s]	Height [mAU]	Area %
1	11.469	MM	0.2876	338.13330	19.59572	0.9977
2	13.477	MM	0.4420	3.35515e4	1265.20679	99.0023

One-pot Isomerisation and Hydrogenation: Diethyl (2-phenylpropyl)phosphonate: (-)-19

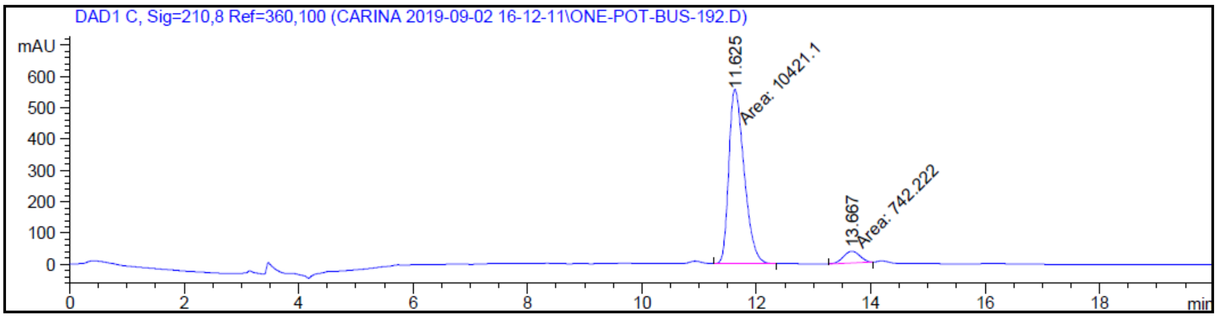

Peak #	RetTime [min]	Type	Width [min]	Area [mAU*s]	Height [mAU]	Area %
1	11.625	MM	0.3109	1.04211e4	558.64392	93.3513
2	13.667	MM	0.3296	742.22211	37.52897	6.6487

Diethyl (2-(4-fluorophenyl)propyl)phosphonate (20):

HPLC trace: racemic sample: **20**

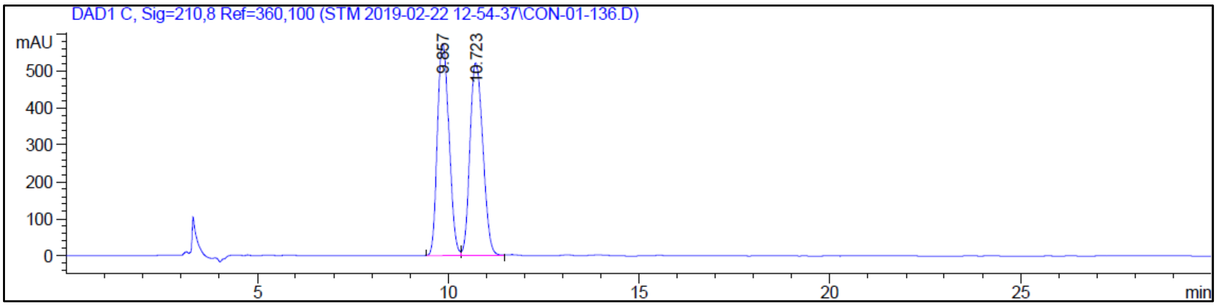

Peak #	RetTime [min]	Type	Width [min]	Area [mAU*s]	Height [mAU]	Area %
1	9.857	BV	0.3389	1.21530e4	574.19360	49.8414
2	10.723	VB	0.3754	1.22303e4	521.84021	50.1586

HPLC trace: Hydrogenation of the *E*-isomer: **(+)-20**

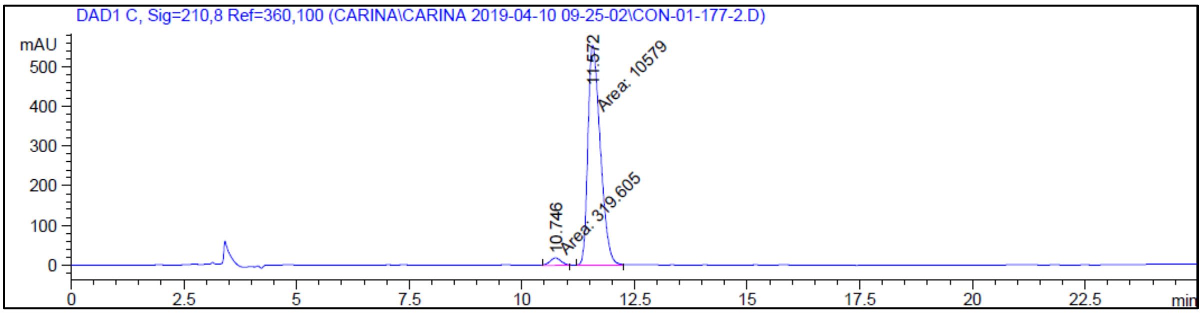

Peak #	RetTime [min]	Type	Width [min]	Area [mAU*s]	Height [mAU]	Area %
1	10.746	MM	0.2847	319.60501	18.71310	2.9325
2	11.572	MM	0.3185	1.05790e4	553.56372	97.0675

HPLC trace: Hydrogenation of the *Z*-isomer: **(-)-20**

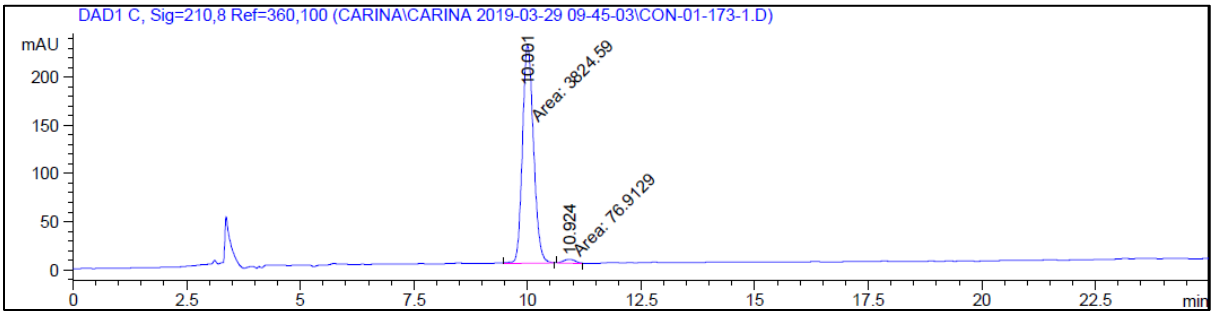

Peak #	RetTime [min]	Type	Width [min]	Area [mAU*s]	Height [mAU]	Area %
1	10.001	MM	0.2816	3824.58862	226.37099	98.0286
2	10.924	MM	0.3099	76.91286	4.13630	1.9714

Diethyl (2-(4-chlorophenyl)propyl)phosphonate (21):

HPLC trace: racemic sample: **21**

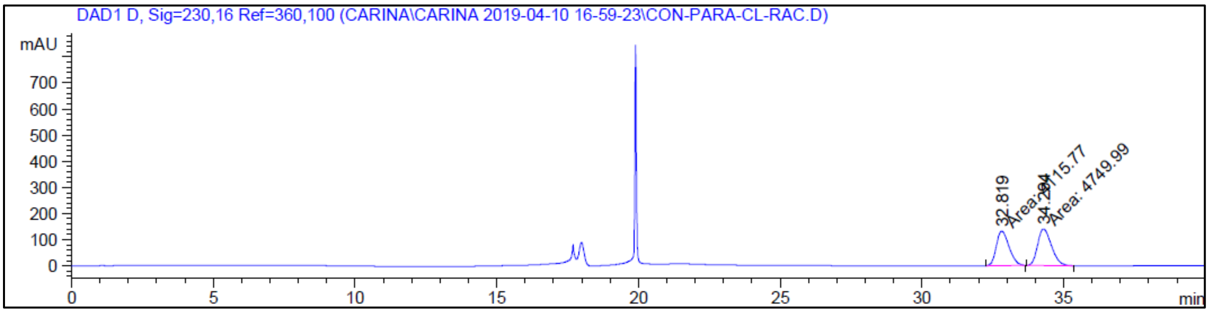

Peak #	RetTime [min]	Type	Width [min]	Area [mAU*s]	Height [mAU]	Area %
1	32.819	MM	0.5185	4115.76904	132.28470	46.4232
2	34.294	MM	0.5639	4749.98633	140.39131	53.5768

HPLC trace: Hydrogenation of the *E*-isomer: **(+)-21**

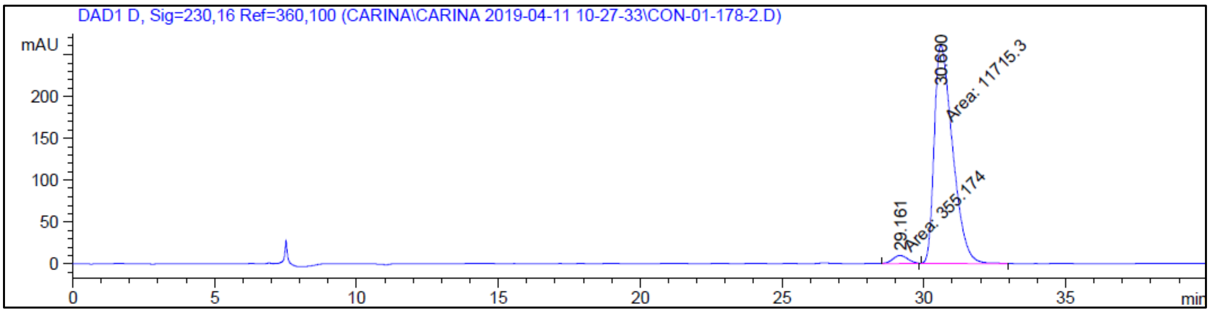

Peak #	RetTime [min]	Type	Width [min]	Area [mAU*s]	Height [mAU]	Area %
1	29.161	MM	0.6158	355.17386	9.61346	2.9425
2	30.600	MM	0.7501	1.17153e4	260.30472	97.0575

HPLC trace: Hydrogenation of the *Z*-isomer: **(-)-21**

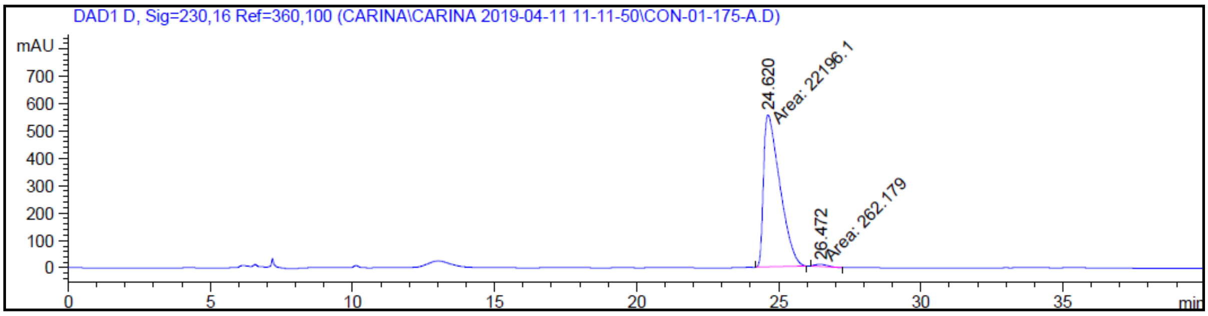

Peak #	RetTime [min]	Type	Width [min]	Area [mAU*s]	Height [mAU]	Area %
1	24.620	MM	0.6649	2.21961e4	556.35767	98.8326
2	26.472	MM	0.5397	262.17914	8.09707	1.1674

Diethyl (2-(4-bromophenyl)propyl)phosphonate (22):

HPLC trace: racemic sample: **22**

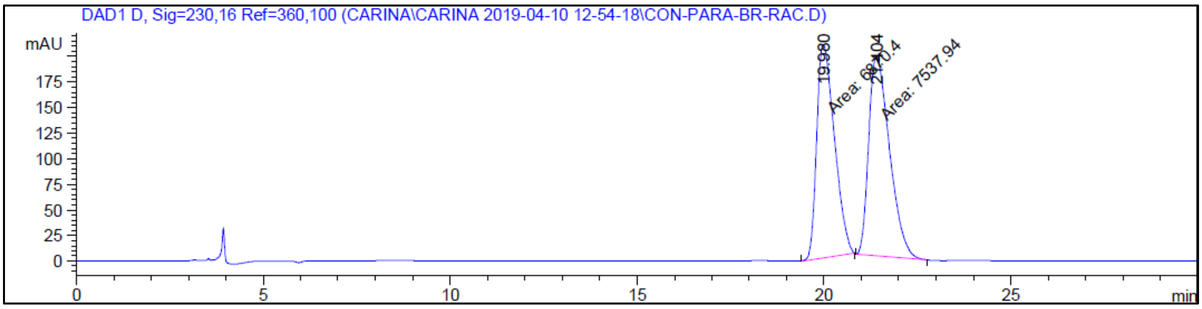

Peak #	RetTime [min]	Type	Width [min]	Area [mAU*s]	Height [mAU]	Area %
1	19.980	MM	0.5491	6870.39893	208.53989	47.6835
2	21.404	MM	0.6446	7537.93750	194.90073	52.3165

HPLC trace: Hydrogenation of the *E*-isomer: **(+)-22**

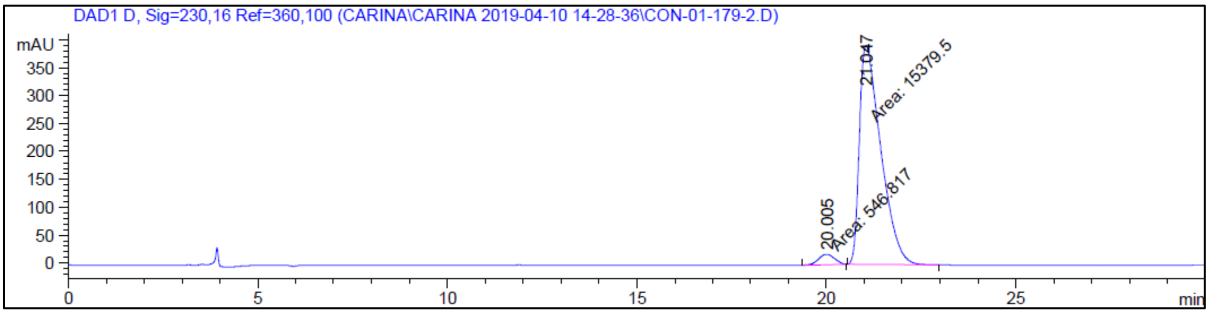

Peak #	RetTime [min]	Type	Width [min]	Area [mAU*s]	Height [mAU]	Area %
1	20.005	MM	0.4867	546.81683	18.72453	3.4334
2	21.047	MM	0.6535	1.53795e4	392.25497	96.5666

HPLC trace: Hydrogenation of the *Z*-isomer: **(-)-22**

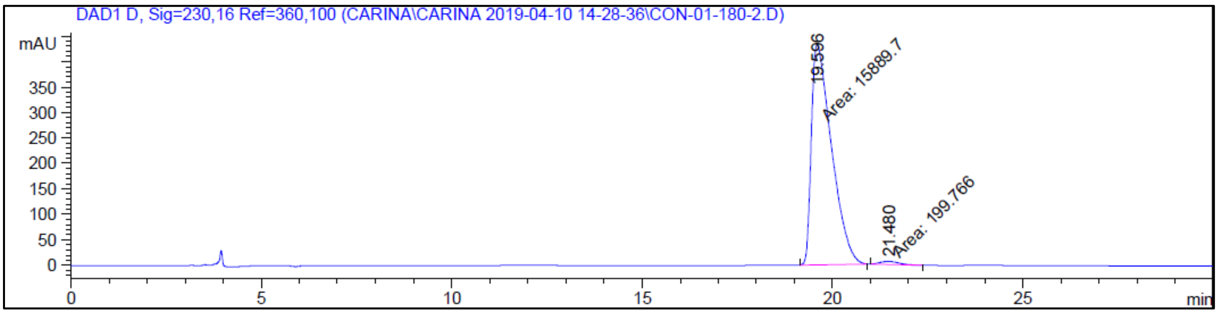

Peak #	RetTime [min]	Type	Width [min]	Area [mAU*s]	Height [mAU]	Area %
1	19.596	MM	0.6115	1.58897e4	433.09000	98.7584
2	21.480	MM	0.5679	199.76573	5.86244	1.2416

Diethyl (2-(4-(trifluoromethyl)phenyl)propyl)phosphonate (23):

HPLC trace: racemic sample: **23**

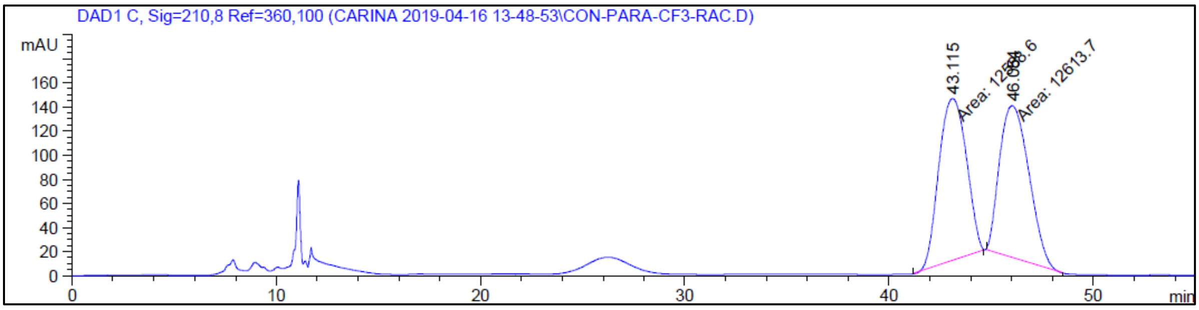

Peak #	RetTime [min]	Type	Width [min]	Area [mAU*s]	Height [mAU]	Area %
1	43.115	MM	1.5631	1.25886e4	134.22606	49.9502
2	46.054	MM	1.6703	1.26137e4	125.86597	50.0498

HPLC trace: Hydrogenation of the *E*-isomer: **(+)-23**

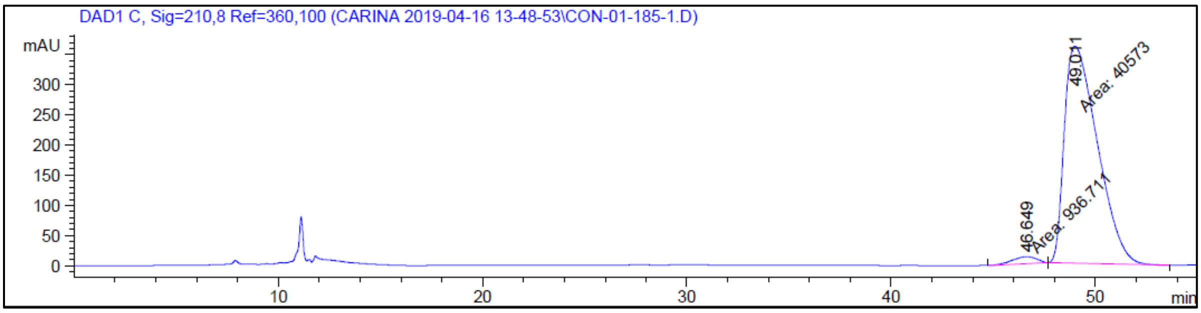

Peak #	RetTime [min]	Type	Width [min]	Area [mAU*s]	Height [mAU]	Area %
1	46.649	MM	1.4017	936.71118	11.13741	2.2566
2	49.011	MM	1.8774	4.05730e4	360.18695	97.7434

HPLC trace: Hydrogenation of the *Z*-isomer: **(-)-23**

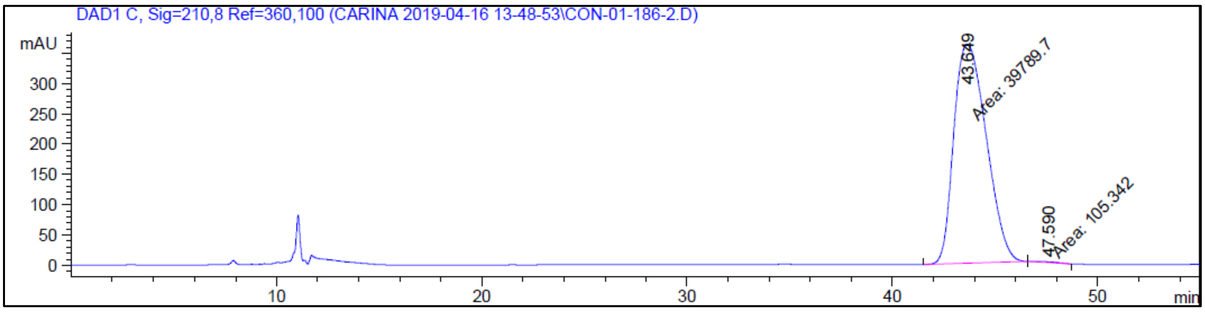

Peak #	RetTime [min]	Type	Width [min]	Area [mAU*s]	Height [mAU]	Area %
1	43.649	MM	1.8310	3.97897e4	362.17877	99.7360
2	47.590	MM T	1.1814	105.34184	1.48610	0.2640

Diethyl (2-(4-tolyl)propyl)phosphonate (24):

HPLC trace: racemic sample: **24**

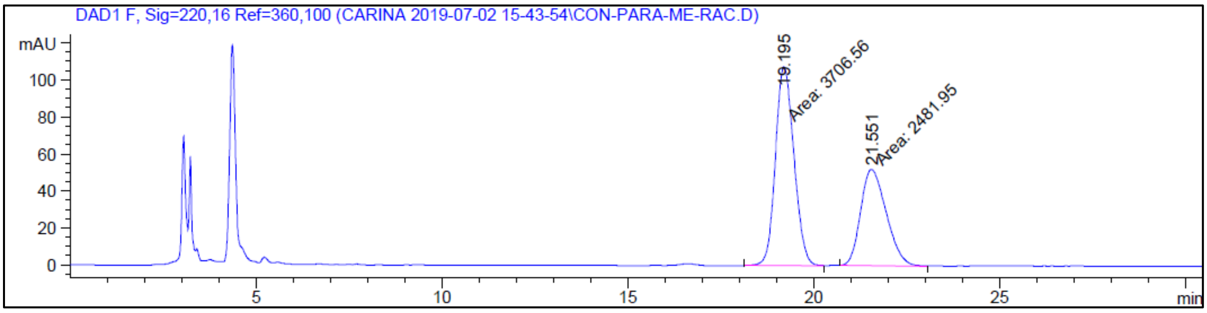

Peak #	RetTime [min]	Type	Width [min]	Area [mAU*s]	Height [mAU]	Area %
1	19.195	MM	0.5749	3706.55933	107.46366	59.8942
2	21.551	MM	0.7968	2481.94922	51.91702	40.1058

HPLC trace: Hydrogenation of the *E*-isomer: **(+)-24**

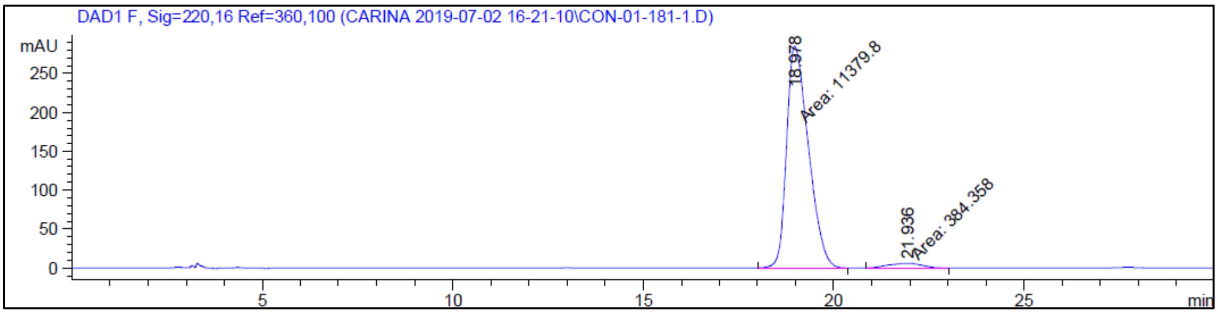

Peak #	RetTime [min]	Type	Width [min]	Area [mAU*s]	Height [mAU]	Area %
1	18.978	MM	0.6655	1.13798e4	285.00610	96.7328
2	21.936	MM	1.0384	384.35846	6.16890	3.2672

HPLC trace: Hydrogenation of the *Z*-isomer: **(-)-24**

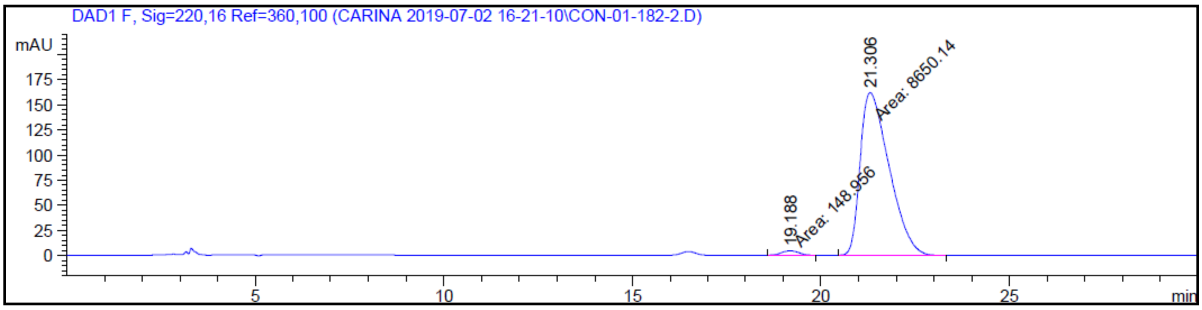

Peak #	RetTime [min]	Type	Width [min]	Area [mAU*s]	Height [mAU]	Area %
1	19.188	MM	0.5426	148.95581	4.57533	1.6929
2	21.306	MM	0.8916	8650.13574	161.70041	98.3071

Diethyl (2-(4-(*tert*-butyl)phenyl)propyl)phosphonate (25):

HPLC trace: racemic sample: **25**

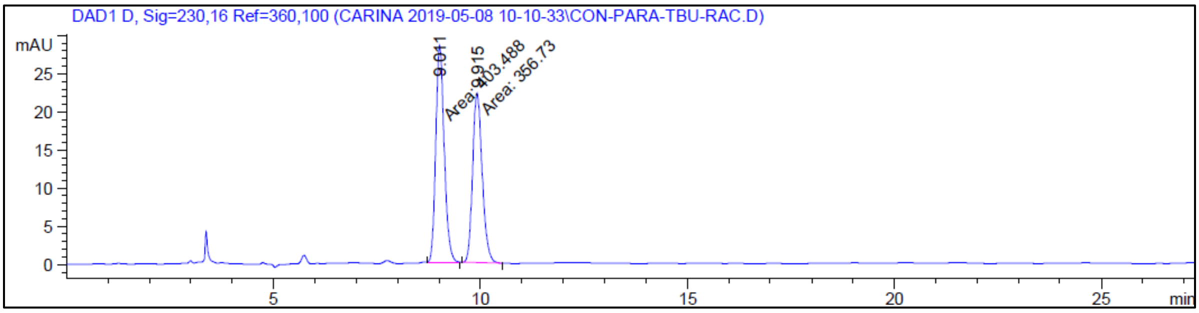

Peak #	RetTime [min]	Type	Width [min]	Area [mAU*s]	Height [mAU]	Area %
1	9.011	MM	0.2368	403.48837	28.39643	53.0753
2	9.915	MM	0.2674	356.73038	22.23635	46.9247

HPLC trace: Hydrogenation of the *E*-isomer: **(+)-25**

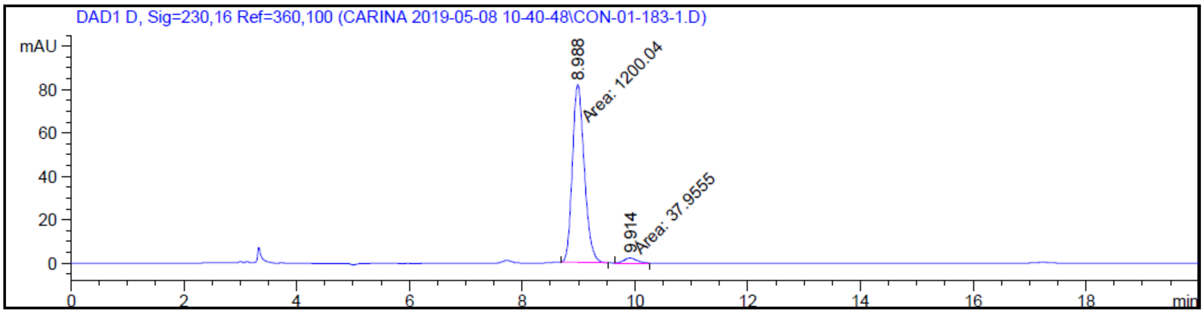

Peak #	RetTime [min]	Type	Width [min]	Area [mAU*s]	Height [mAU]	Area %
1	8.988	MM	0.2443	1200.03809	81.88293	96.9341
2	9.914	MM	0.2735	37.95551	2.31282	3.0659

HPLC trace: Hydrogenation of the *Z*-isomer: **(-)-25**

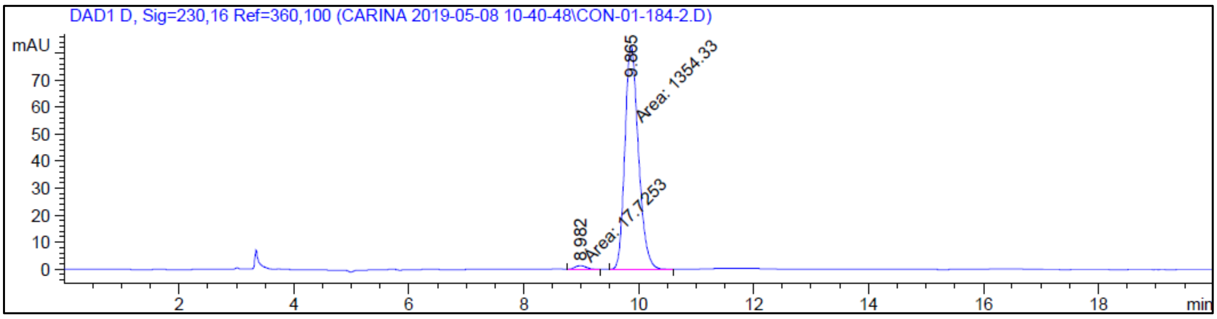

Peak #	RetTime [min]	Type	Width [min]	Area [mAU*s]	Height [mAU]	Area %
1	8.982	MM	0.2215	17.72525	1.33403	1.2919
2	9.865	MM	0.2725	1354.33411	82.84174	98.7081

Diethyl (2-(3-bromophenyl)propyl)phosphonate (26):

HPLC trace: racemic sample: **26**

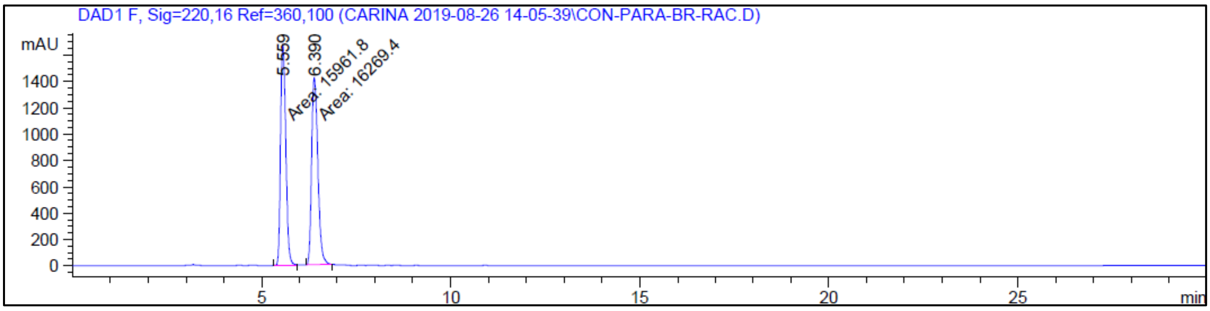

Peak #	RetTime [min]	Type	Width [min]	Area [mAU*s]	Height [mAU]	Area %
1	5.559	MM	0.1588	1.59618e4	1675.68738	49.5228
2	6.390	MM	0.1909	1.62694e4	1420.50696	50.4772

HPLC trace: Hydrogenation of the *E*-isomer: **(+)-26**

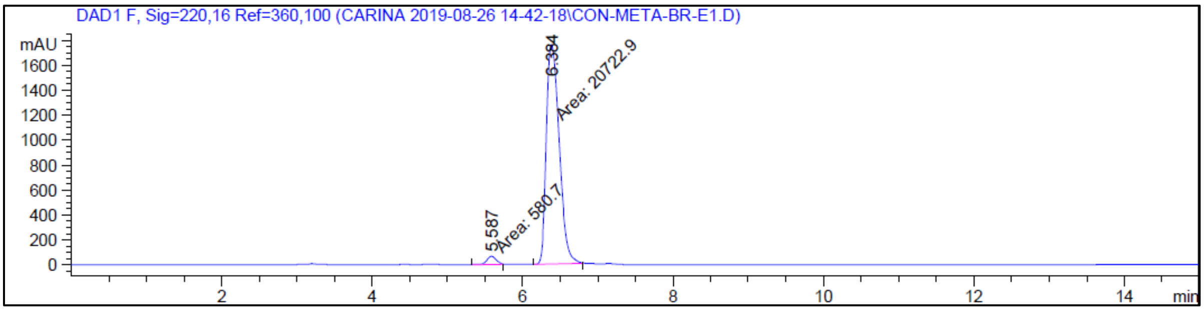

Peak #	RetTime [min]	Type	Width [min]	Area [mAU*s]	Height [mAU]	Area %
1	5.587	MM	0.1465	580.70013	66.05540	2.7258
2	6.384	MM	0.1964	2.07229e4	1758.99243	97.2742

HPLC trace: Hydrogenation of the *Z*-isomer: **(-)-26**

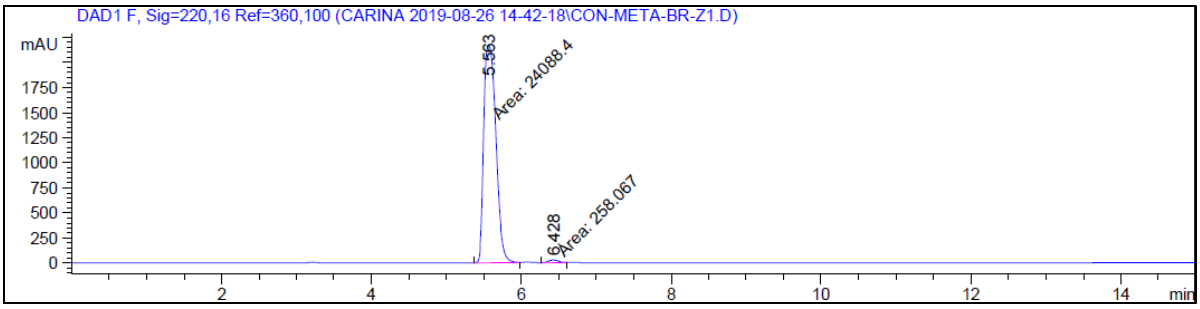

Peak #	RetTime [min]	Type	Width [min]	Area [mAU*s]	Height [mAU]	Area %
1	5.563	MM	0.1847	2.40884e4	2173.72412	98.9400
2	6.428	MM	0.1586	258.06717	27.11945	1.0600

Dimethyl (2-phenylpropyl)phosphonate (27):

HPLC trace: racemic sample: **27**

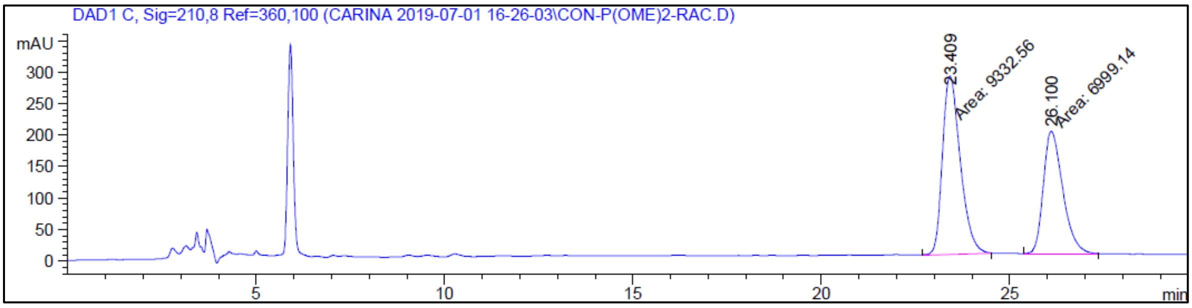

Peak #	RetTime [min]	Type	Width [min]	Area [mAU*s]	Height [mAU]	Area %
1	23.409	MM	0.5490	9332.56055	283.32281	57.1438
2	26.100	MM	0.5994	6999.14160	194.61896	42.8562

HPLC trace: Hydrogenation of the *E*-isomer: **(+)-27**

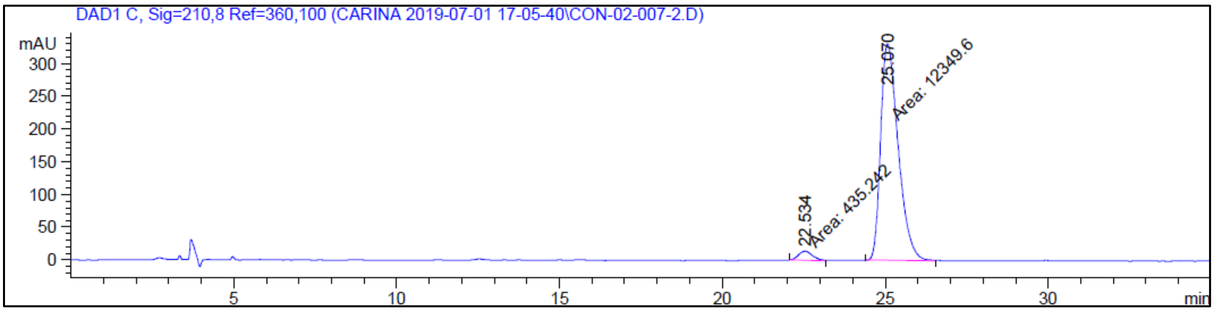

Peak #	RetTime [min]	Type	Width [min]	Area [mAU*s]	Height [mAU]	Area %
1	22.534	MM	0.5120	435.24225	14.16699	3.4044
2	25.070	MM	0.6234	1.23496e4	330.14307	96.5956

HPLC trace: Hydrogenation of the *Z*-isomer: **(-)-27**

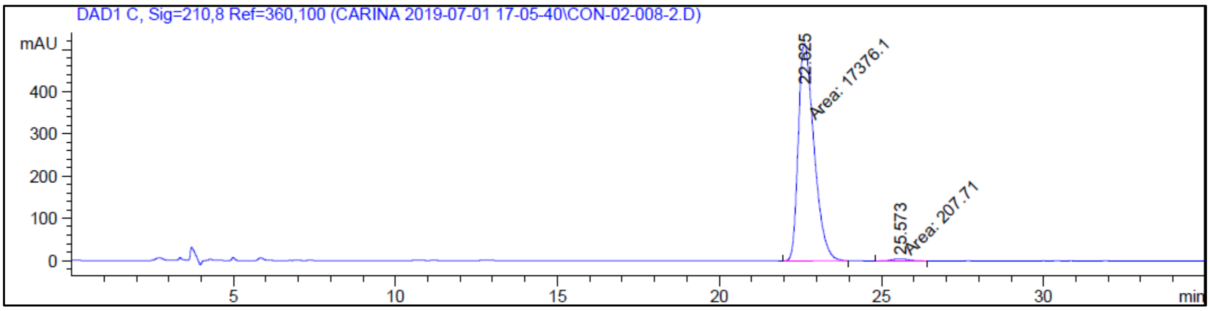

Peak #	RetTime [min]	Type	Width [min]	Area [mAU*s]	Height [mAU]	Area %
1	22.625	MM	0.5630	1.73761e4	514.36853	98.8187
2	25.573	MM	0.6572	207.70972	5.26756	1.1813

Diethyl (2-(4-methoxyphenyl)propyl)phosphonate (28):

HPLC trace: racemic sample: **28**

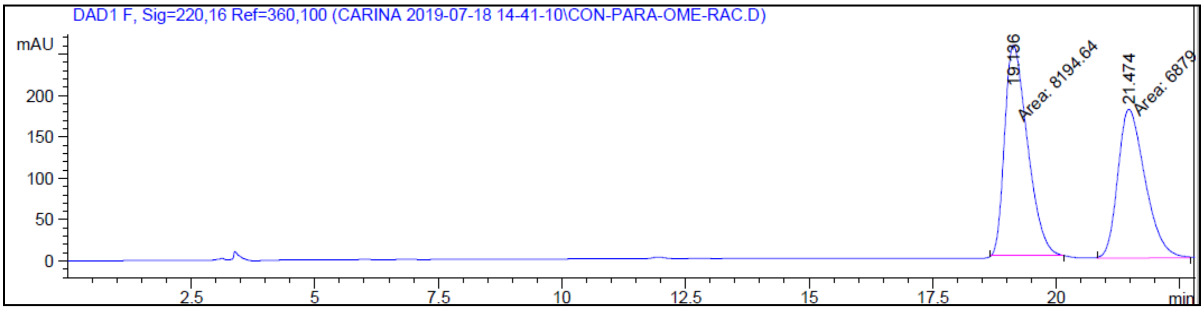

Peak #	RetTime [min]	Type	Width [min]	Area [mAU*s]	Height [mAU]	Area %
1	19.136	MM	0.5369	8194.63672	254.40019	54.3637
2	21.474	MM	0.6387	6879.10010	179.50084	45.6363

HPLC trace: Hydrogenation of the *E*-isomer: **(+)-28**

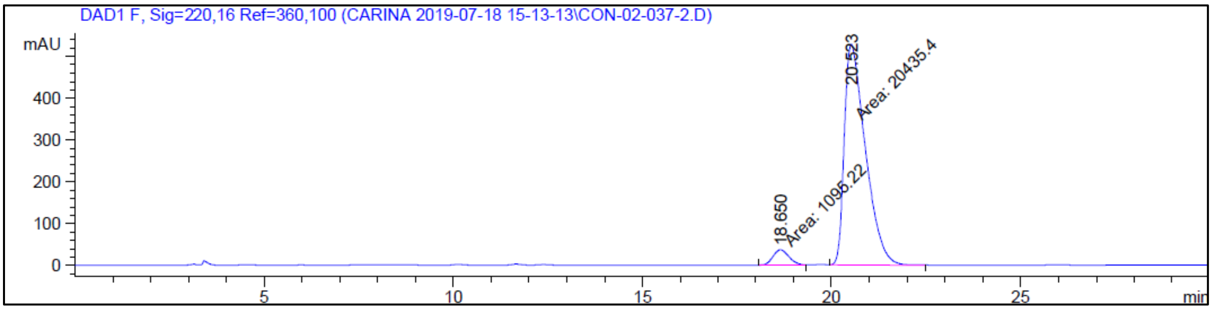

Peak #	RetTime [min]	Type	Width [min]	Area [mAU*s]	Height [mAU]	Area %
1	18.650	MM	0.4943	1095.21692	36.92450	5.0868
2	20.523	MM	0.6451	2.04354e4	527.94647	94.9132

HPLC trace: Hydrogenation of the *Z*-isomer: **(-)-28**

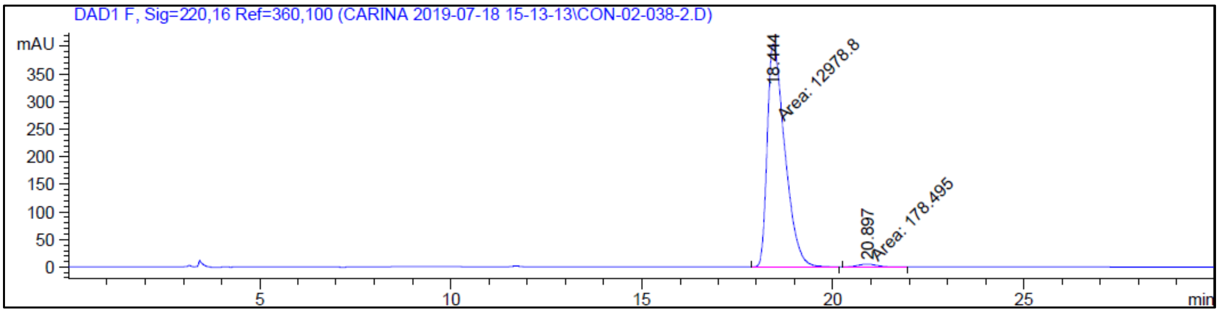

Peak #	RetTime [min]	Type	Width [min]	Area [mAU*s]	Height [mAU]	Area %
1	18.444	MM	0.5352	1.29788e4	404.17386	98.6434
2	20.897	MM	0.5890	178.49486	5.05070	1.3566

Diethyl (2-(naphthalen-2-yl)propyl)phosphonate (29):

HPLC trace: racemic sample: **29**

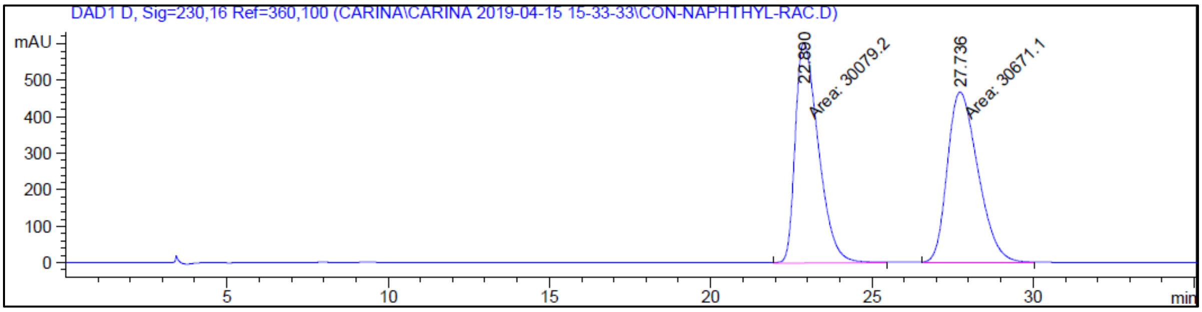

Peak #	RetTime [min]	Type	Width [min]	Area [mAU*s]	Height [mAU]	Area %
1	22.890	MM	0.8326	3.00792e4	602.14160	49.5128
2	27.736	MM	1.0973	3.06711e4	465.83661	50.4872

HPLC trace: Hydrogenation of the *E*-isomer: **(+)-29**

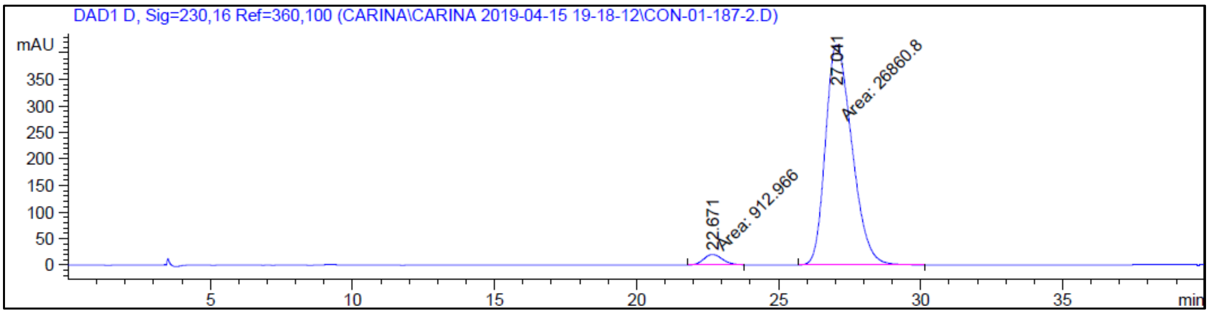

Peak #	RetTime [min]	Type	Width [min]	Area [mAU*s]	Height [mAU]	Area %
1	22.671	MM	0.7617	912.96643	19.97685	3.2872
2	27.041	MM	1.0798	2.68608e4	414.59750	96.7128

HPLC trace: Hydrogenation of the *Z*-isomer: **(-)-29**

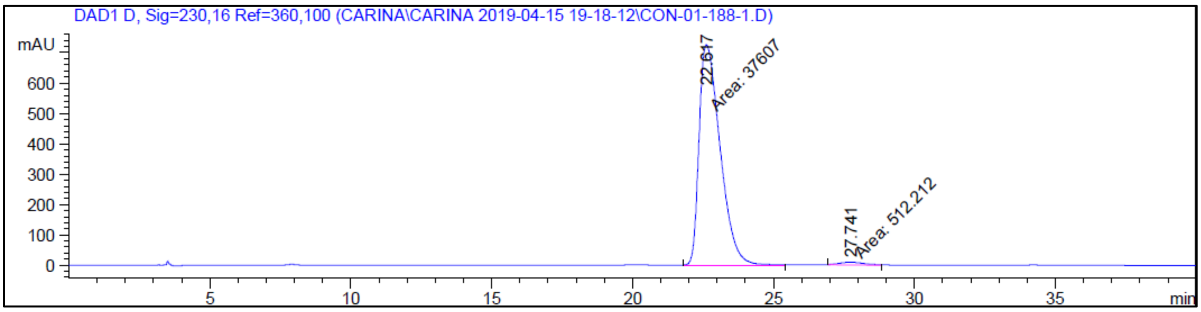

Peak #	RetTime [min]	Type	Width [min]	Area [mAU*s]	Height [mAU]	Area %
1	22.617	MM	0.8634	3.76070e4	725.96863	98.6563
2	27.741	MM	1.0034	512.21191	8.50805	1.3437

Diethyl (2-phenylbutyl)phosphonate (30):

HPLC trace: racemic sample: **30**

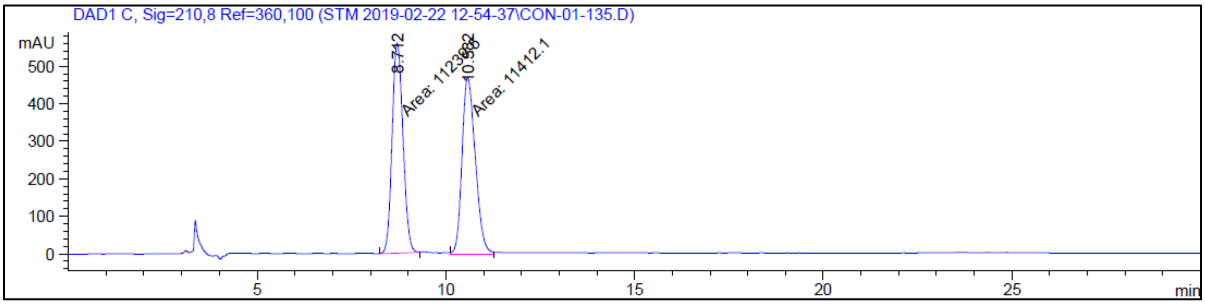

Peak #	RetTime [min]	Type	Width [min]	Area [mAU*s]	Height [mAU]	Area %
1	8.712	MM	0.3349	1.12395e4	559.31256	49.6190
2	10.582	MM	0.4048	1.14121e4	469.89471	50.3810

HPLC trace: Hydrogenation of the *E*-isomer: **(+)-30**

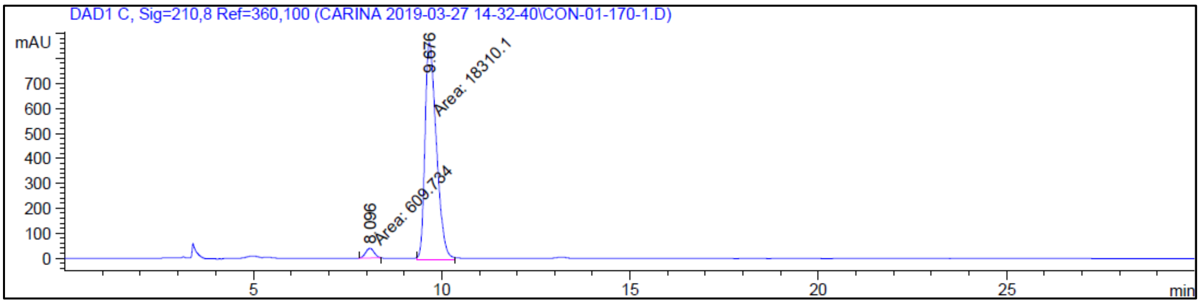

Peak #	RetTime [min]	Type	Width [min]	Area [mAU*s]	Height [mAU]	Area %
1	8.096	MM	0.2614	609.73364	38.87682	3.2227
2	9.676	MM	0.3516	1.83101e4	867.93365	96.7773

HPLC trace: Hydrogenation of the *Z*-isomer: **(-)-30**

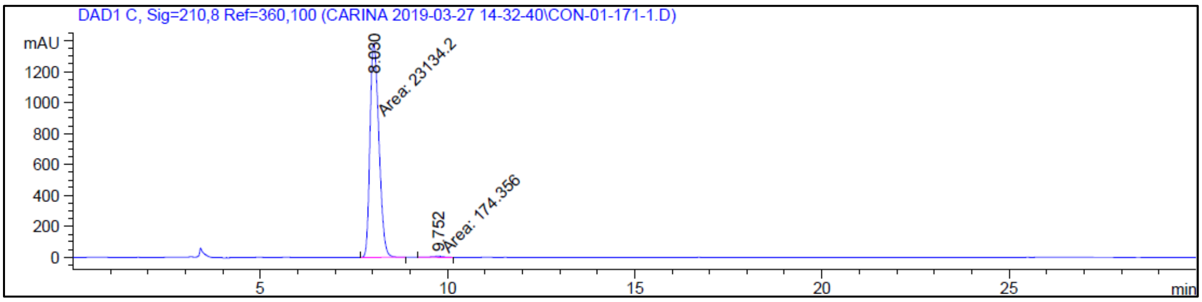

Peak #	RetTime [min]	Type	Width [min]	Area [mAU*s]	Height [mAU]	Area %
1	8.030	MM	0.2790	2.31342e4	1382.21948	99.2520
2	9.752	MM	0.4057	174.35568	7.16207	0.7480

Diethyl (2-(2-fluorophenyl)propyl)phosphonate (31):

HPLC trace: racemic sample: **31**

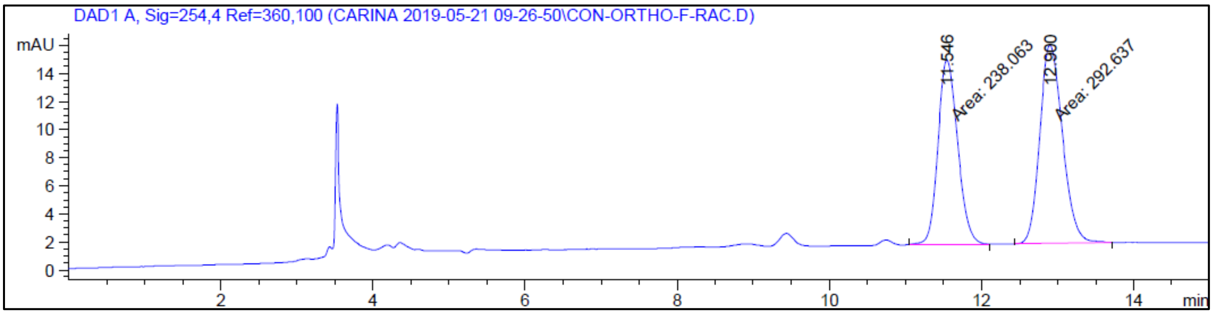

Peak #	RetTime [min]	Type	Width [min]	Area [mAU*s]	Height [mAU]	Area %
1	11.546	MM	0.3015	238.06270	13.15818	44.8583
2	12.900	MM	0.3454	292.63675	14.12004	55.1417

HPLC trace: Hydrogenation of the *E*-isomer: **(+)-31**

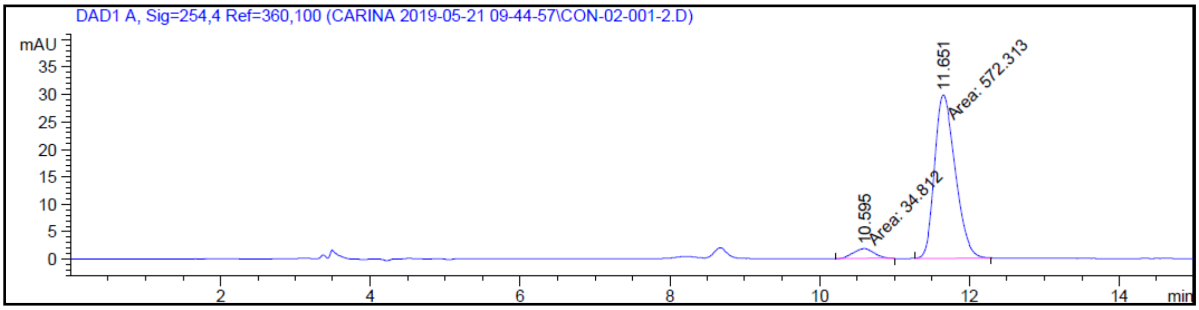

Peak #	RetTime [min]	Type	Width [min]	Area [mAU*s]	Height [mAU]	Area %
1	10.595	MM	0.3269	36.01491	1.83638	5.9203
2	11.651	MM	0.3206	572.31348	29.75652	94.0797

HPLC trace: Hydrogenation of the *Z*-isomer: **(-)-31**

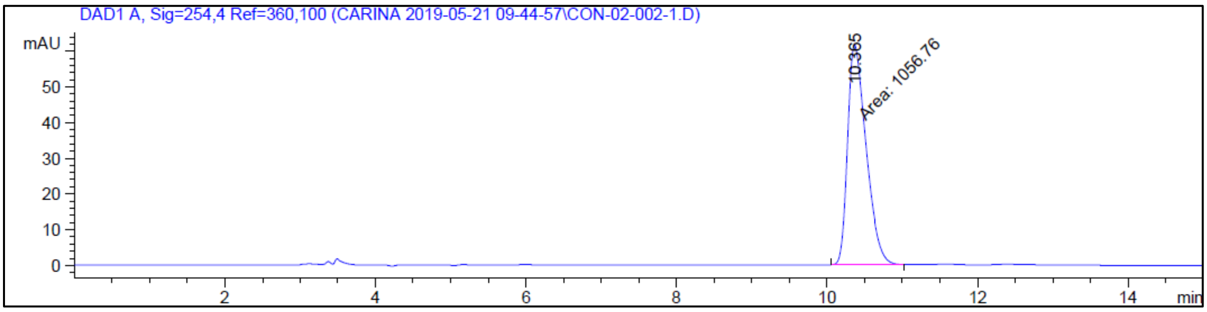

Peak #	RetTime [min]	Type	Width [min]	Area [mAU*s]	Height [mAU]	Area %
1	10.365	MM	0.2849	1056.76050	61.82899	100.0000

Diethyl (2-(2-tolyl)propyl)phosphonate (32):

HPLC trace: racemic sample: **32**

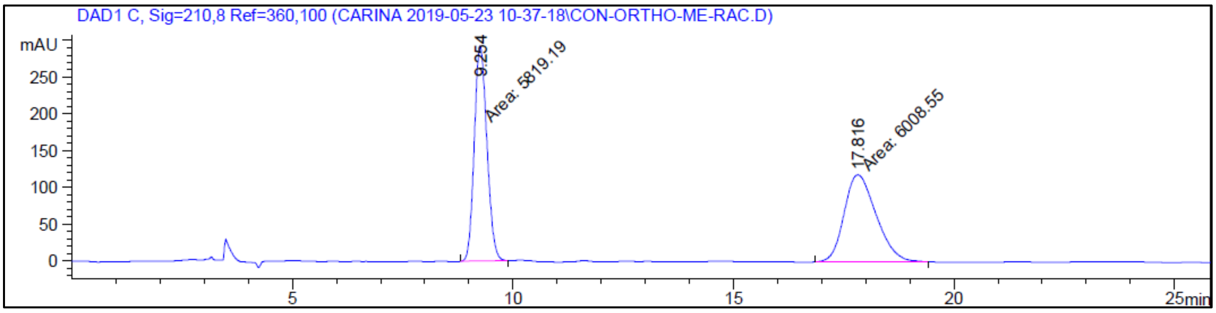

Peak #	RetTime [min]	Type	Width [min]	Area [mAU*s]	Height [mAU]	Area %
1	9.254	MM	0.3315	5819.18945	292.60989	49.1995
2	17.816	MM	0.8439	6008.54932	118.66042	50.8005

HPLC trace: Hydrogenation of the *E*-isomer: **(+)-32**

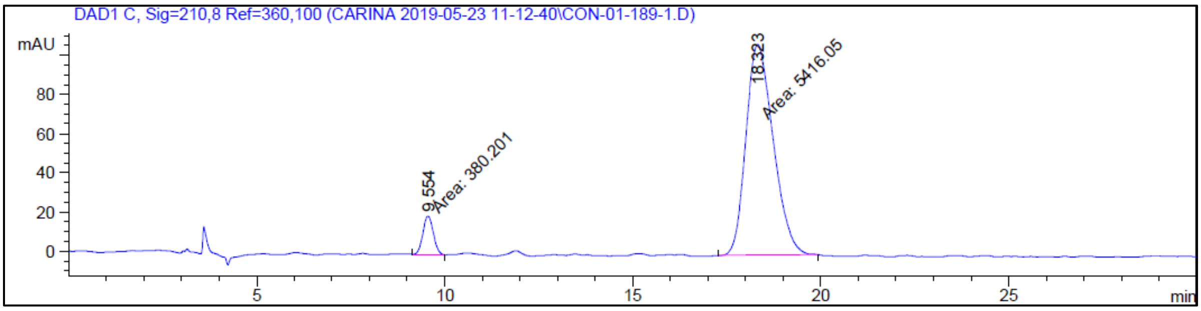

Peak #	RetTime [min]	Type	Width [min]	Area [mAU*s]	Height [mAU]	Area %
1	9.554	MM	0.3223	380.20148	19.66327	6.5594
2	18.323	MM	0.8402	5416.04590	107.43330	93.4406

HPLC trace: Hydrogenation of the *Z*-isomer: **(-)-32**

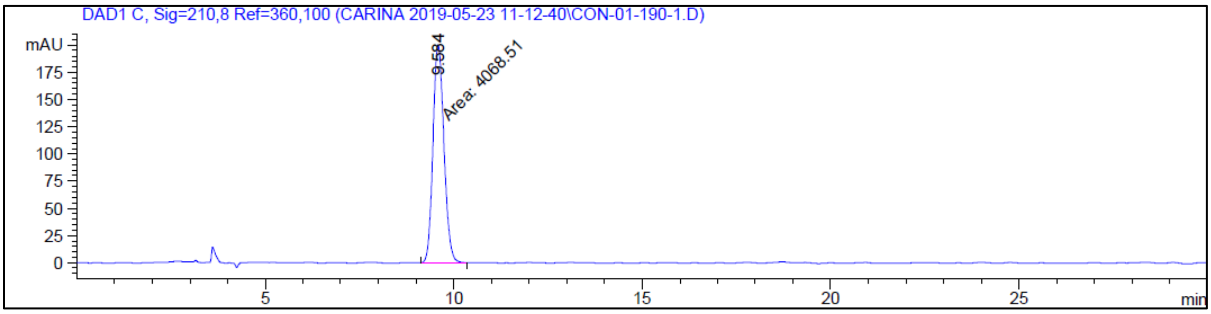

Peak #	RetTime [min]	Type	Width [min]	Area [mAU*s]	Height [mAU]	Area %
1	9.584	MM	0.3381	4068.51099	200.53094	100.0000

Diethyl (2-(2-chlorophenyl)propyl)phosphonate (33):

HPLC trace: racemic sample: **33**

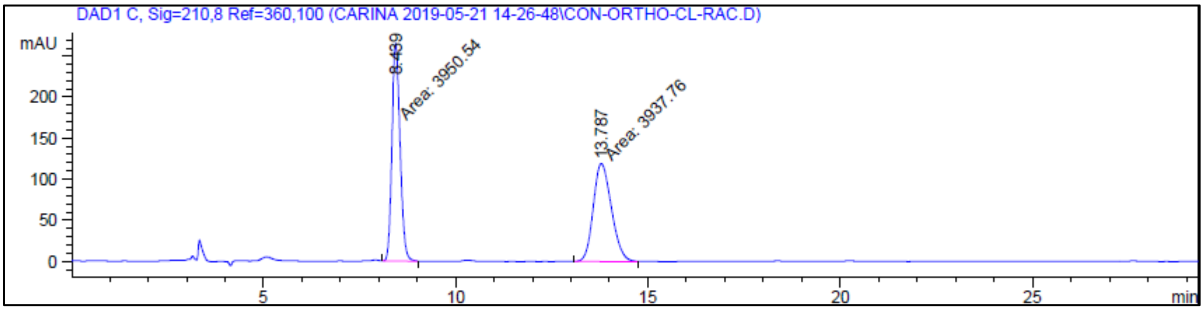

Peak #	RetTime [min]	Type	Width [min]	Area [mAU*s]	Height [mAU]	Area %
1	8.439	MM	0.2491	3950.54126	264.32837	50.0810
2	13.787	MM	0.5487	3937.76099	119.61113	49.9190

HPLC trace: Hydrogenation of the *E*-isomer: **(+)-33**

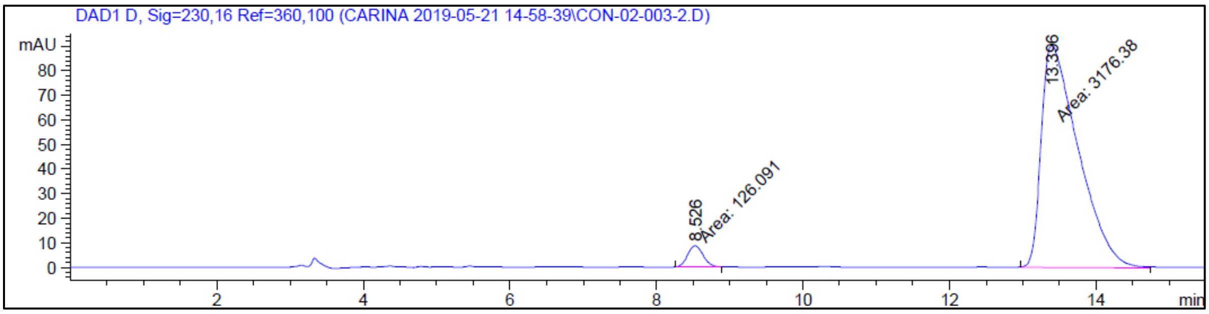

Peak #	RetTime [min]	Type	Width [min]	Area [mAU*s]	Height [mAU]	Area %
1	8.526	MM	0.2438	126.09103	8.61867	3.8181
2	13.396	MM	0.5858	3176.38135	90.37035	96.1819

HPLC trace: Hydrogenation of the *Z*-isomer: **(-)-33**

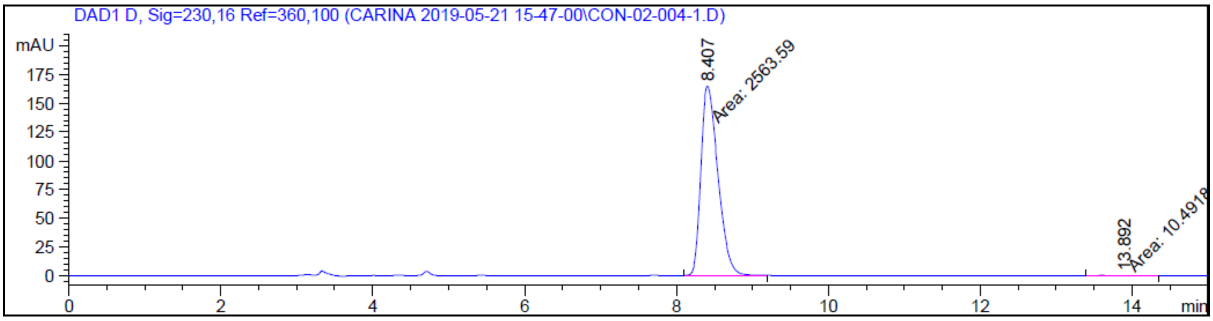

Peak #	RetTime [min]	Type	Width [min]	Area [mAU*s]	Height [mAU]	Area %
1	8.407	MM	0.2599	2563.59082	164.41252	99.5924
2	13.892	MM	0.5108	10.49185	3.42362e-1	0.4076

Diethyl (2-(2-bromophenyl)propyl)phosphonate (34):

HPLC trace: racemic sample: **34**

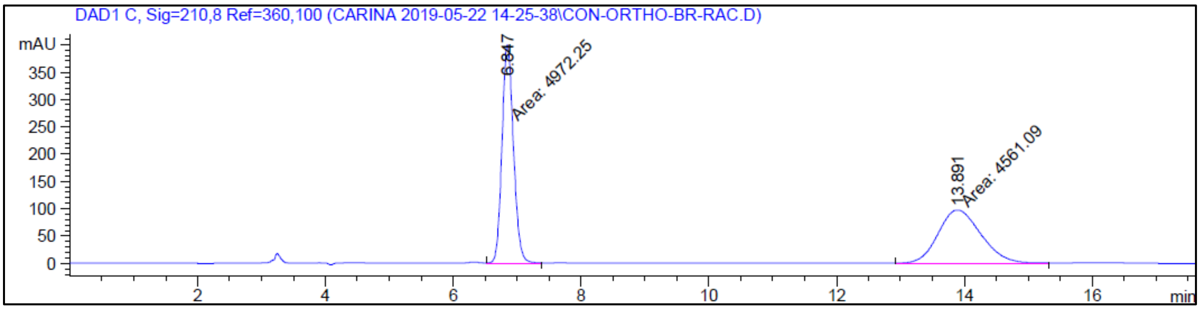

Peak #	RetTime [min]	Type	Width [min]	Area [mAU*s]	Height [mAU]	Area %
1	6.847	MM	0.2076	4972.24951	399.10309	52.1564
2	13.891	MM	0.7793	4561.09180	97.54132	47.8436

HPLC trace: Hydrogenation of the *E*-isomer: **(-)-34**

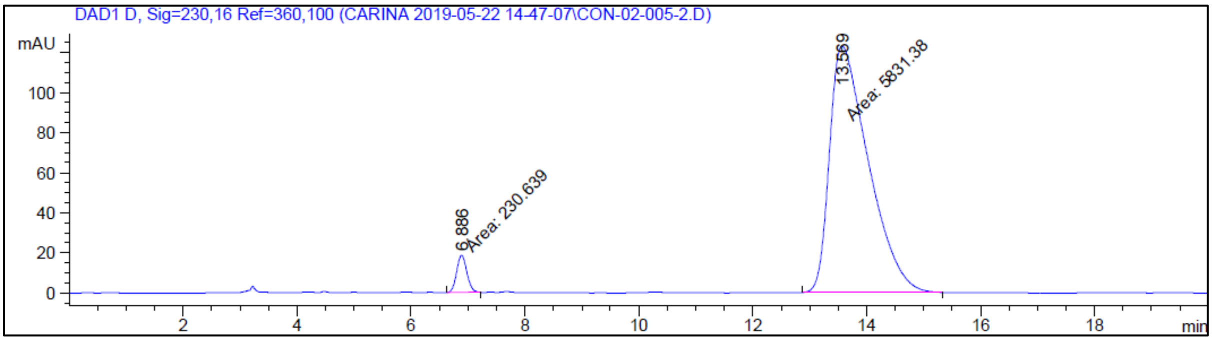

Peak #	RetTime [min]	Type	Width [min]	Area [mAU*s]	Height [mAU]	Area %
1	6.886	MM	0.2069	230.63921	18.58106	3.8047
2	13.569	MM	0.7902	5831.38379	122.98861	96.1953

HPLC trace: Hydrogenation of the *Z*-isomer: **(+)-34**

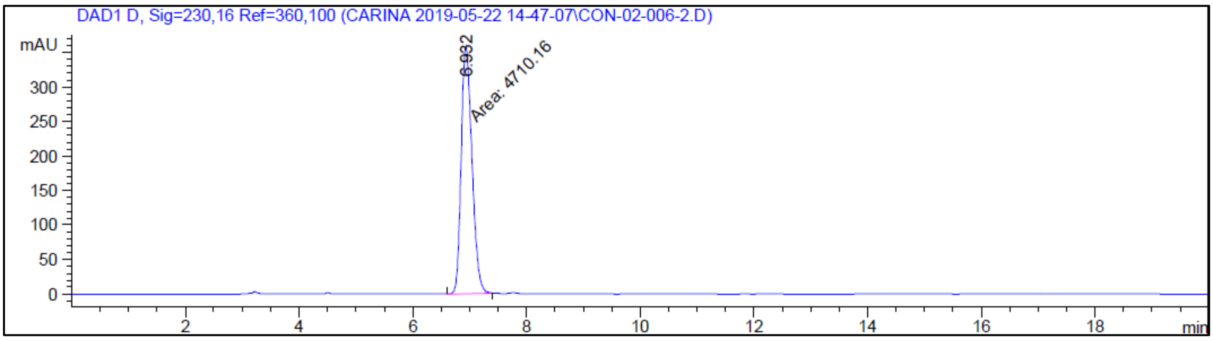

Peak #	RetTime [min]	Type	Width [min]	Area [mAU*s]	Height [mAU]	Area %
1	6.932	MM	0.2196	4710.16406	357.48602	100.0000

NMR Spectra of Key Compouds

^{1}H NMR (600 MHz, $CDCl_3$): ***E*-1**

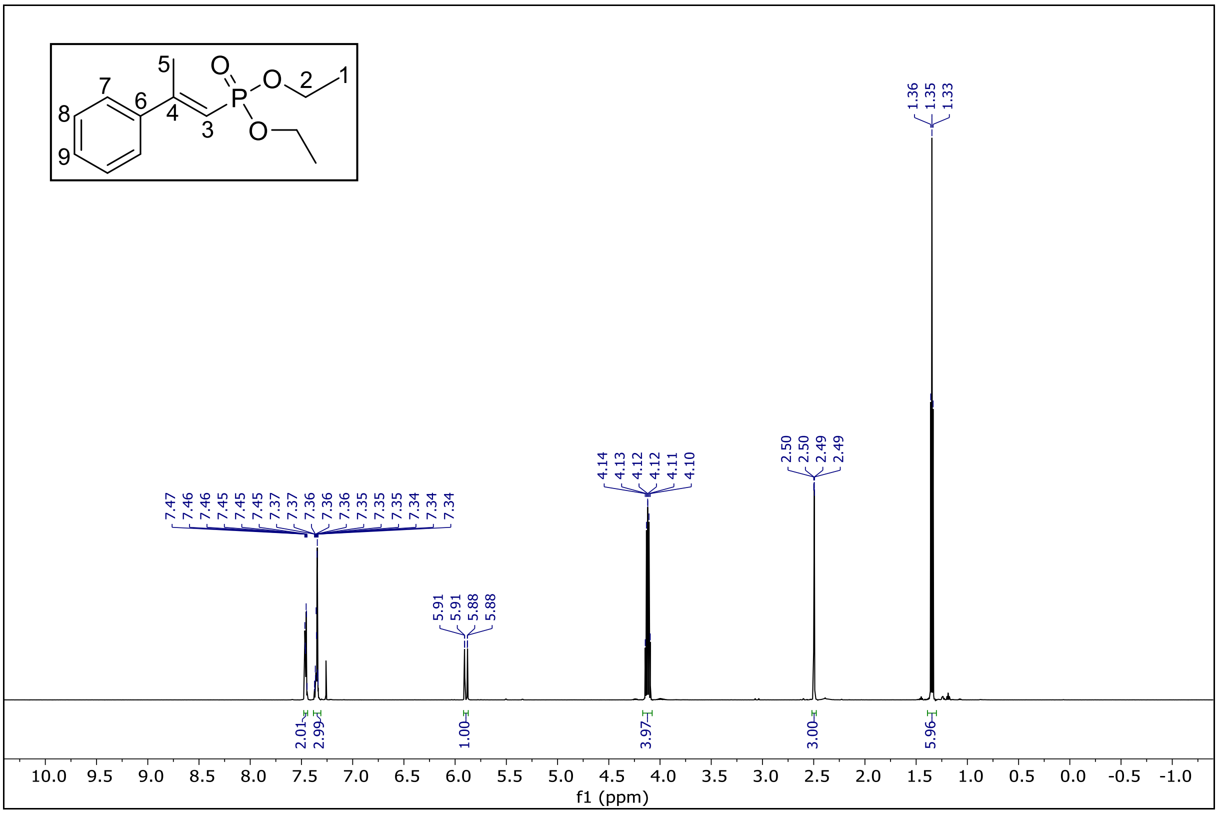

^{13}C NMR (151 MHz, $CDCl_3$): ***E*-1**

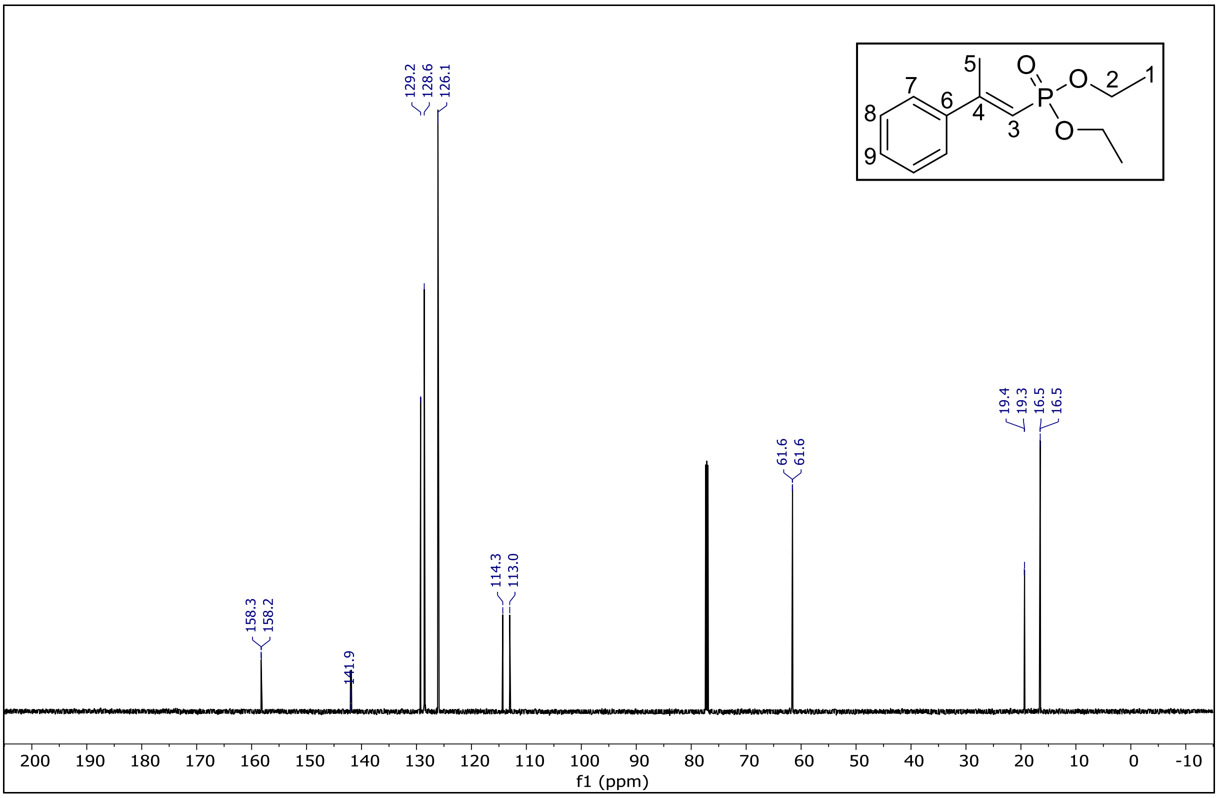

^{31}P NMR (162 MHz, $CDCl_3$): ***E*-1**

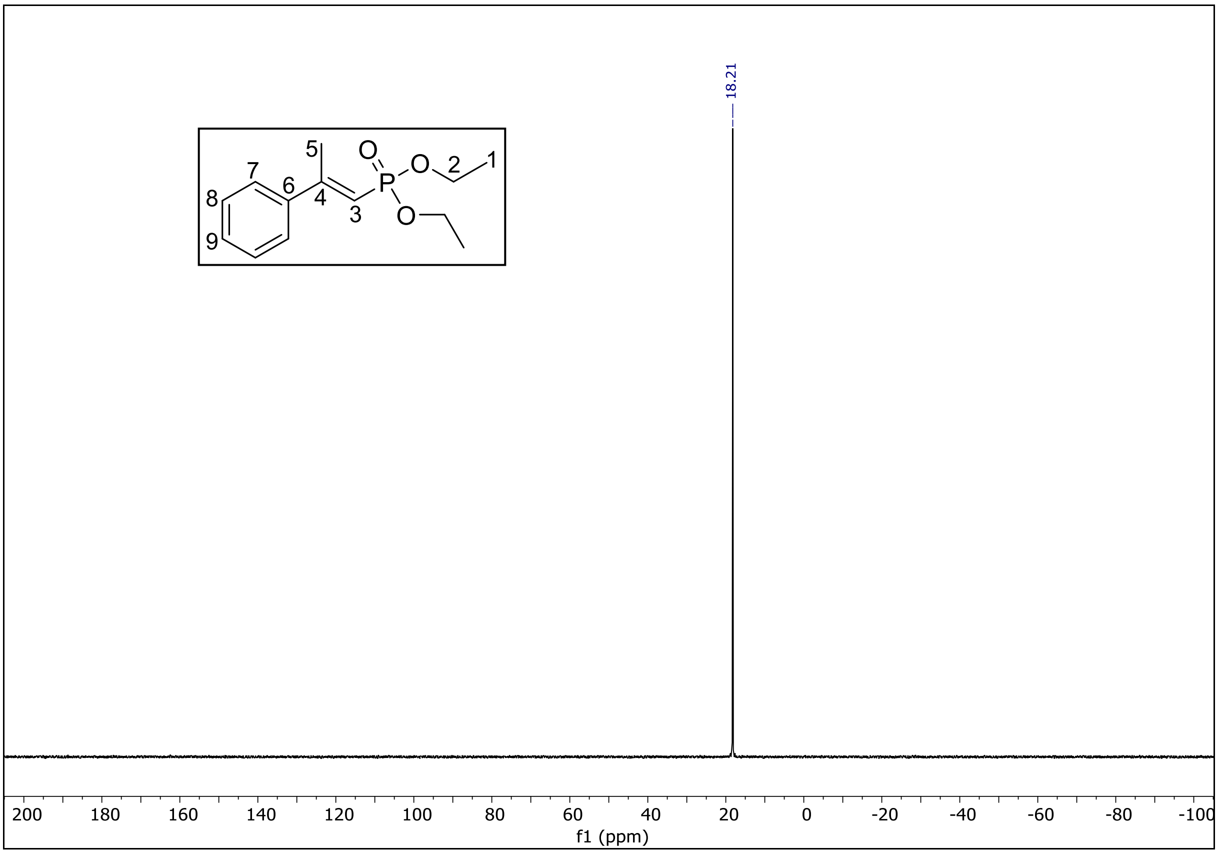

^{1}H NMR (500 MHz, $CDCl_3$): ***E*-2**

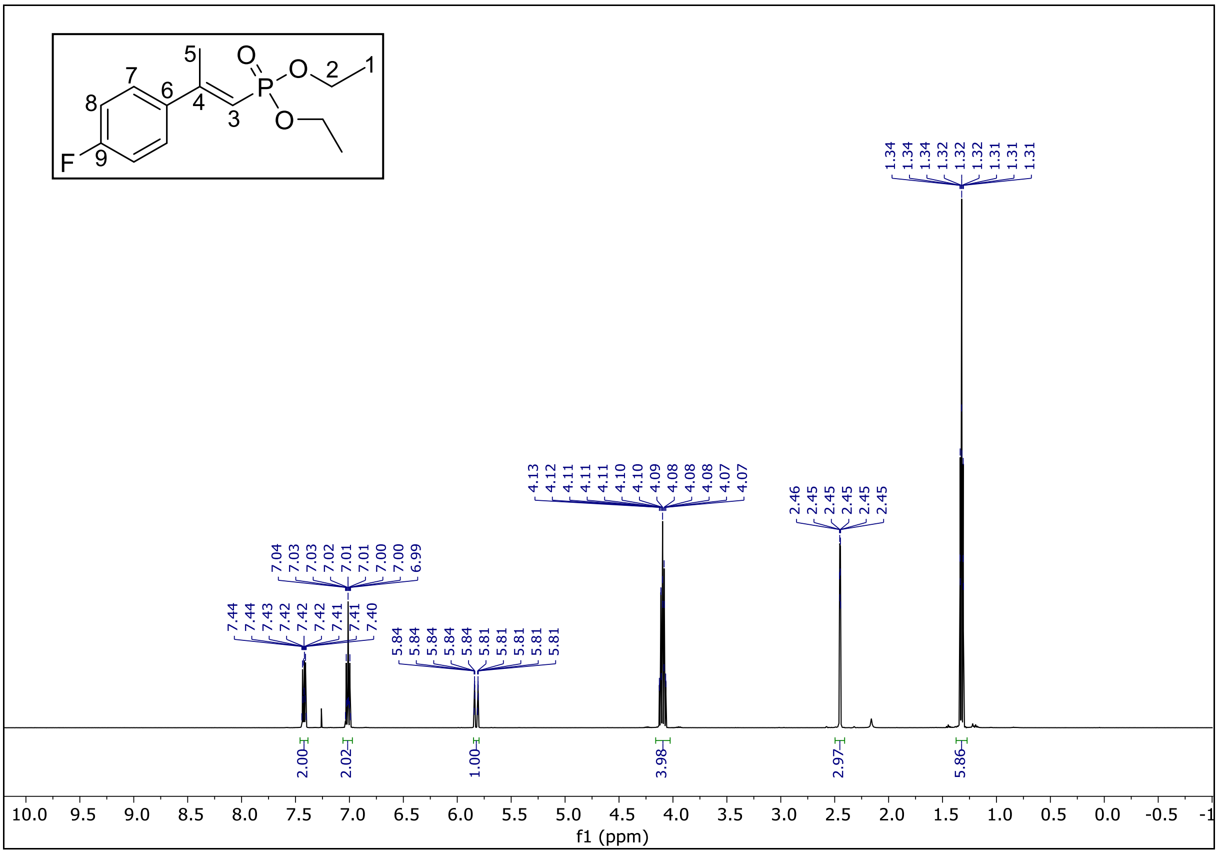

^{13}C NMR (126 MHz, $CDCl_3$): ***E*-2**

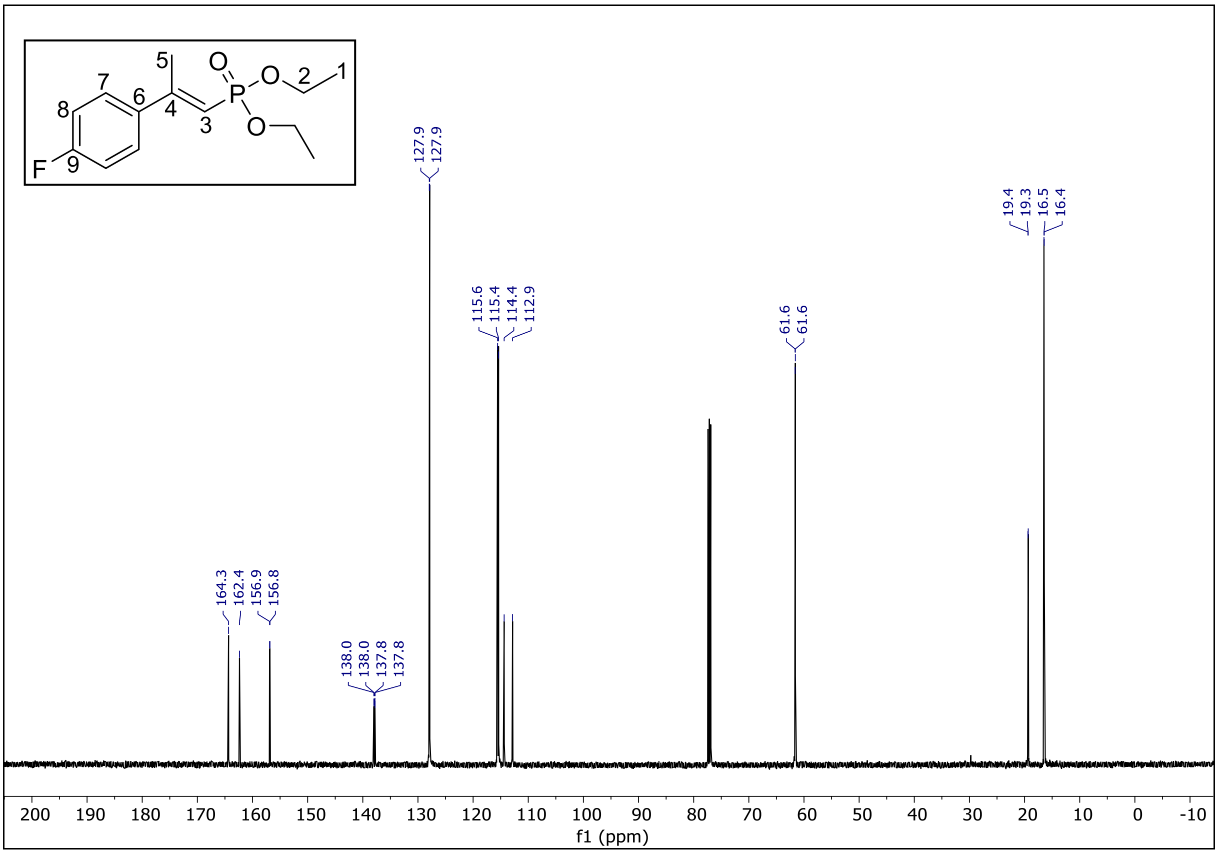

^{19}F NMR (470 MHz, $CDCl_3$): ***E*-2**

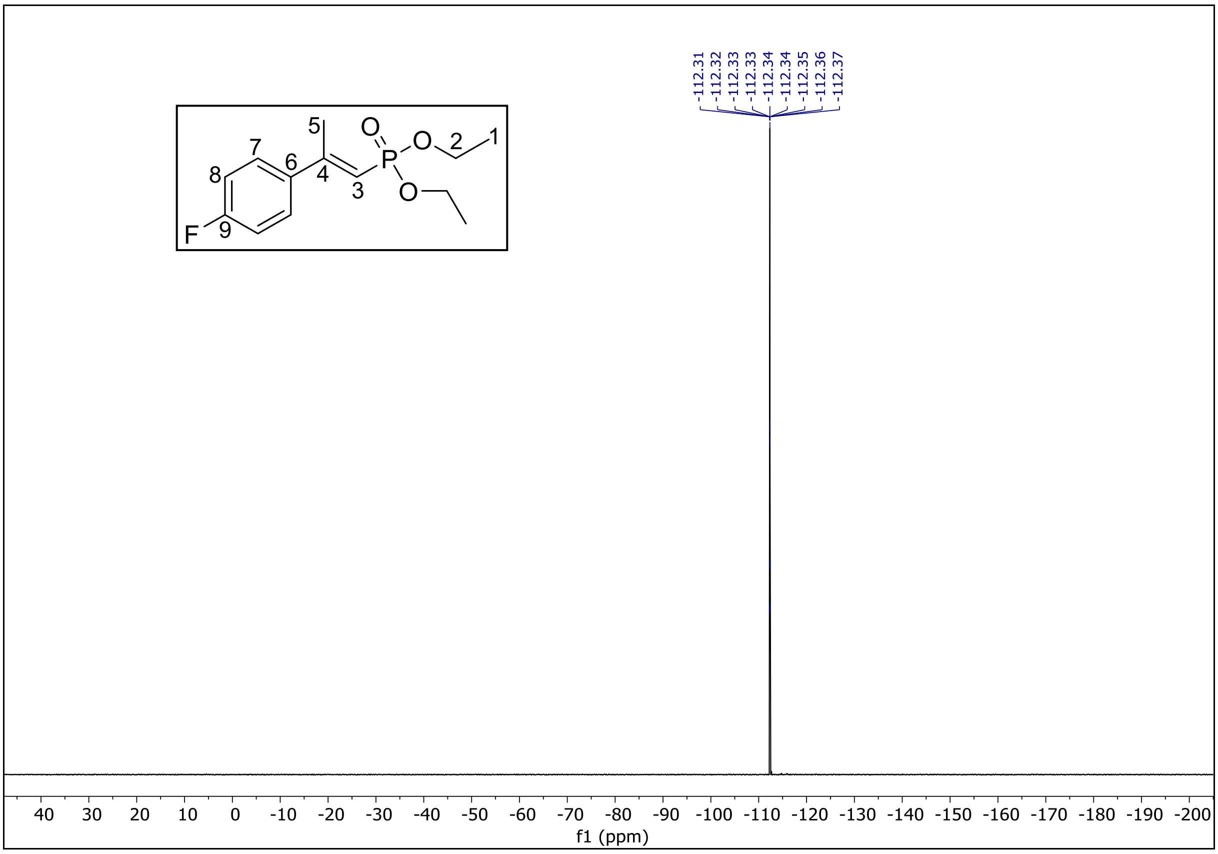

^{31}P NMR (202 MHz, CDCl$_3$): ***E*-2**

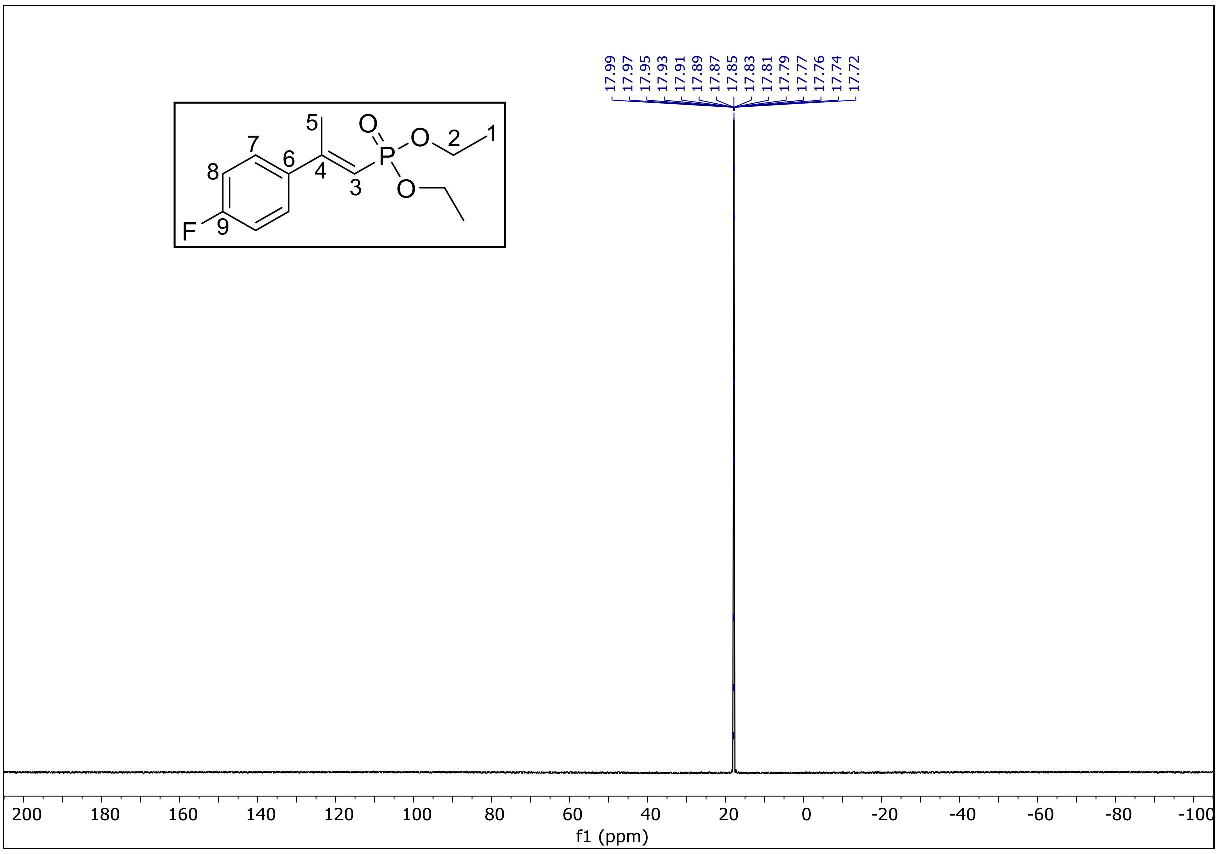

^{1}H NMR (600 MHz, CDCl$_3$): ***E*-3**

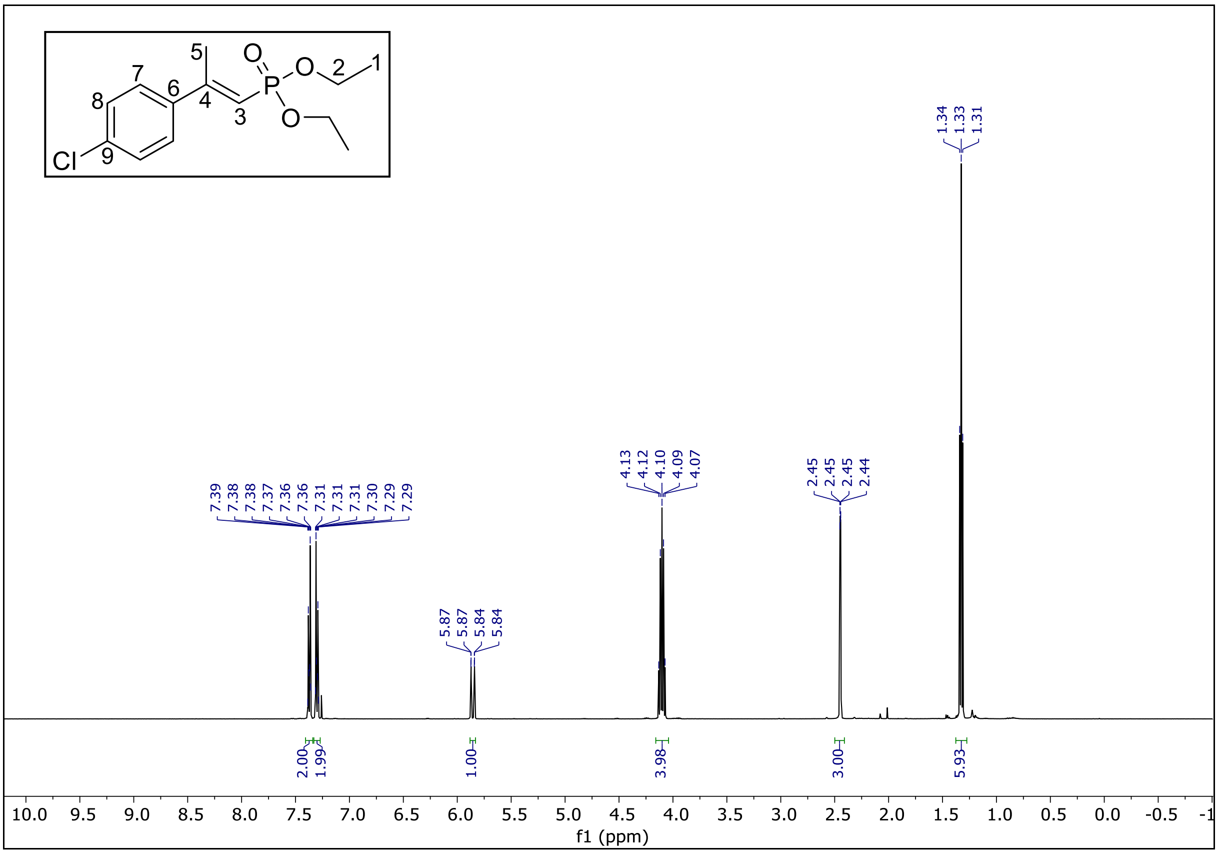

^{13}C NMR (126 MHz, CDCl$_3$): ***E*-3**

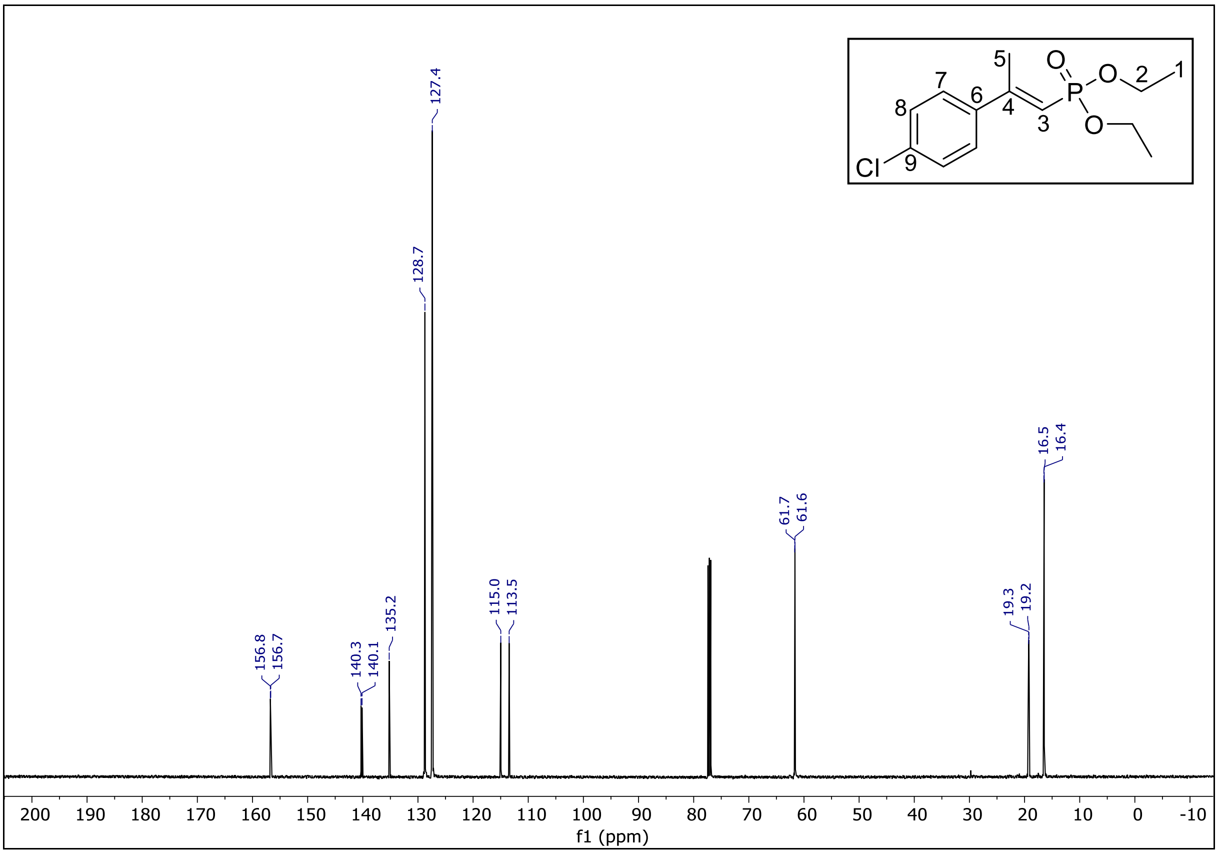

^{31}P NMR (202 MHz, CDCl$_3$): ***E*-3**

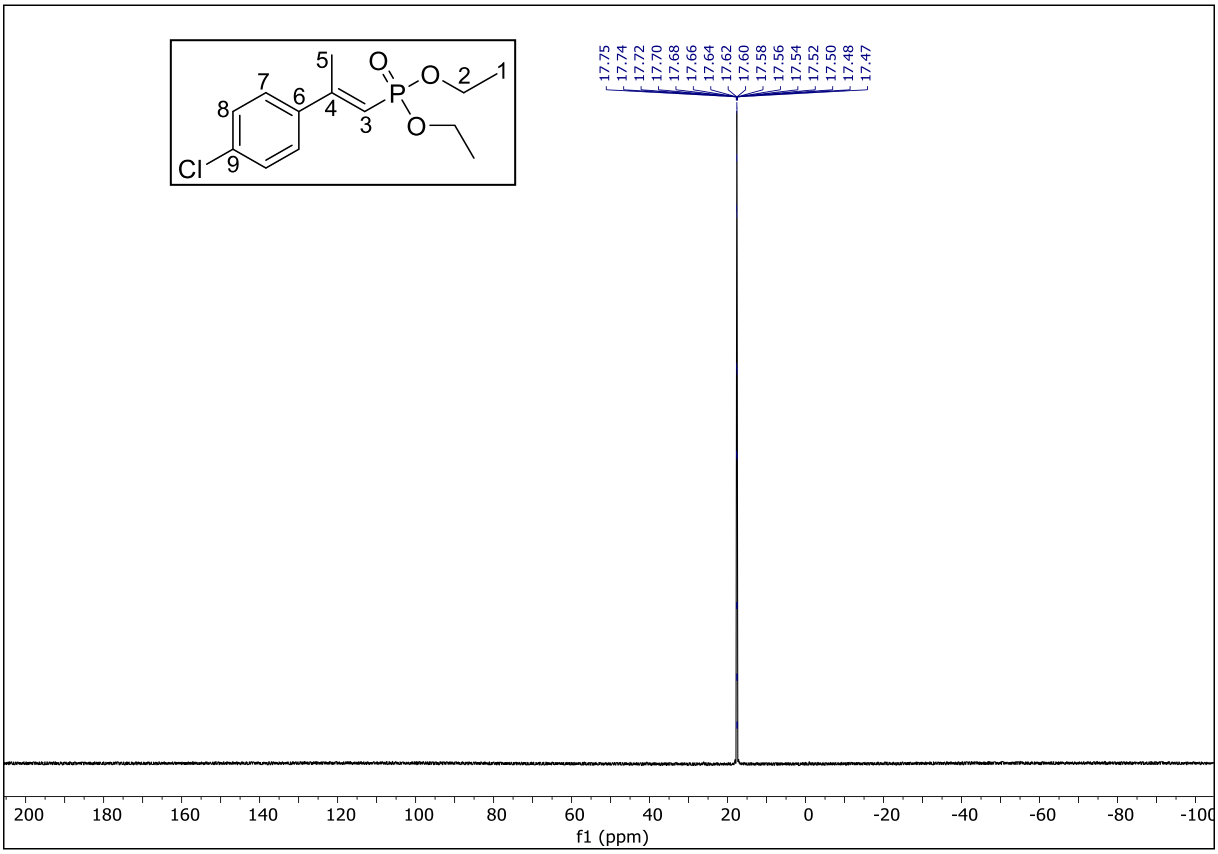

^{1}H NMR (500 MHz, $CDCl_3$): ***E*-4**

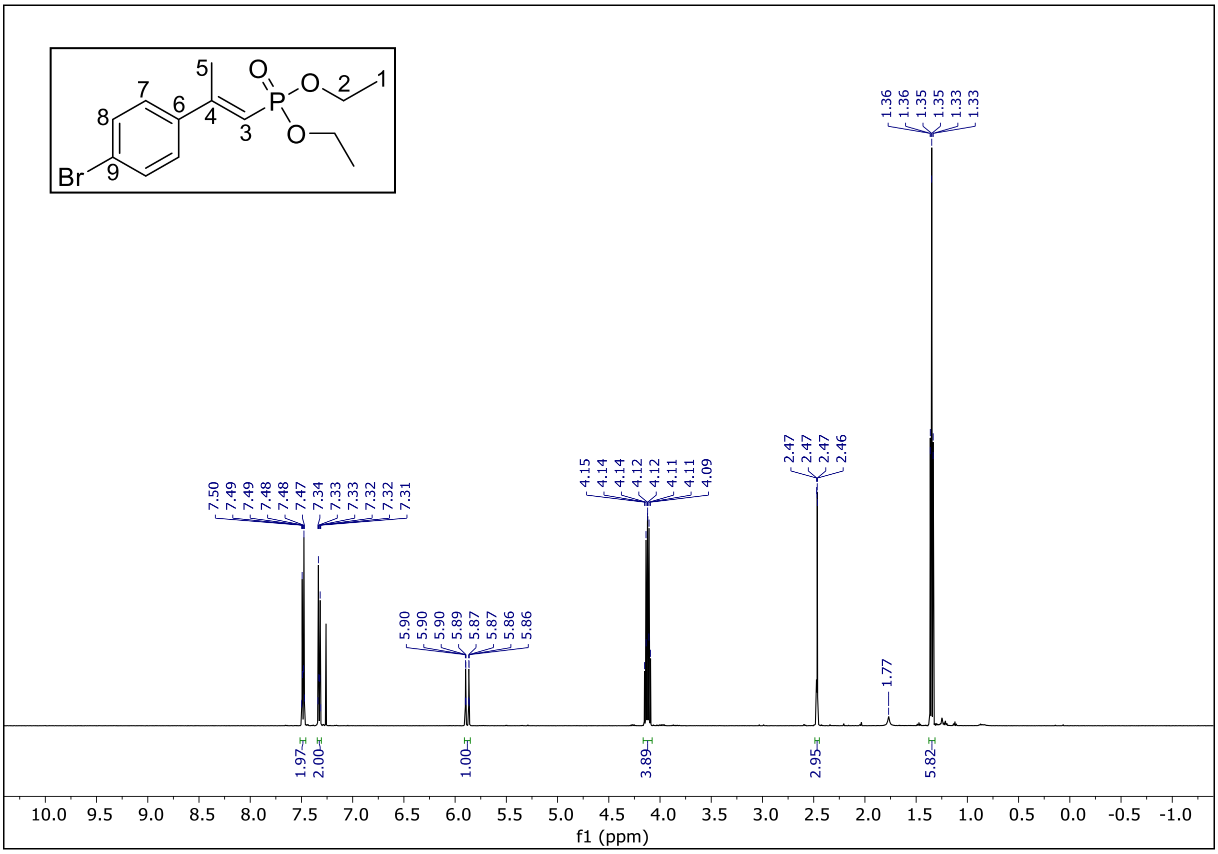

^{13}C NMR (126 MHz, $CDCl_3$): ***E*-4**

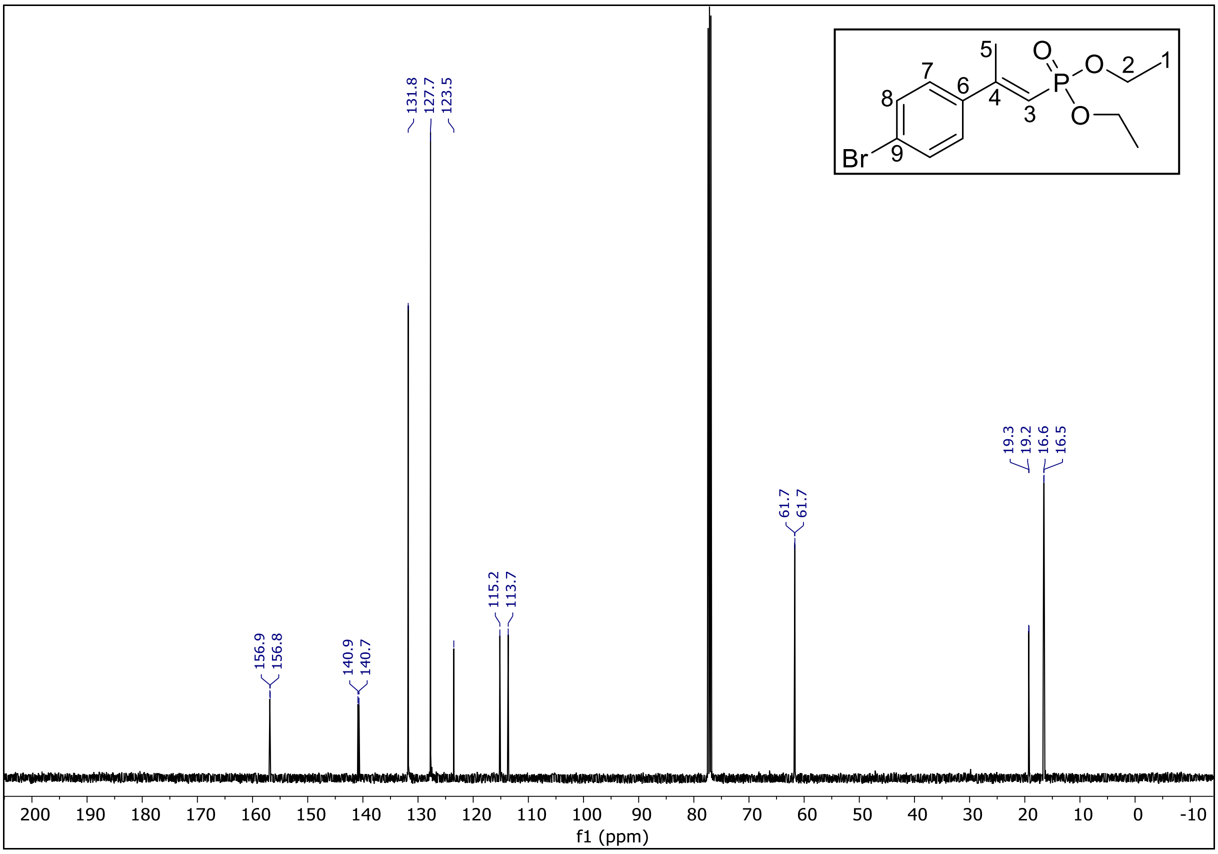

^{31}P NMR (202 MHz, $CDCl_3$): ***E*-4**

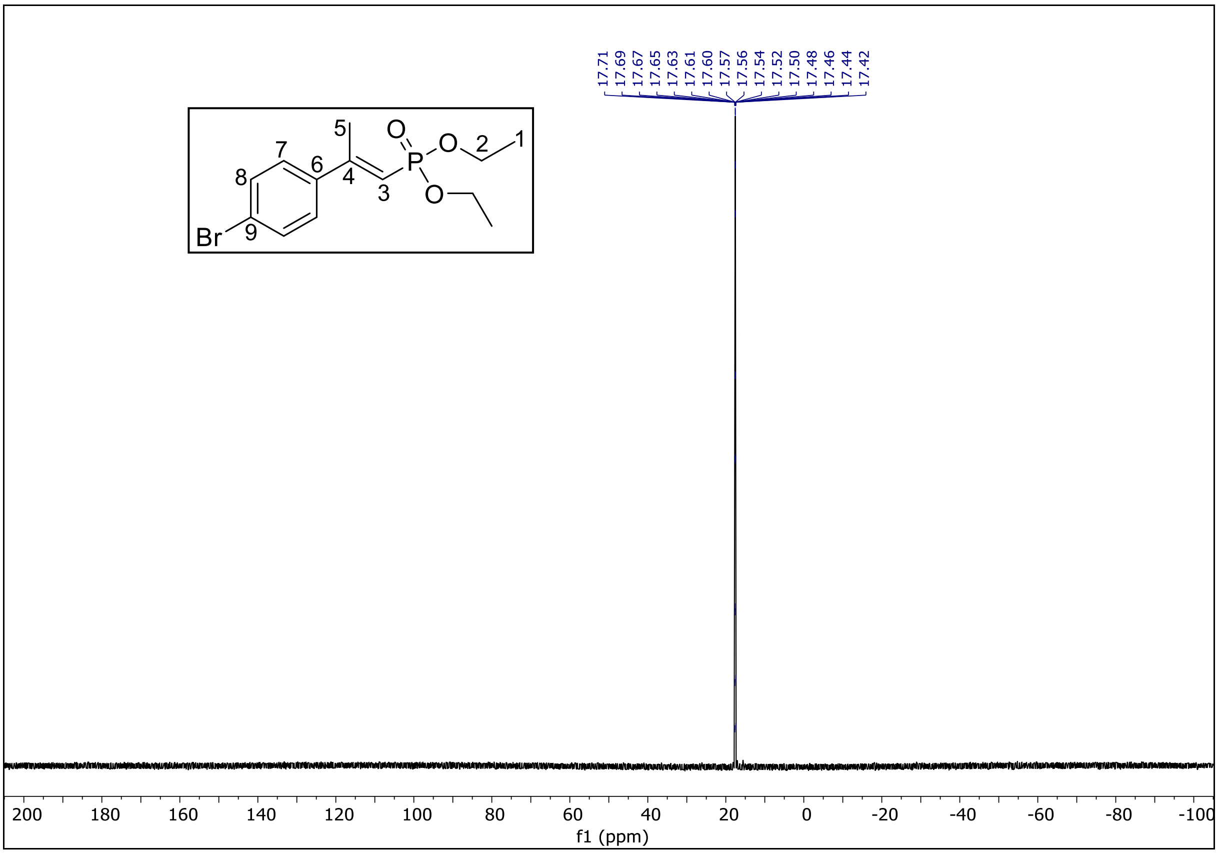

^{1}H NMR (500 MHz, $CDCl_3$): ***E*-5**

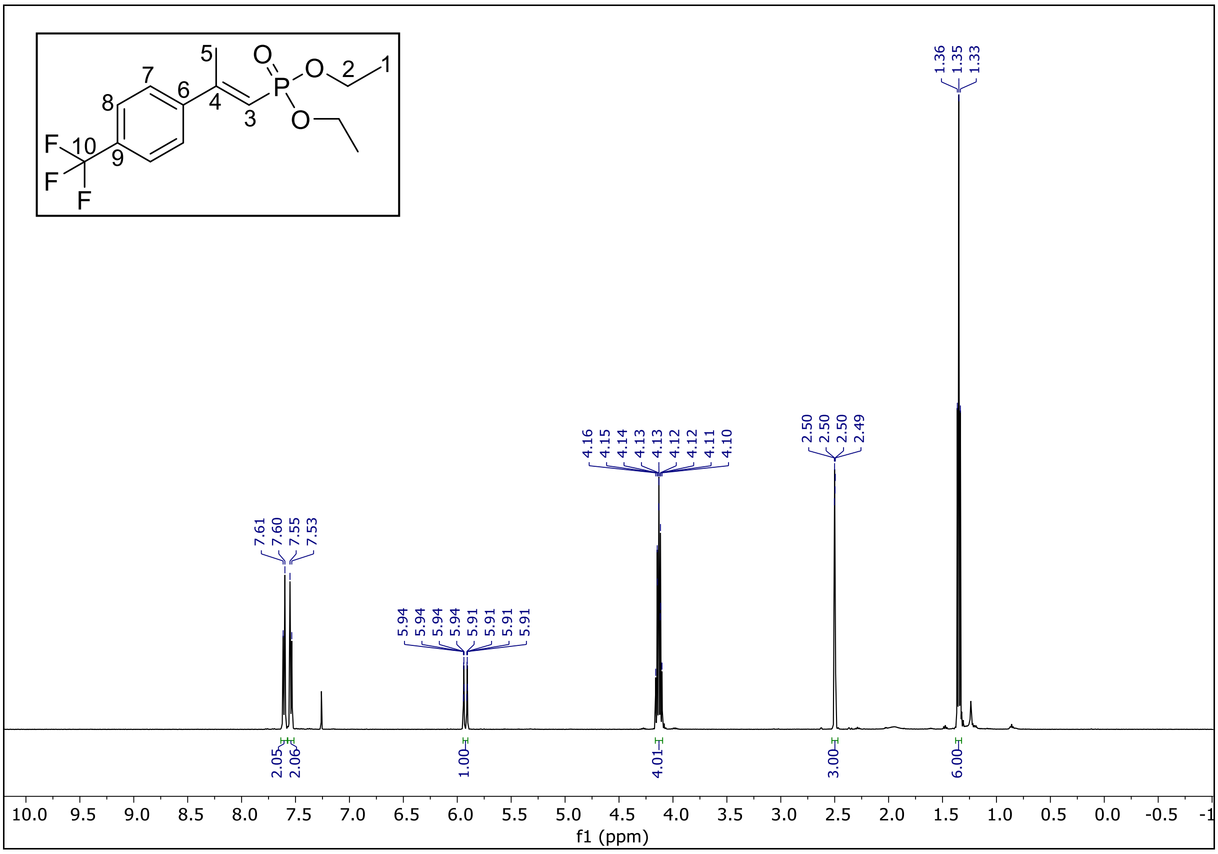

^{13}C NMR (126 MHz, $CDCl_3$): ***E*-5**

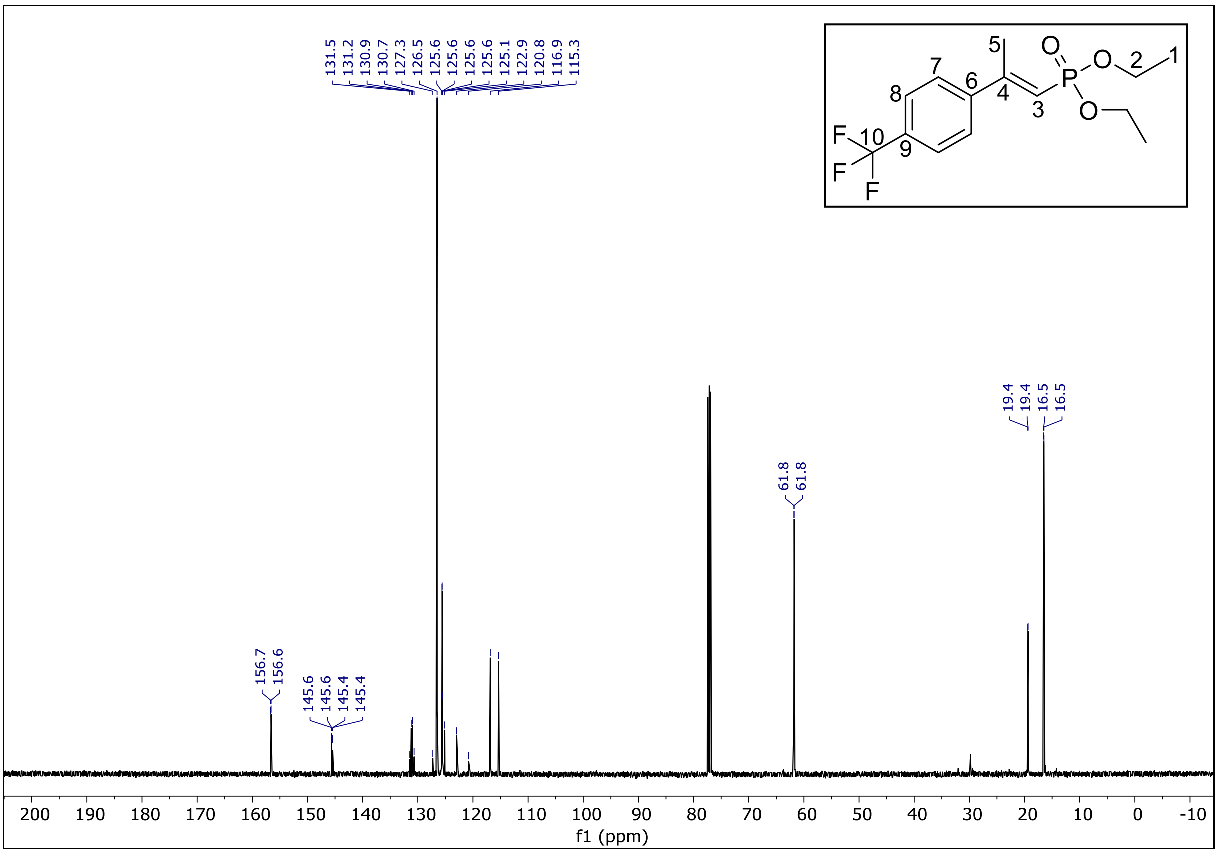

^{19}F NMR (470 MHz, $CDCl_3$): ***E*-5**

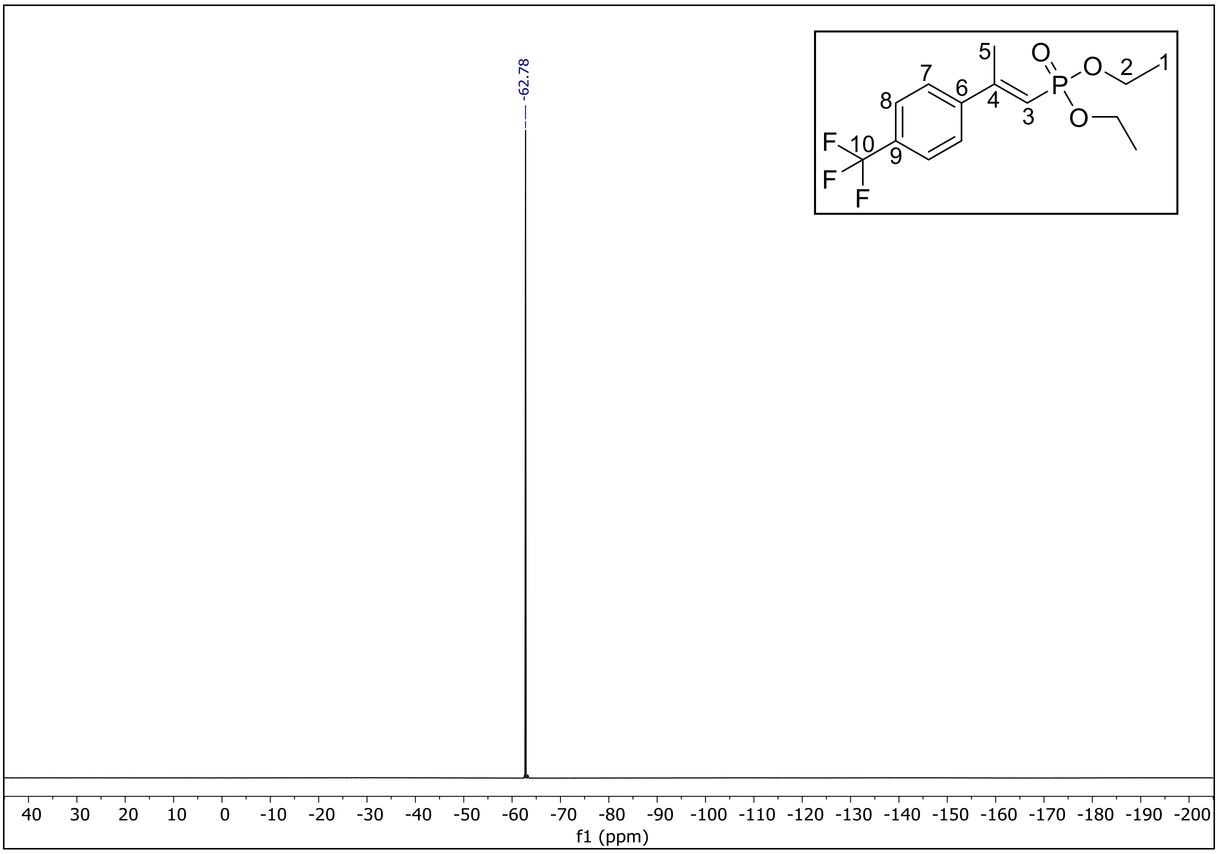

^{31}P NMR (202 MHz, $CDCl_3$): ***E*-5**

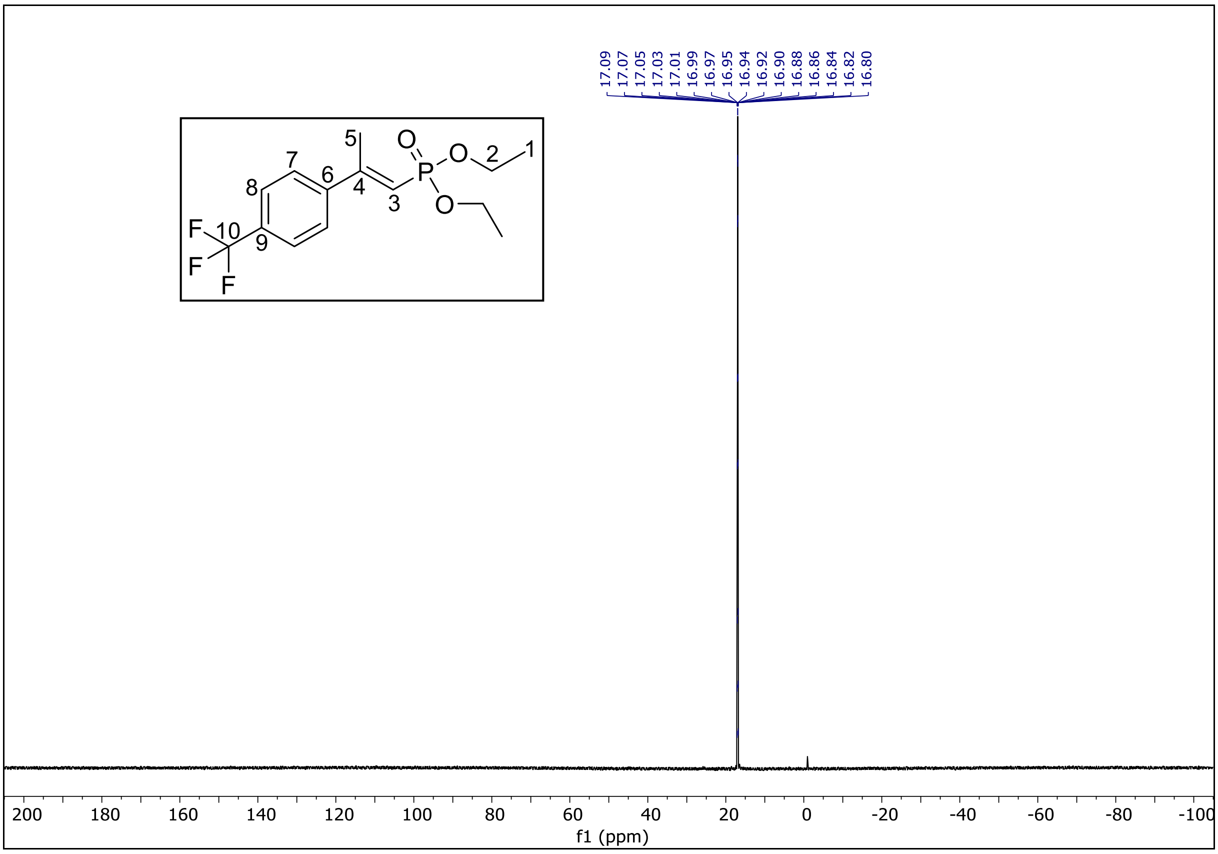

^{1}H NMR (500 MHz, $CDCl_3$): ***E*-6**

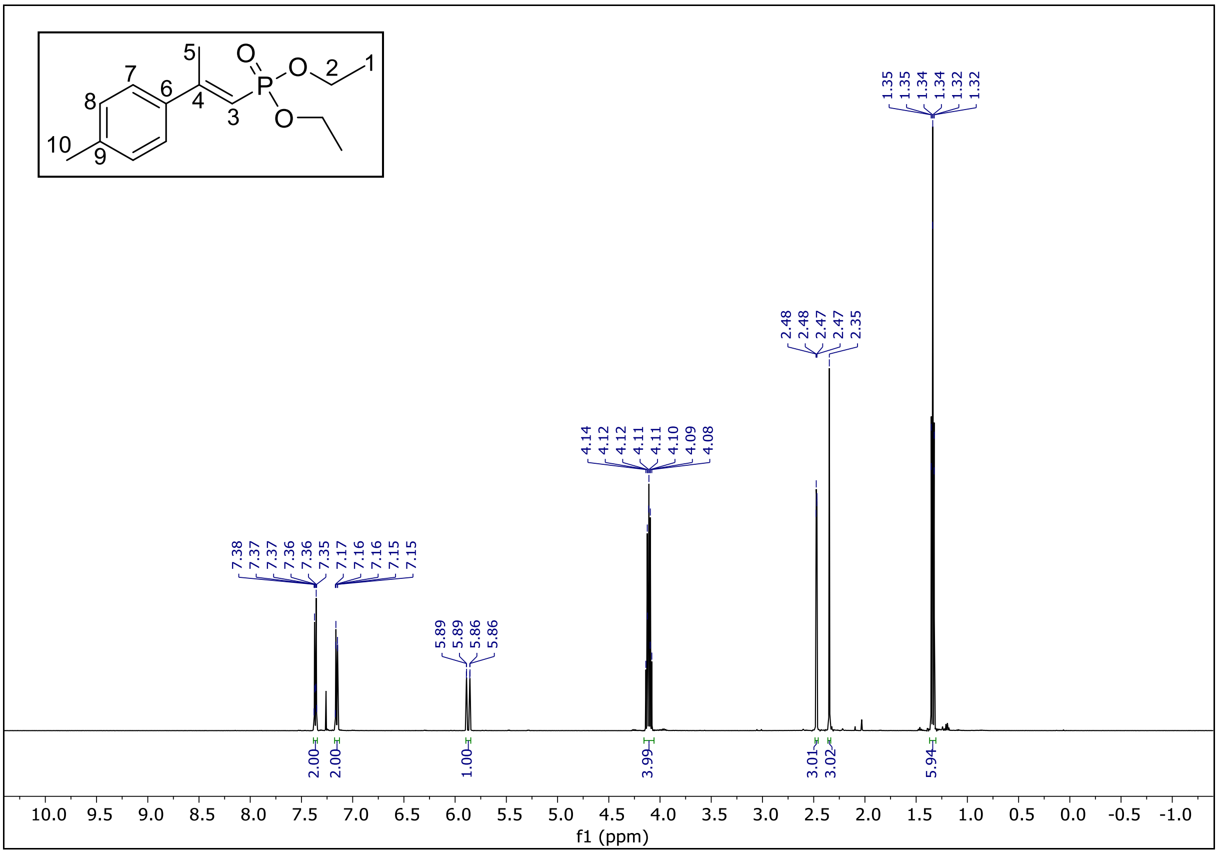

^{13}C NMR (126 MHz, $CDCl_3$): ***E*-6**

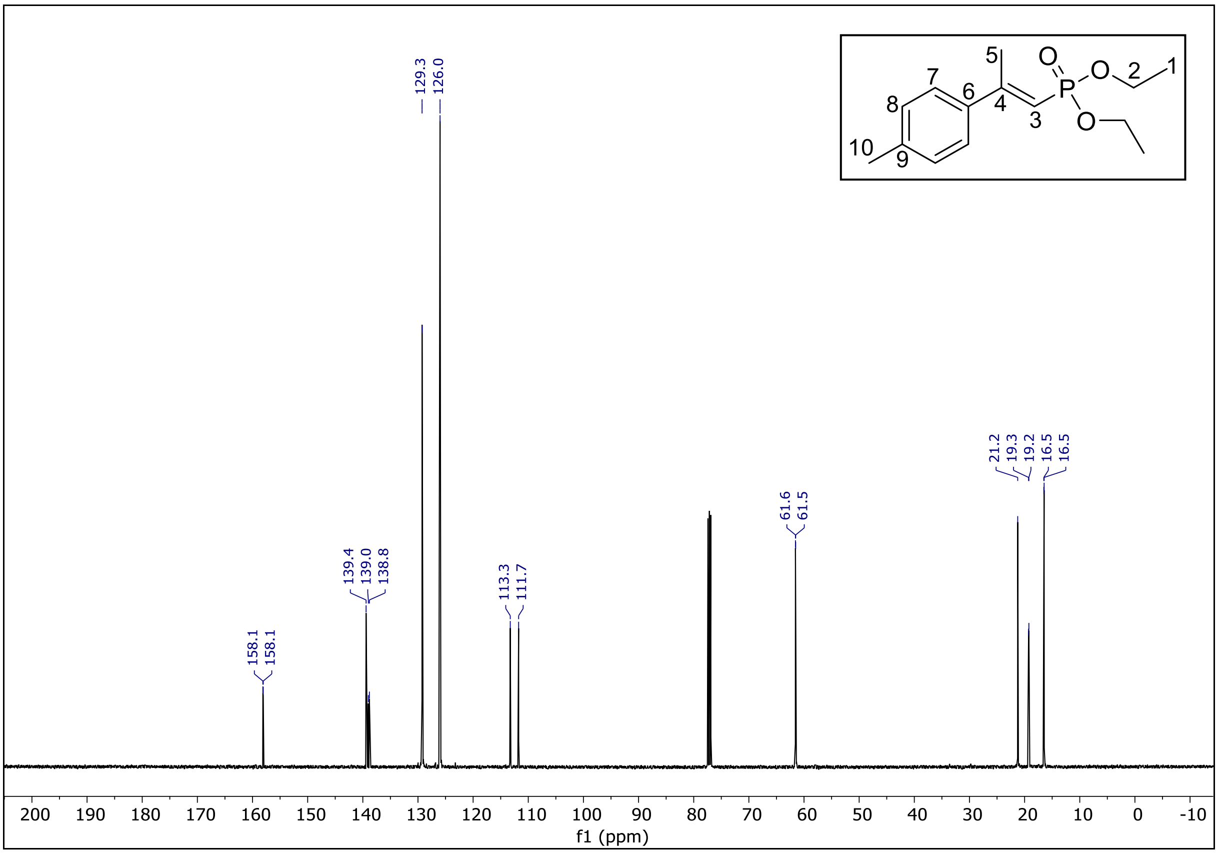

^{31}P NMR (162 MHz, $CDCl_3$): ***E*-6**

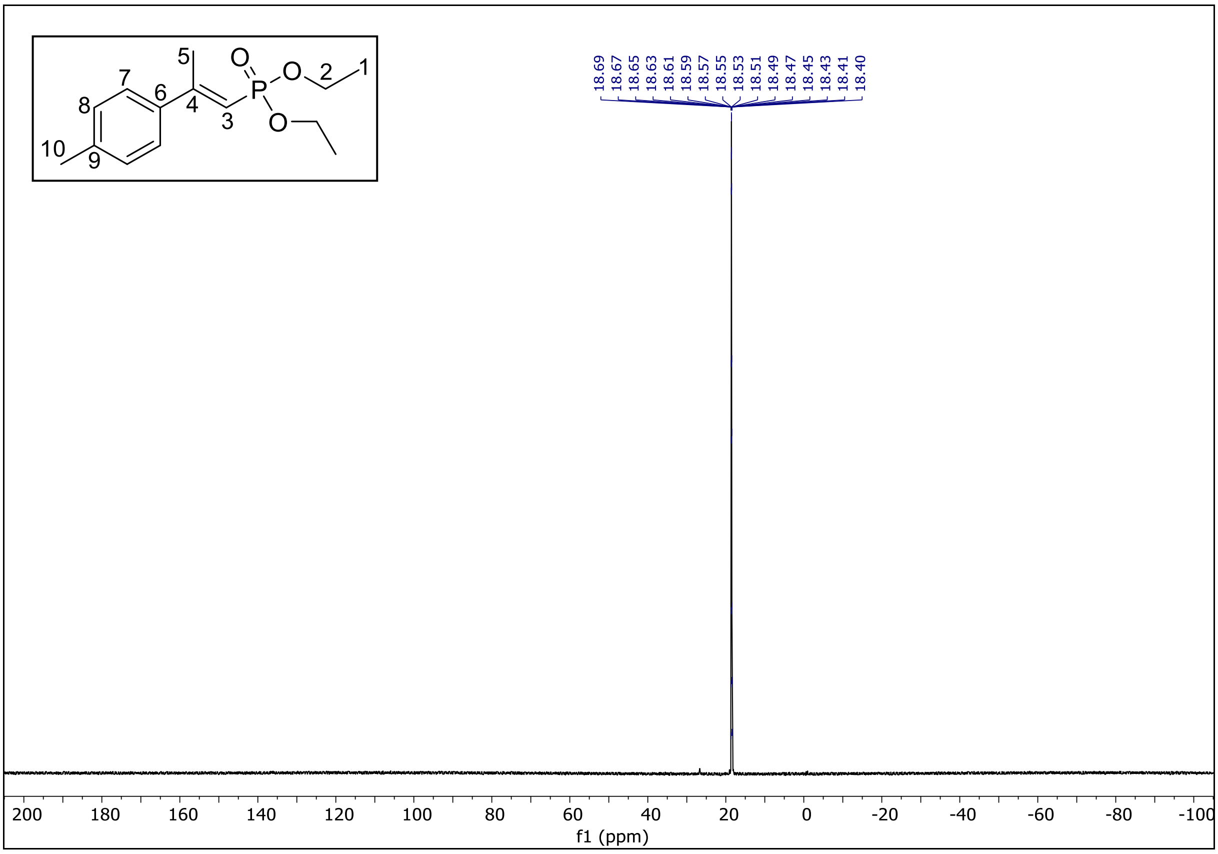

^{1}H NMR (500 MHz, CDCl$_3$): ***E*-7**

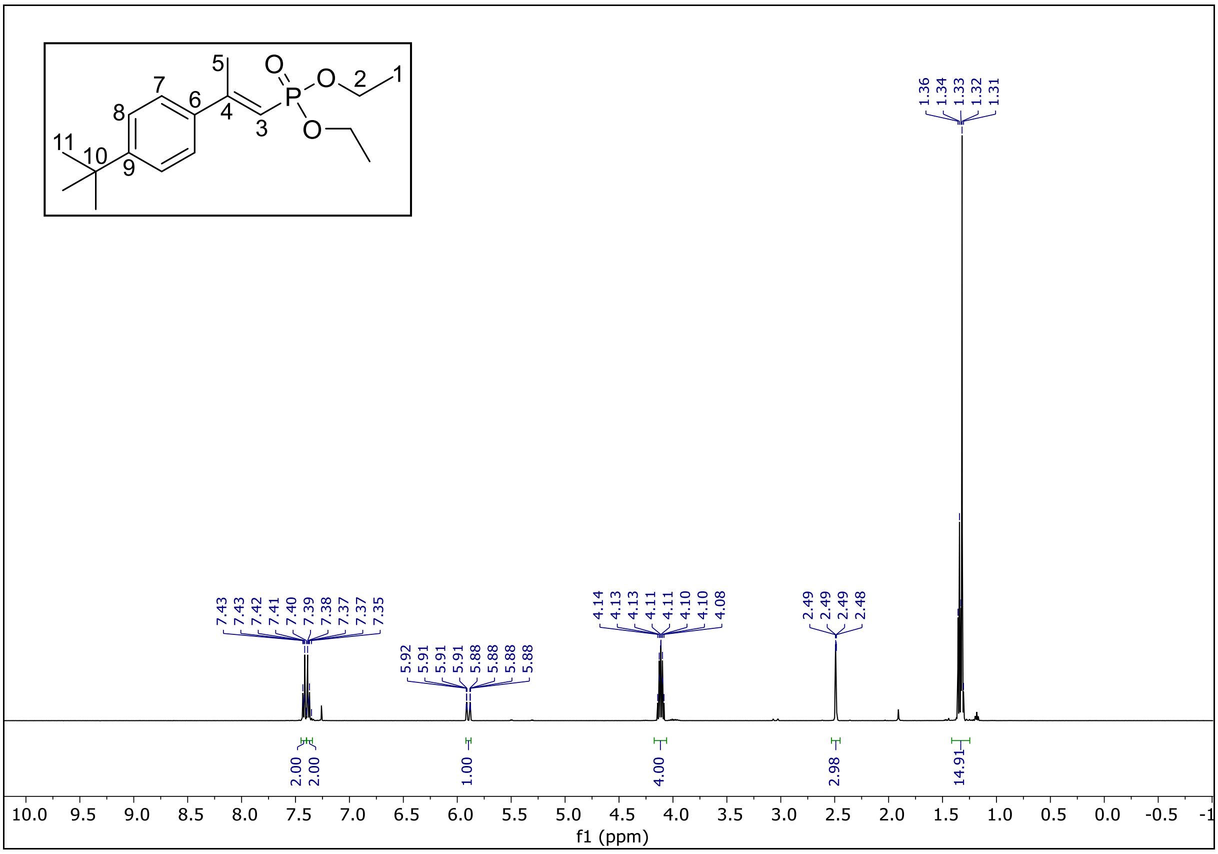

^{13}C NMR (126 MHz, CDCl$_3$): ***E*-7**

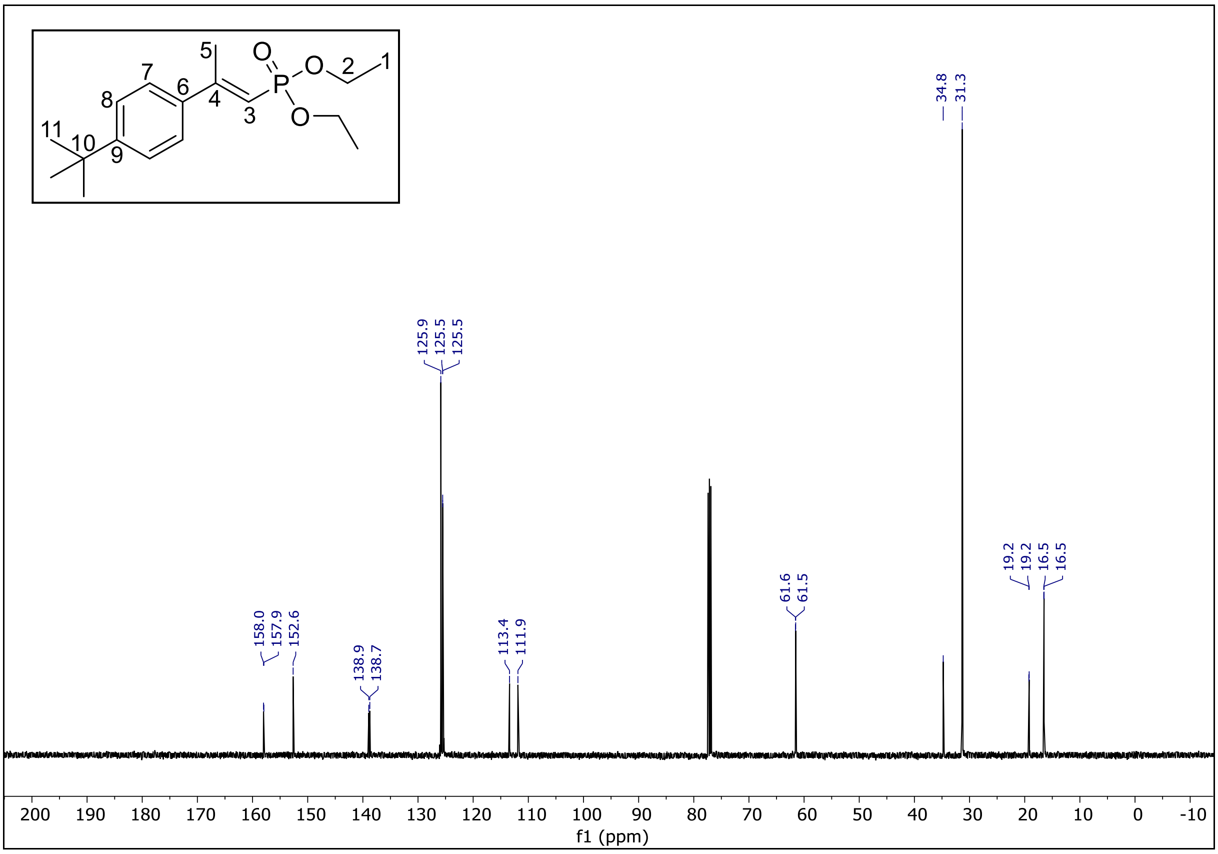

^{31}P NMR (202 MHz, $CDCl_3$): ***E*-7**

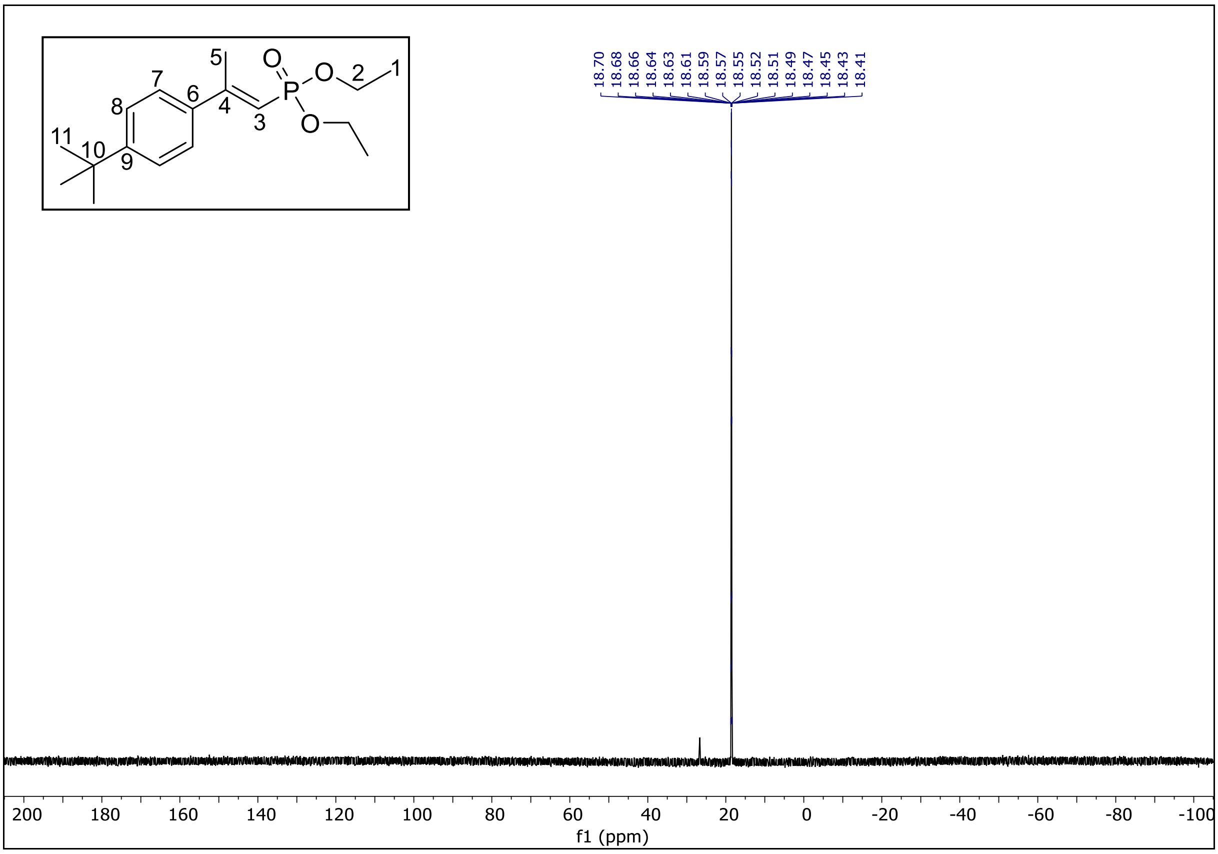

^{1}H NMR (600 MHz, $CDCl_3$): ***E*-8**

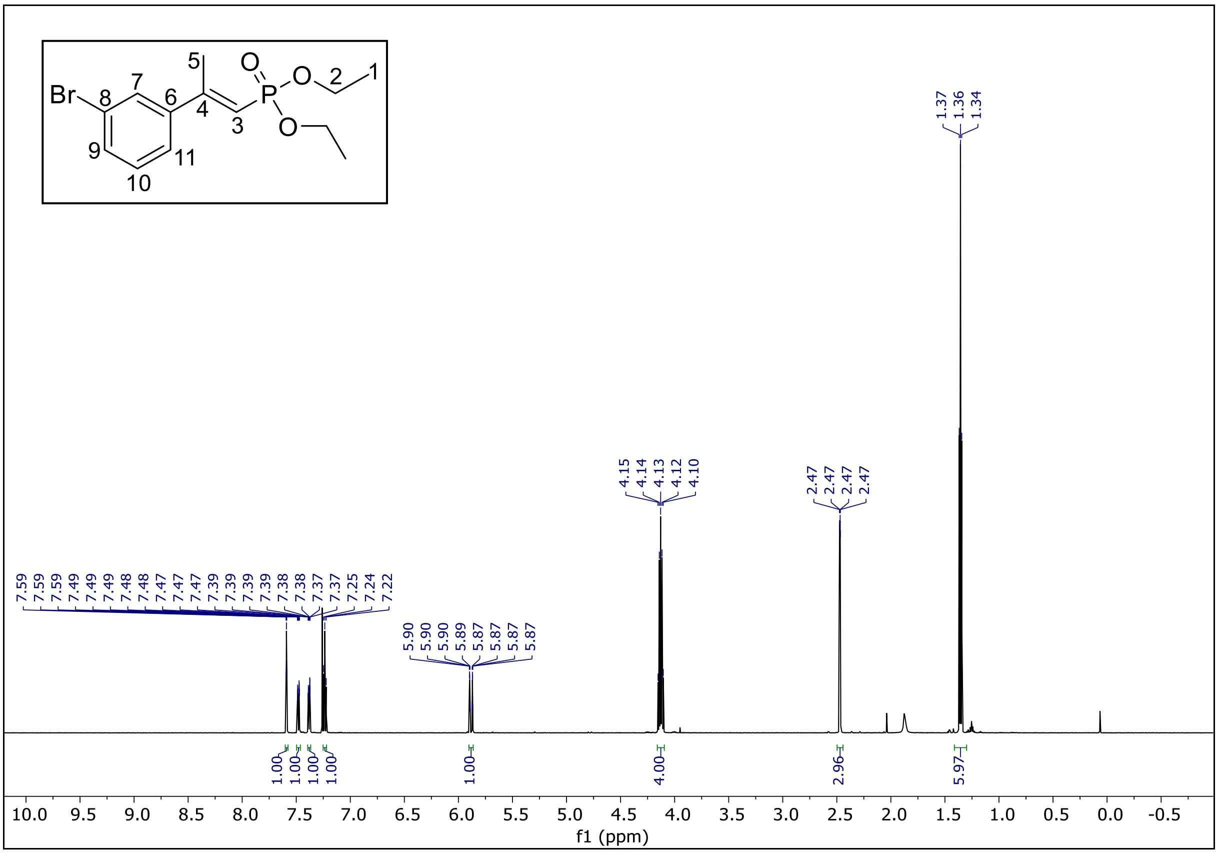

^{13}C NMR (151 MHz, $CDCl_3$): ***E*-8**

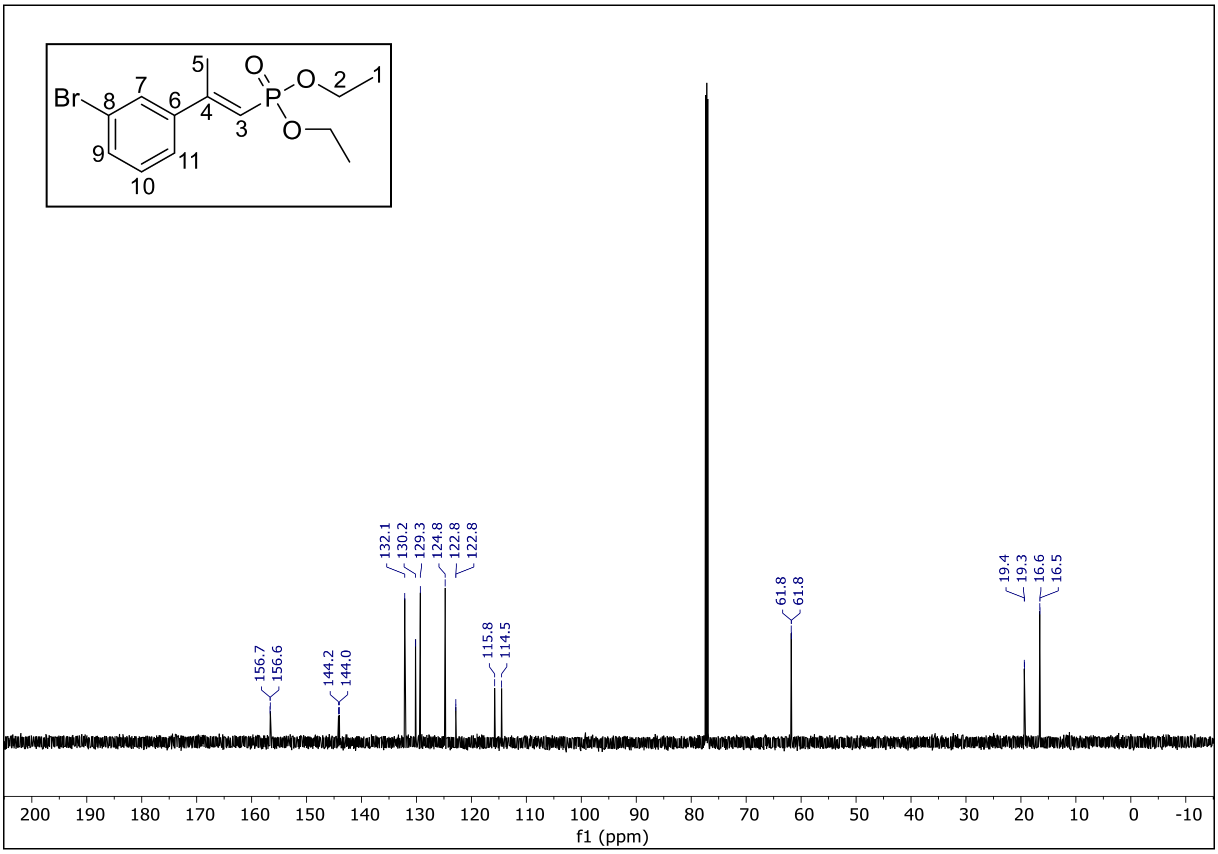

^{31}P NMR (243 MHz, $CDCl_3$): ***E*-8**

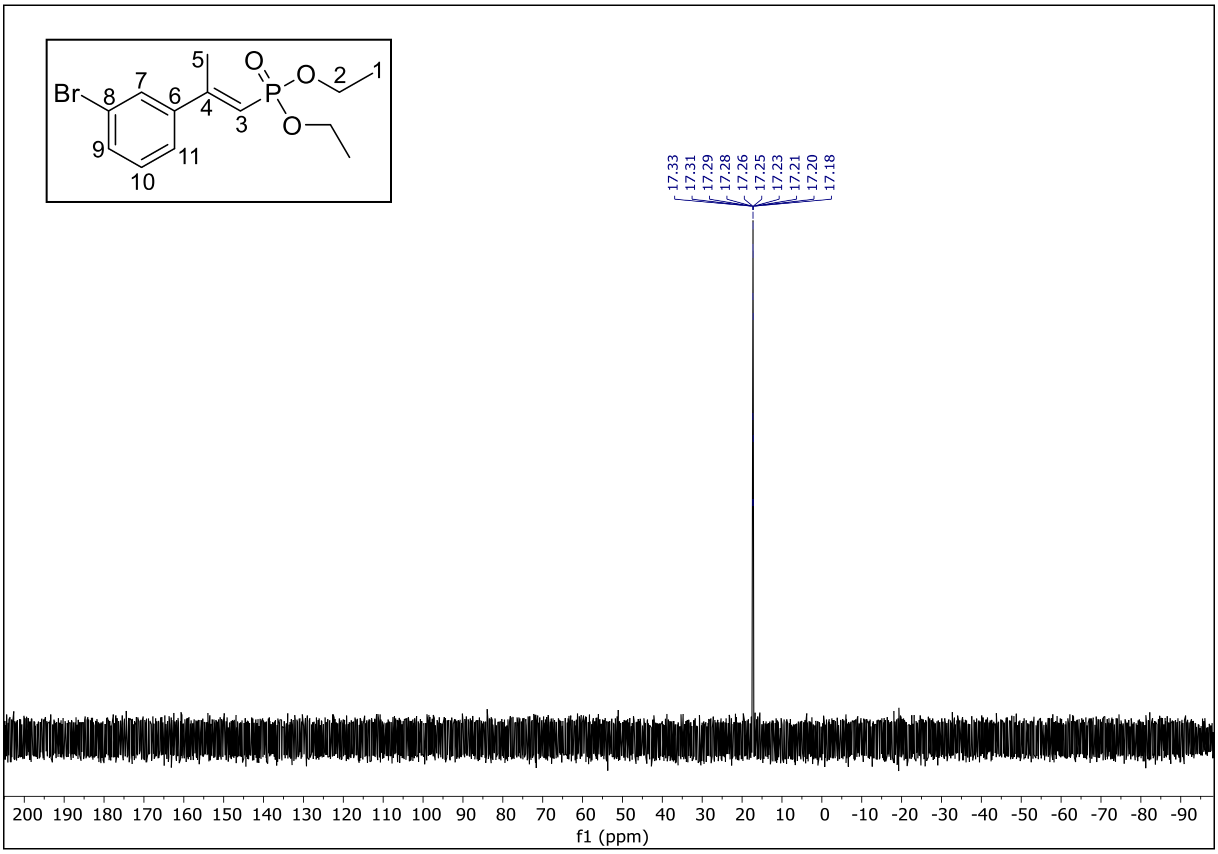

^{1}H NMR (600 MHz, $CDCl_3$): ***E*-9**

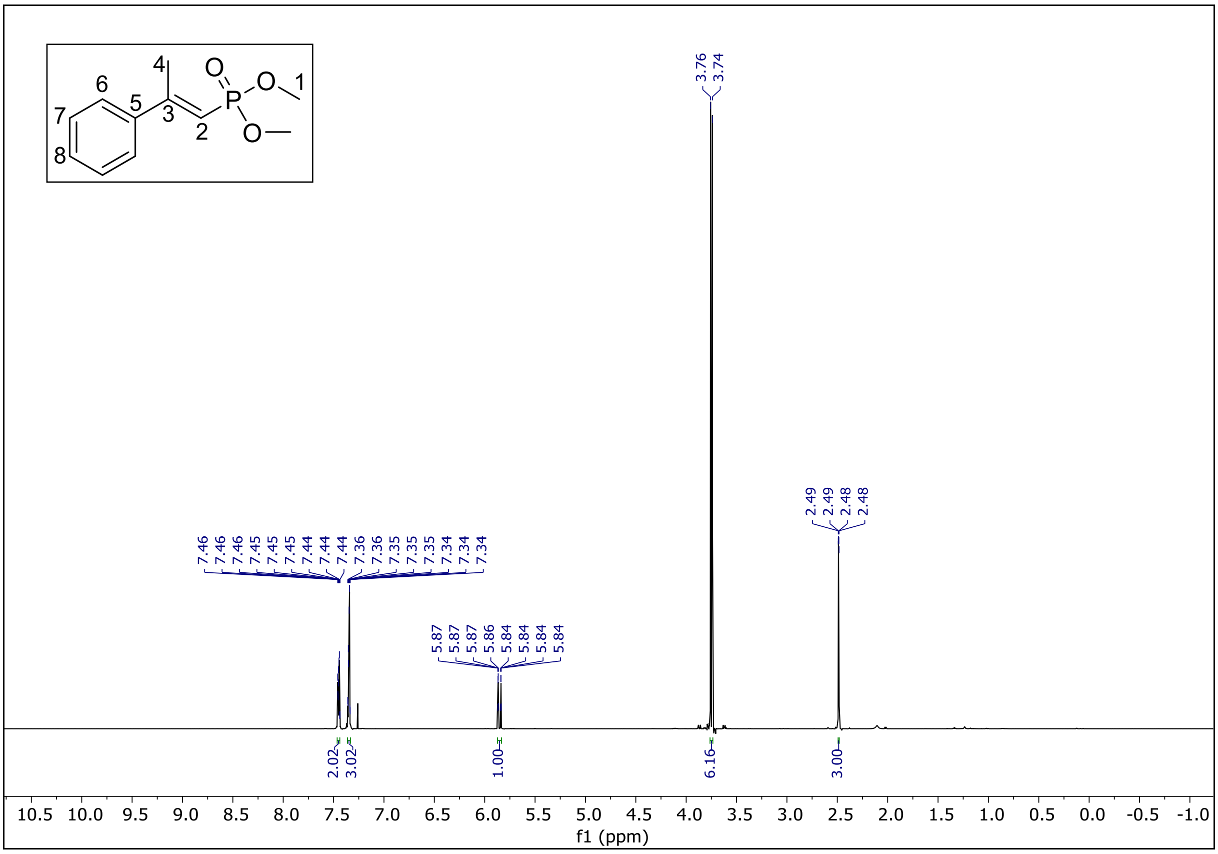

^{13}C NMR (151 MHz, $CDCl_3$): ***E*-9**

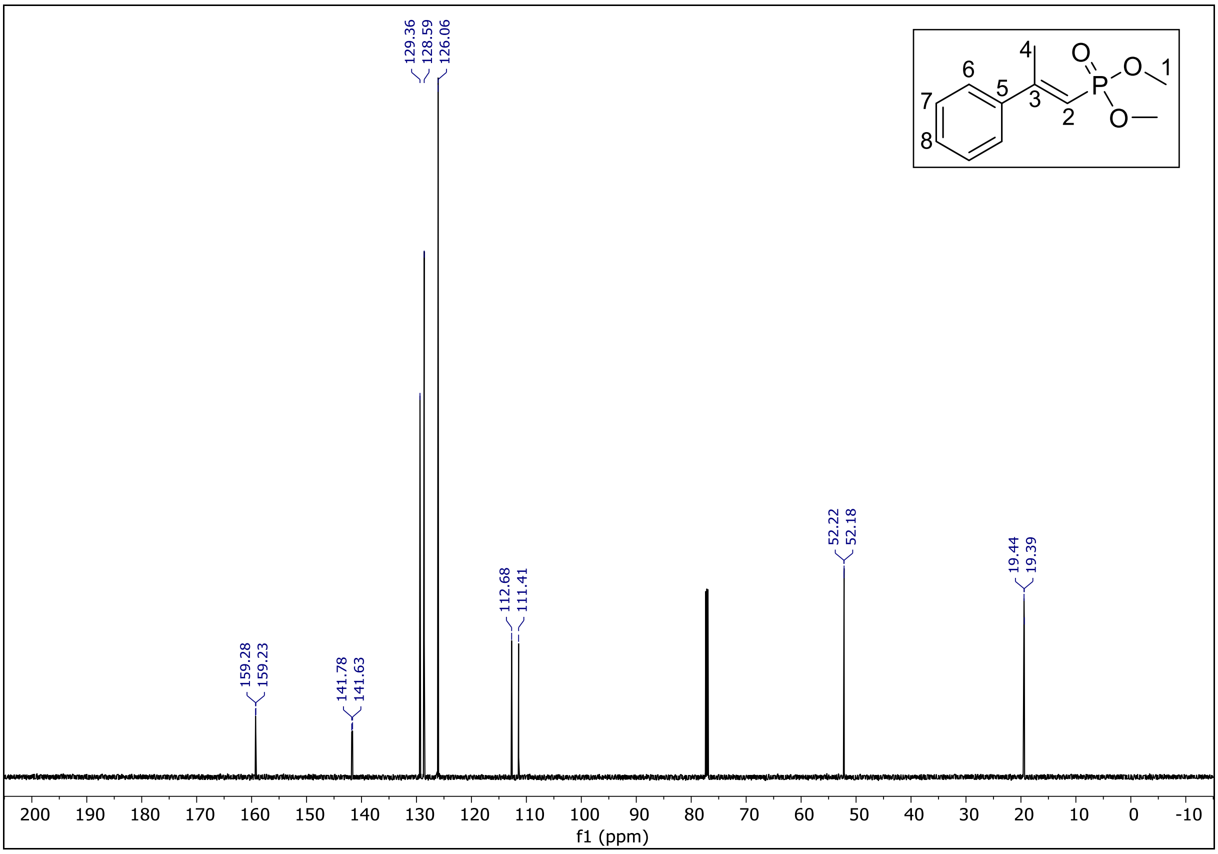

^{31}P NMR (162 MHz, $CDCl_3$): ***E*-9**

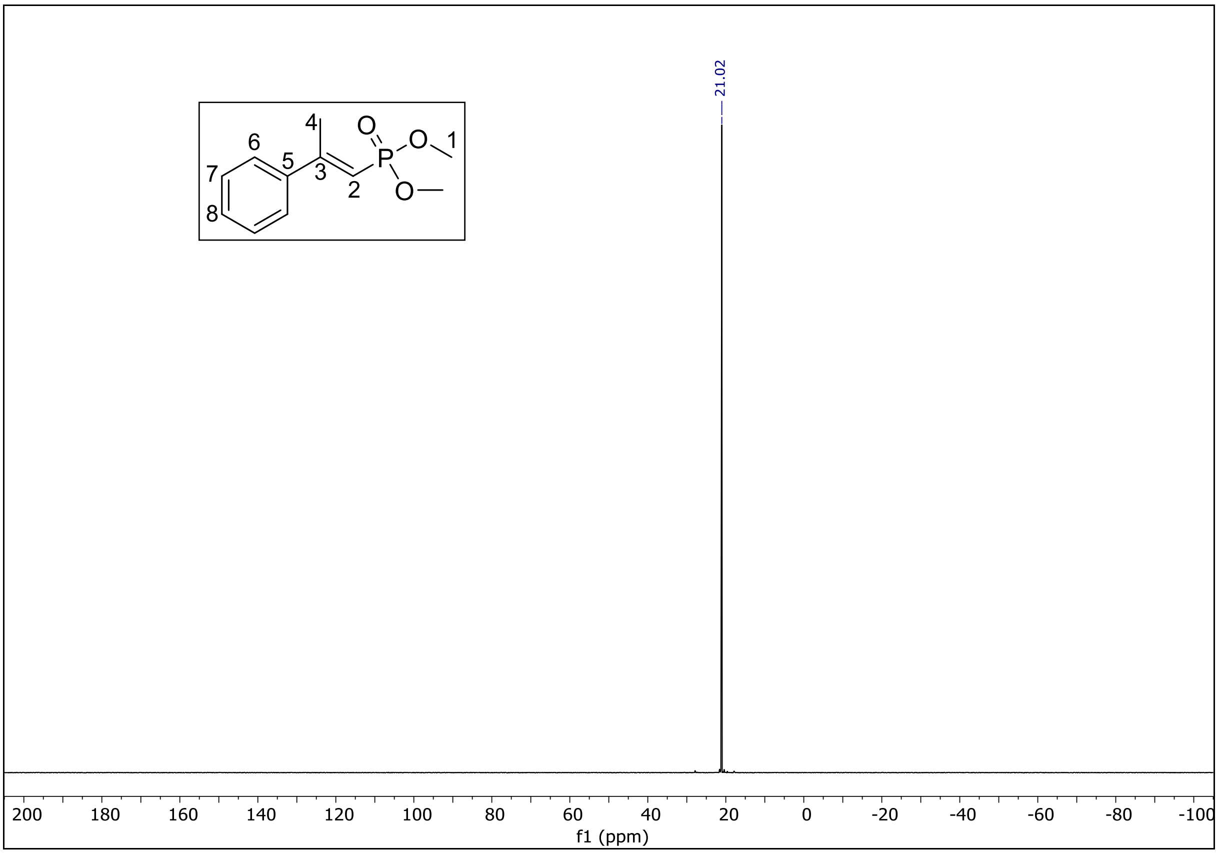

^{1}H NMR (500 MHz, $CDCl_3$): ***E*-10**

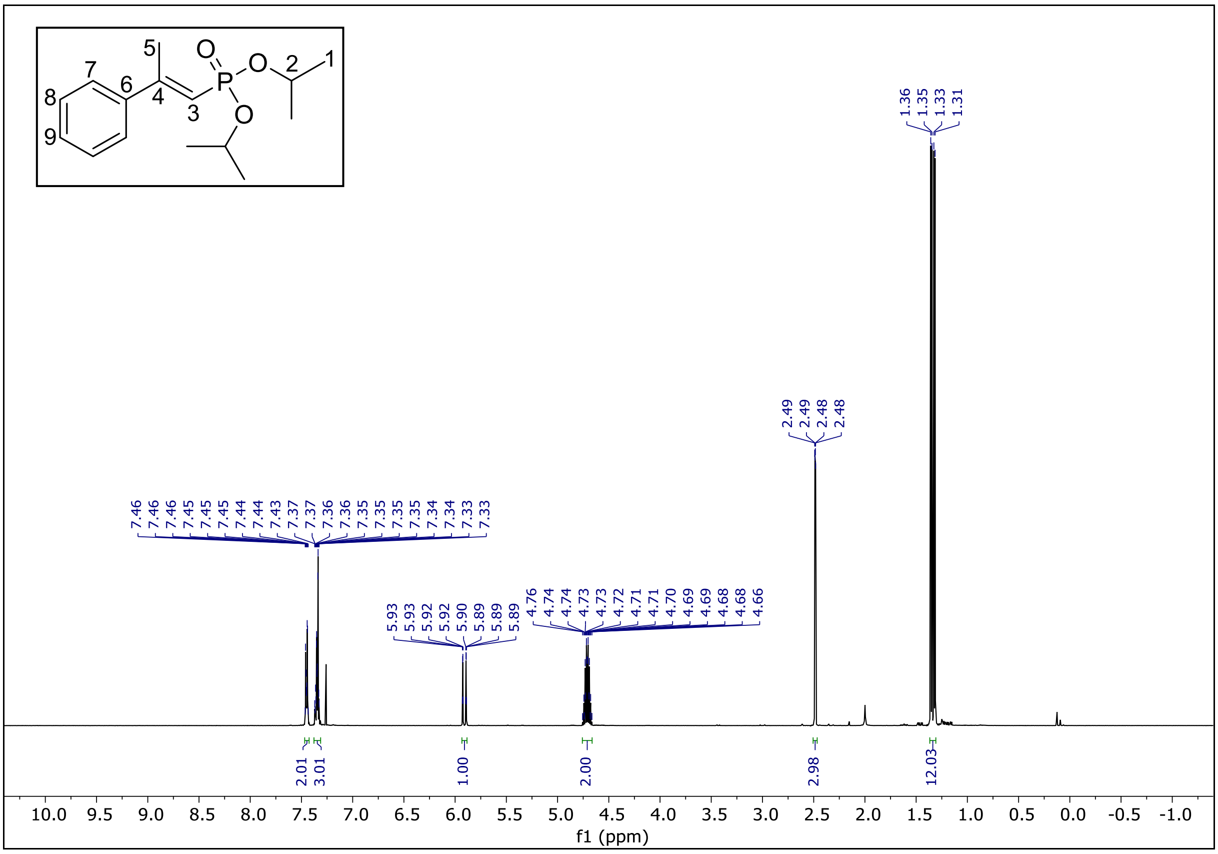

^{13}C NMR (126 MHz, $CDCl_3$): ***E*-10**

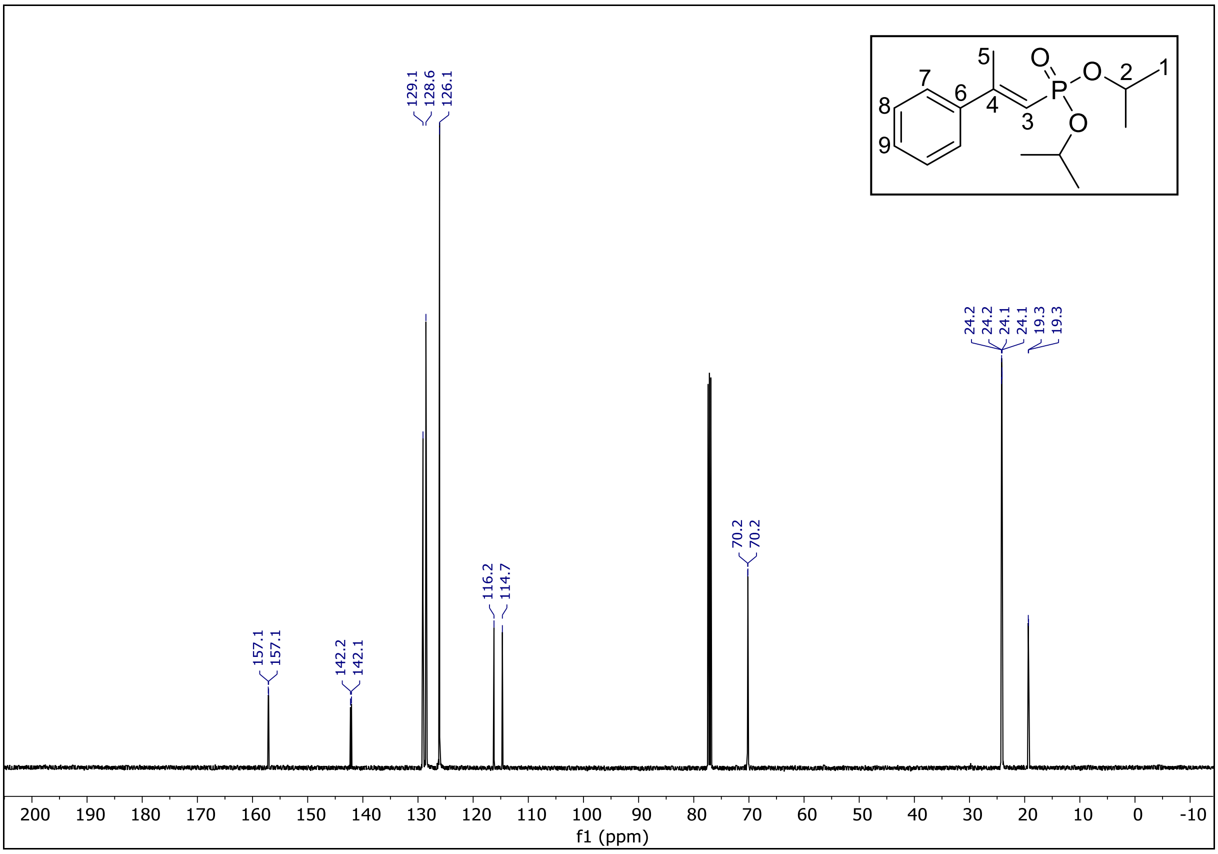

^{31}P NMR (202 MHz, $CDCl_3$): ***E*-10**

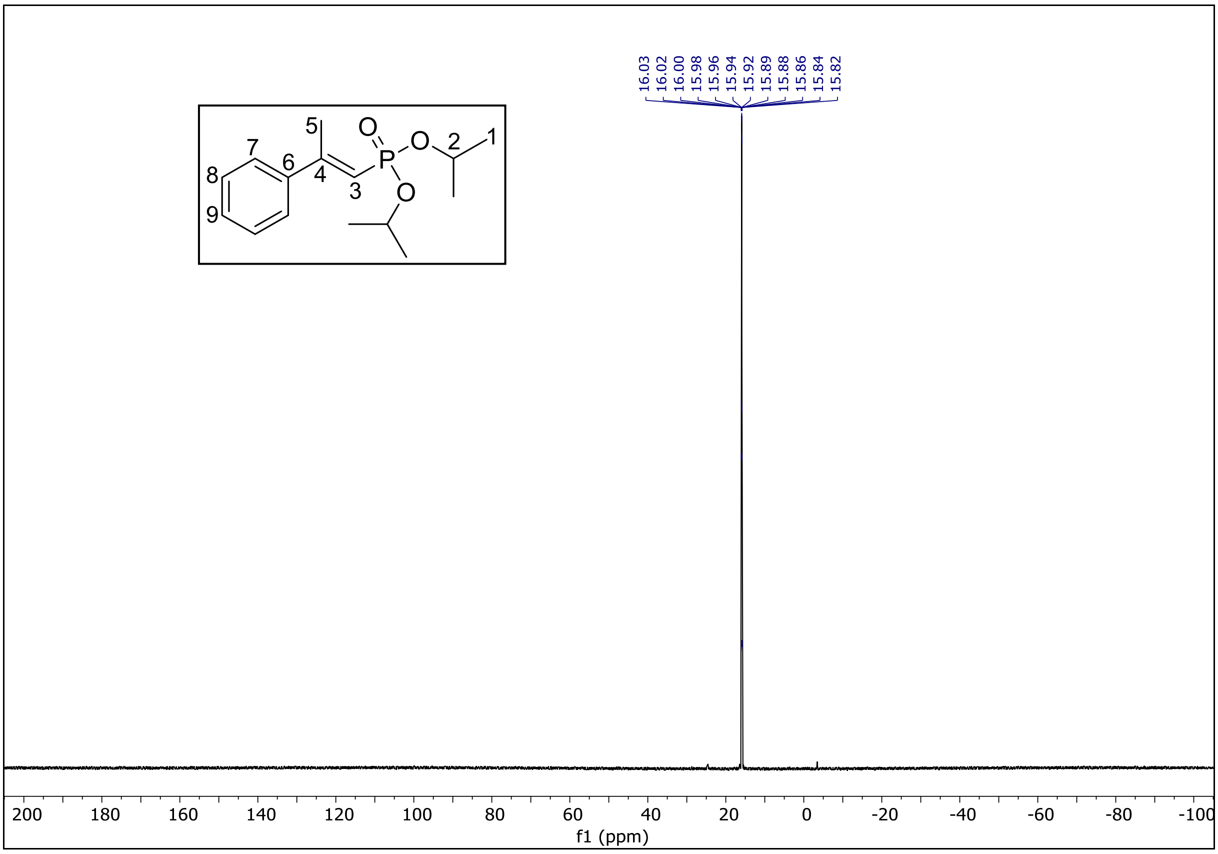

^{1}H NMR (600 MHz, $CDCl_3$): ***E*-11**

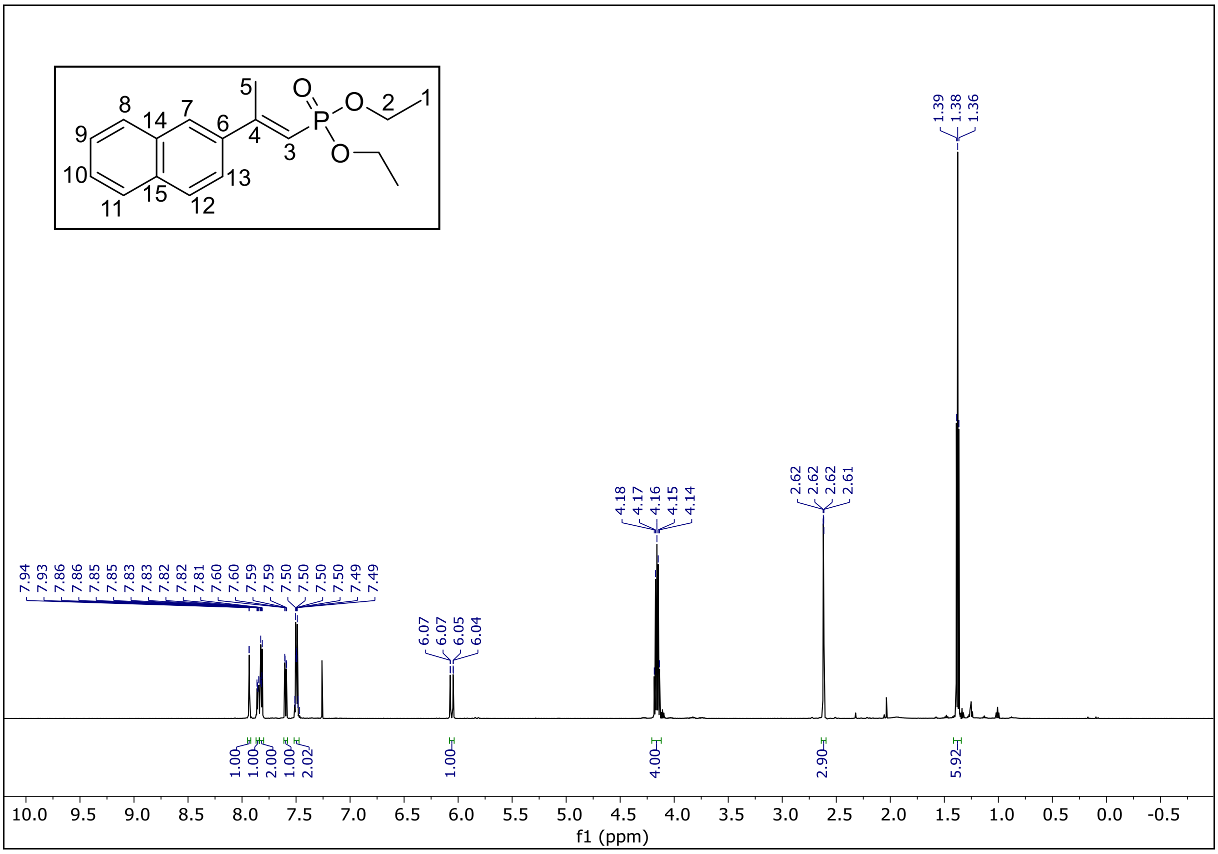

^{13}C NMR (151 MHz, $CDCl_3$): ***E*-11**

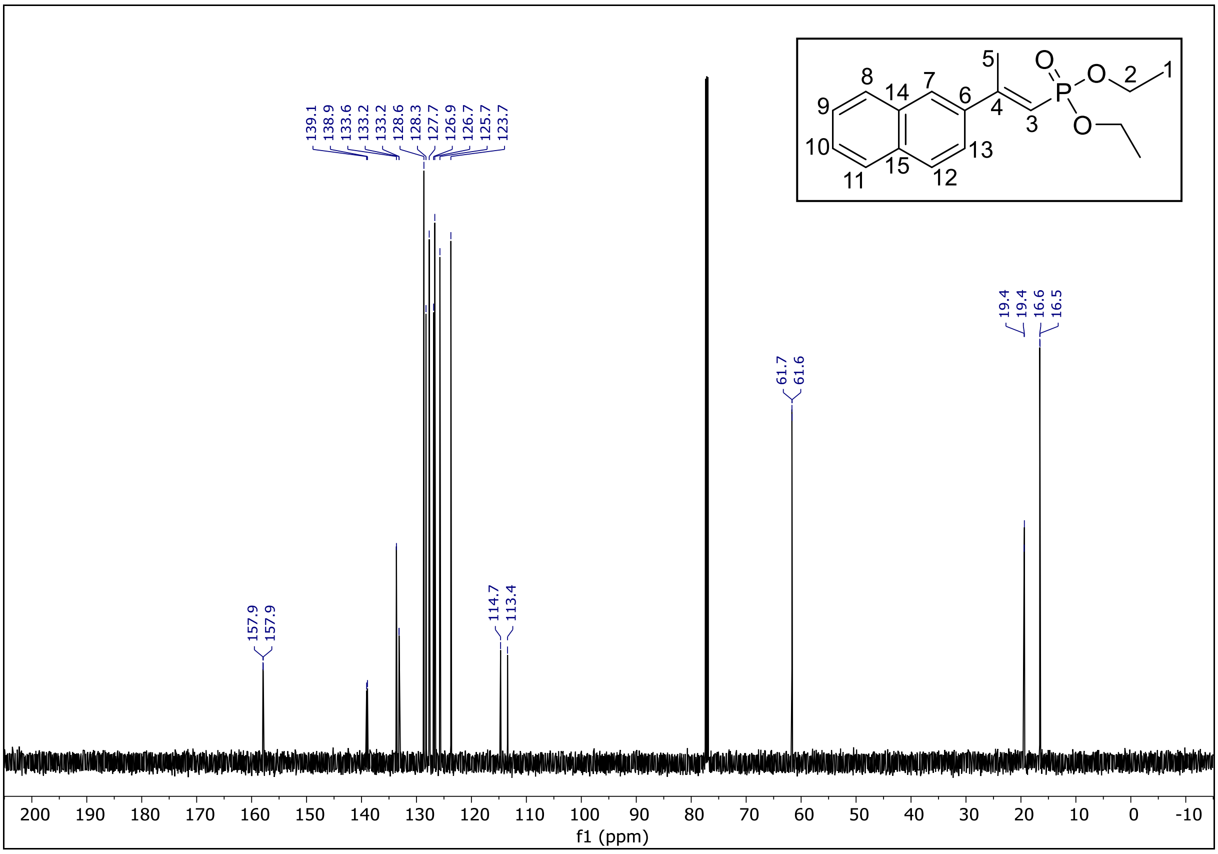

^{31}P NMR (243 MHz, $CDCl_3$): ***E*-11**

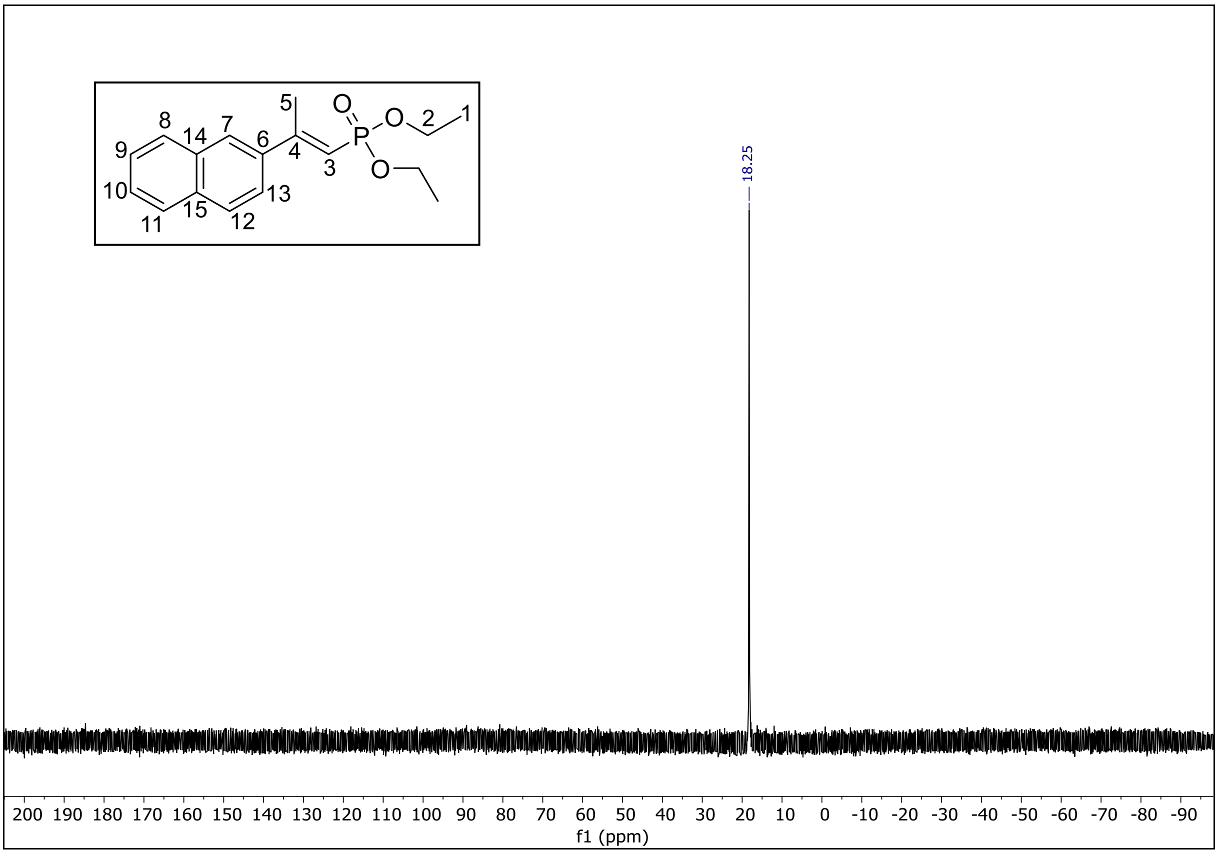

^{1}H NMR (500 MHz, $CDCl_3$): ***E*-12**

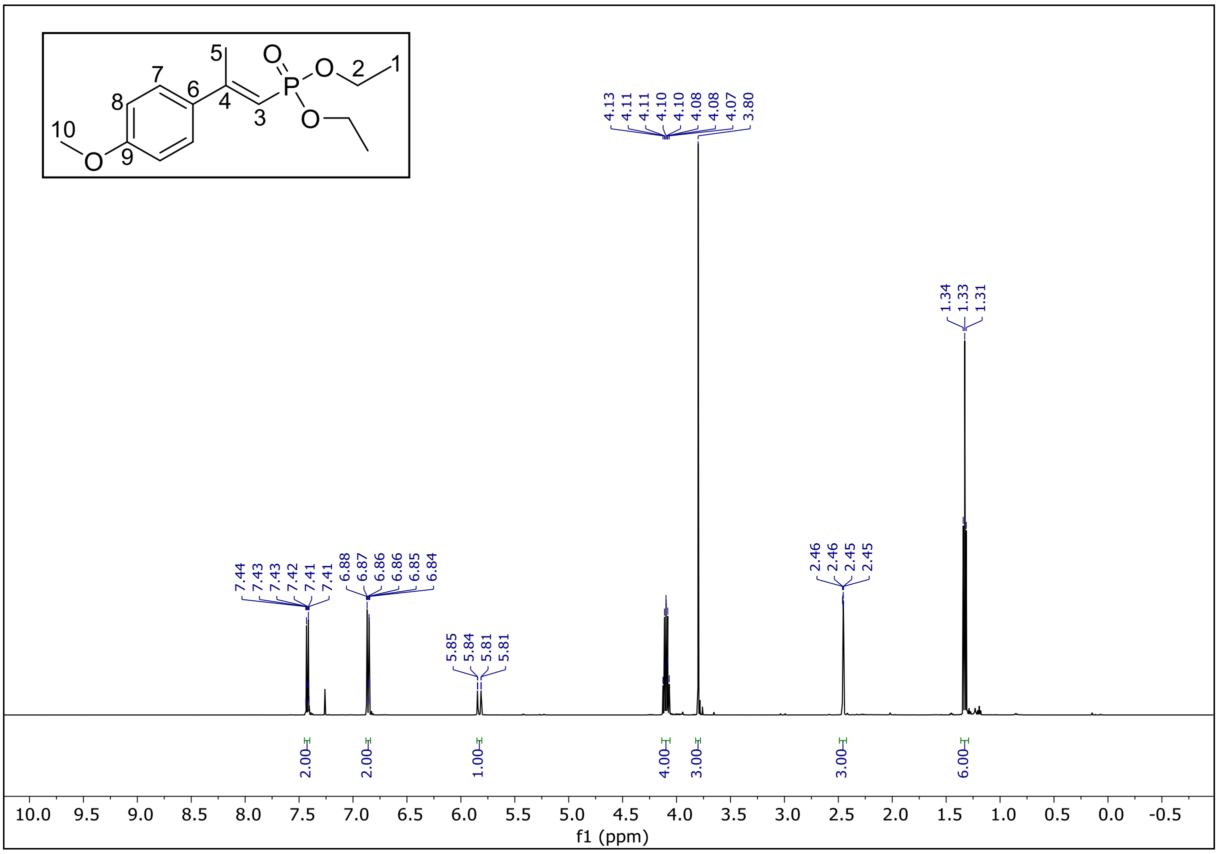

^{13}C NMR (126 MHz, $CDCl_3$): ***E*-12**

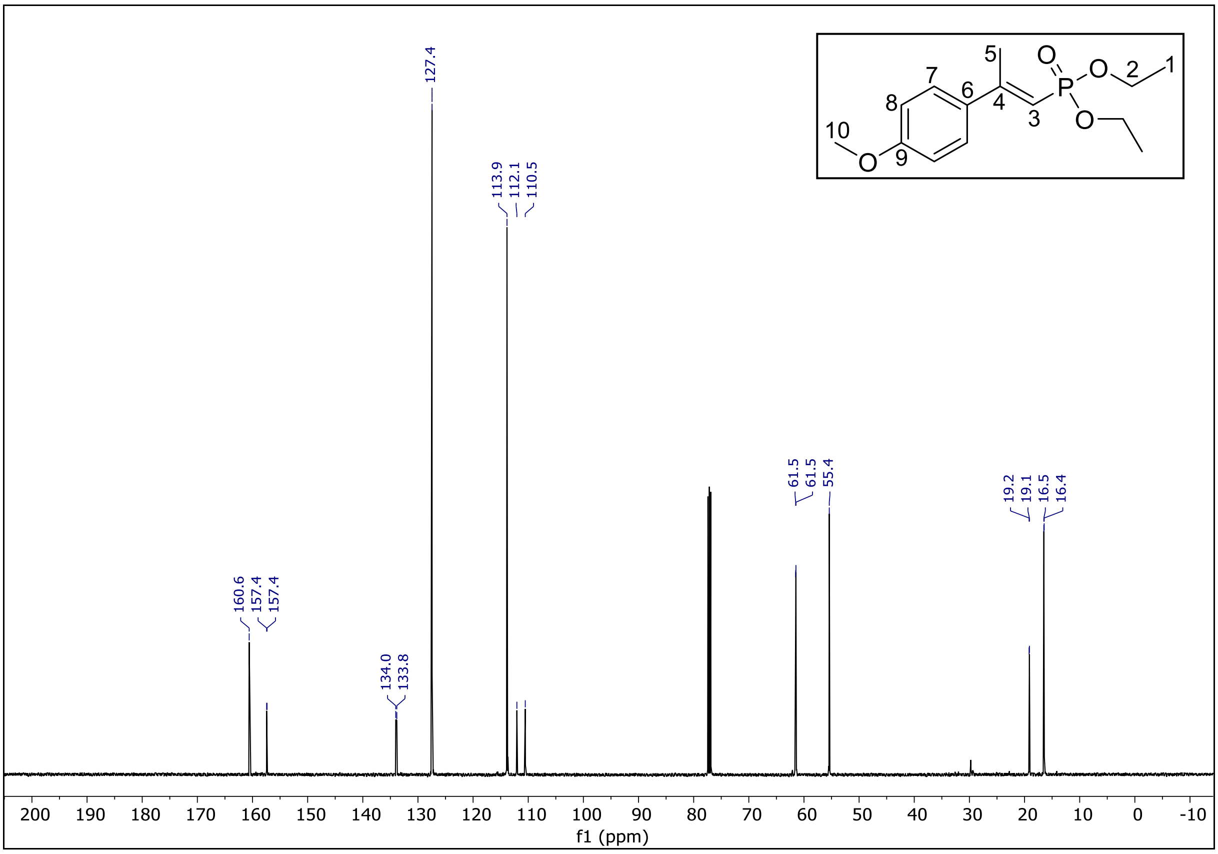

^{31}P NMR (121 MHz, $CDCl_3$): ***E*-12**

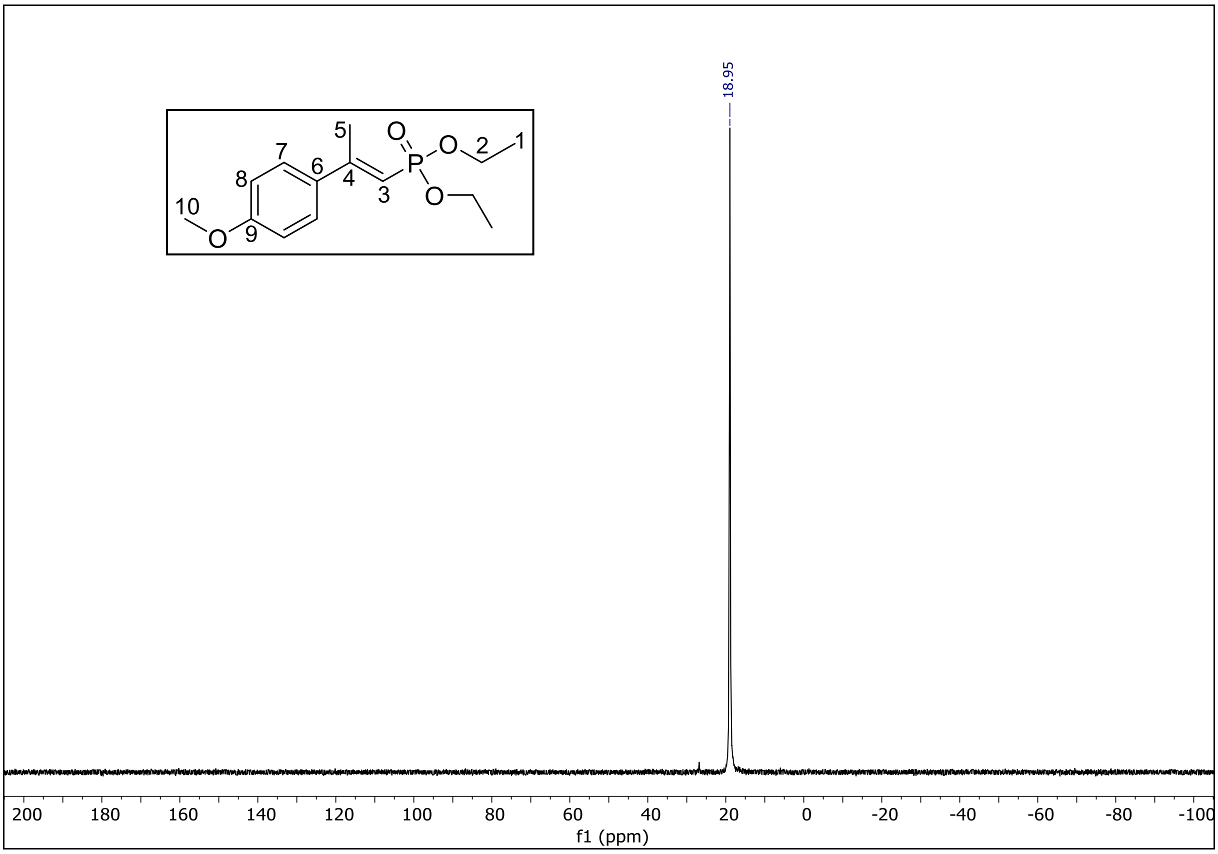

^{1}H NMR (500 MHz, $CDCl_3$): ***E*-13**

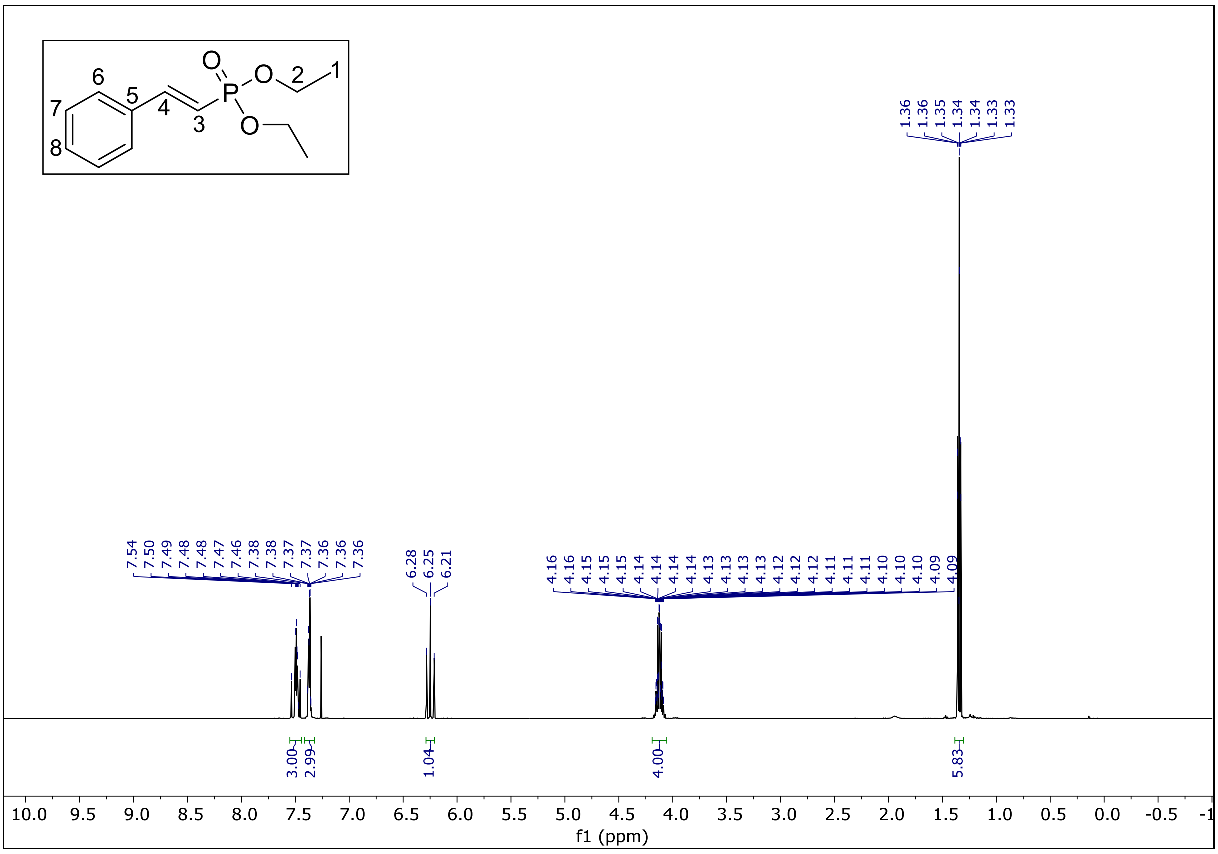

^{13}C NMR (126 MHz, $CDCl_3$): ***E*-13**

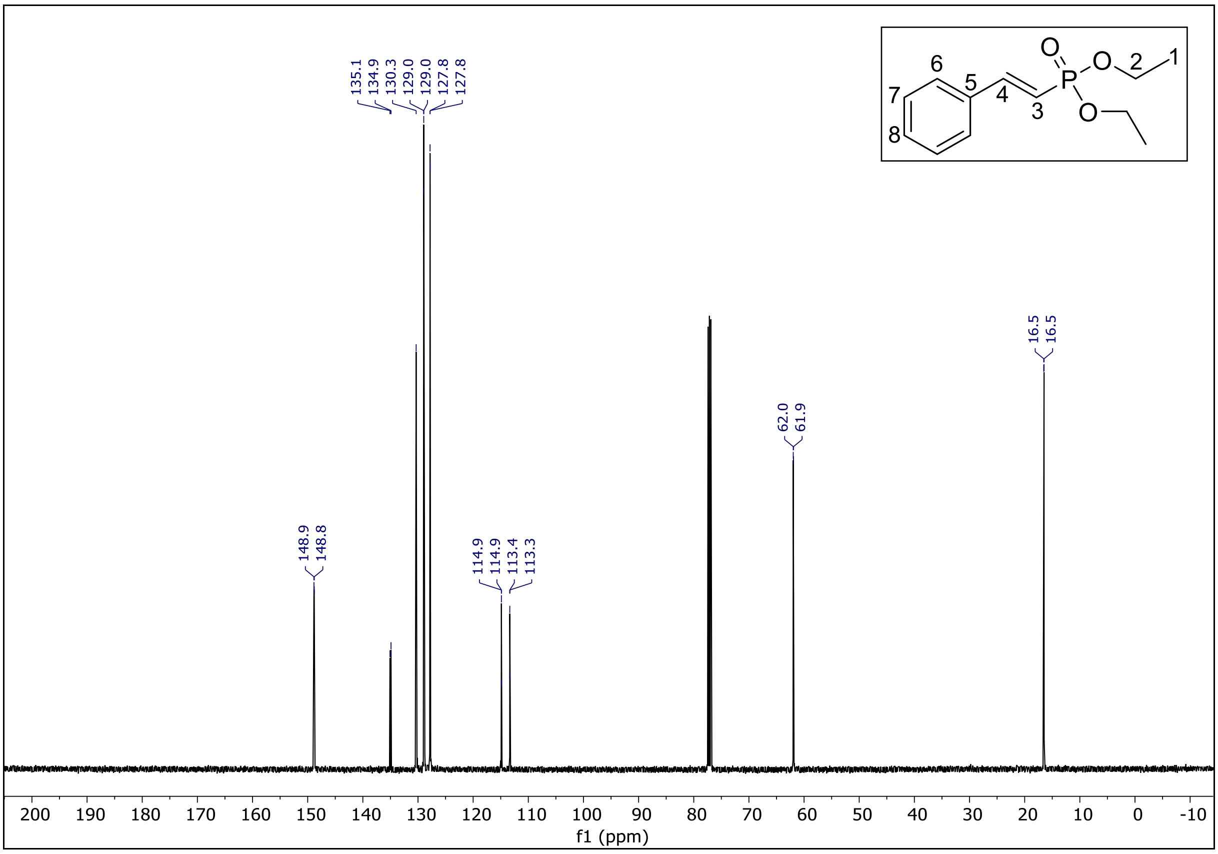

^{31}P NMR (202 MHz, $CDCl_3$): ***E*-13**

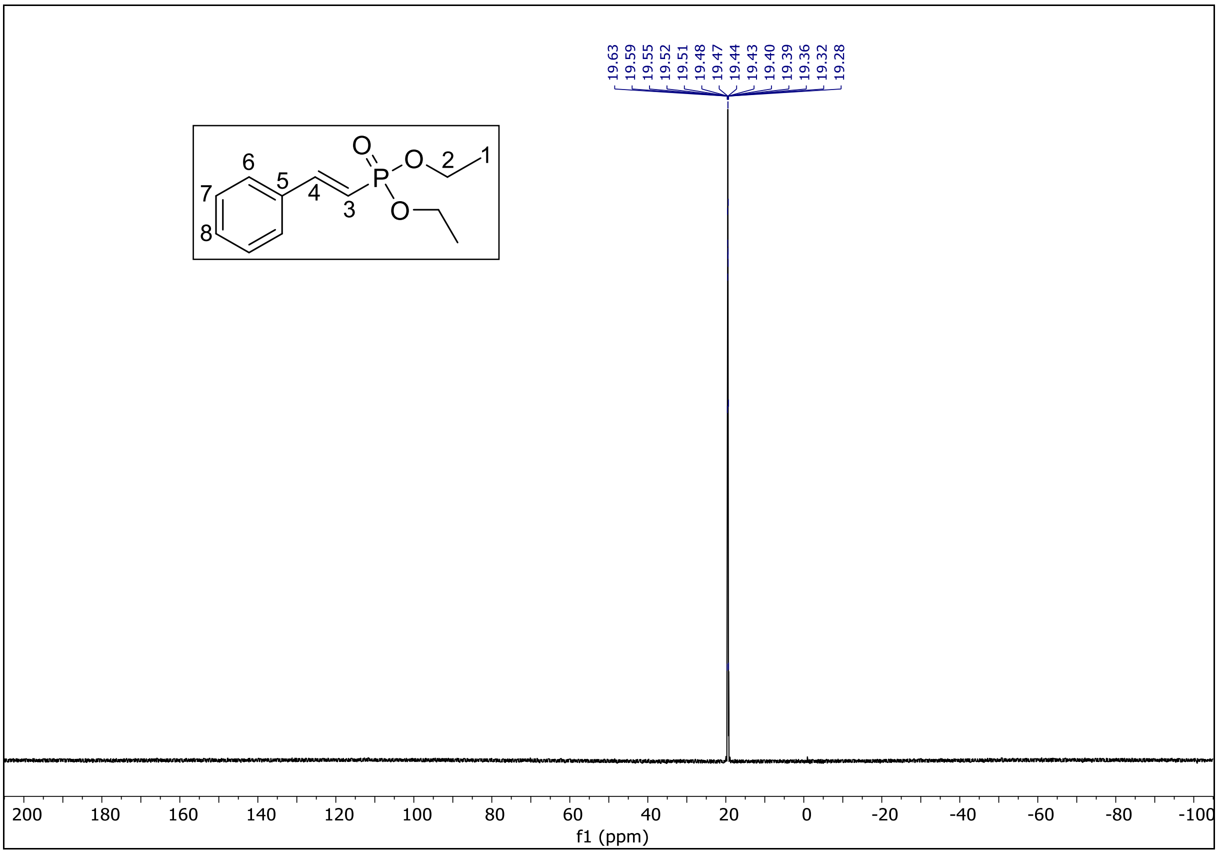

^{1}H NMR (500 MHz, $CDCl_3$): ***E*-14**

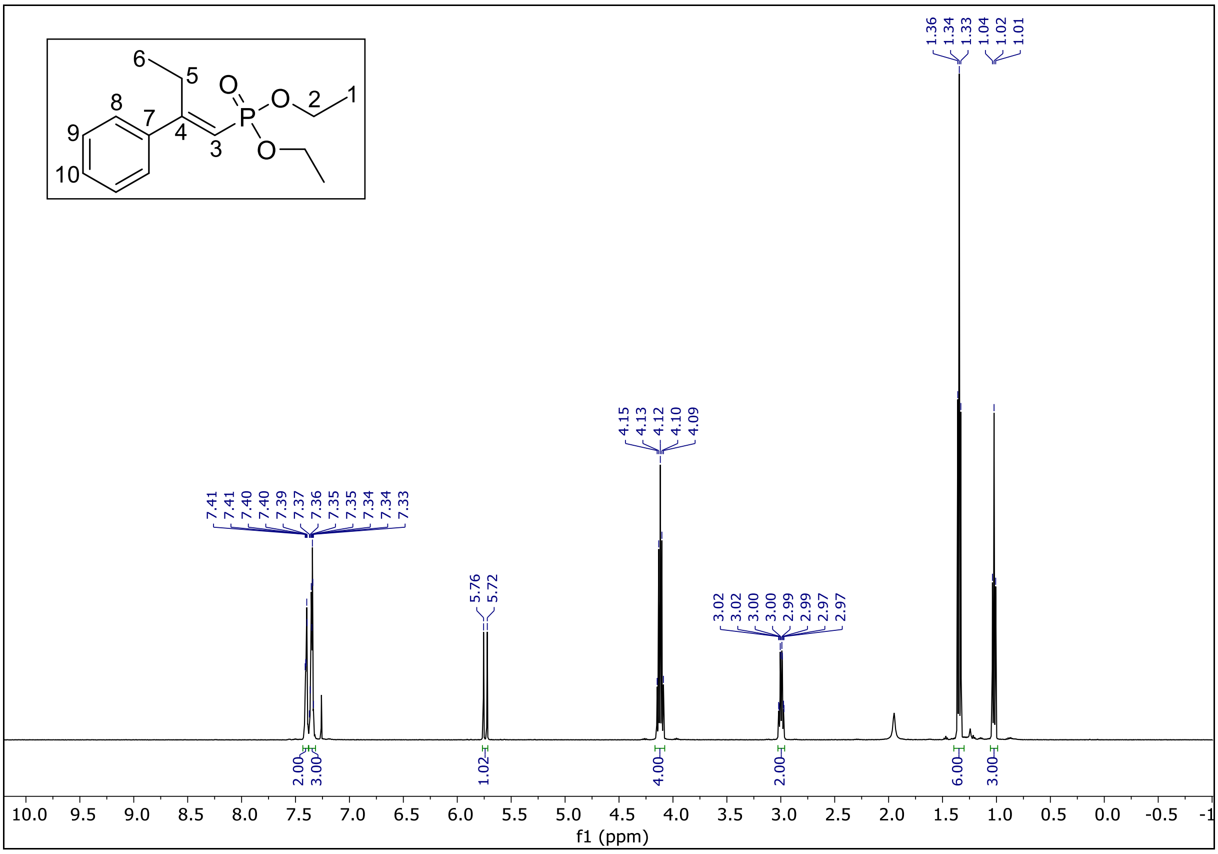

^{13}C NMR (126 MHz, $CDCl_3$): ***E*-14**

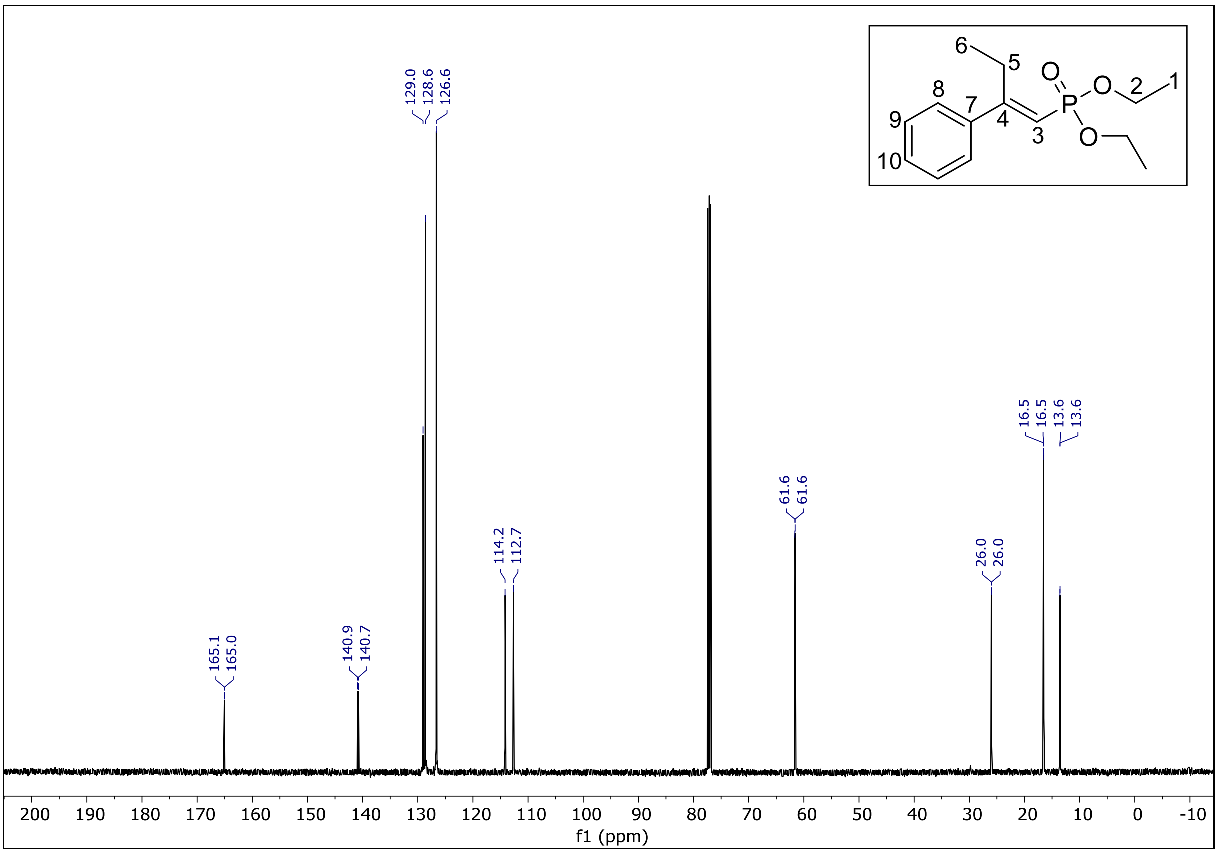

^{31}P NMR (202 MHz, $CDCl_3$): ***E*-14**

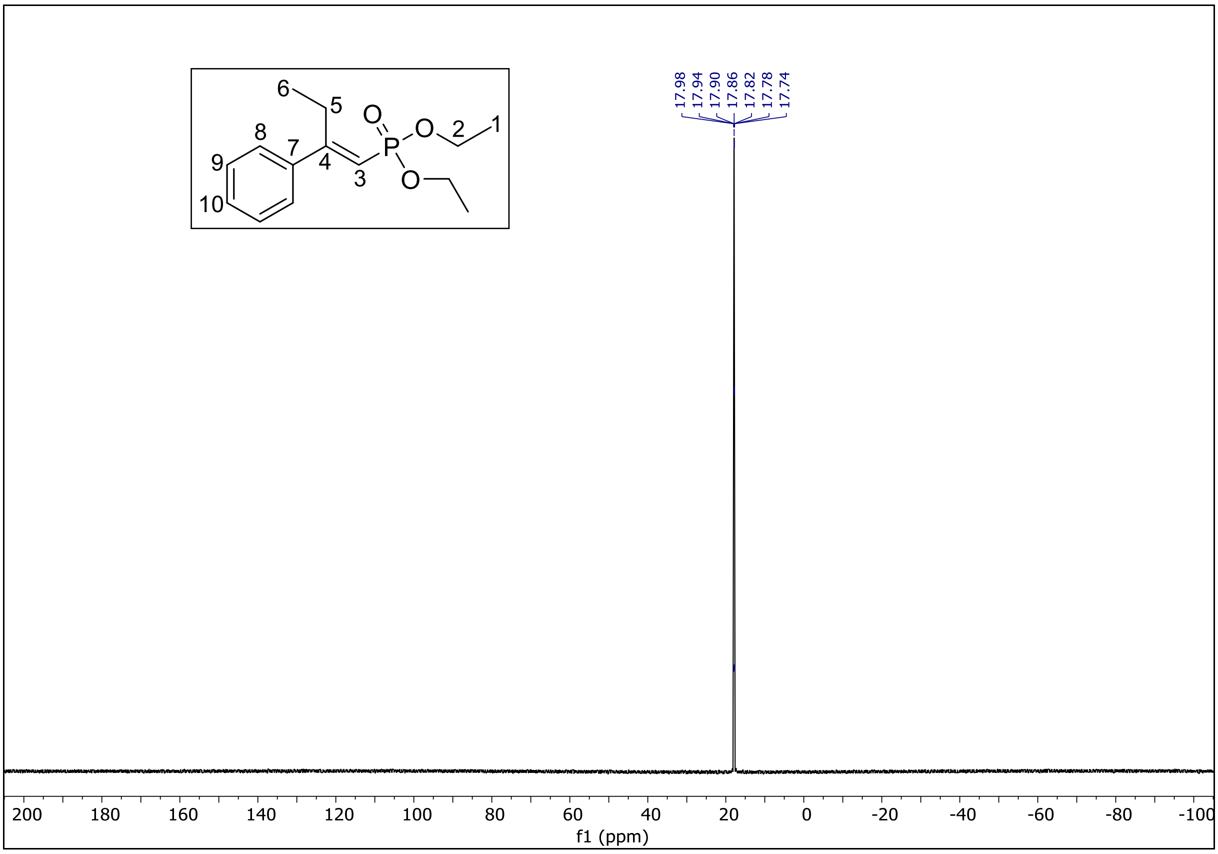

^{1}H NMR (600 MHz, $CDCl_3$): ***E*-15**

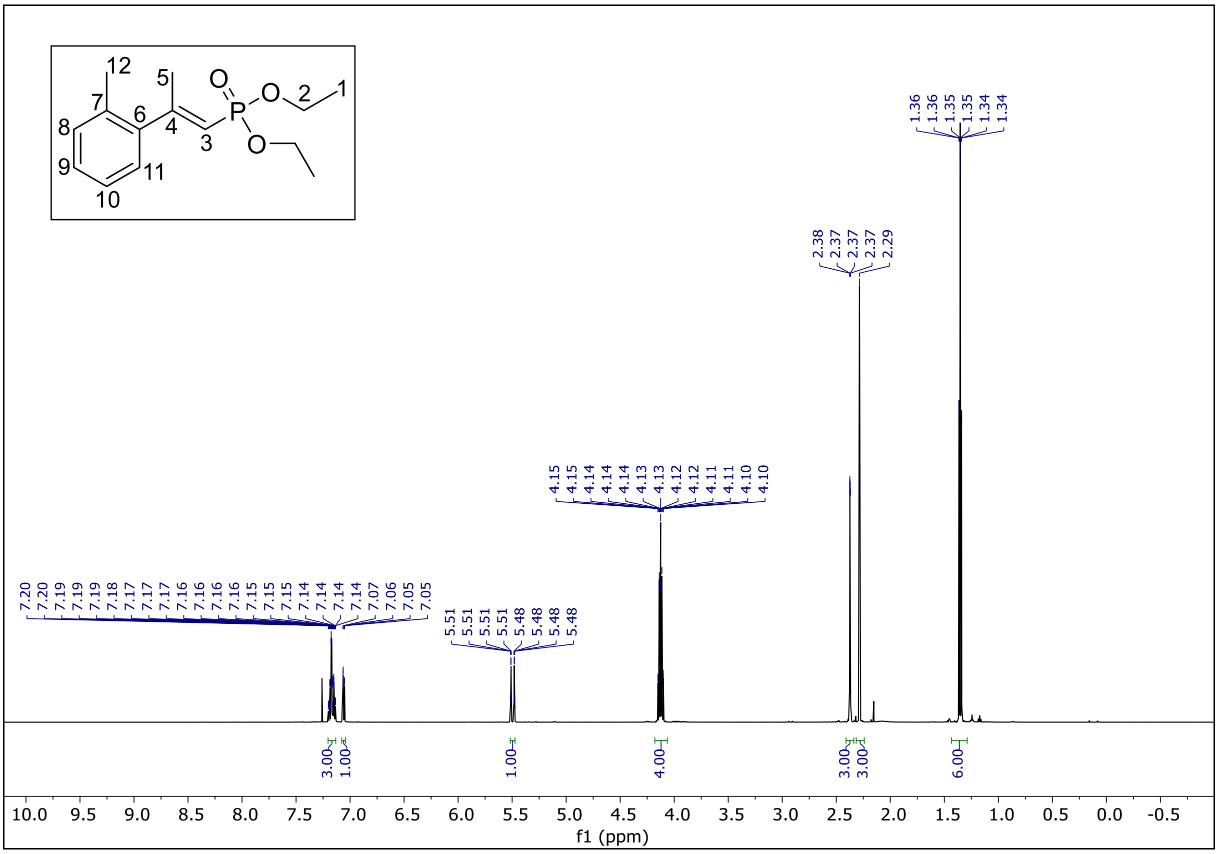

^{13}C NMR (151 MHz, $CDCl_3$): ***E*-15**

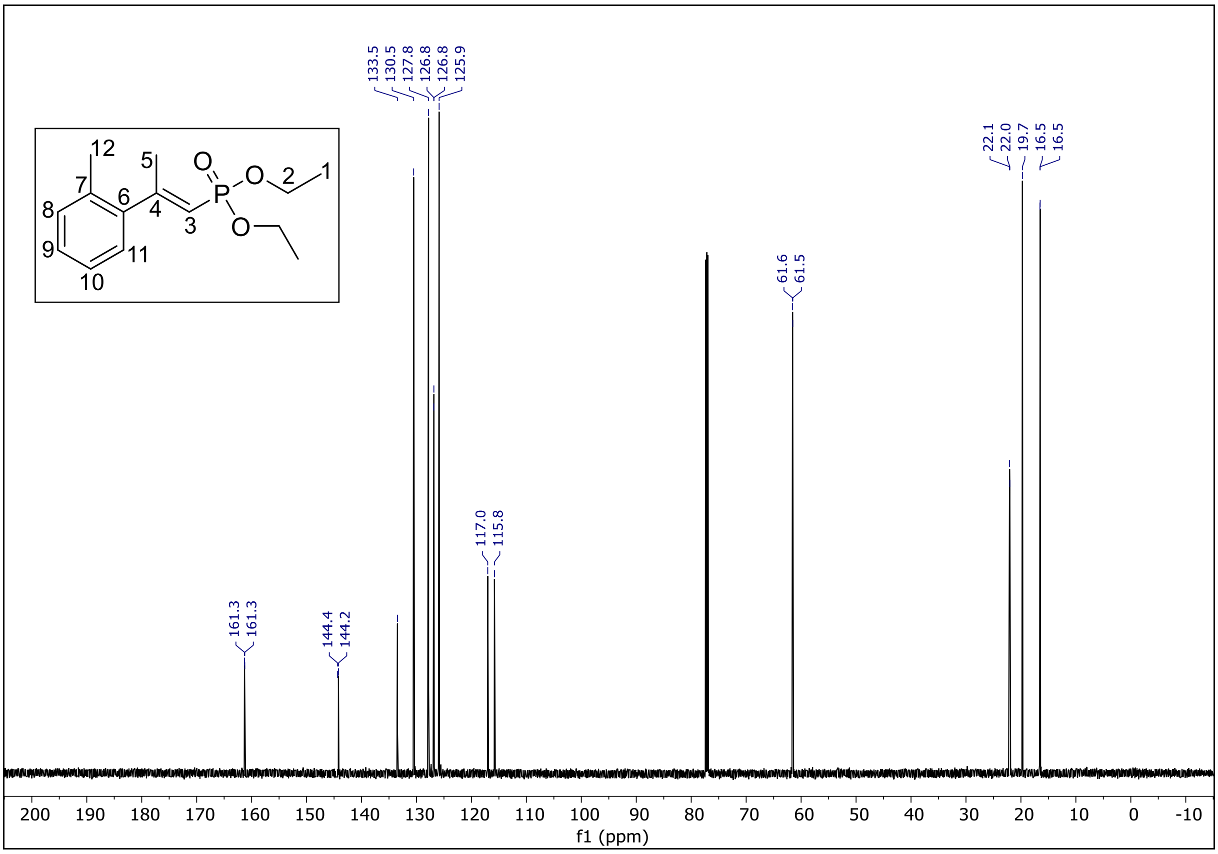

^{31}P NMR (121 MHz, CDCl$_3$): ***E*-15**

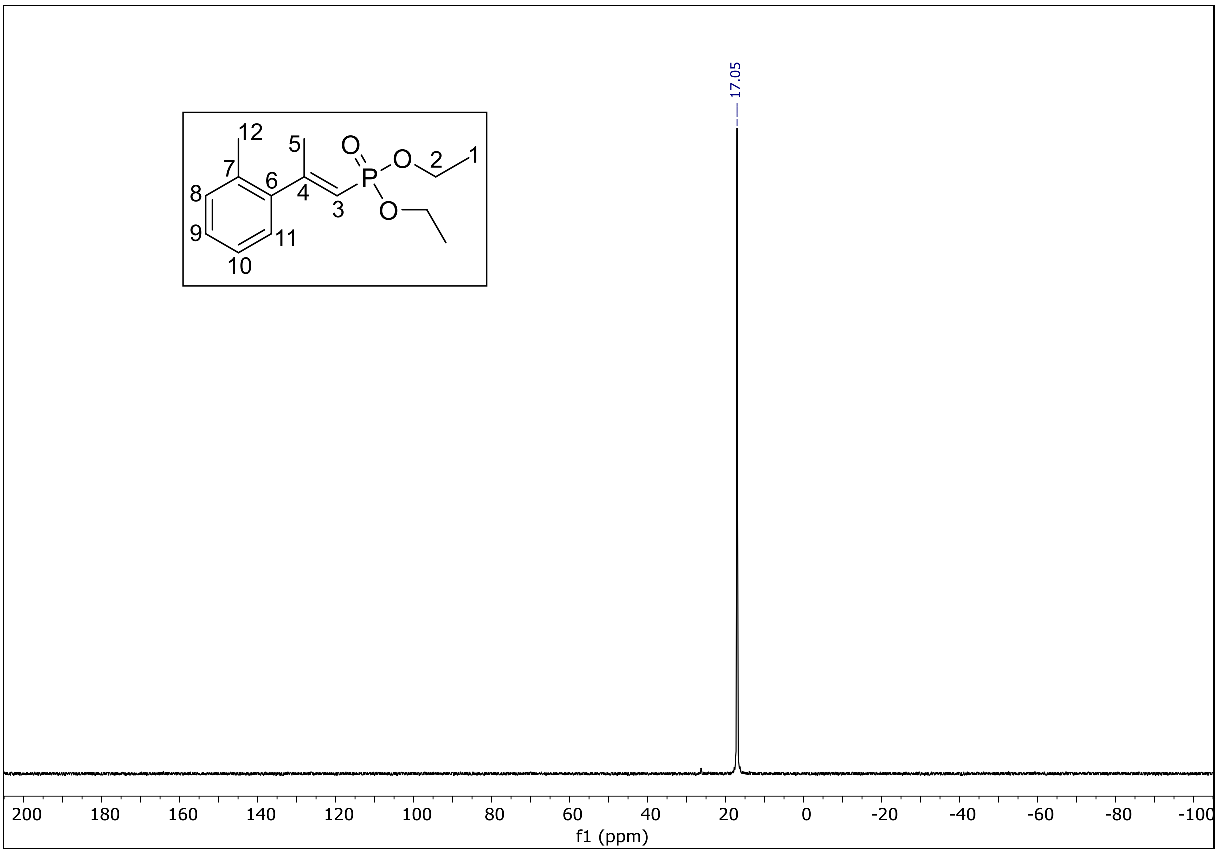

^{1}H NMR (600 MHz, CDCl$_3$): ***E*-16**

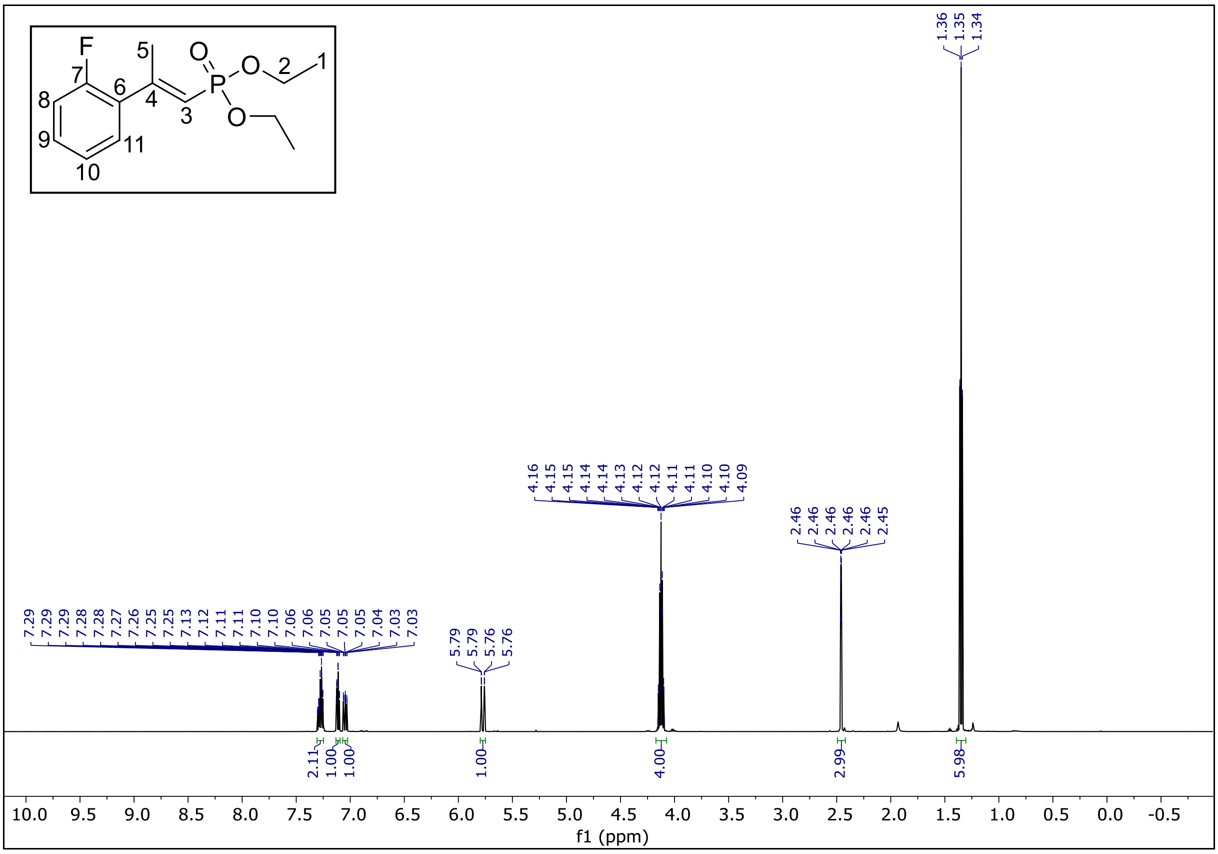

^{13}C NMR (151 MHz, $CDCl_3$): ***E*-16**

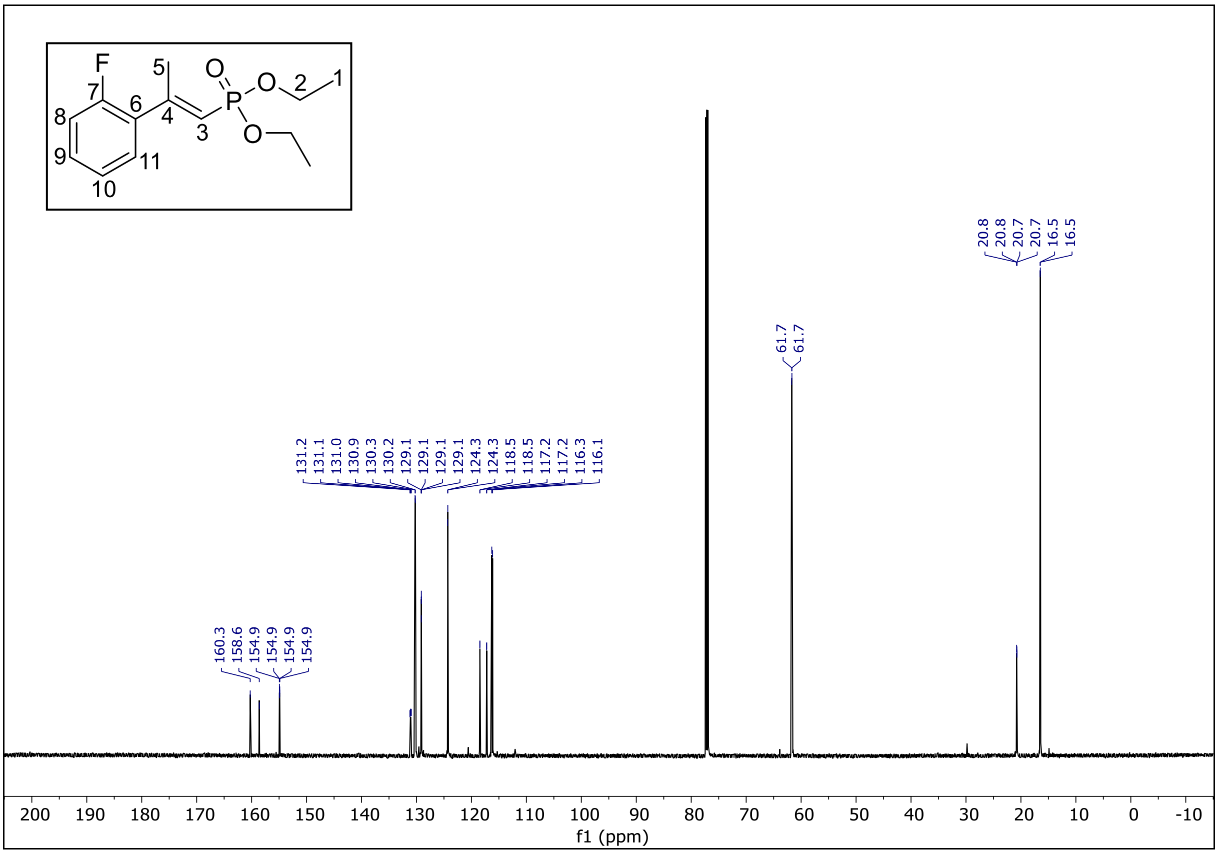

^{19}F NMR (564 MHz, $CDCl_3$): ***E*-16**

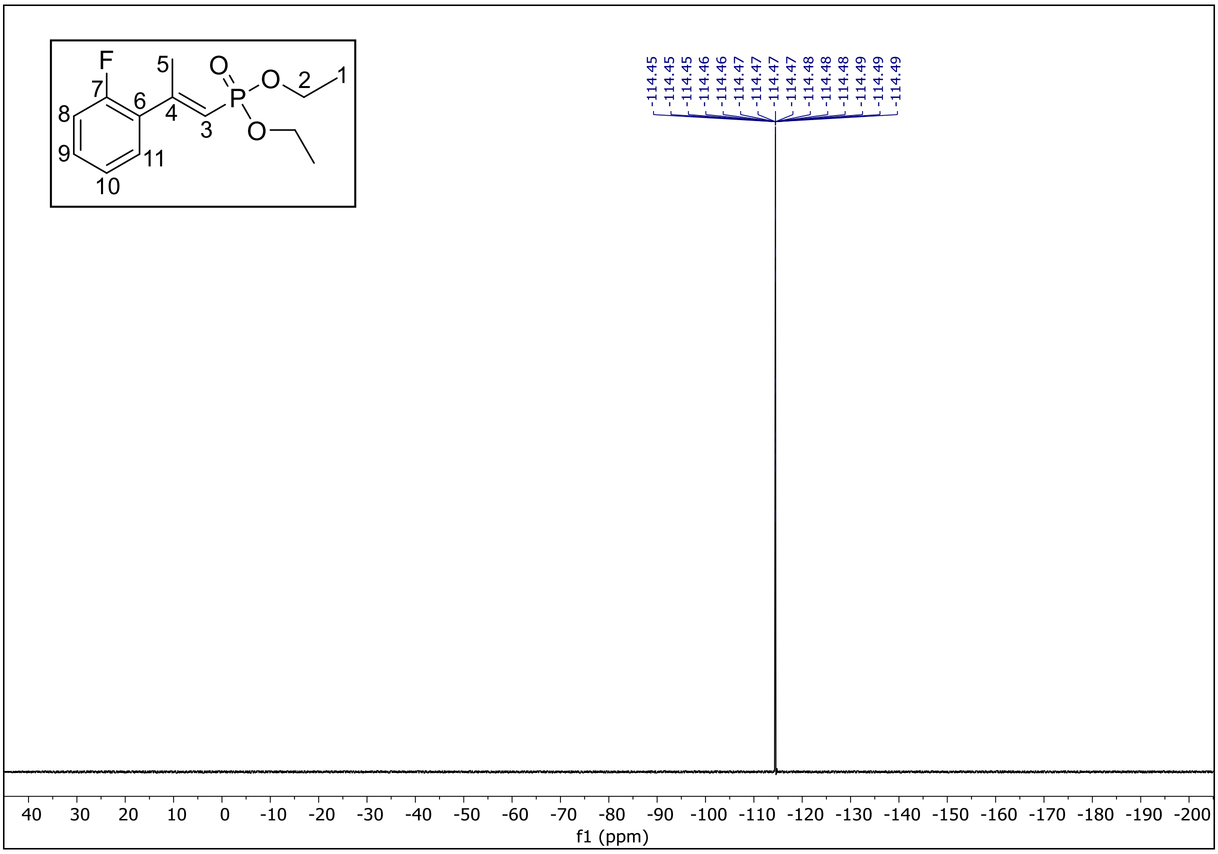

^{31}P NMR (243 MHz, $CDCl_3$): ***E*-16**

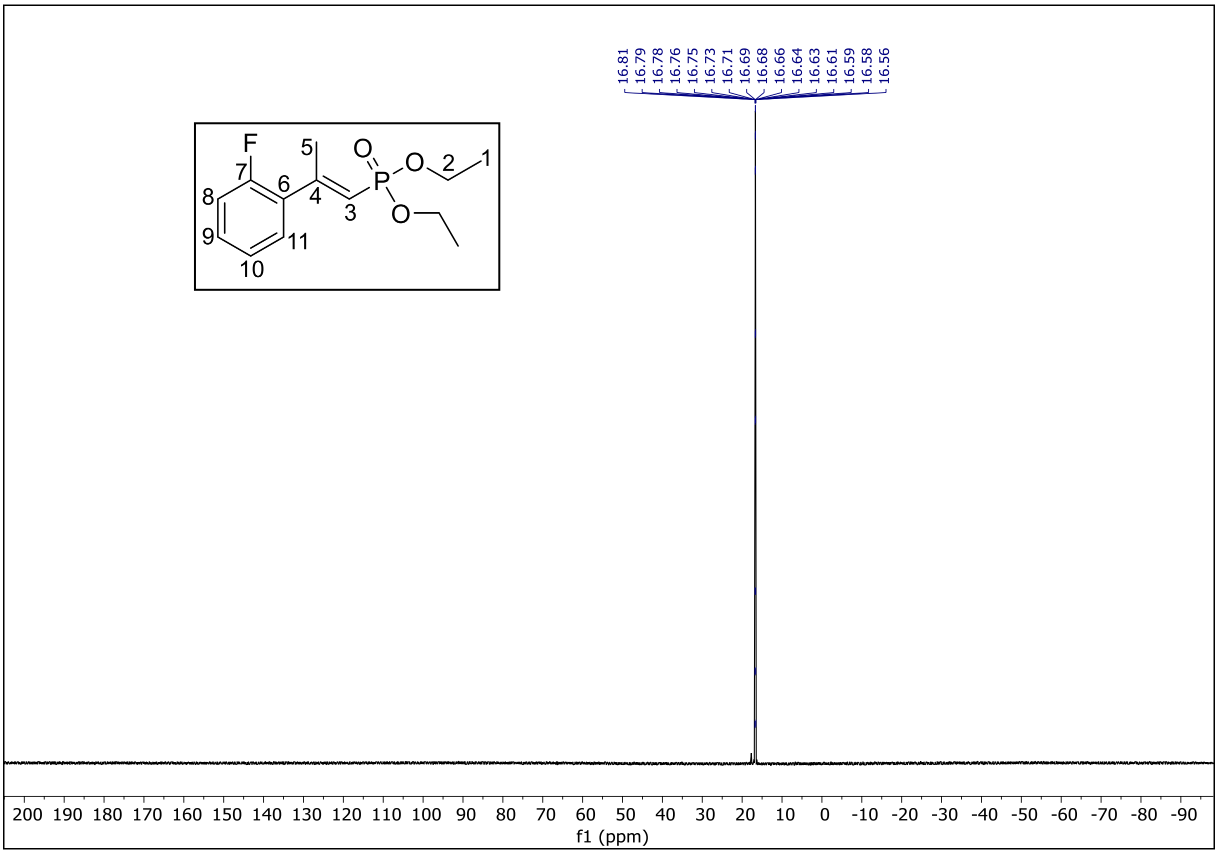

^{1}H NMR (600 MHz, $CDCl_3$): ***E*-17**

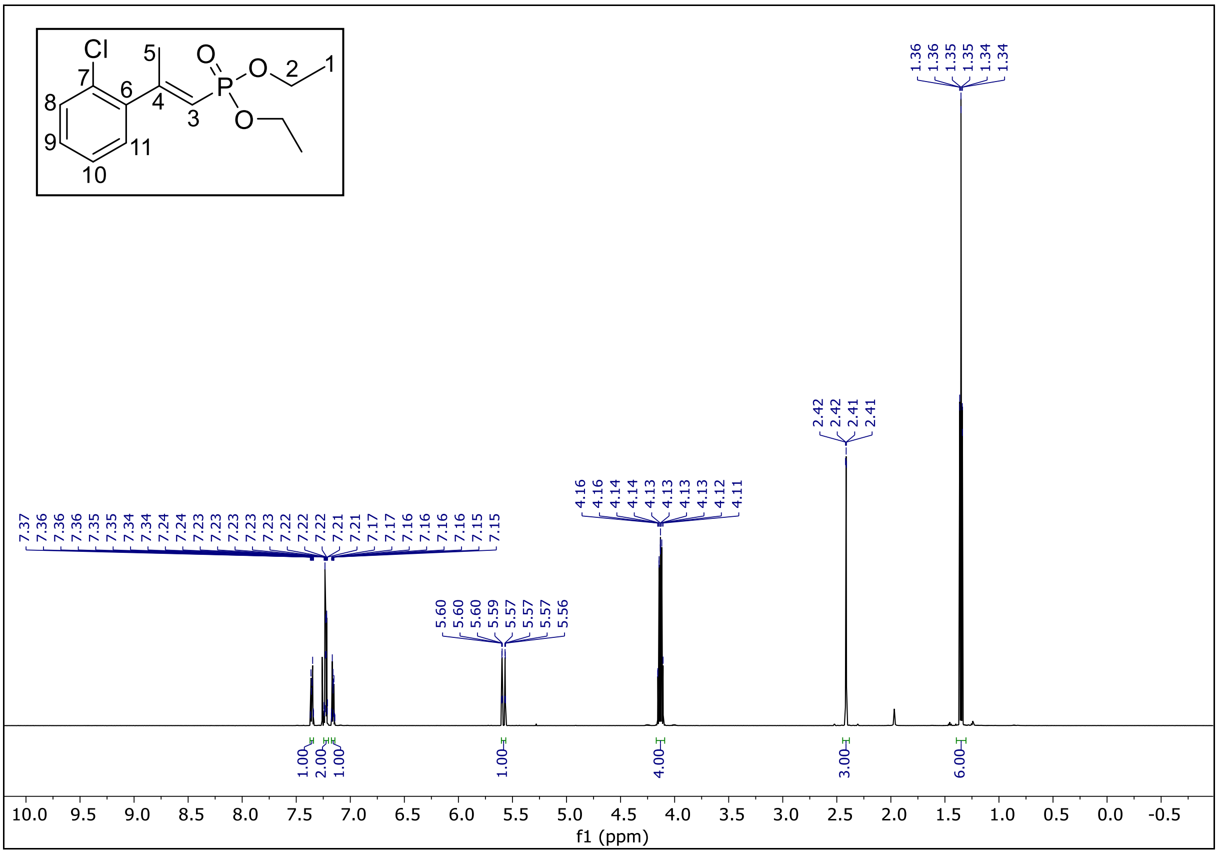

^{13}C NMR (151 MHz, $CDCl_3$): ***E*-17**

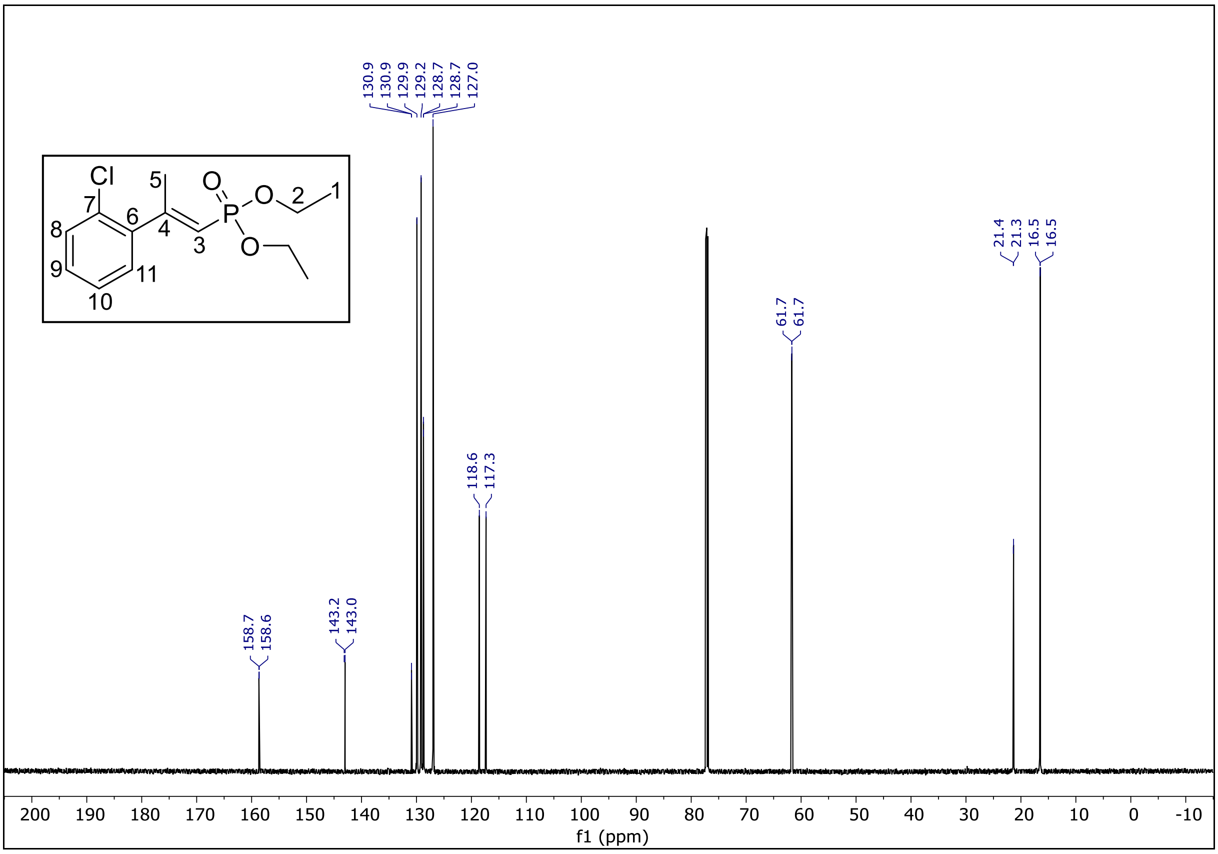

^{31}P NMR (243 MHz, $CDCl_3$): ***E*-17**

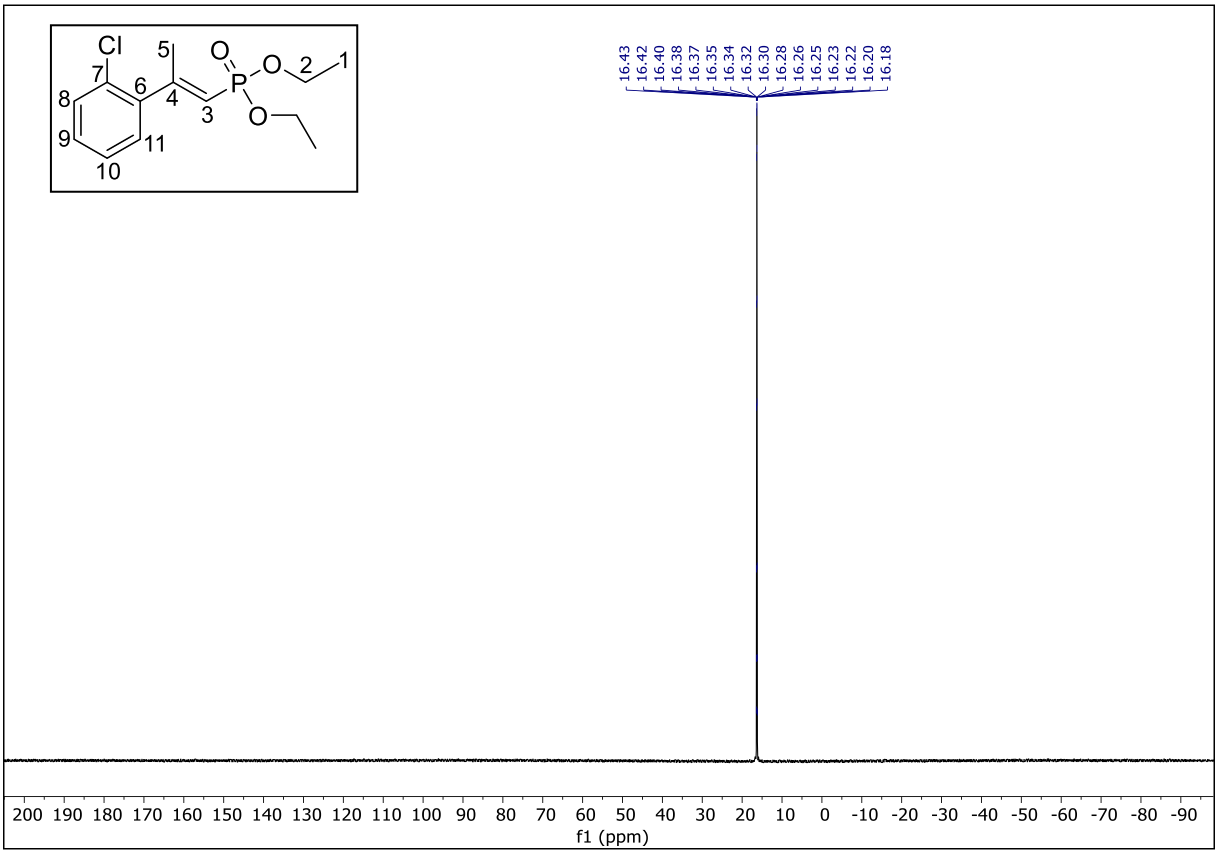

^{1}H NMR (600 MHz, $CDCl_3$): ***E*-18**

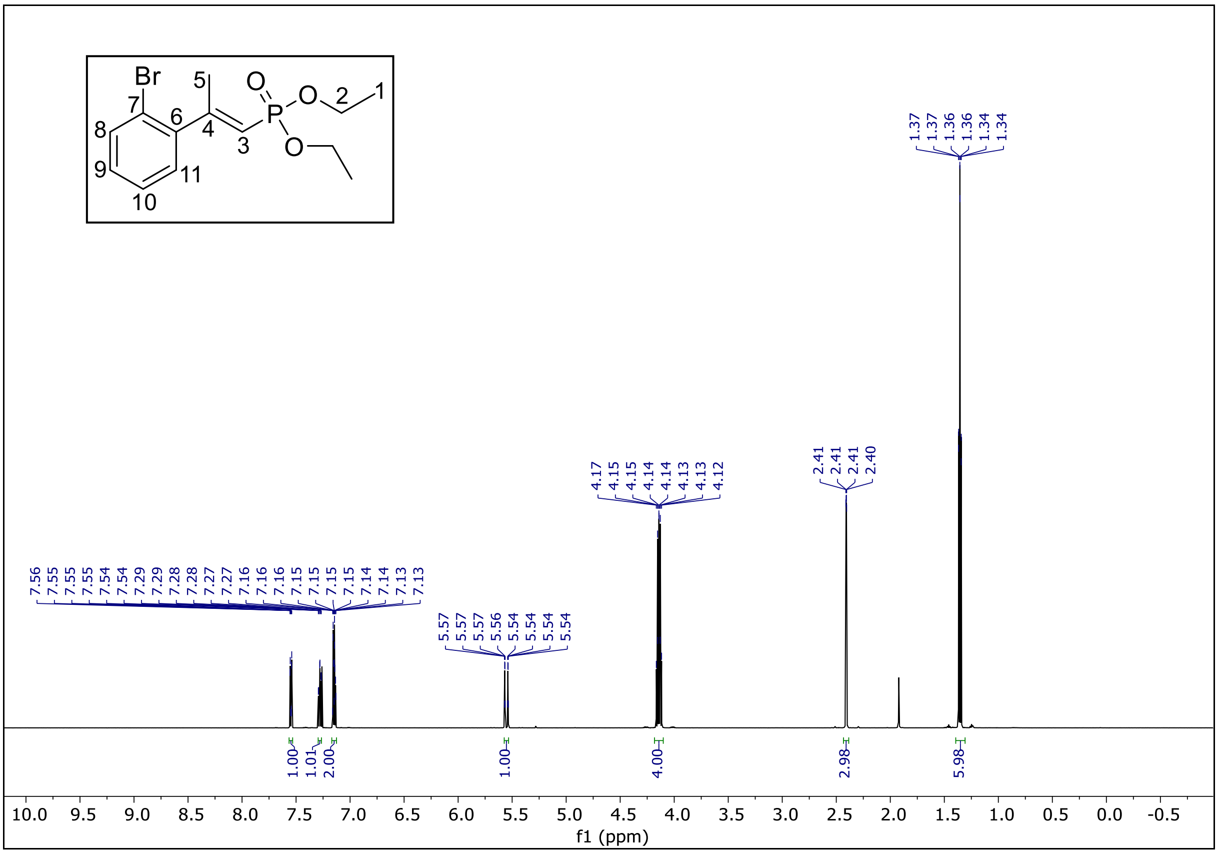

^{13}C NMR (151 MHz, $CDCl_3$): ***E*-18**

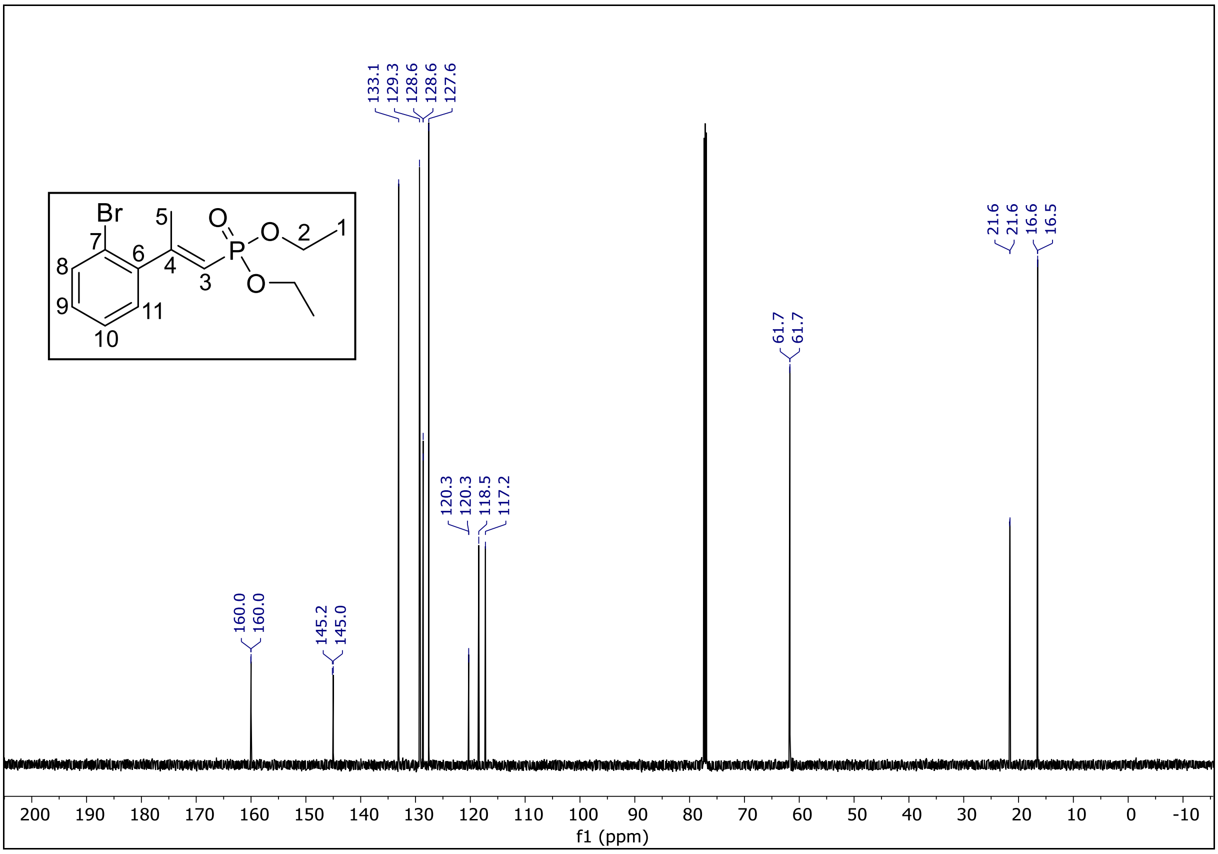

^{31}P NMR (243 MHz, $CDCl_3$): ***E*-18**

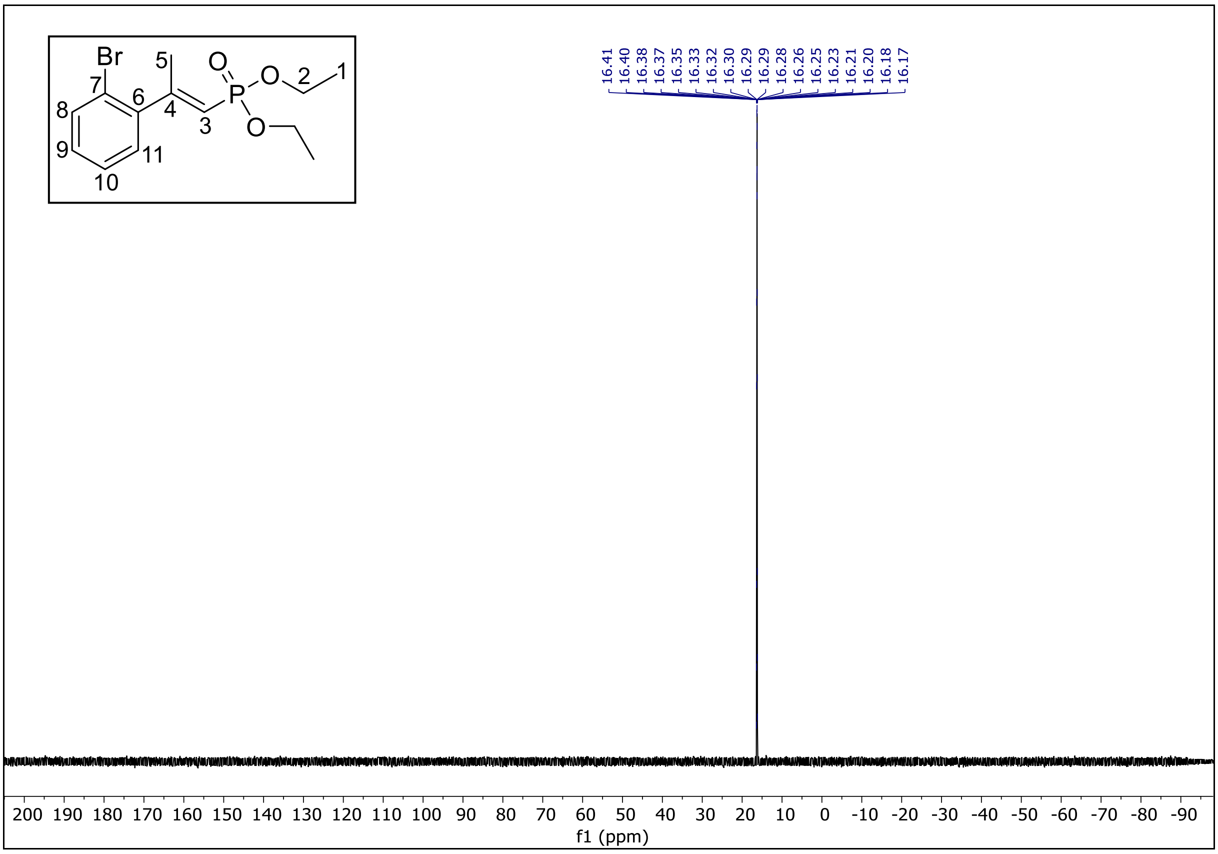

^{1}H NMR (600 MHz, $CDCl_3$): ***Z*-1**

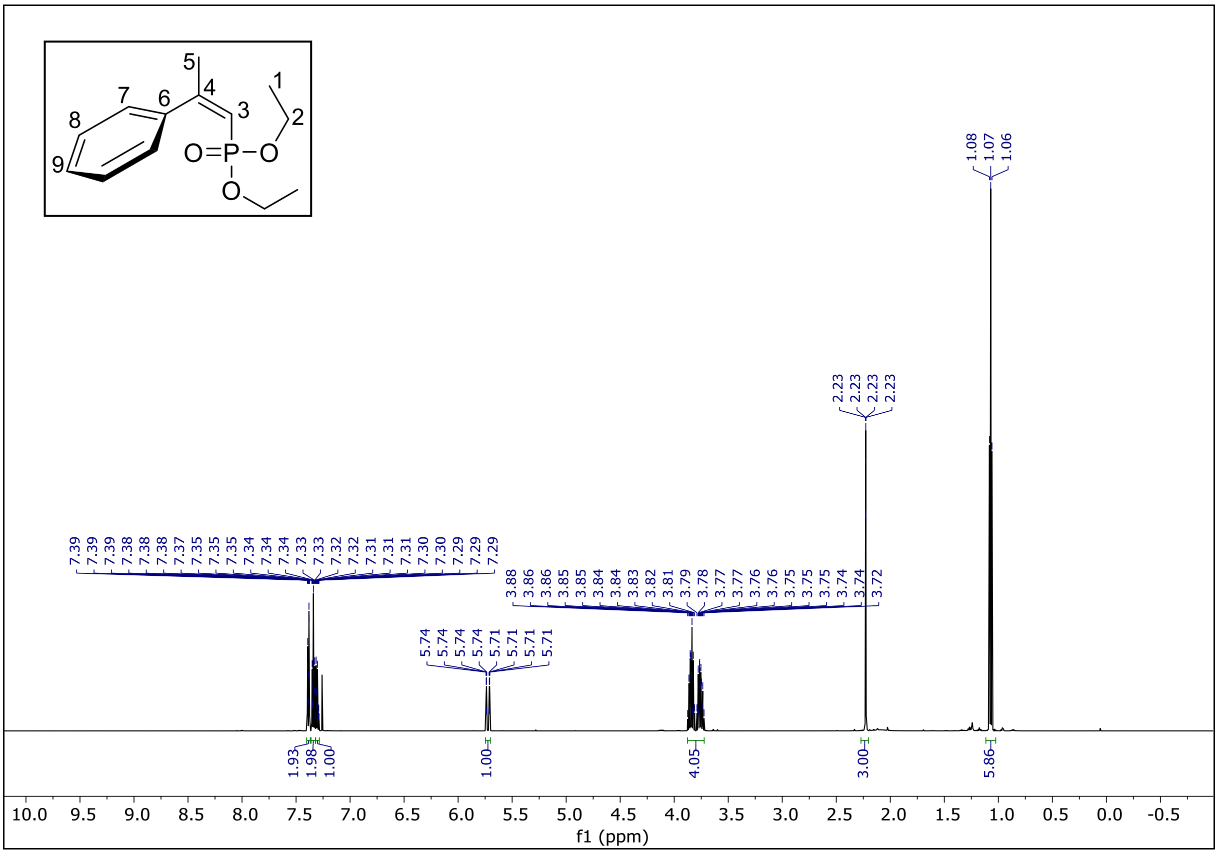

^{13}C NMR (151 MHz, $CDCl_3$): ***Z*-1**

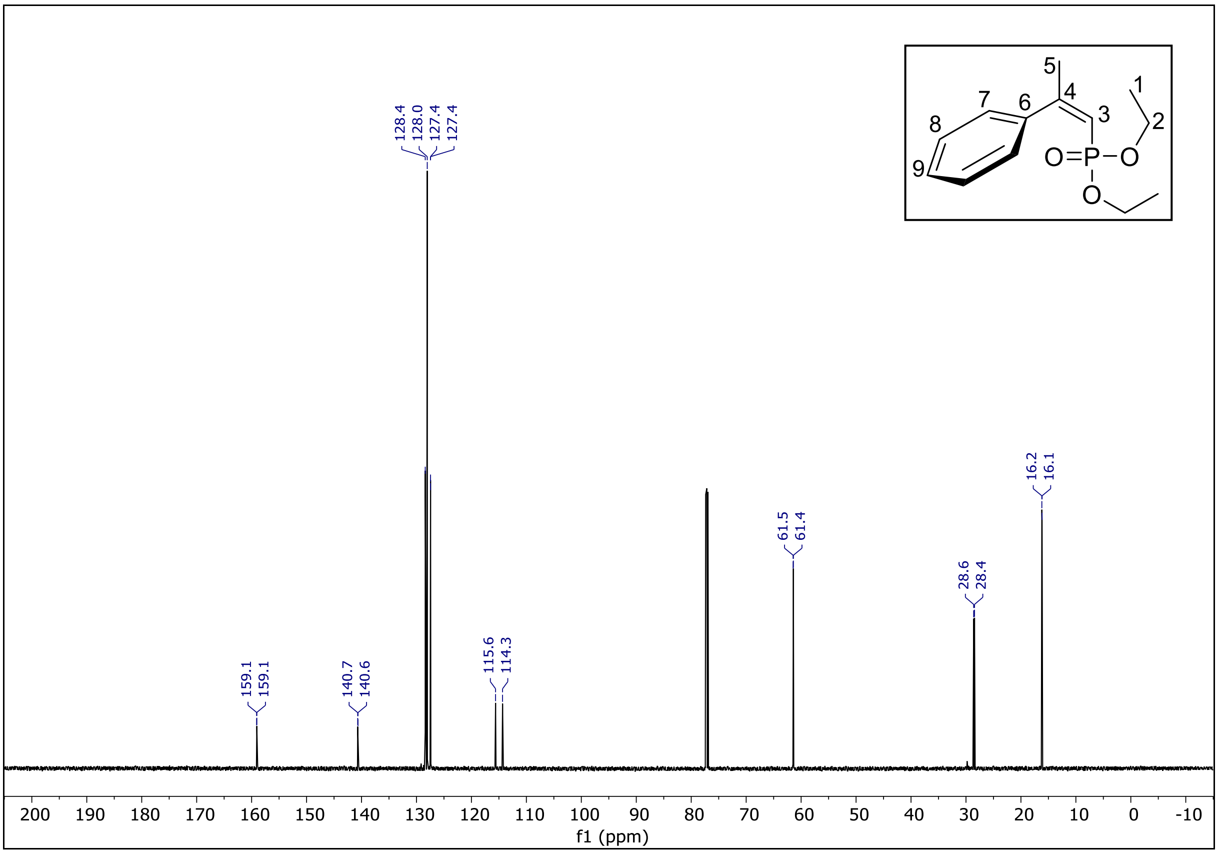

^{31}P NMR (121 MHz, $CDCl_3$): ***Z*-1**

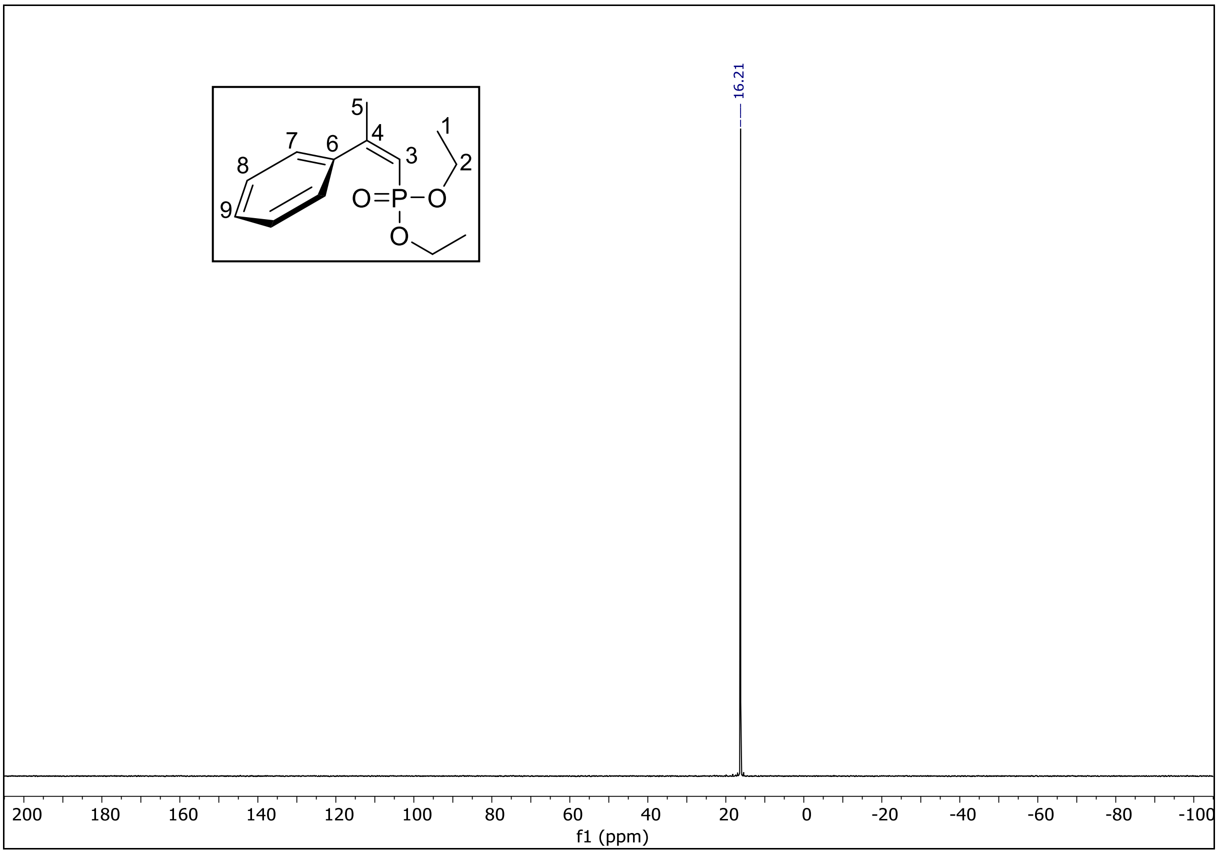

^{1}H NMR (500 MHz, $CDCl_3$): ***Z*-2**

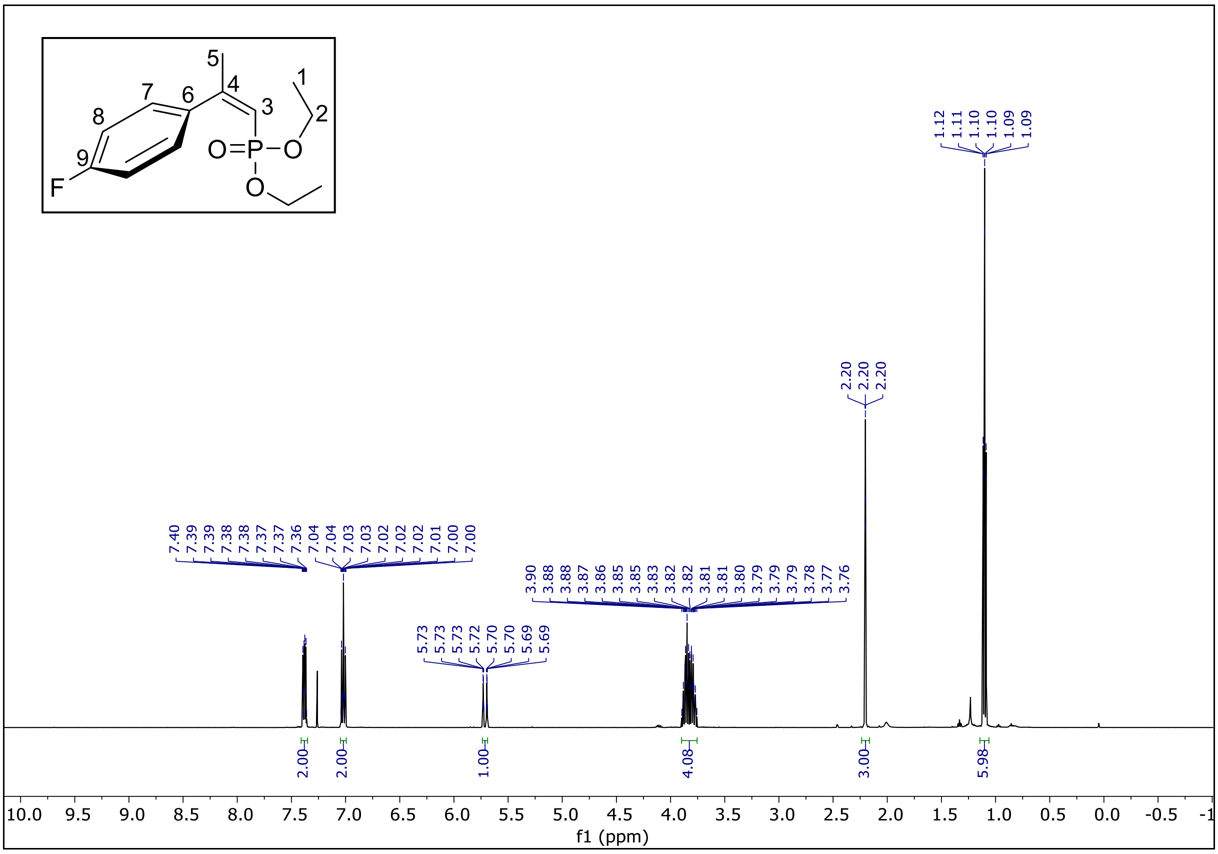

^{13}C NMR (126 MHz, $CDCl_3$): ***Z*-2**

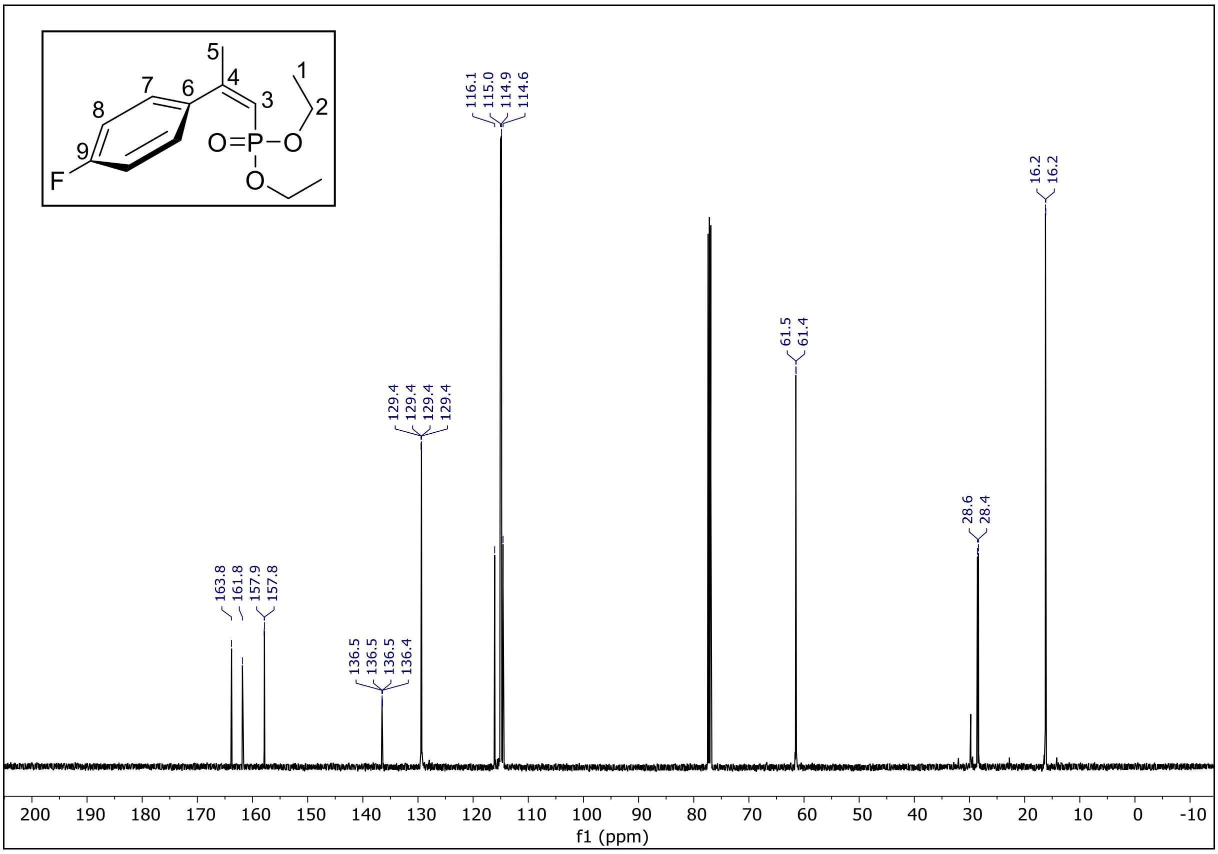

^{19}F NMR (470 MHz, $CDCl_3$): ***Z*-2**

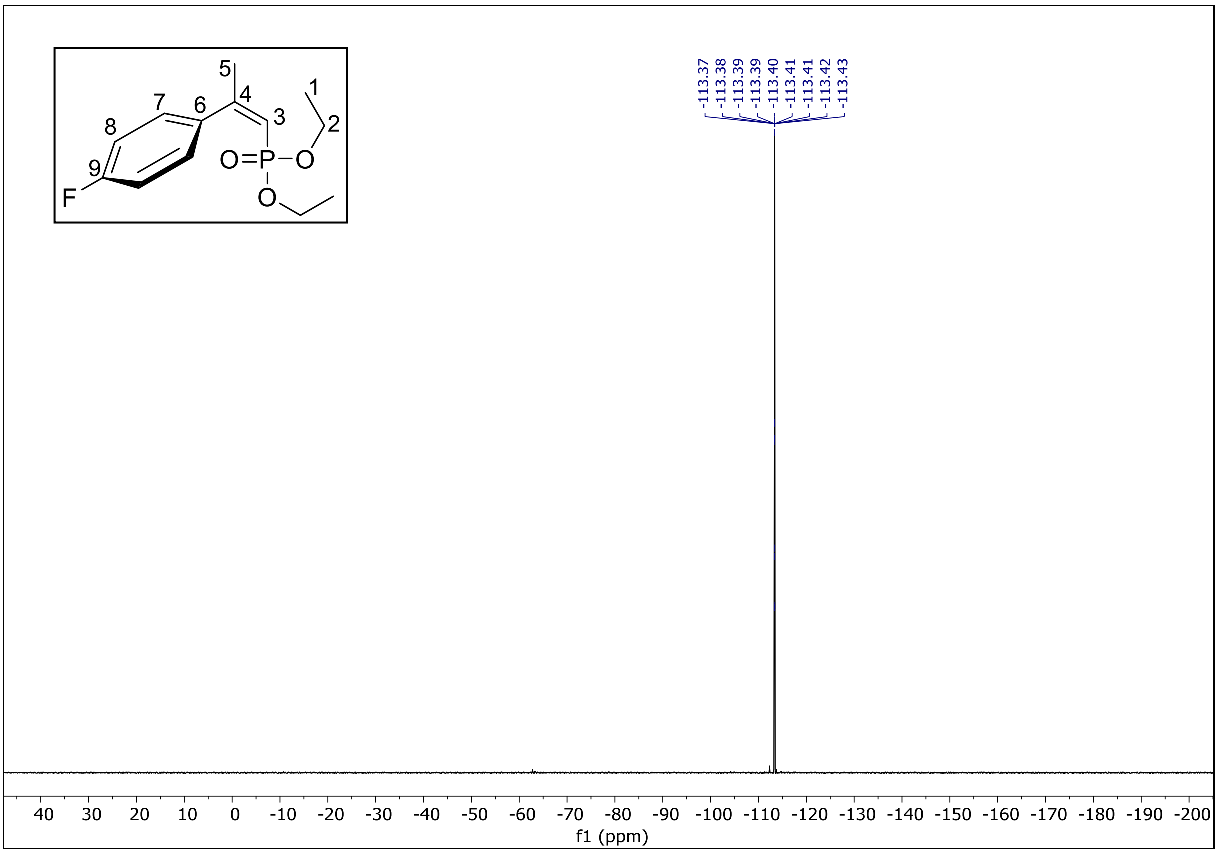

^{31}P NMR (202 MHz, $CDCl_3$): ***Z*-2**

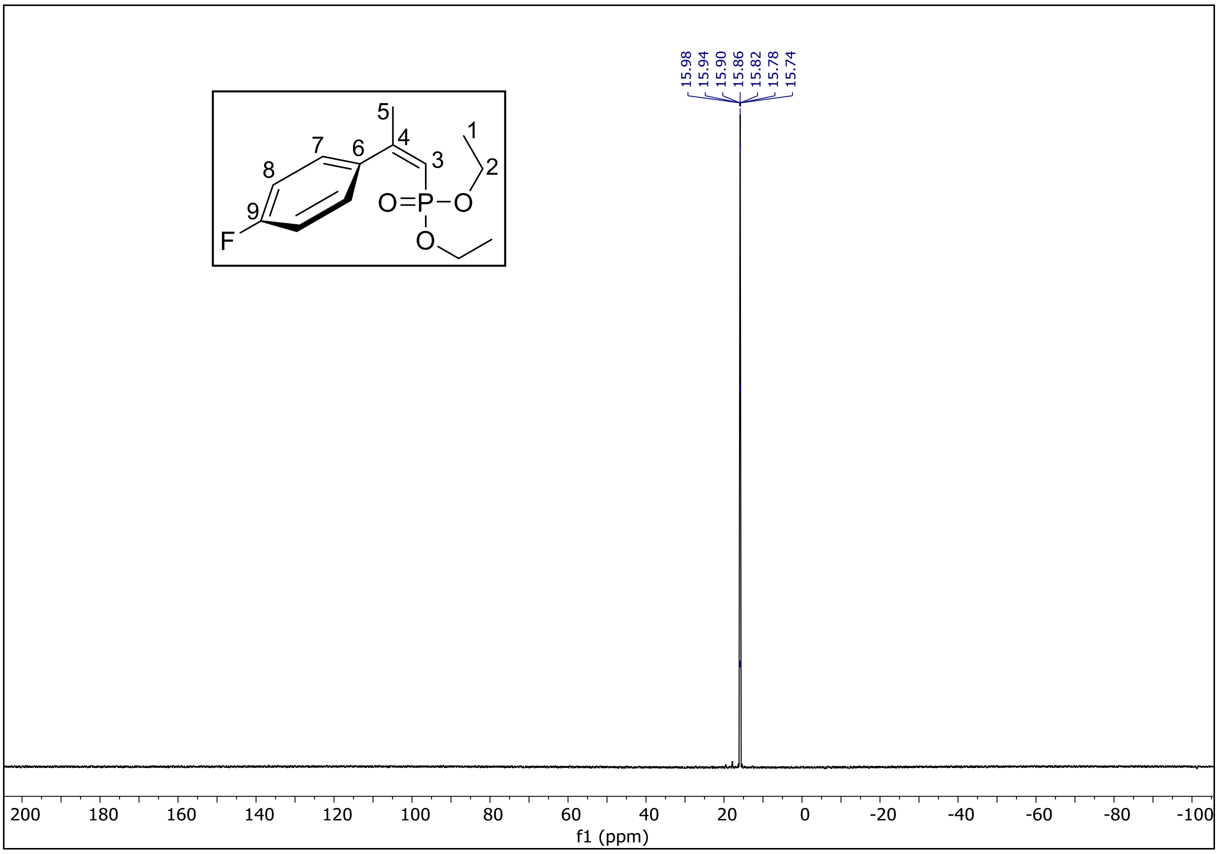

^{1}H NMR (500 MHz, $CDCl_3$): ***Z*-3**

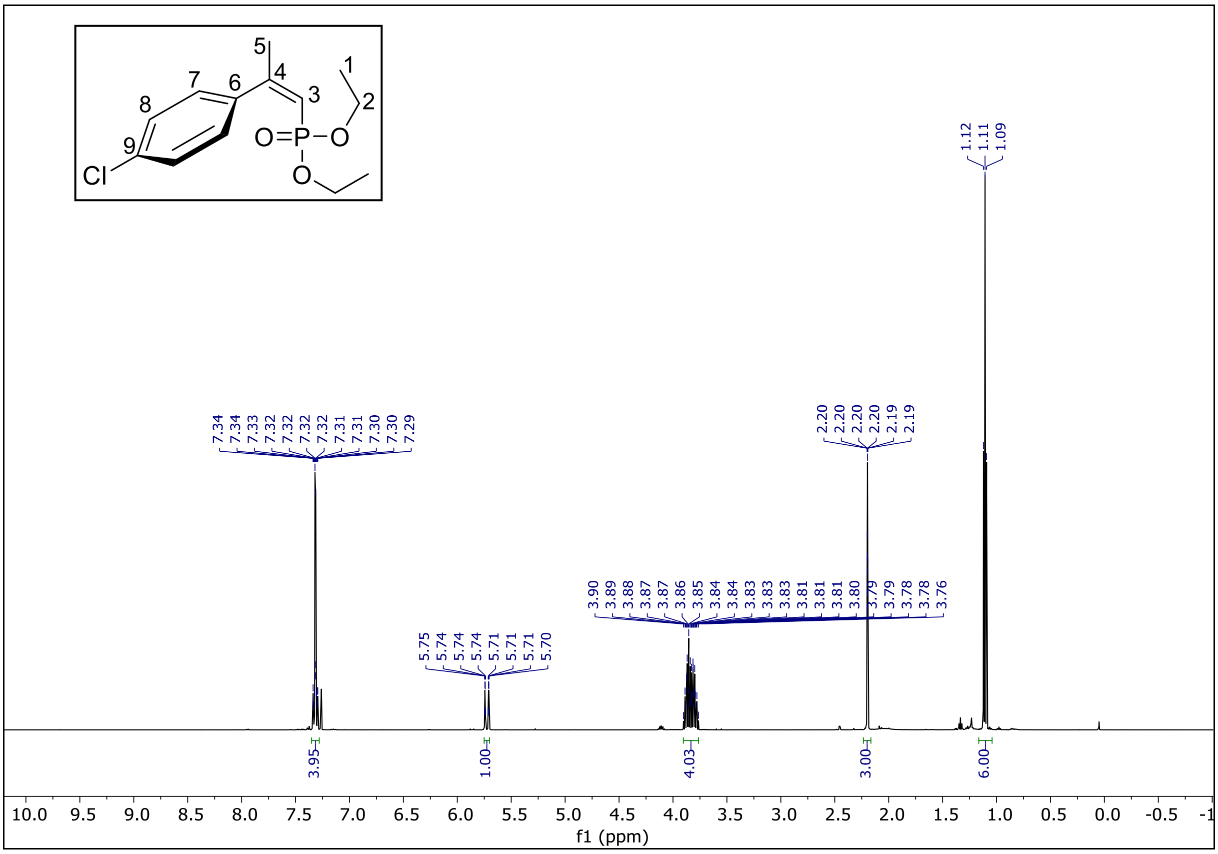

^{13}C NMR (101 MHz, $CDCl_3$): ***Z*-3**

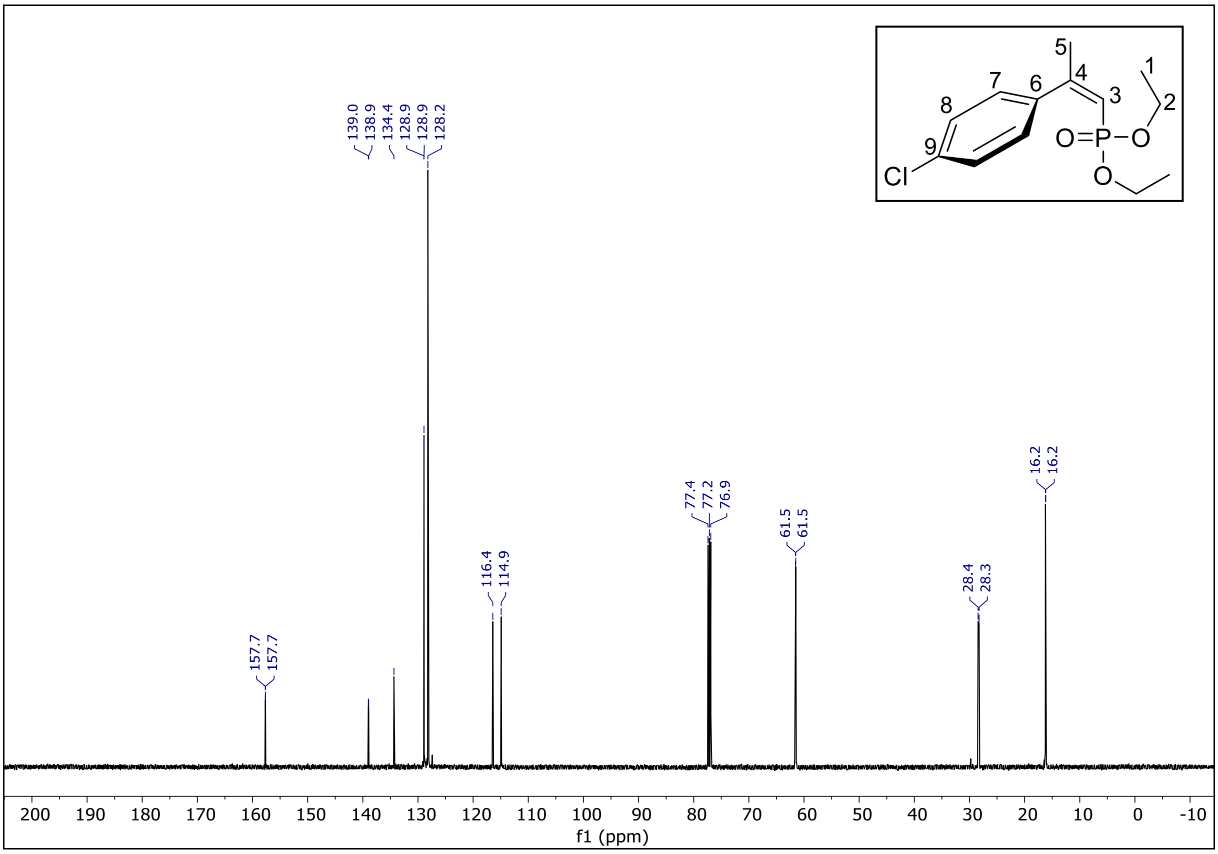

^{31}P NMR (202 MHz, $CDCl_3$): ***Z*-3**

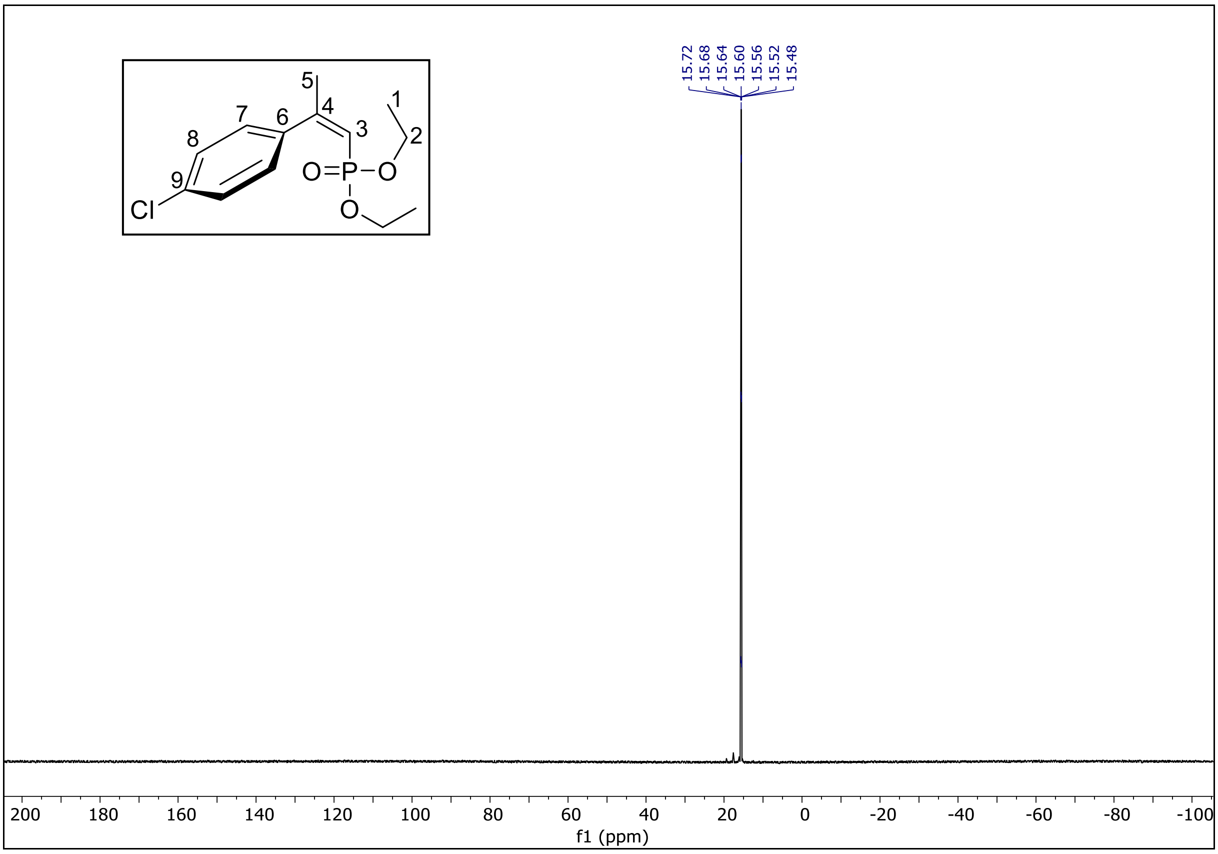

^{1}H NMR (600 MHz, $CDCl_3$): ***Z*-4**

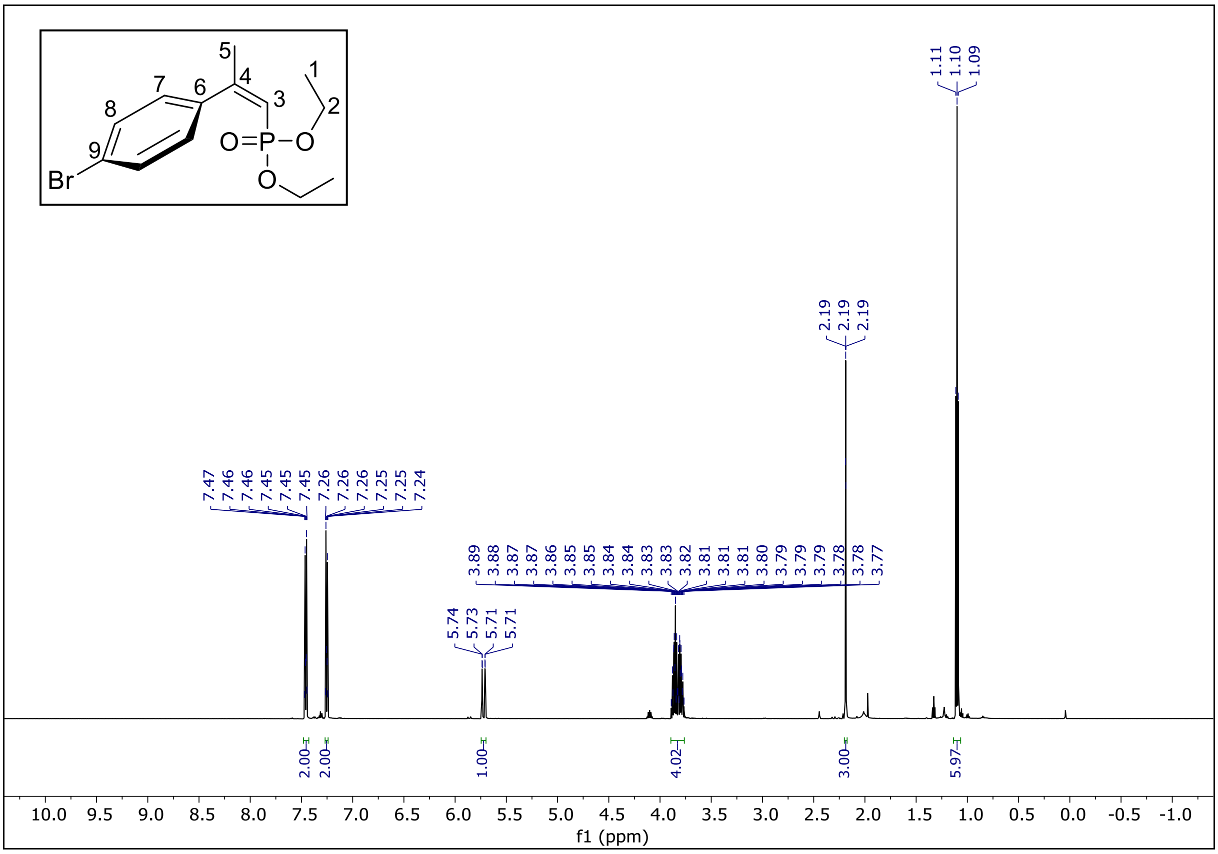

^{13}C NMR (151 MHz, $CDCl_3$): ***Z*-4**

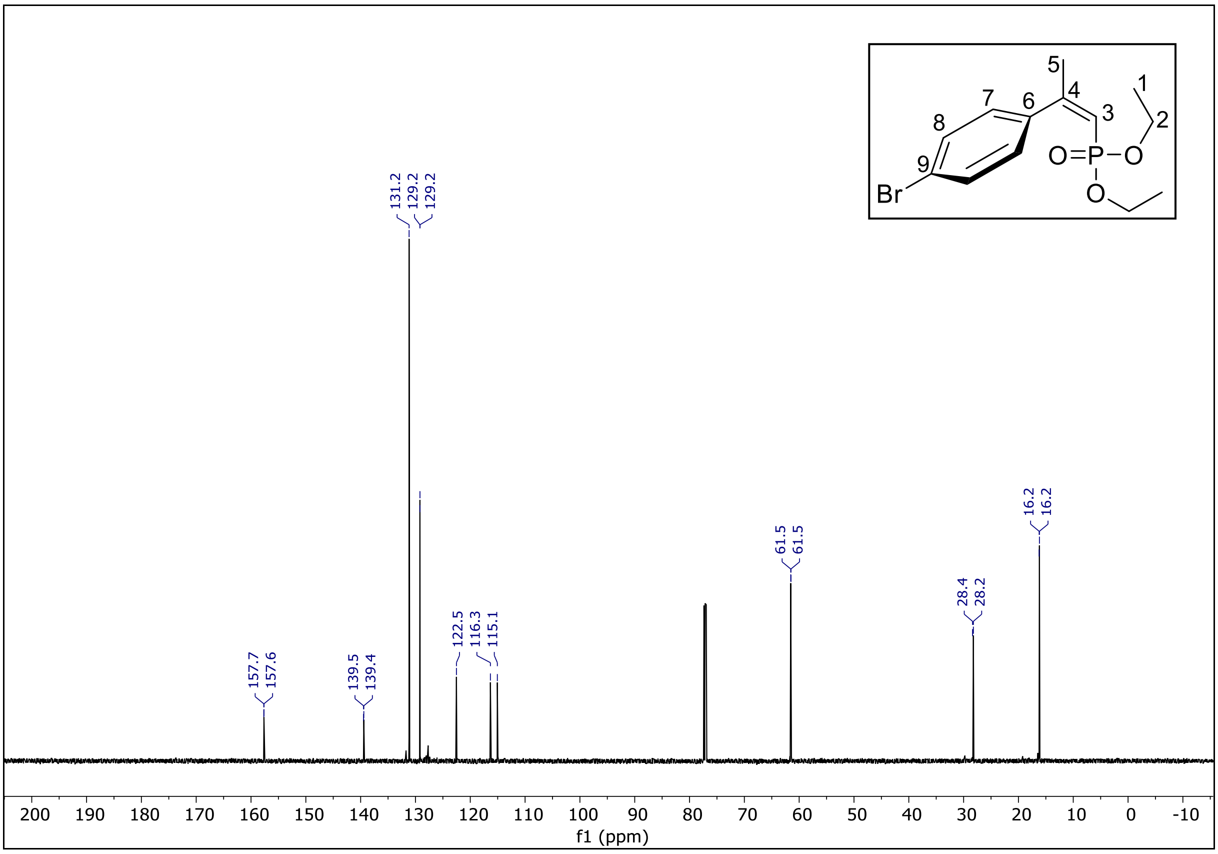

^{31}P NMR (243 MHz, $CDCl_3$): ***Z*-4**

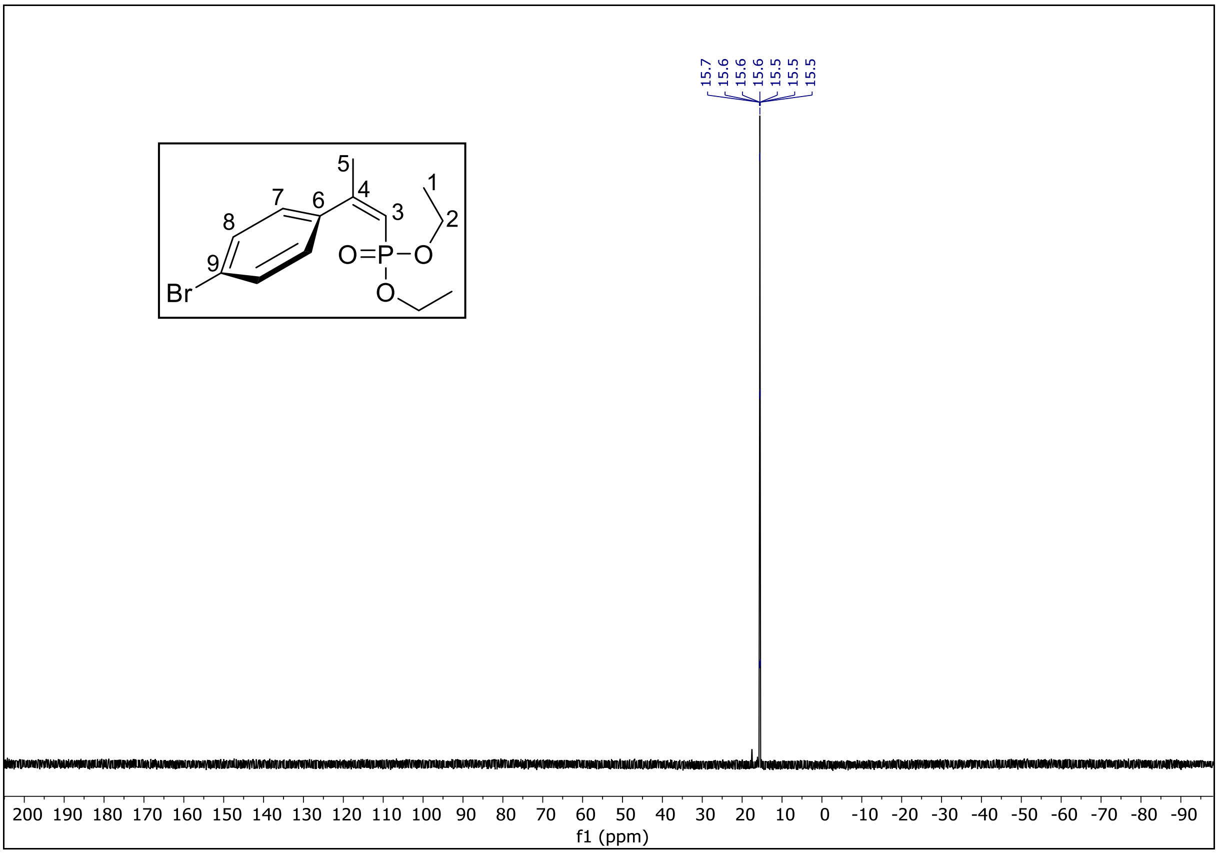

^{1}H NMR (600 MHz, $CDCl_3$): ***Z*-5**

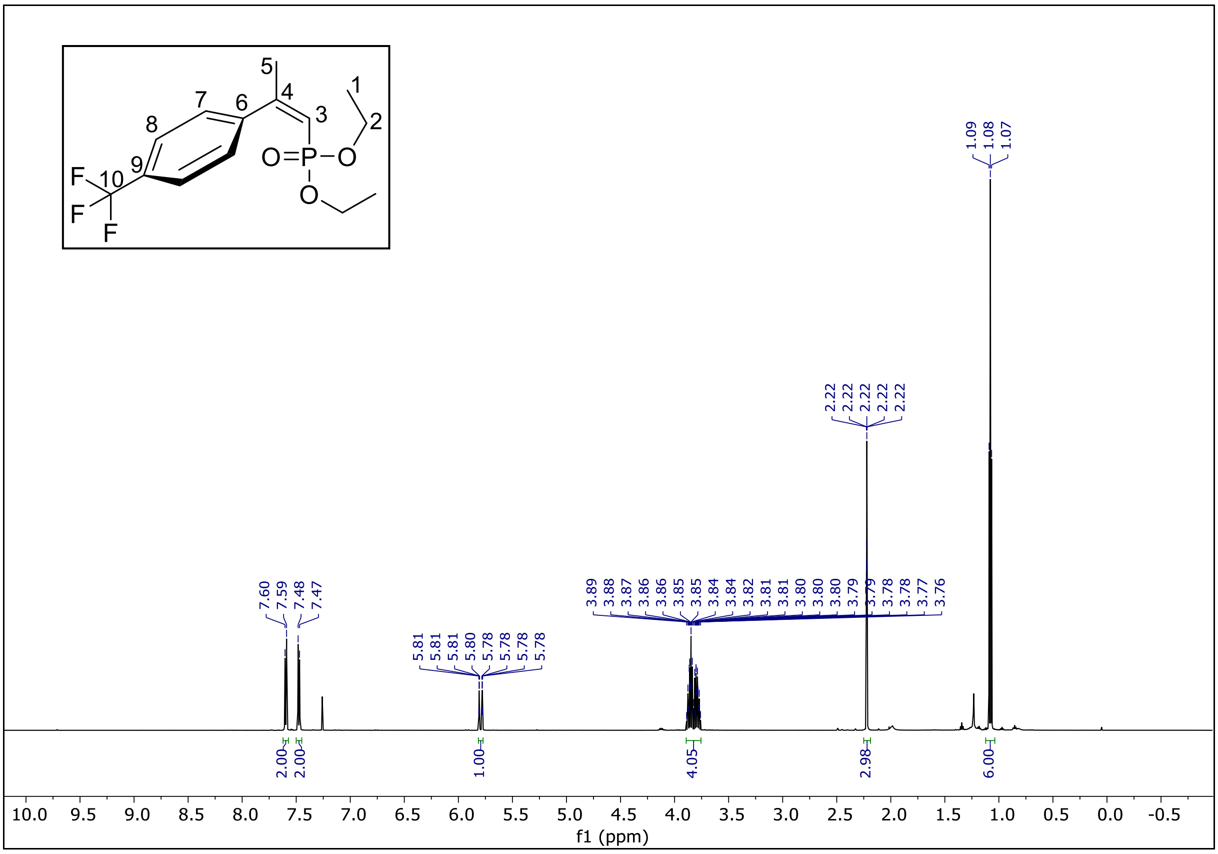

^{13}C NMR (151 MHz, $CDCl_3$): ***Z*-5**

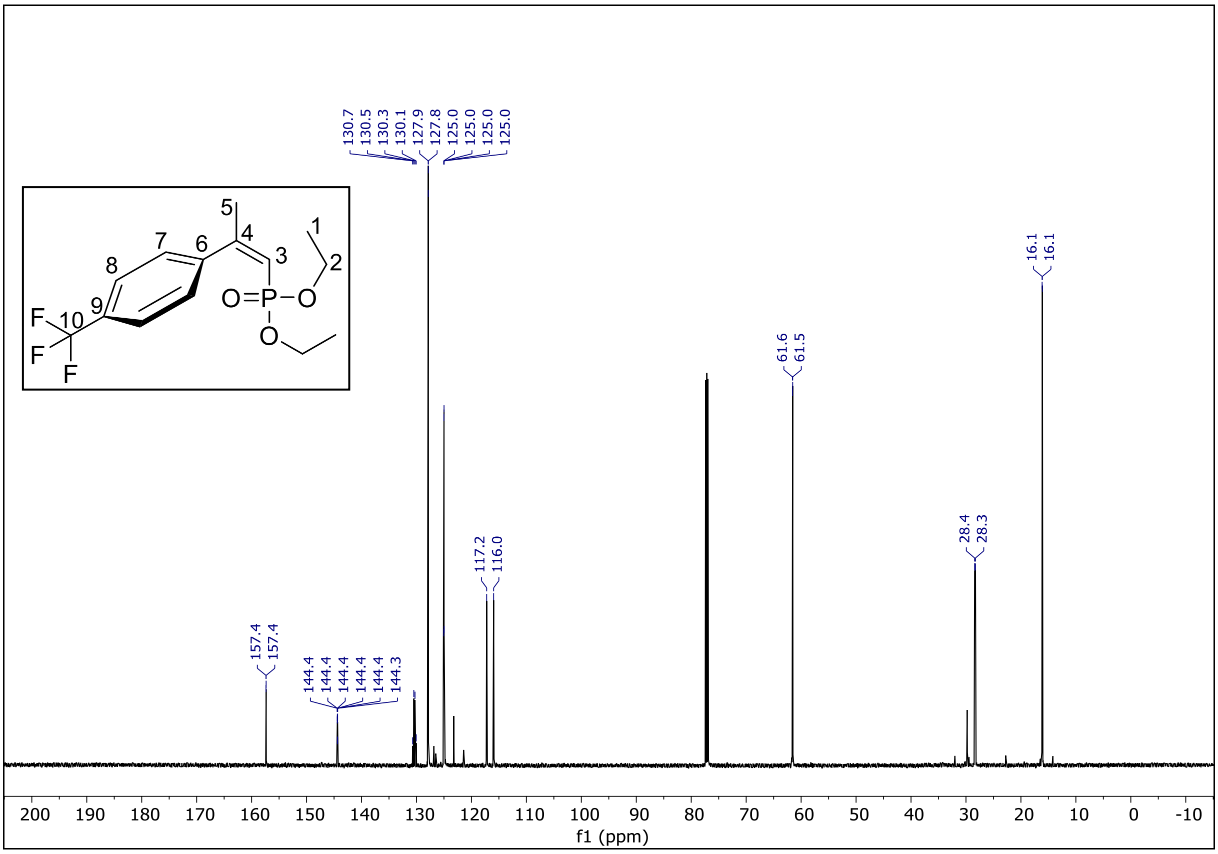

^{19}F NMR (564 MHz, $CDCl_3$): ***Z*-5**

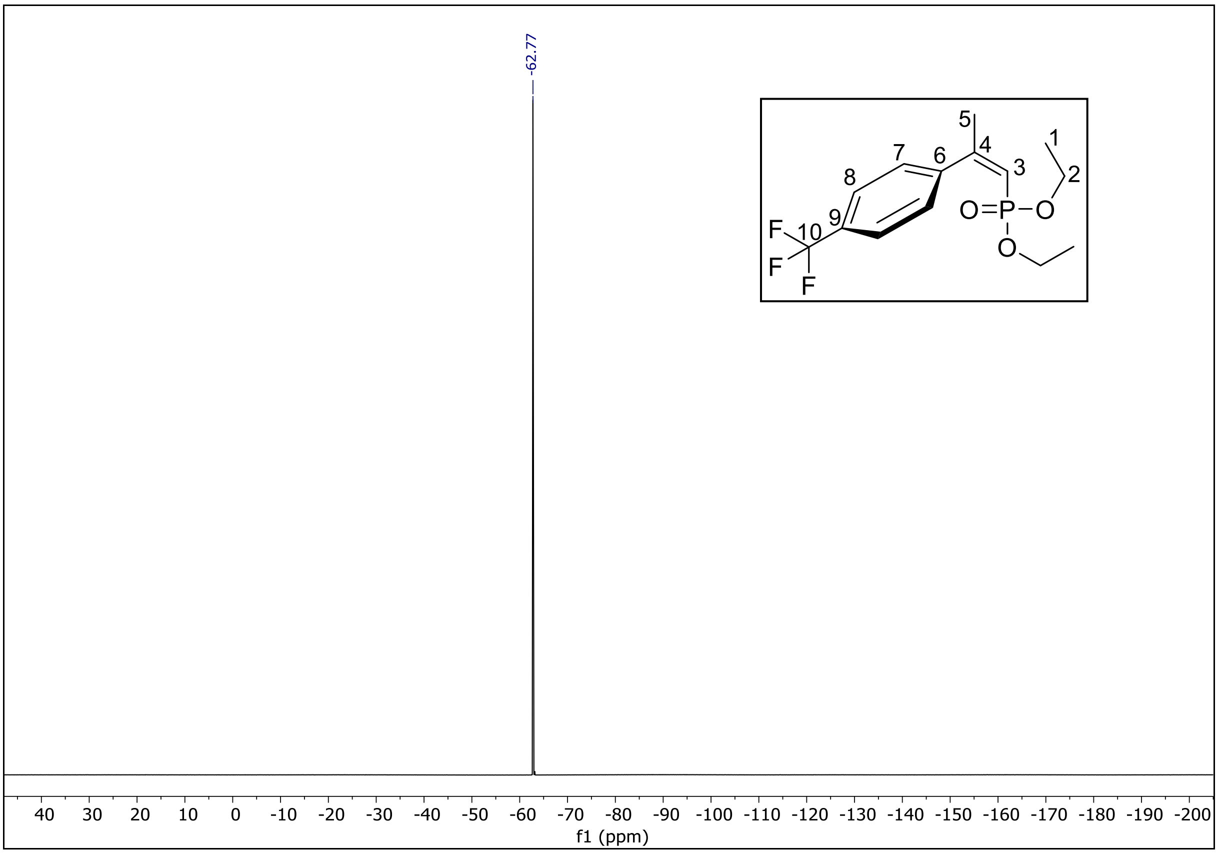

^{31}P NMR (243 MHz, $CDCl_3$): ***Z*-5**

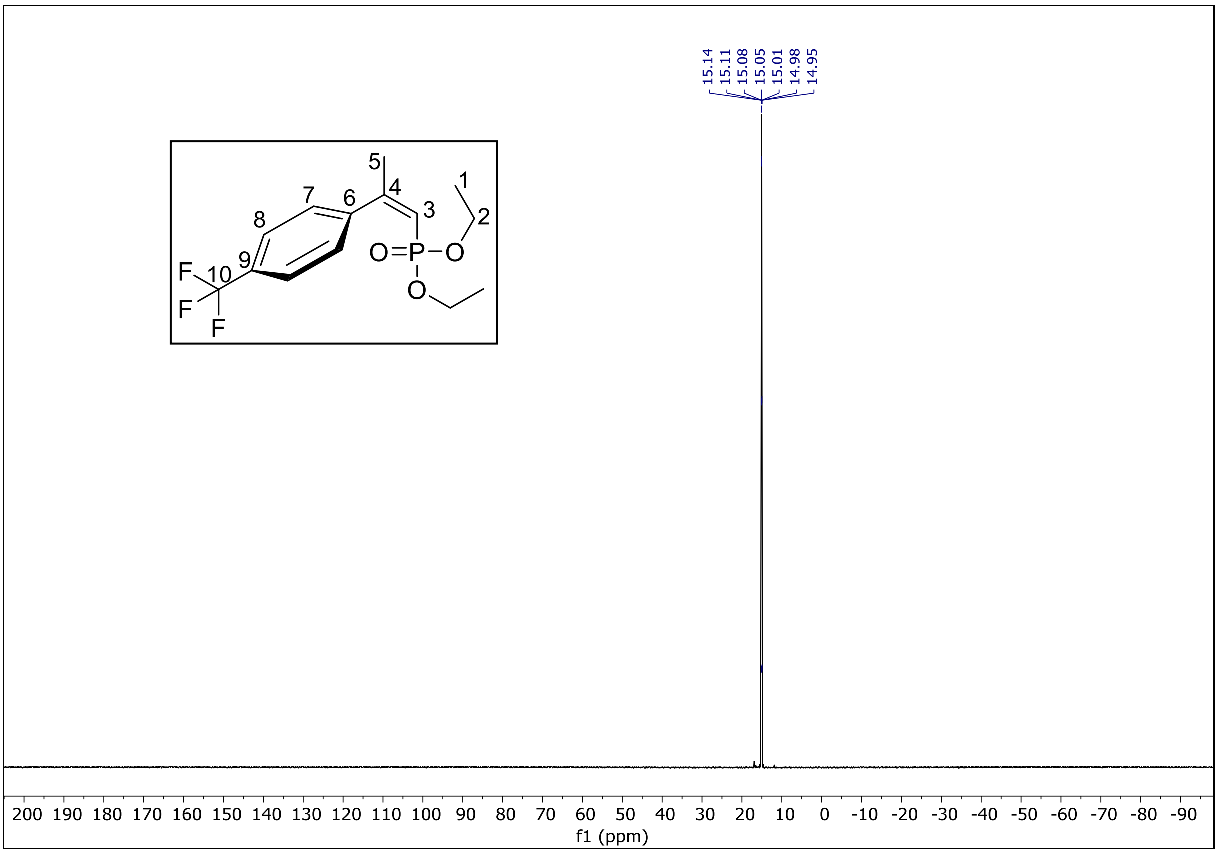

^{1}H NMR (500 MHz, $CDCl_3$): ***Z*-6**

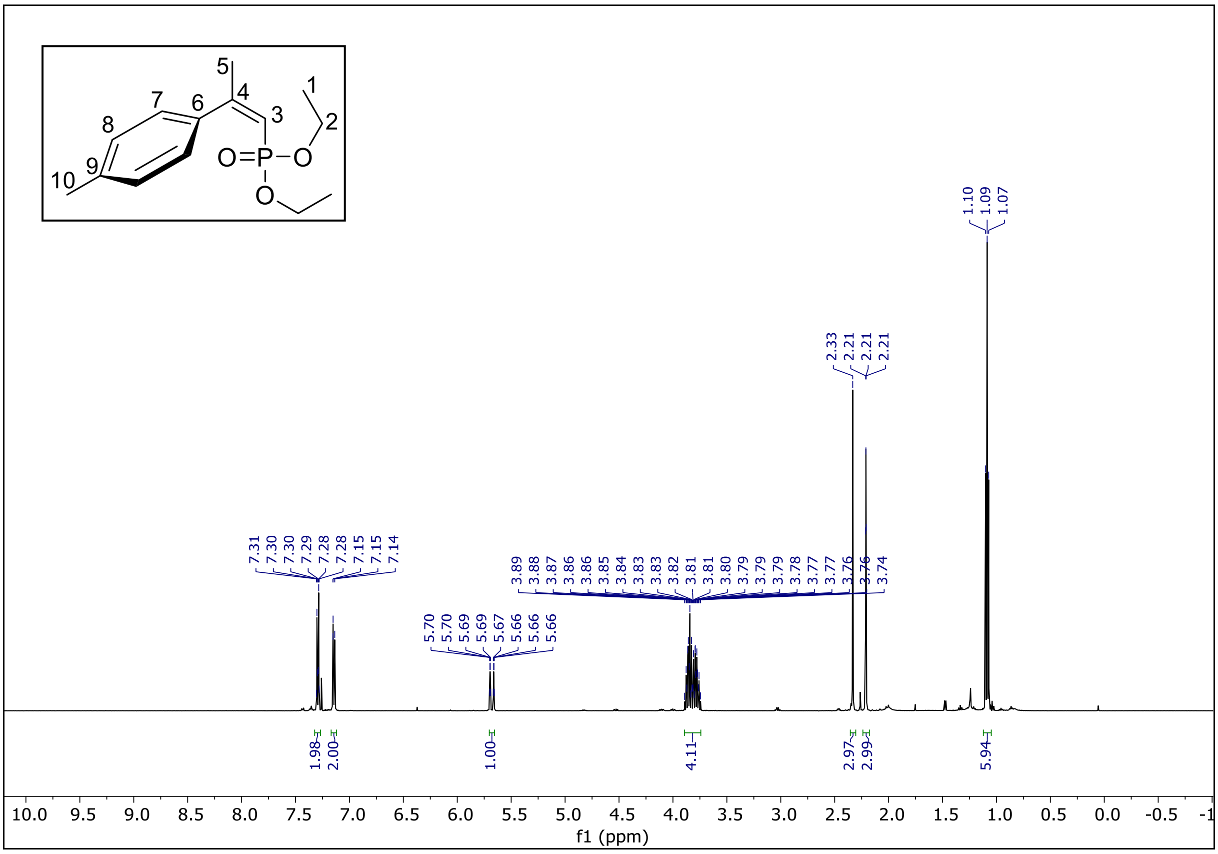

^{13}C NMR (126 MHz, $CDCl_3$): ***Z*-6**

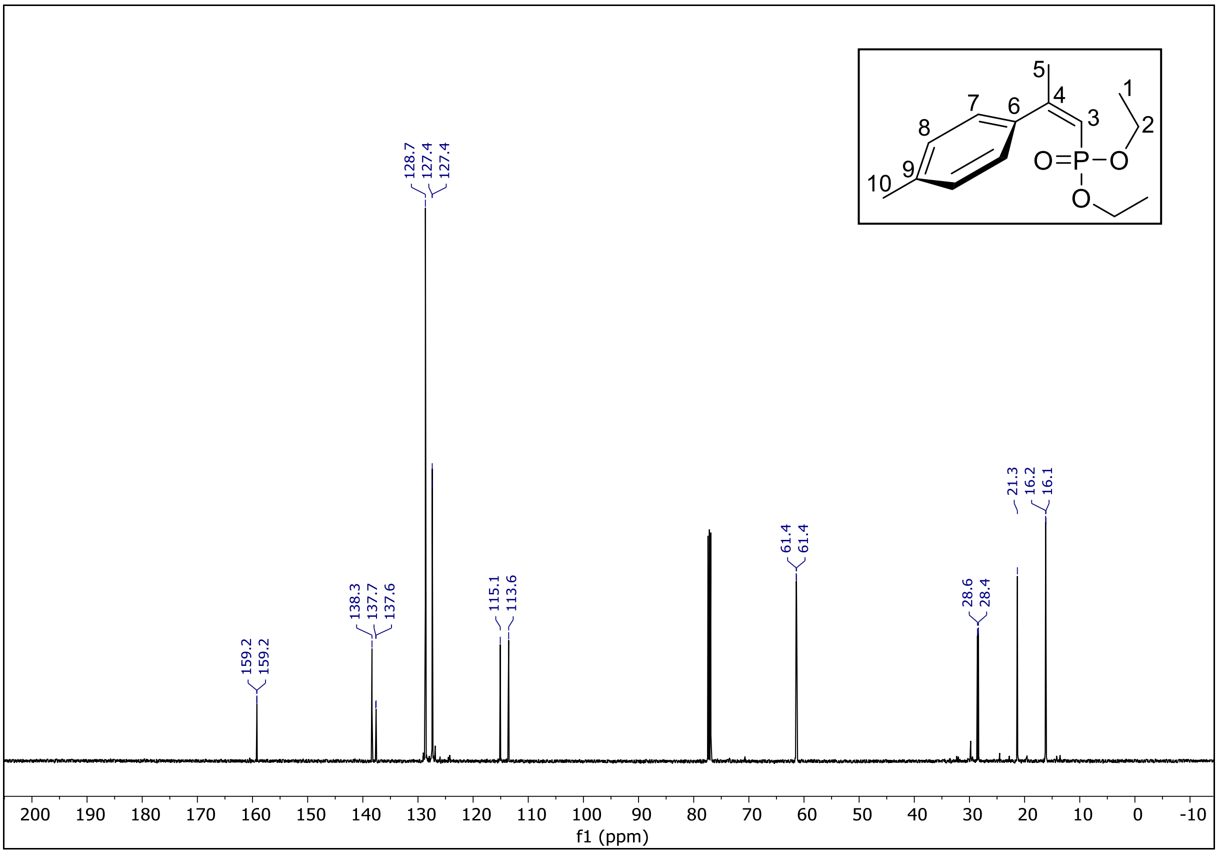

^{31}P NMR (162 MHz, $CDCl_3$): ***Z*-6**

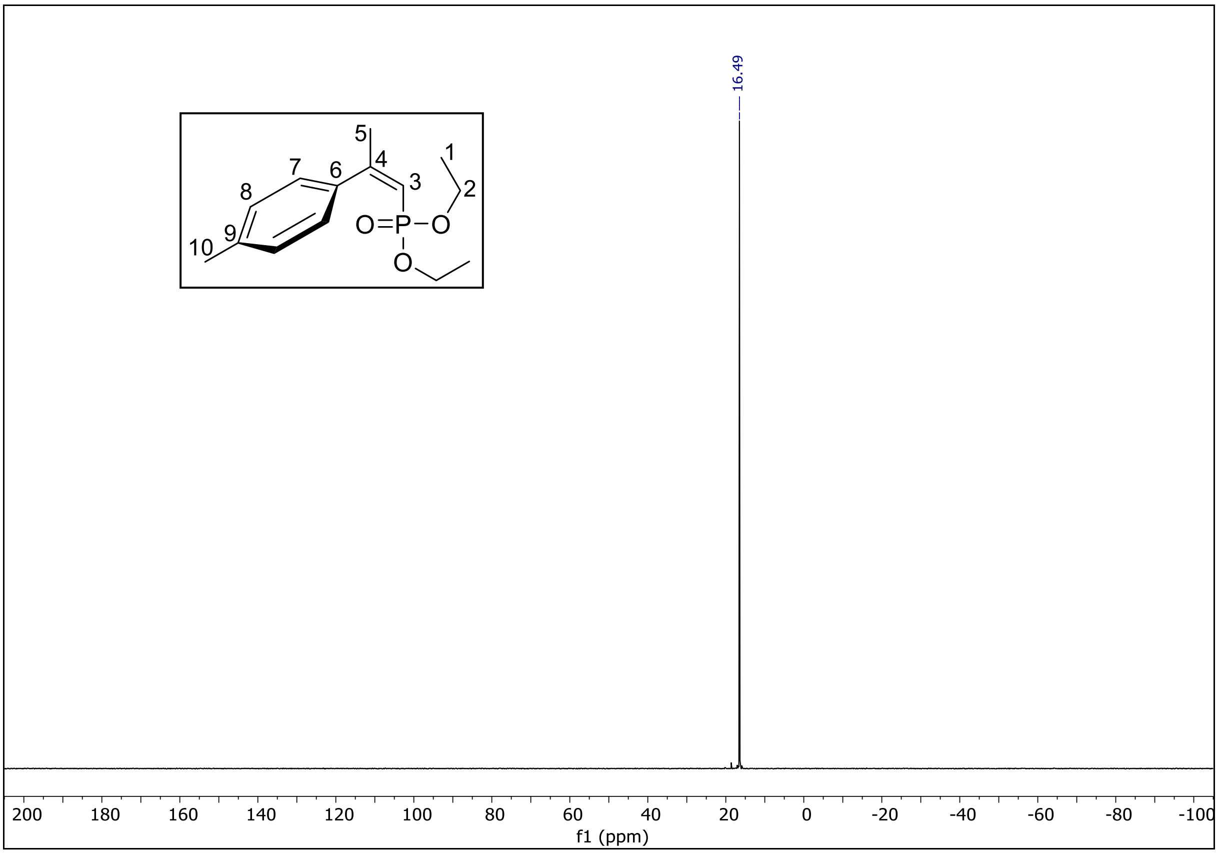

^{1}H NMR (600 MHz, $CDCl_3$): ***Z*-7**

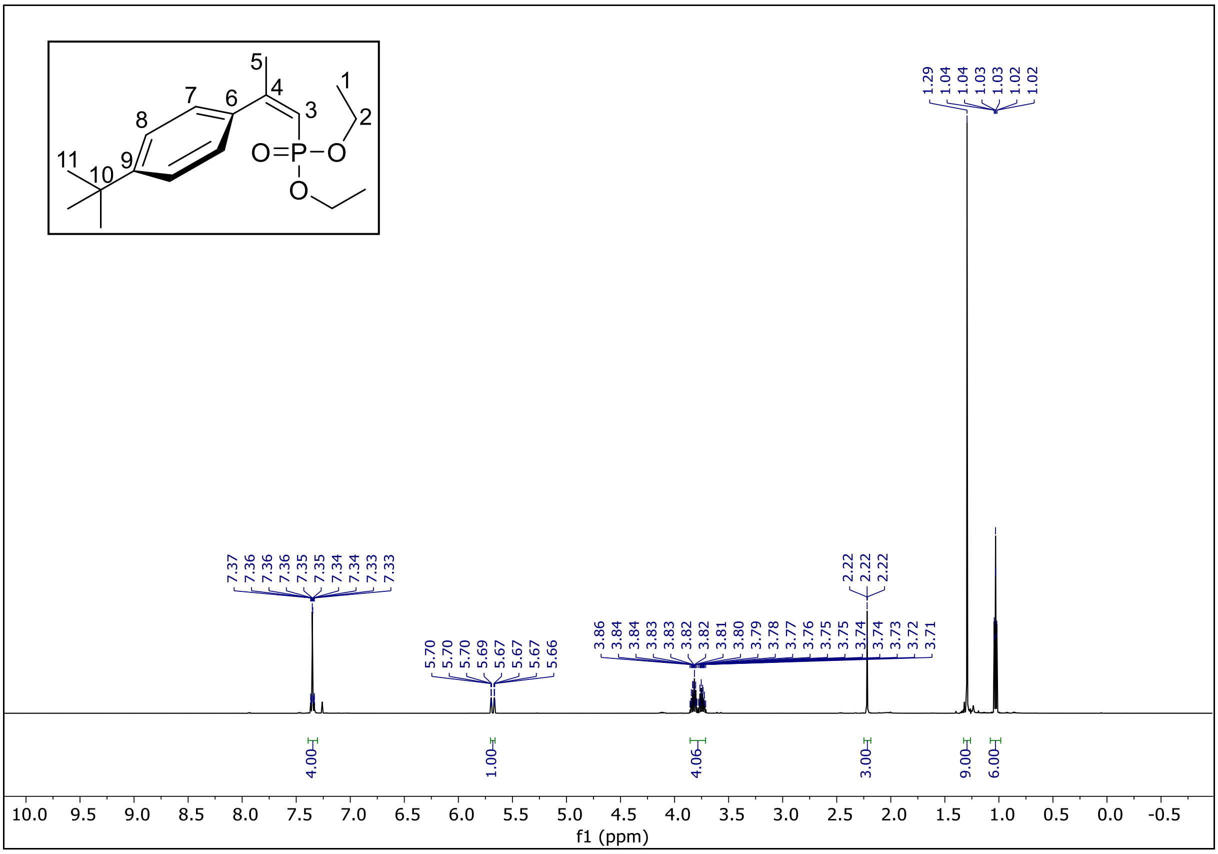

^{13}C NMR (151 MHz, $CDCl_3$): ***Z*-7**

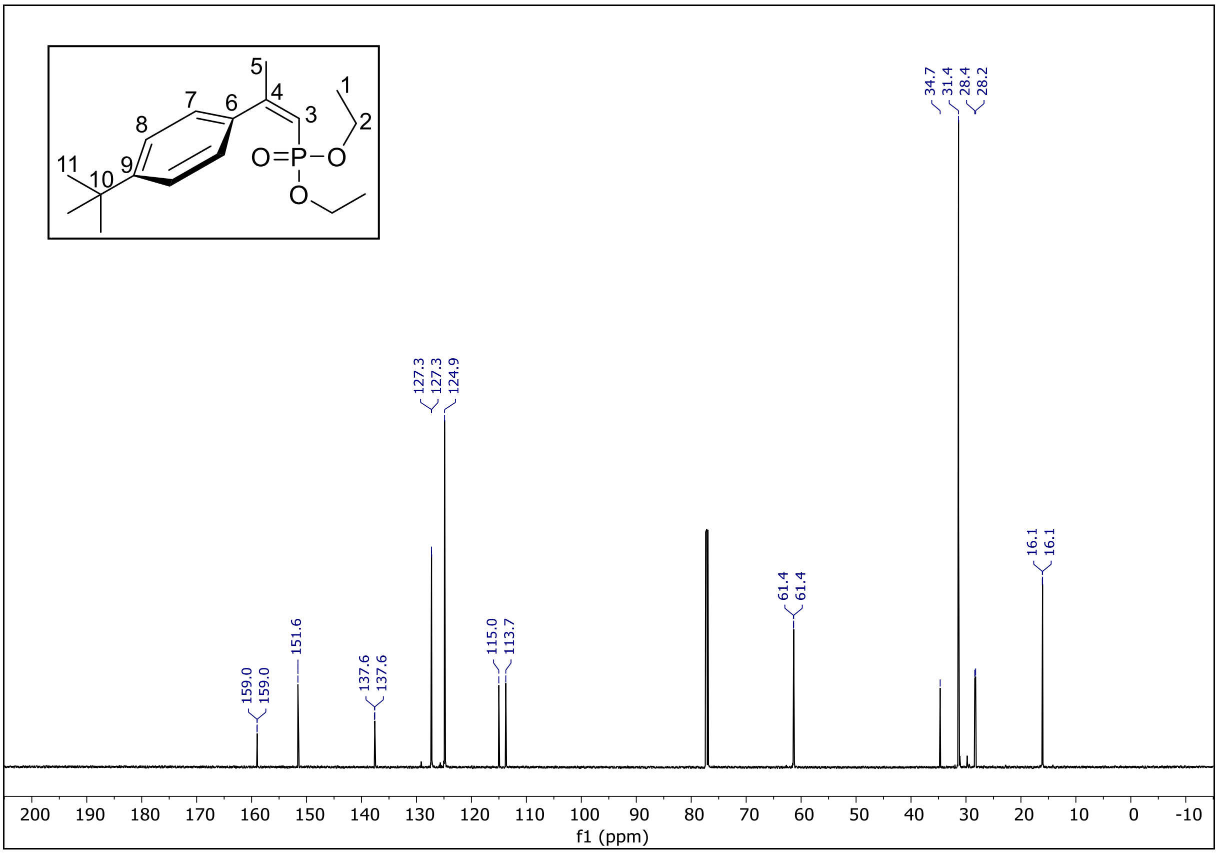

^{31}P NMR (243 MHz, $CDCl_3$): ***Z*-7**

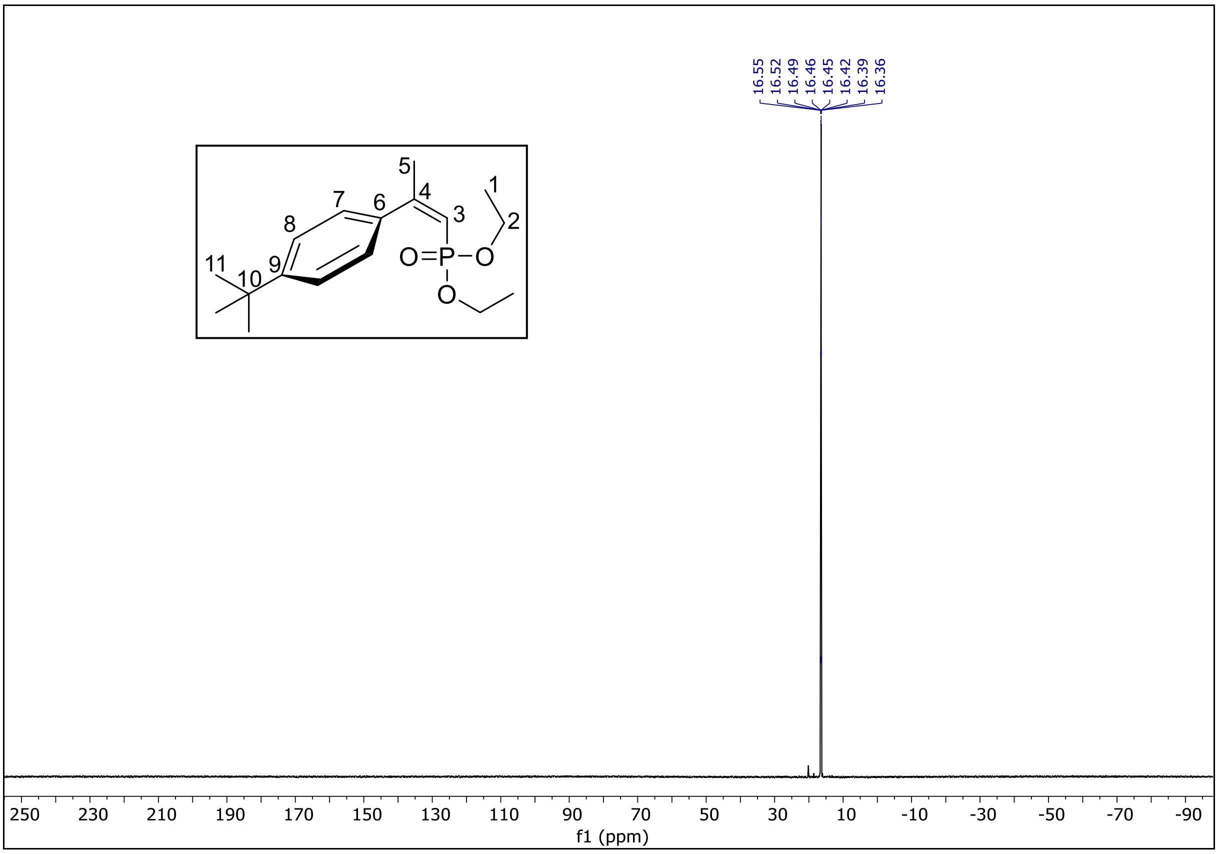

^{1}H NMR (500 MHz, $CDCl_3$): ***Z*-8**

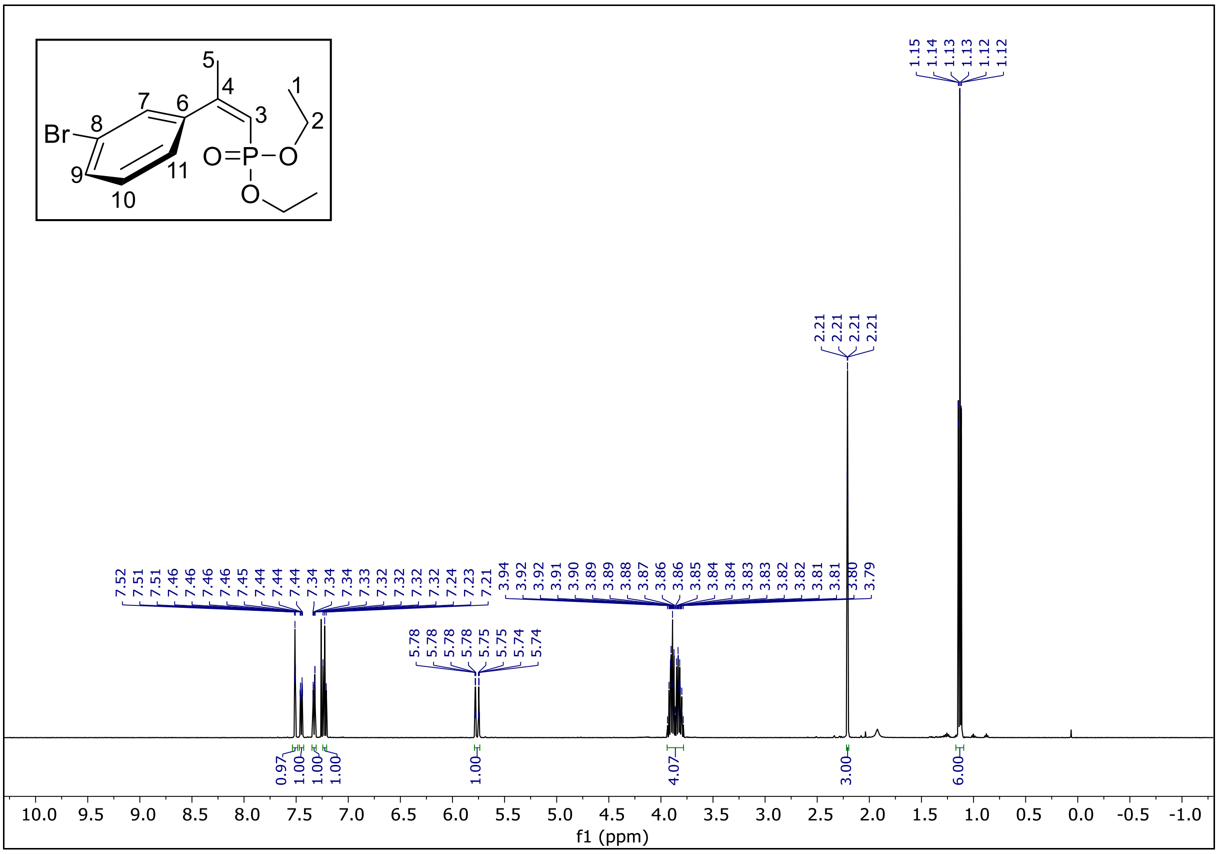

^{13}C NMR (126 MHz, $CDCl_3$): ***Z*-8**

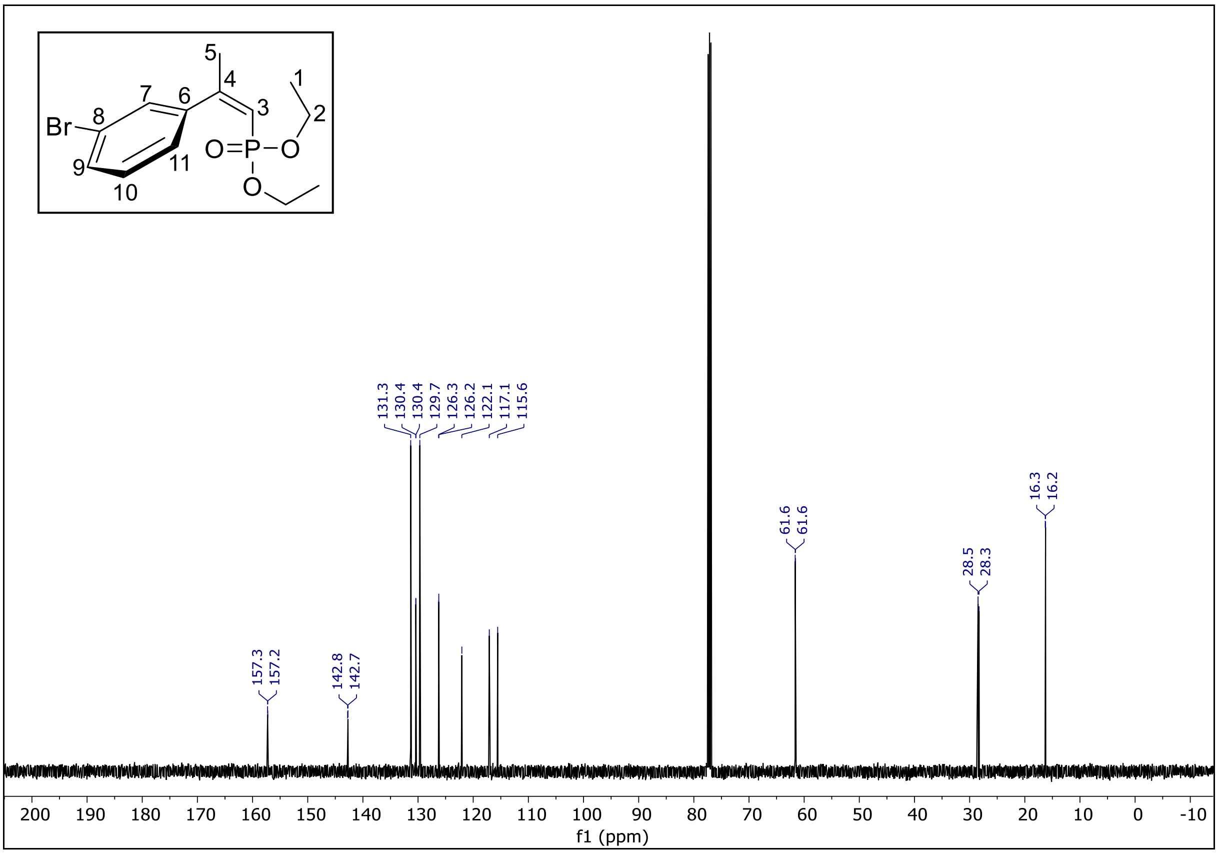

^{31}P NMR (202 MHz, CDCl$_3$): ***Z*-8**

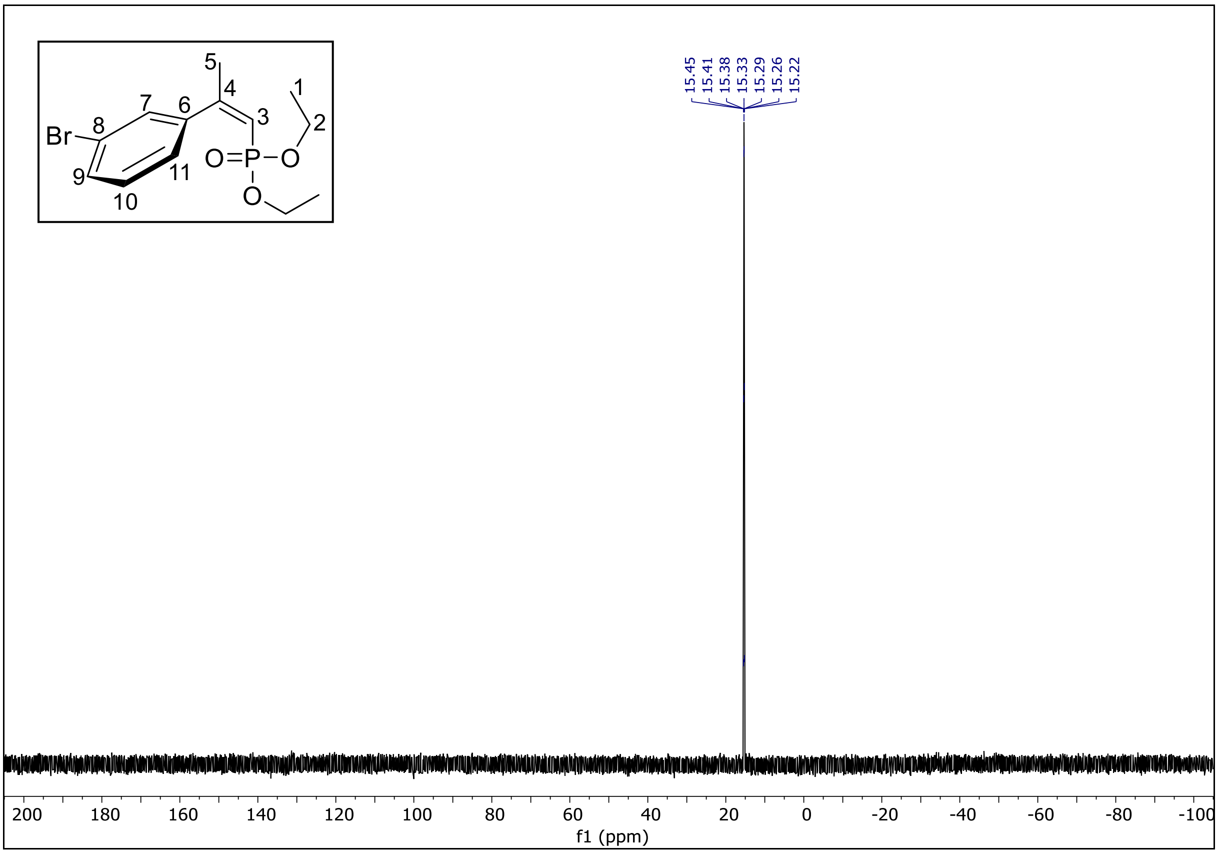

^{1}H NMR (600 MHz, CDCl$_3$): ***Z*-9**

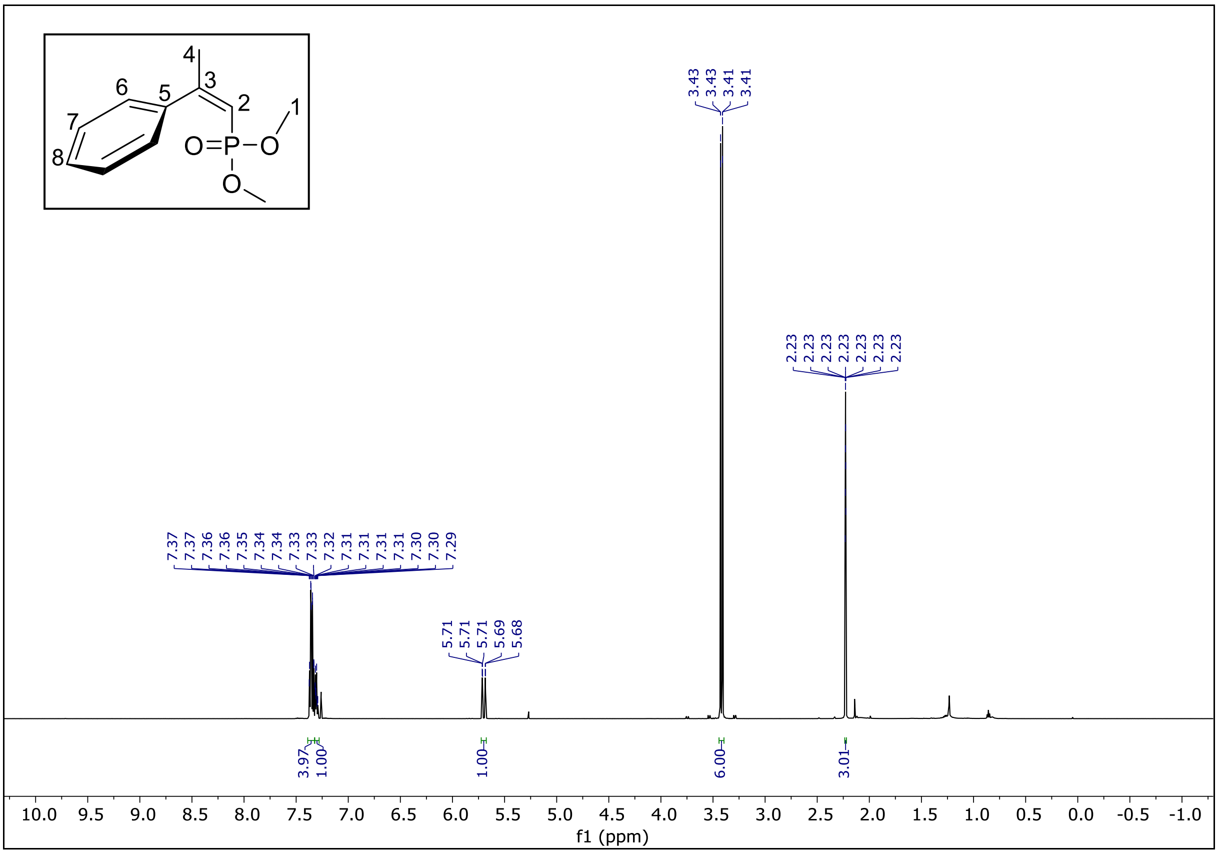

^{13}C NMR (151 MHz, $CDCl_3$): ***Z*-9**

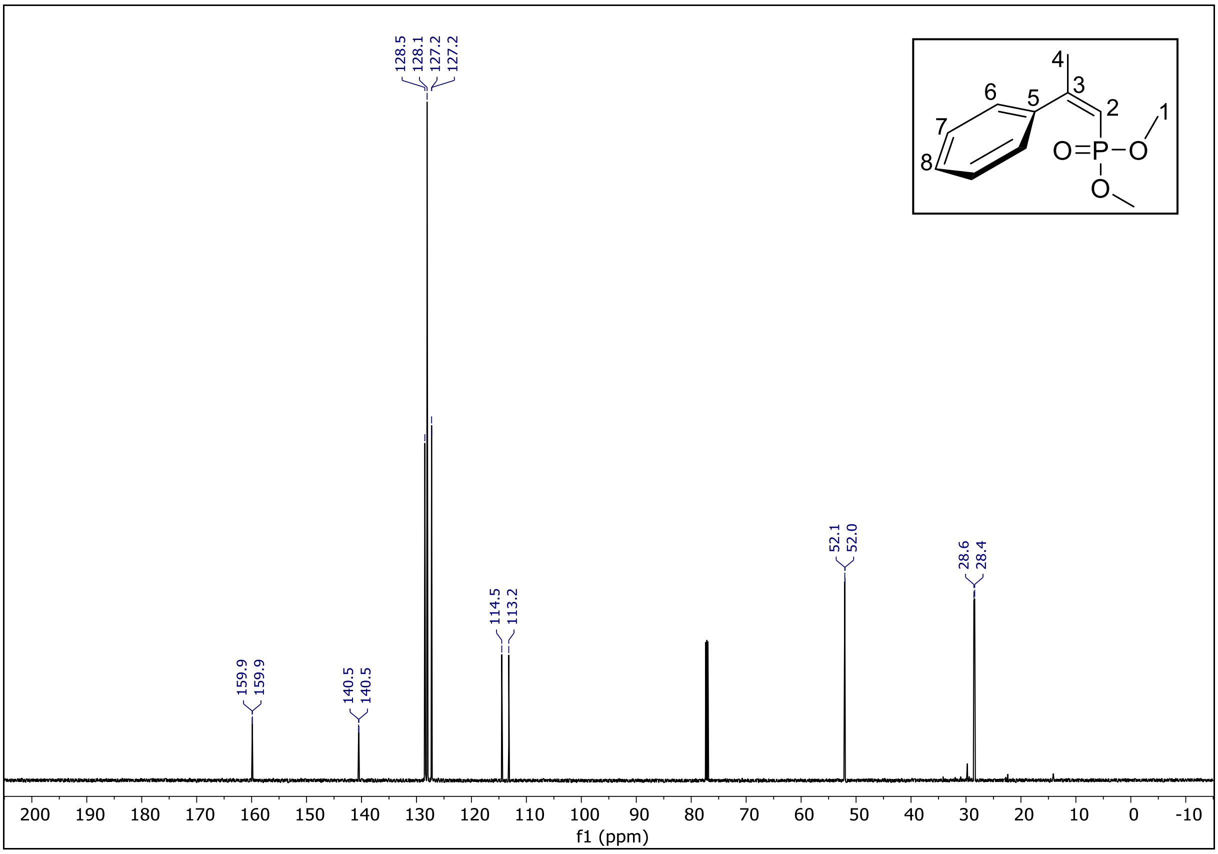

^{31}P NMR (162 MHz, $CDCl_3$): ***Z*-9**

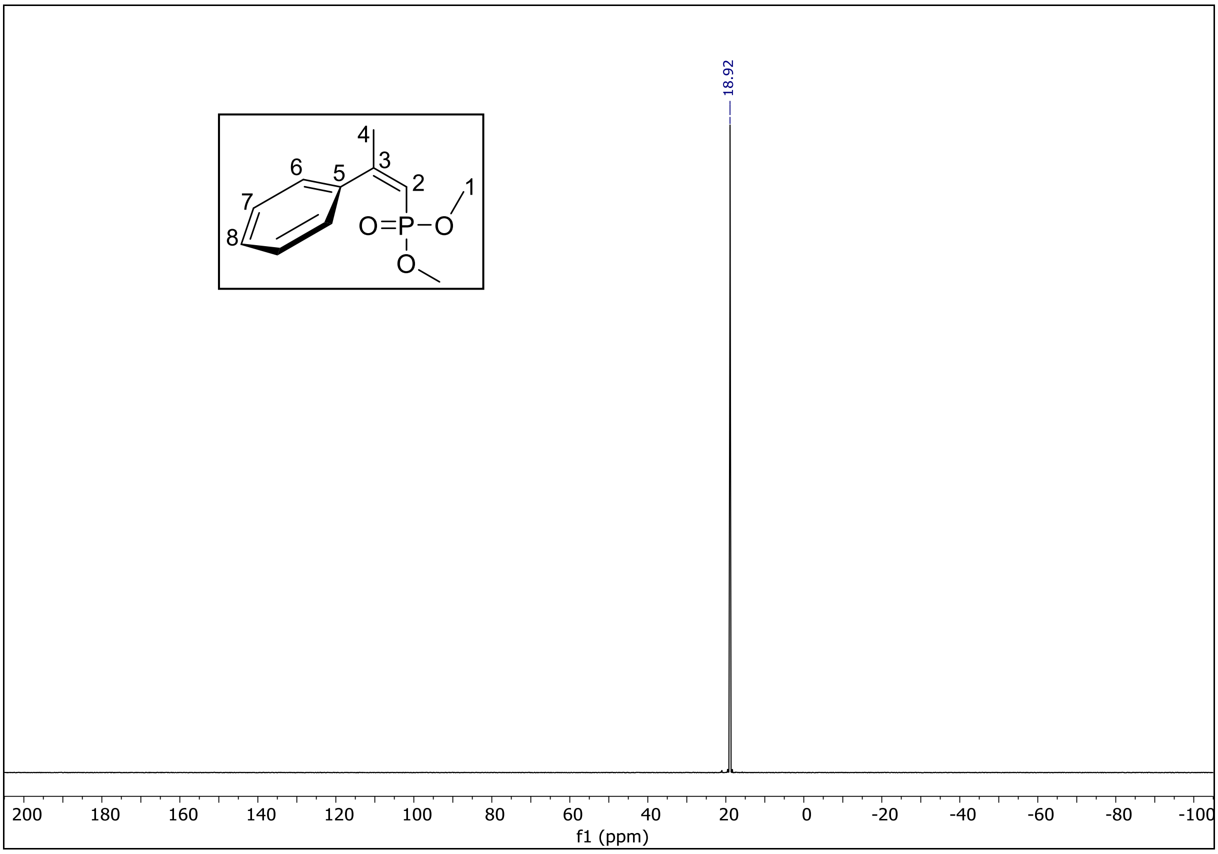

^{1}H NMR (600 MHz, $CDCl_3$): ***Z*-10**

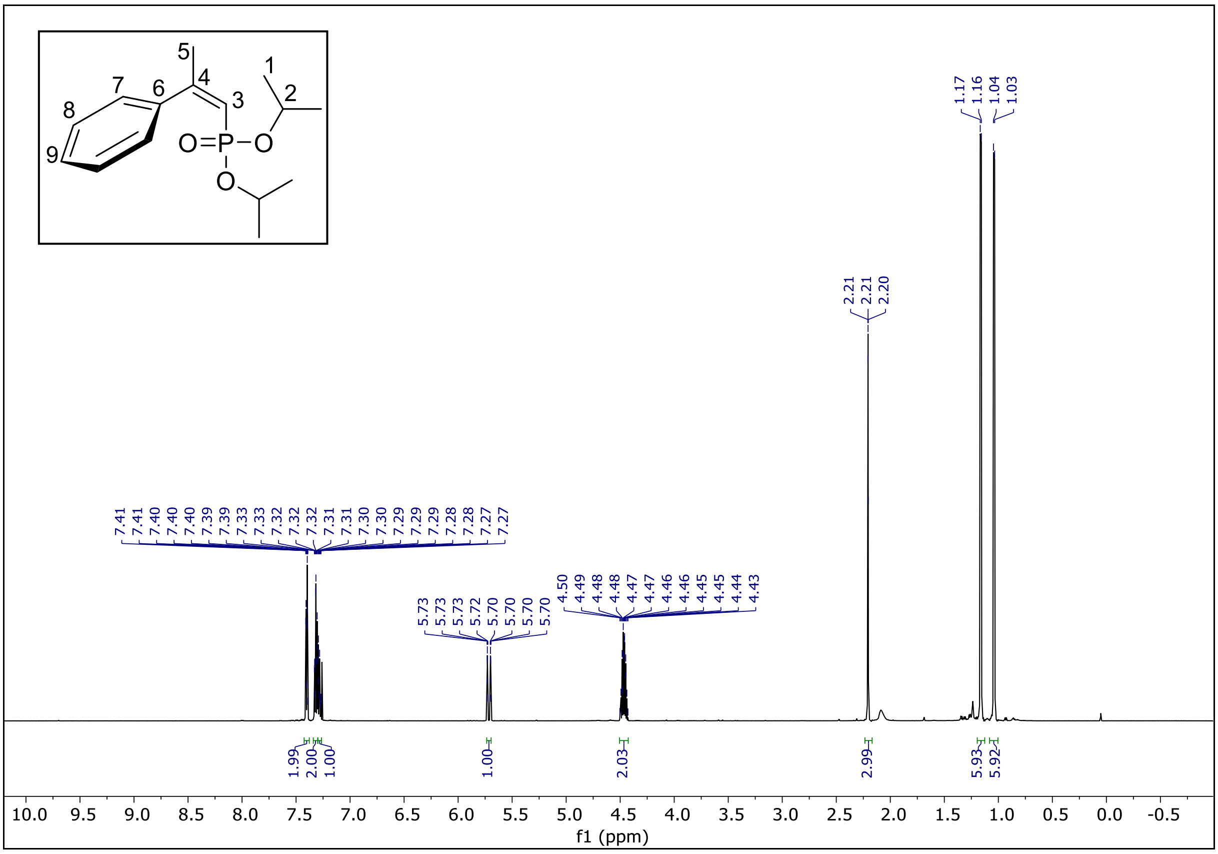

^{13}C NMR (151 MHz, $CDCl_3$): ***Z*-10**

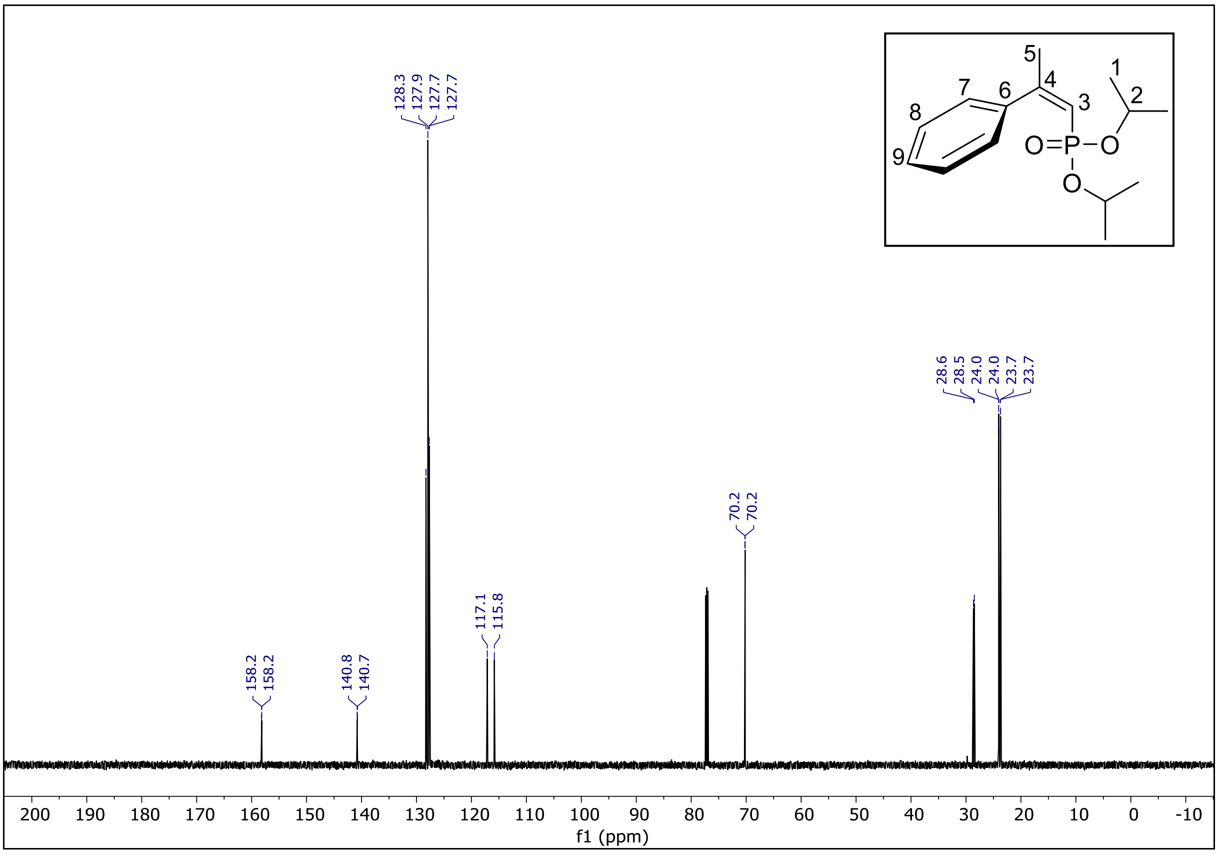

^{31}P NMR (243 MHz, $CDCl_3$): ***Z*-10**

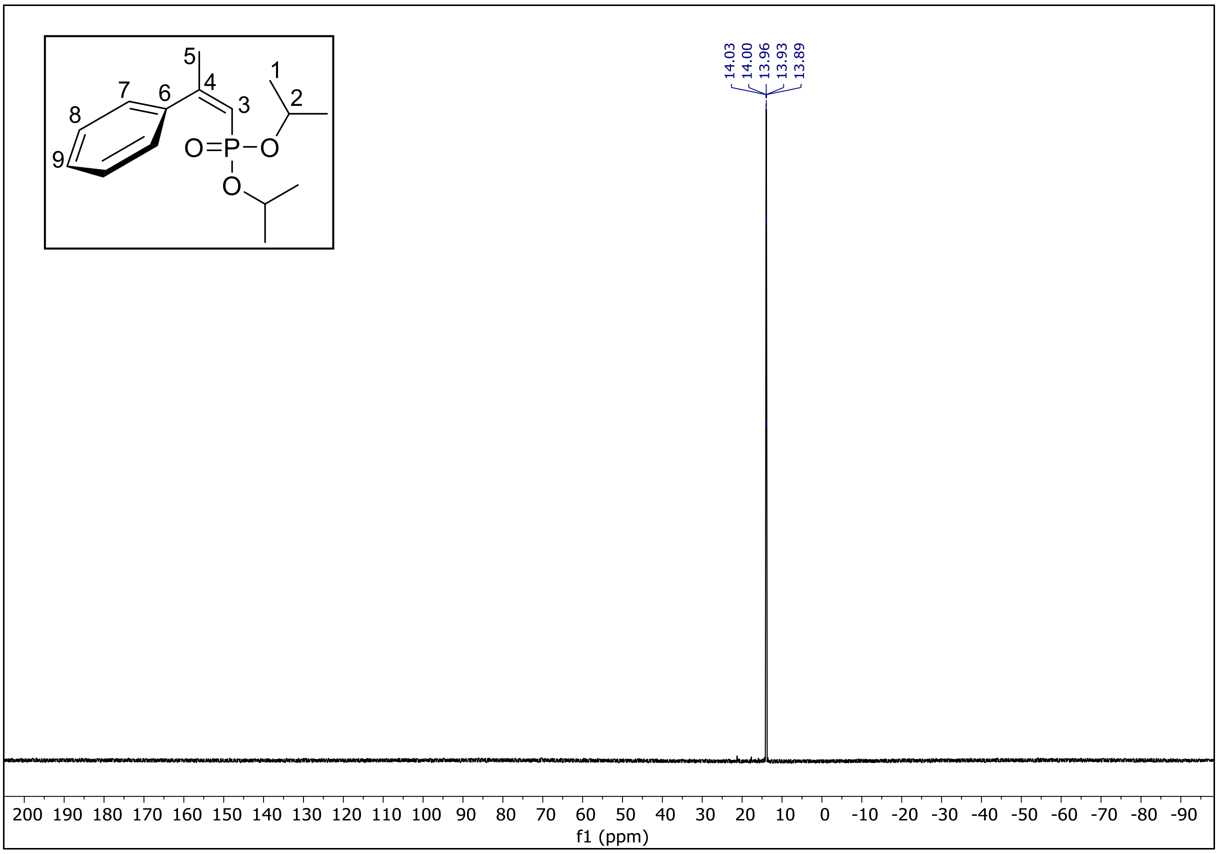

^{1}H NMR (300 MHz, $CDCl_3$): ***Z*-11**

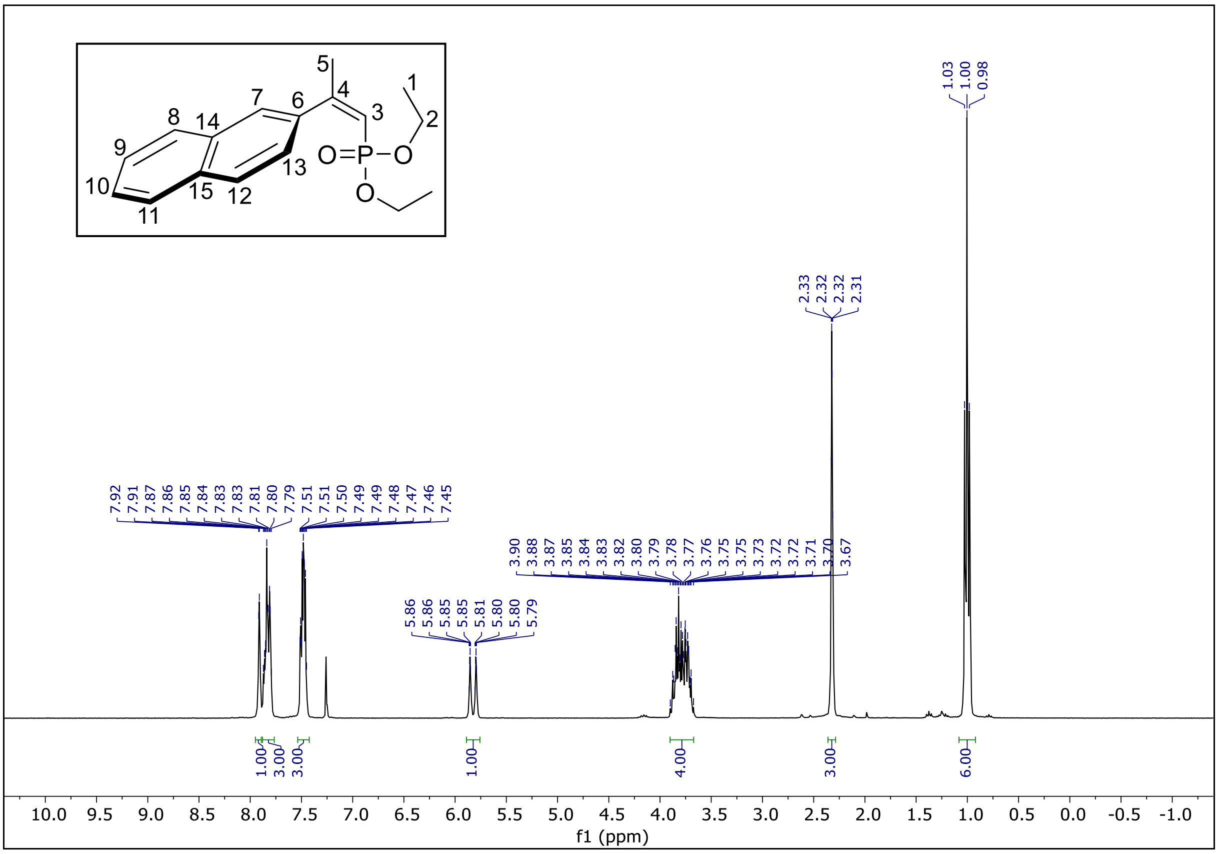

^{13}C NMR (151 MHz, $CDCl_3$): ***Z*-11**

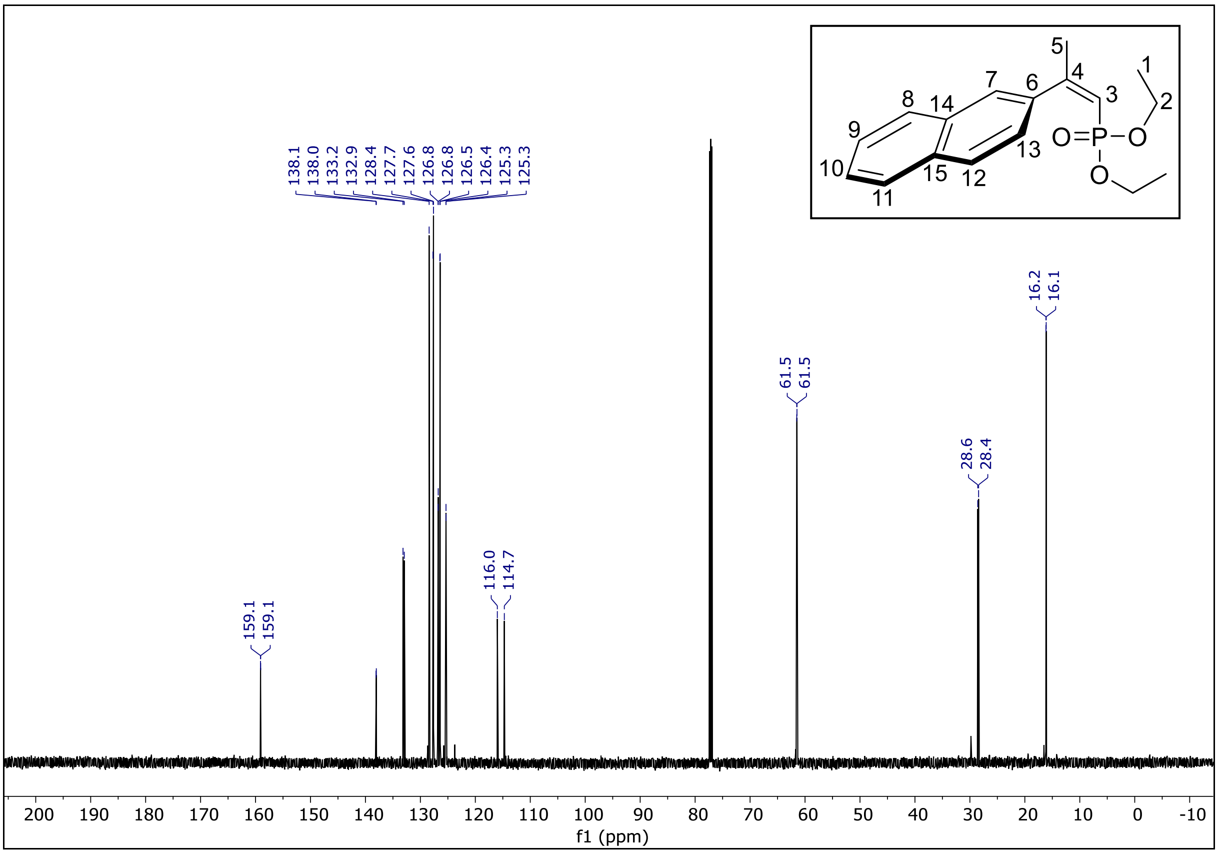

^{31}P NMR (121 MHz, $CDCl_3$): ***Z*-11**

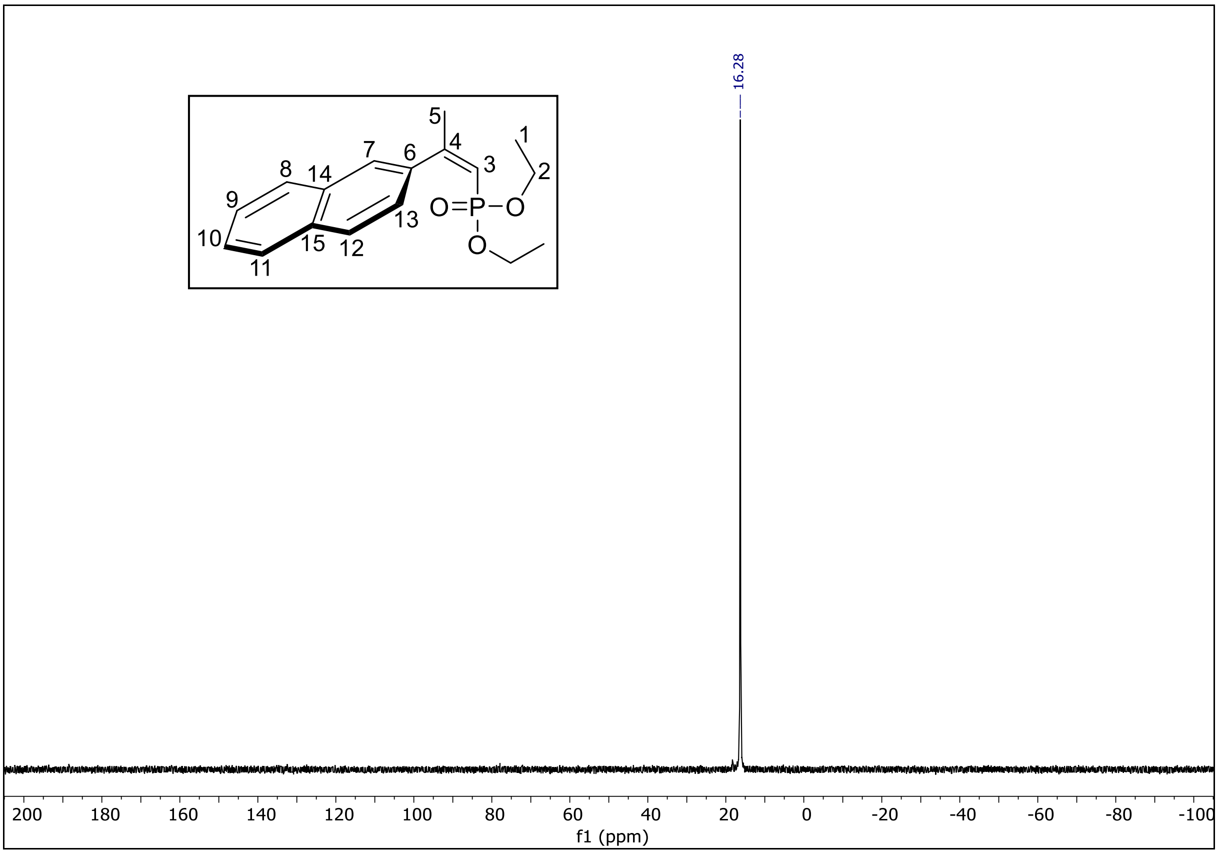

^{1}H NMR (600 MHz, $CDCl_3$): ***Z*-12**

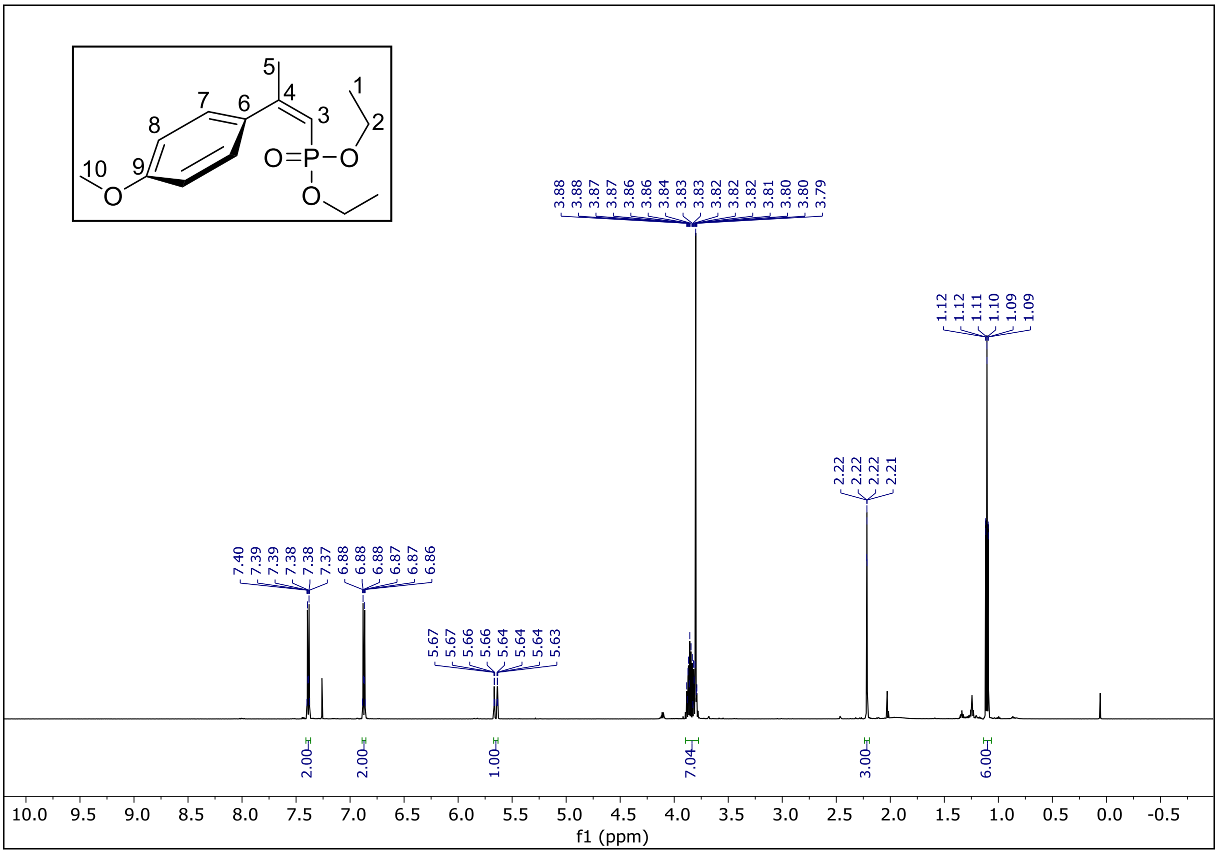

^{13}C NMR (151 MHz, $CDCl_3$): ***Z*-12**

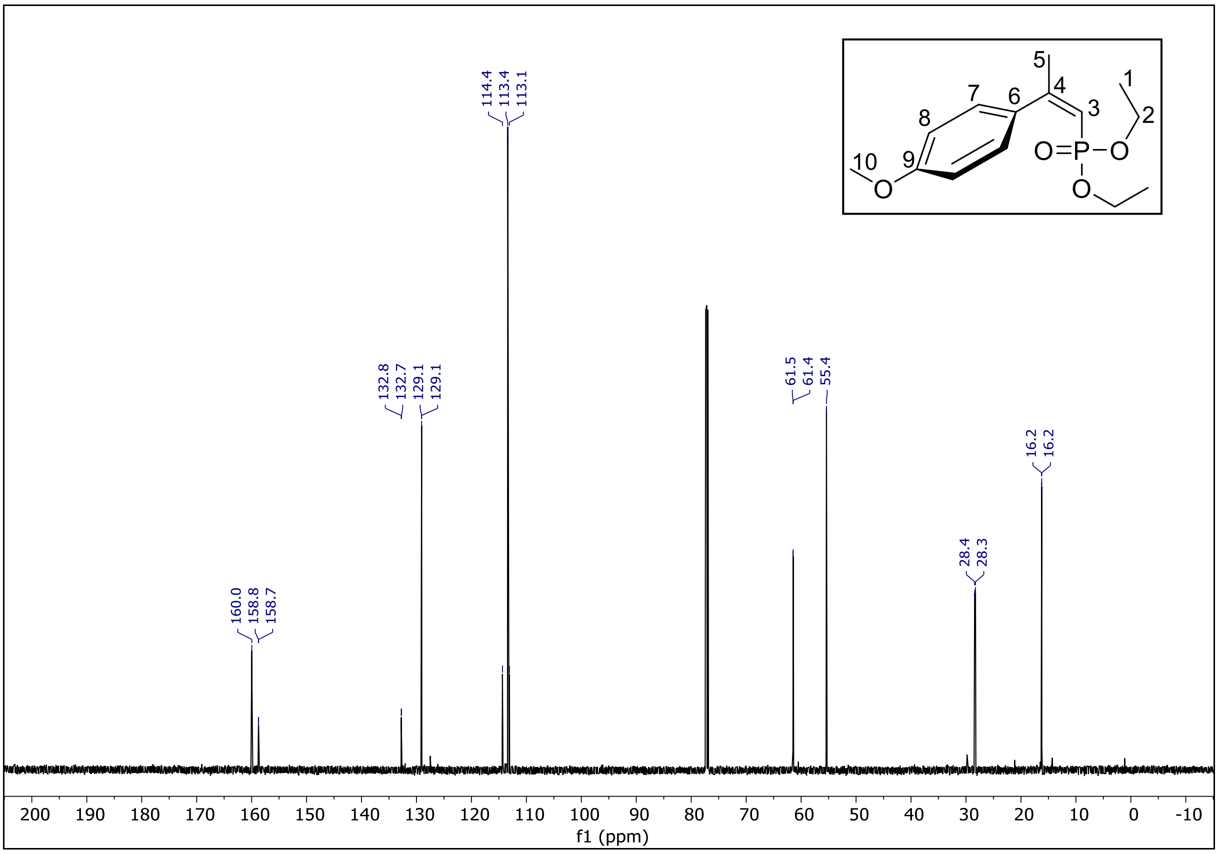

^{31}P NMR (162 MHz, $CDCl_3$): ***Z*-12**

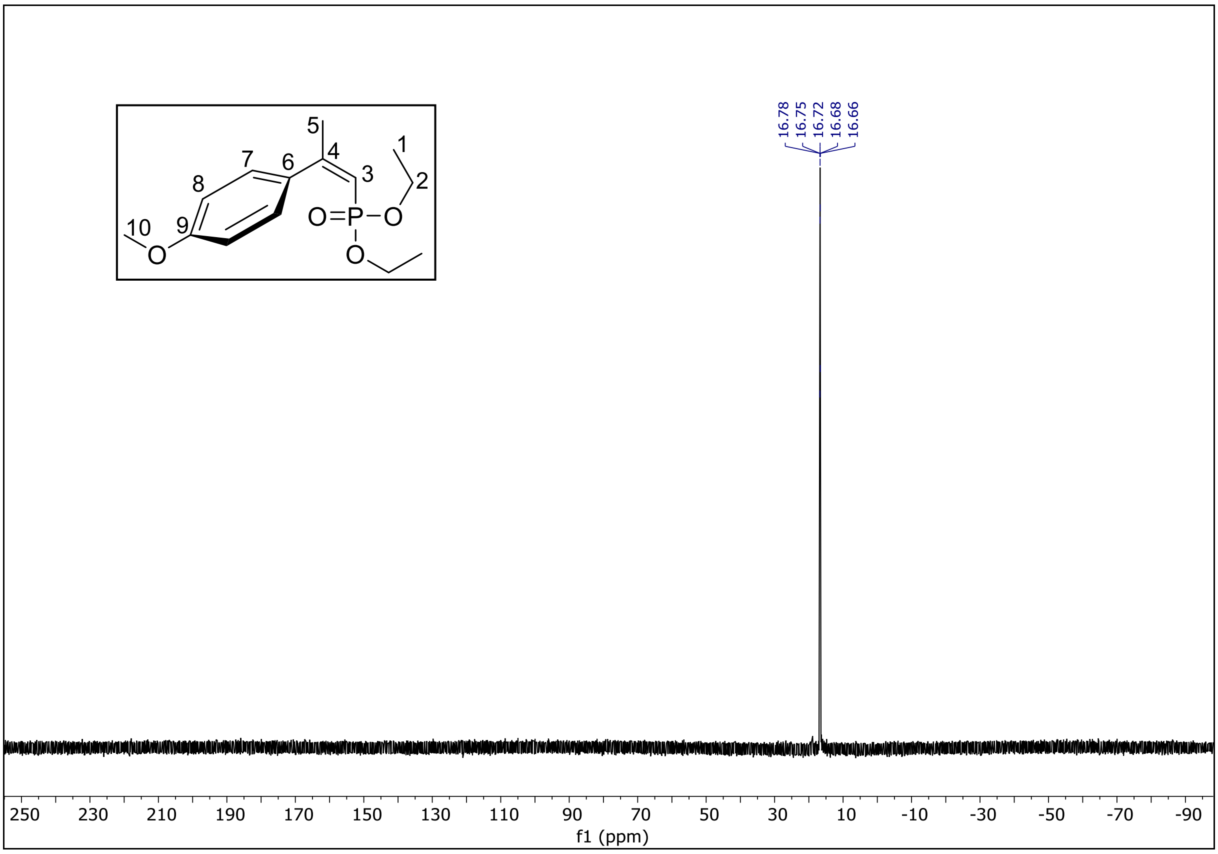

^{1}H NMR (600 MHz, $CDCl_3$): **(*E/Z*)-13** (1:1)

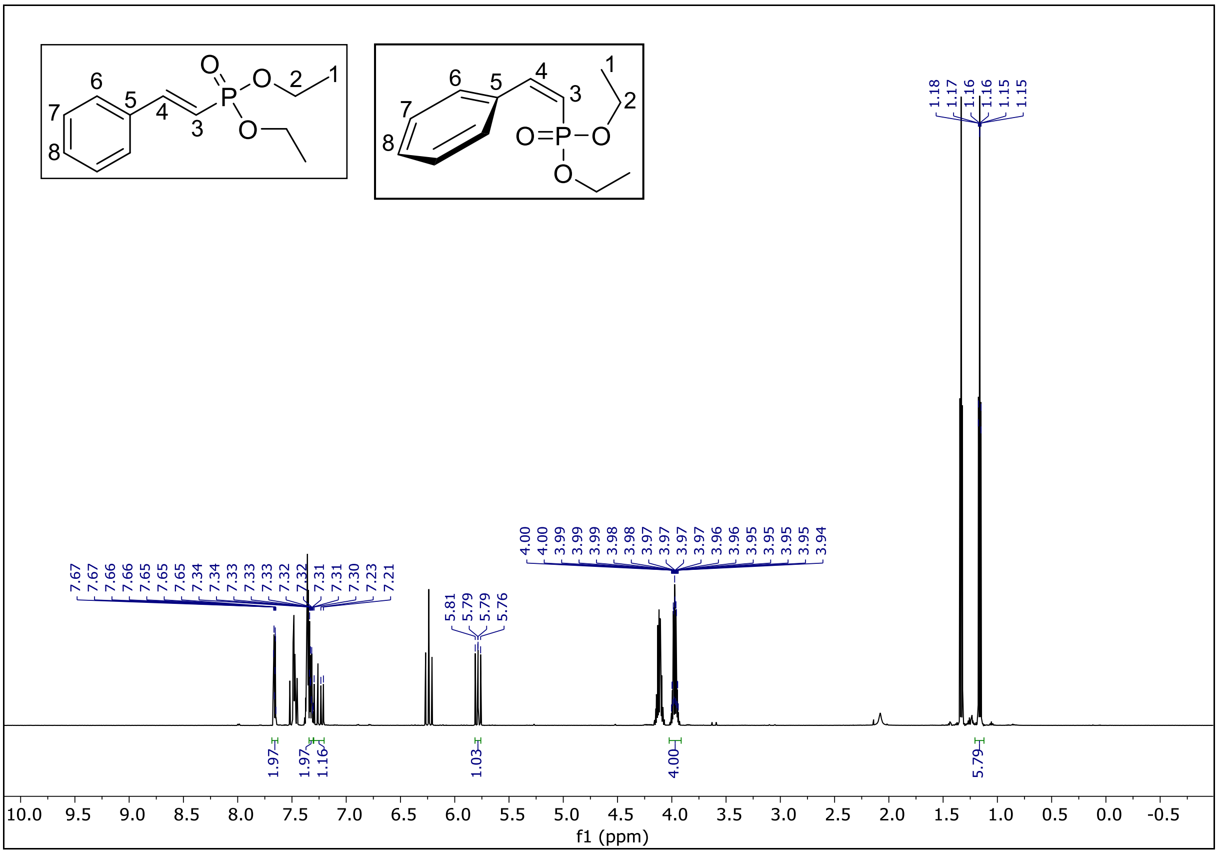

^{13}C NMR (151 MHz, CDCl$_3$): ***(E/Z)*-13** (1:1)

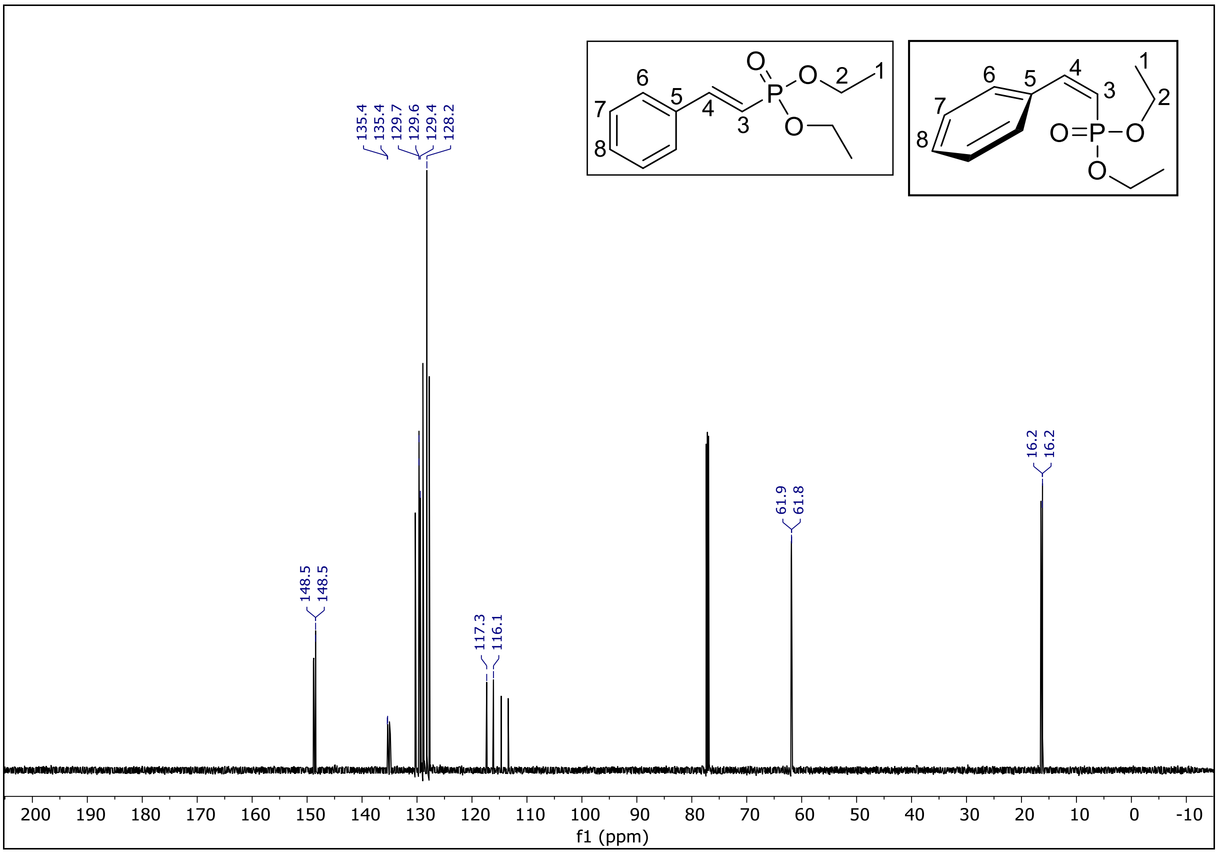

^{31}P NMR (162 MHz, CDCl$_3$): ***Z*-13**

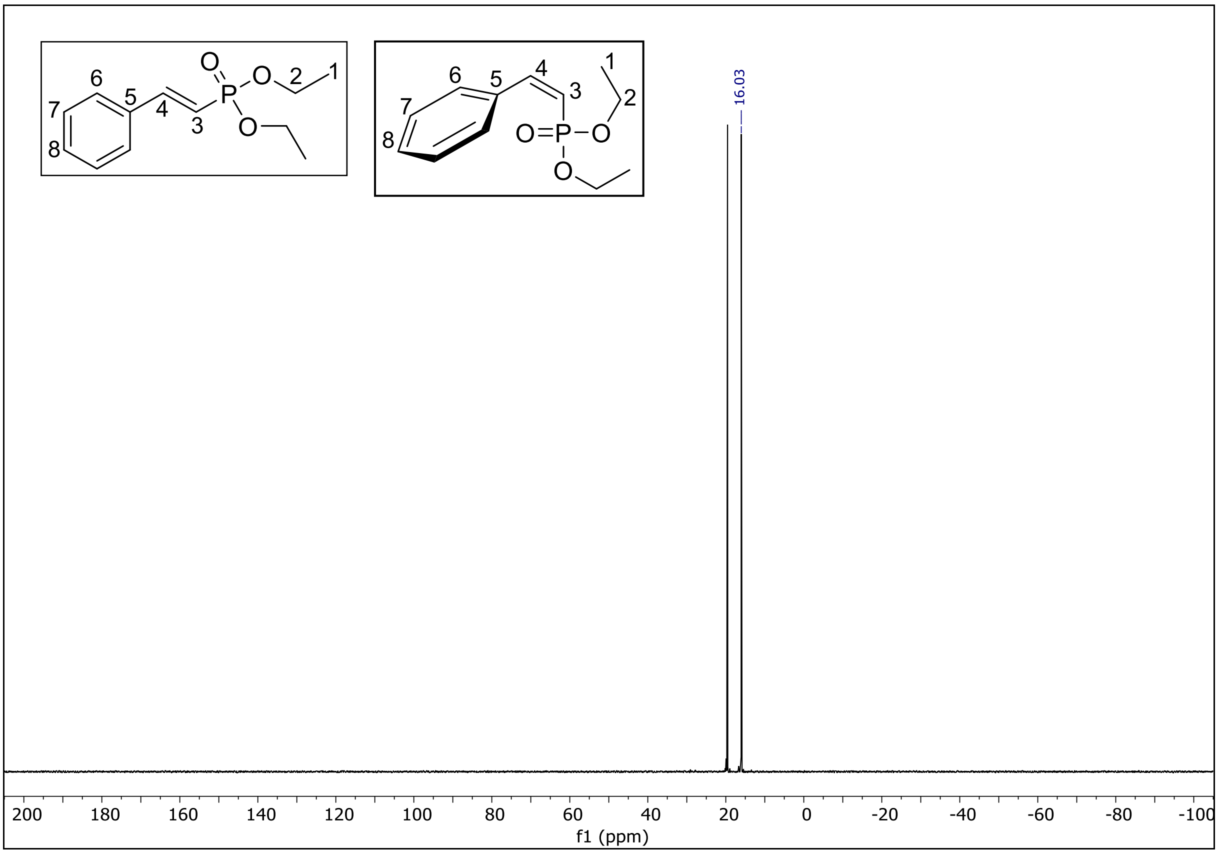

^{1}H NMR (500 MHz, $CDCl_3$): ***Z*-14**

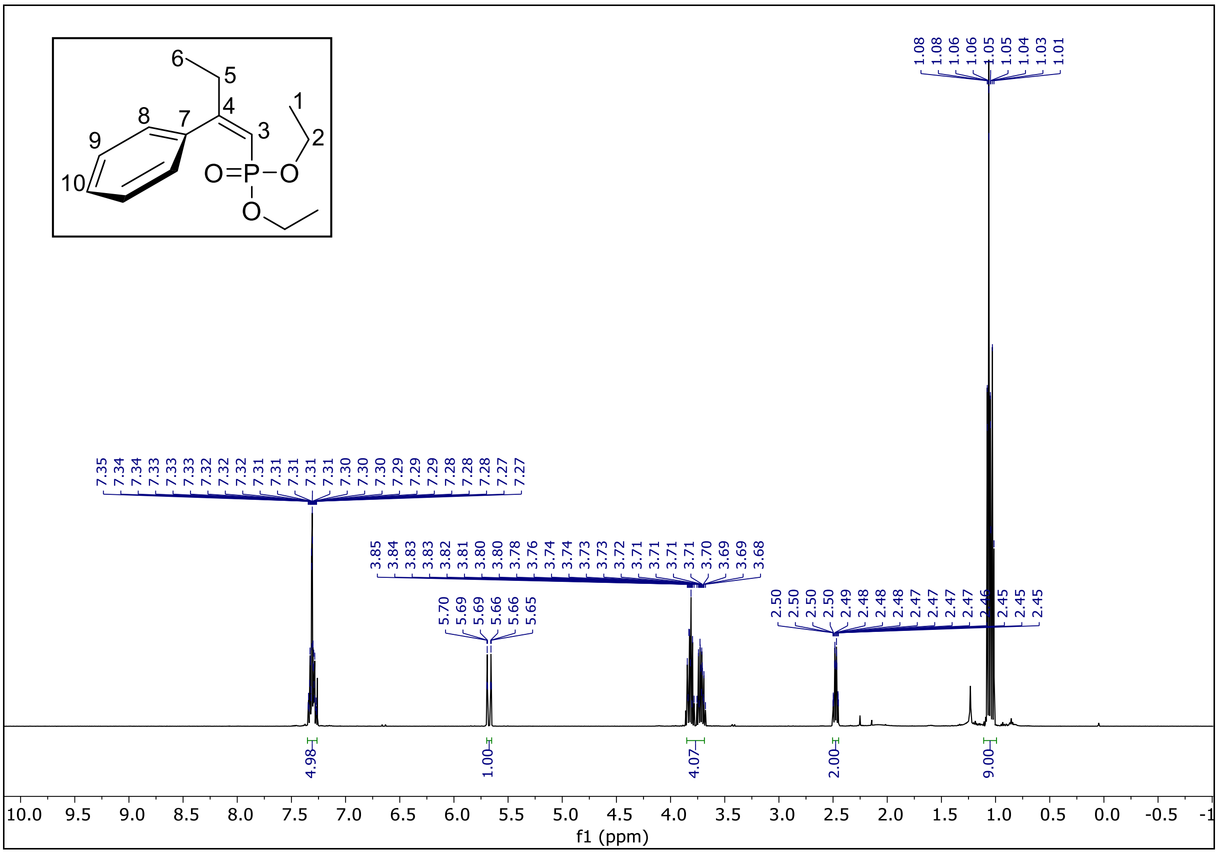

^{13}C NMR (126 MHz, $CDCl_3$): ***Z*-14**

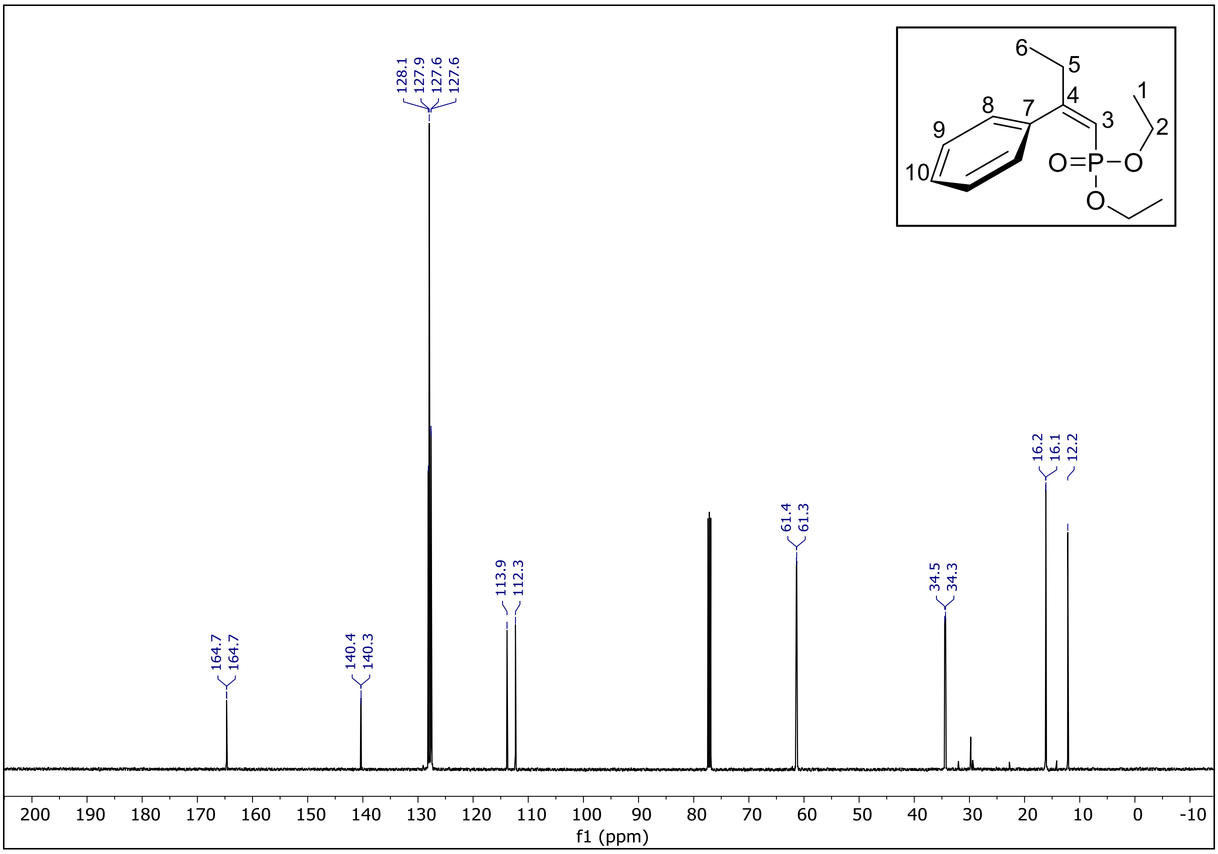

^{31}P NMR (202 MHz, $CDCl_3$): ***Z*-14**

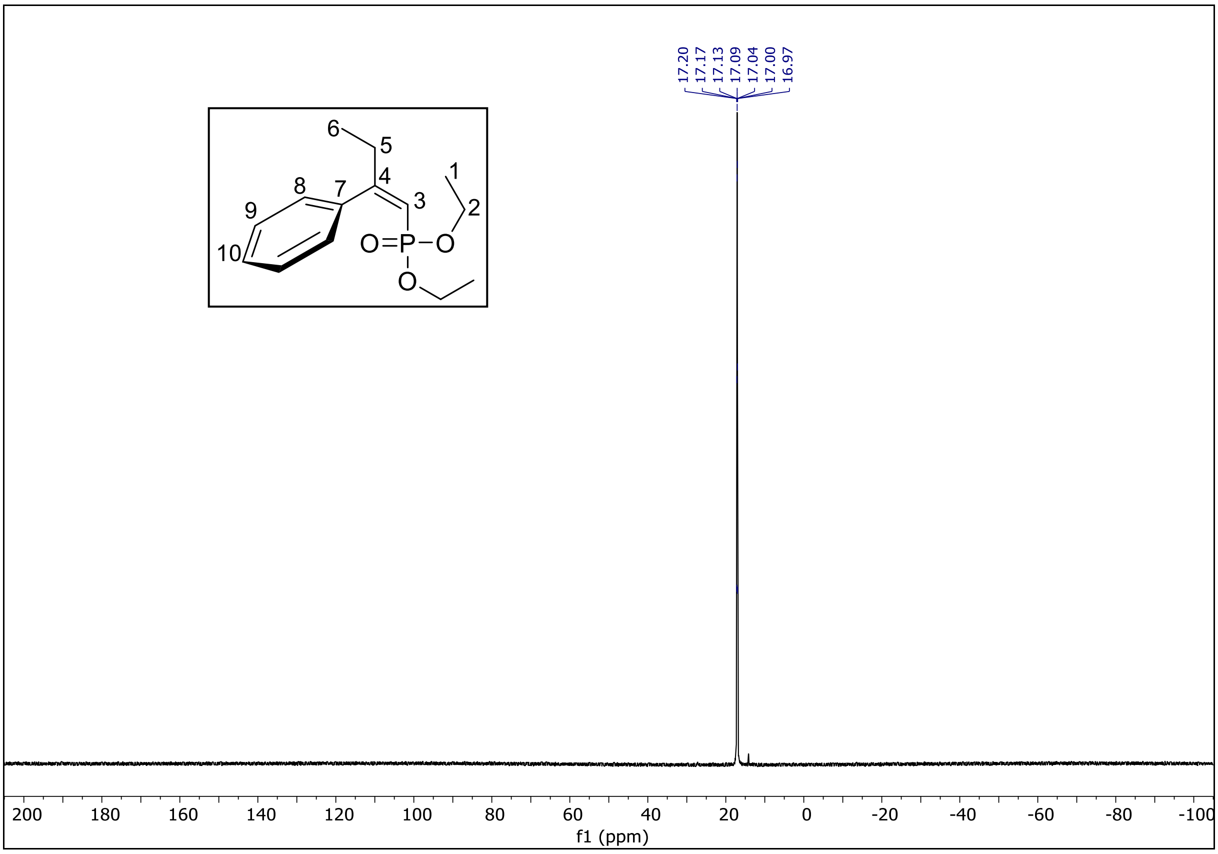

^{1}H NMR (600 MHz, $CDCl_3$): ***Z*-15**

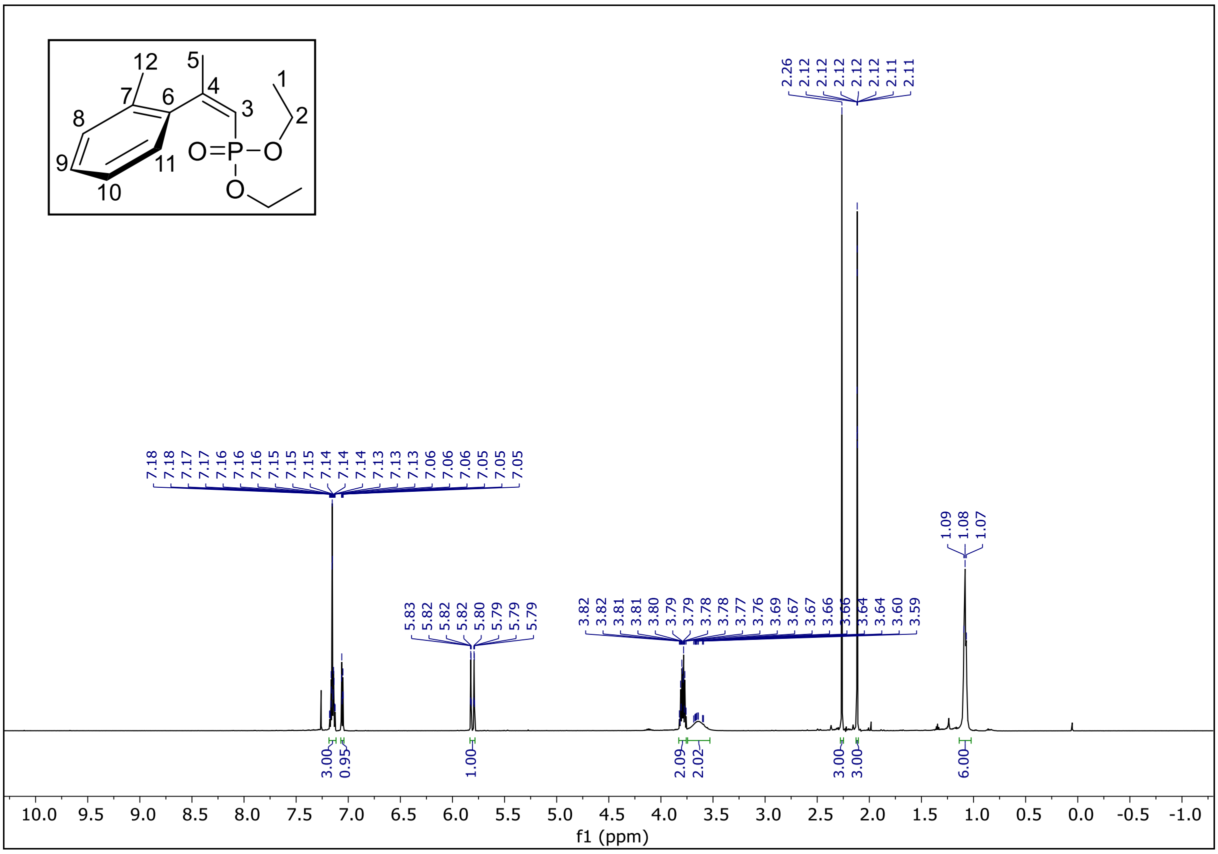

^{13}C NMR (151 MHz, $CDCl_3$): ***Z*-15**

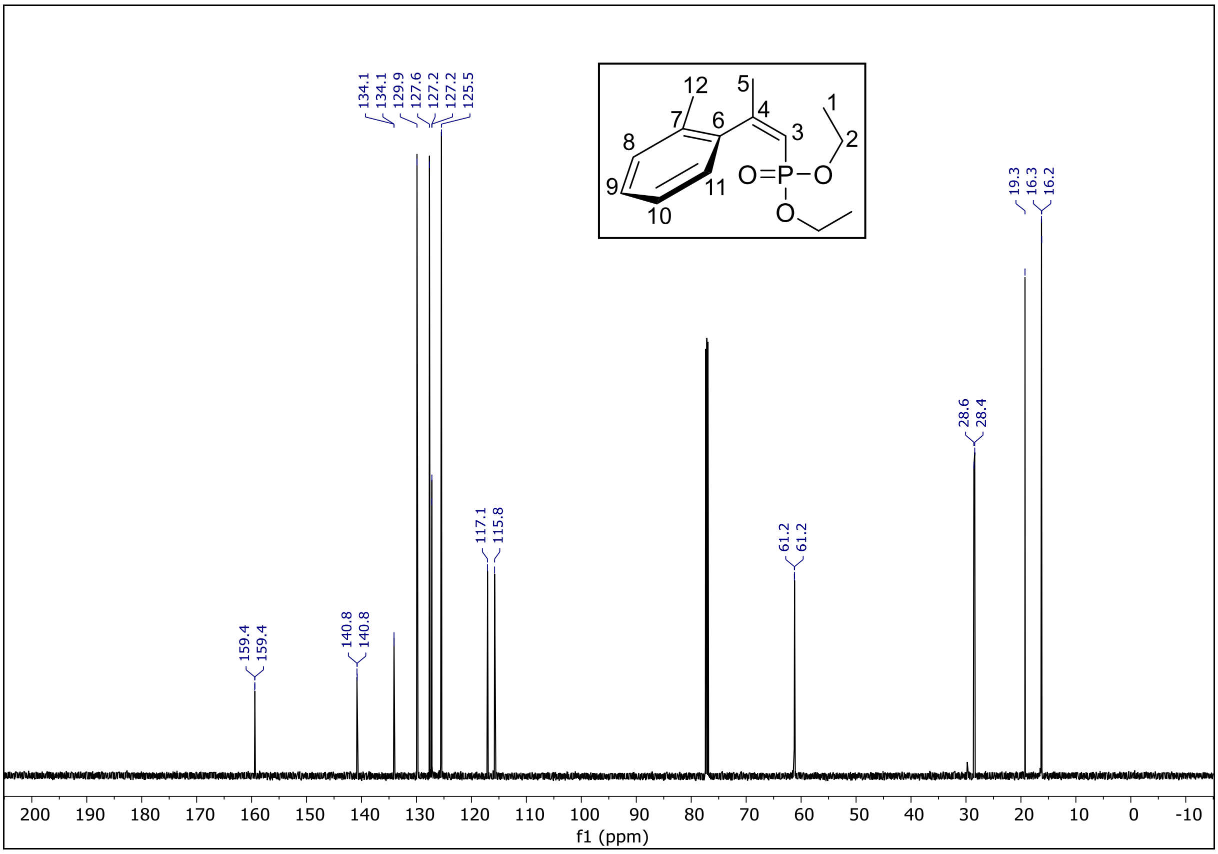

^{31}P NMR (121 MHz, $CDCl_3$): ***Z*-15**

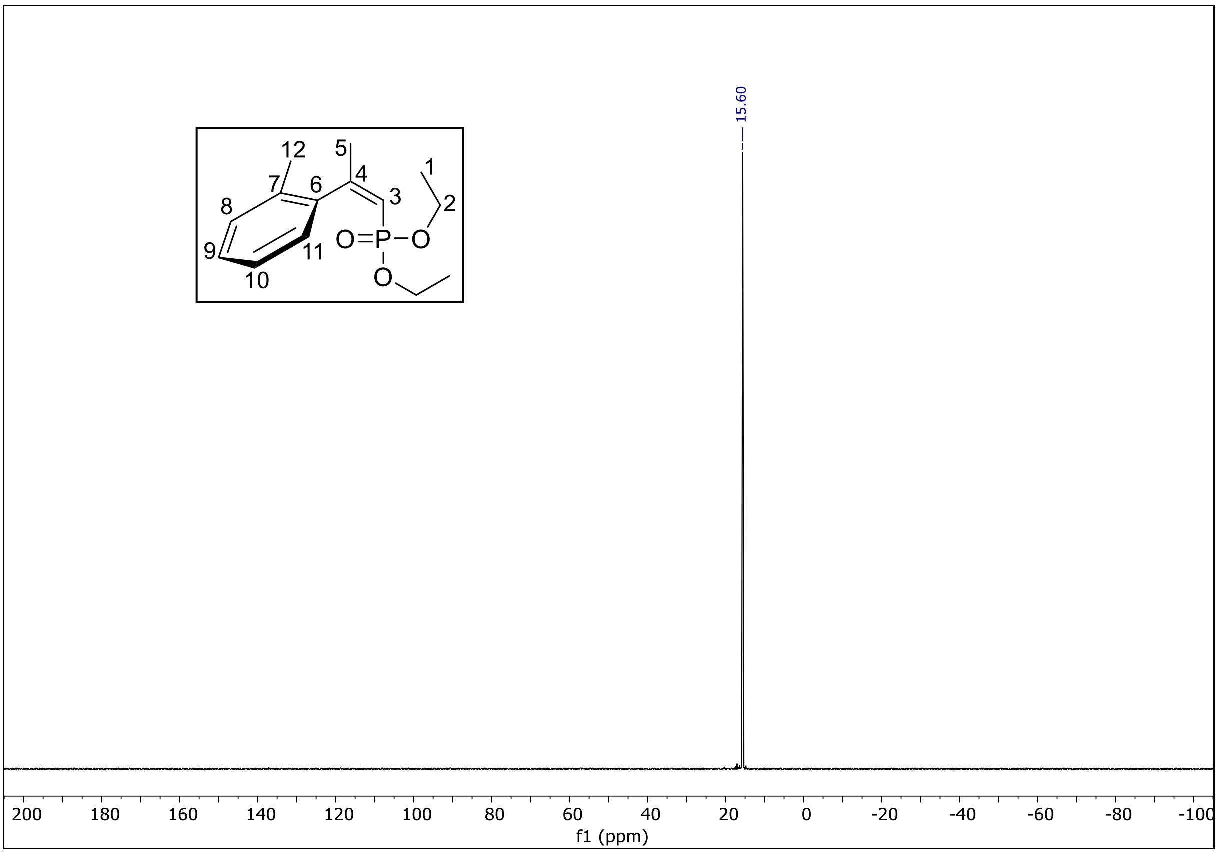

^{1}H NMR (600 MHz, $CDCl_3$): ***Z*-16**

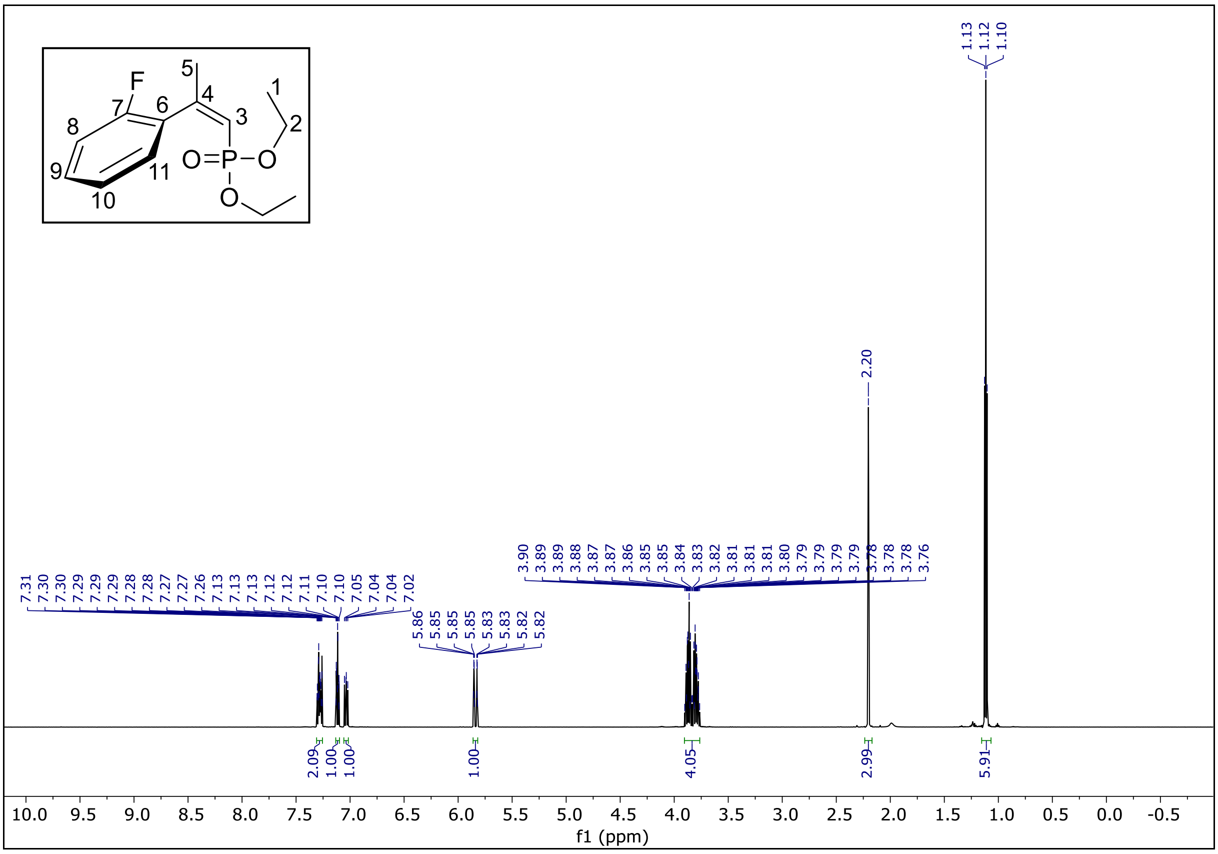

^{13}C NMR (151 MHz, $CDCl_3$): ***Z*-16**

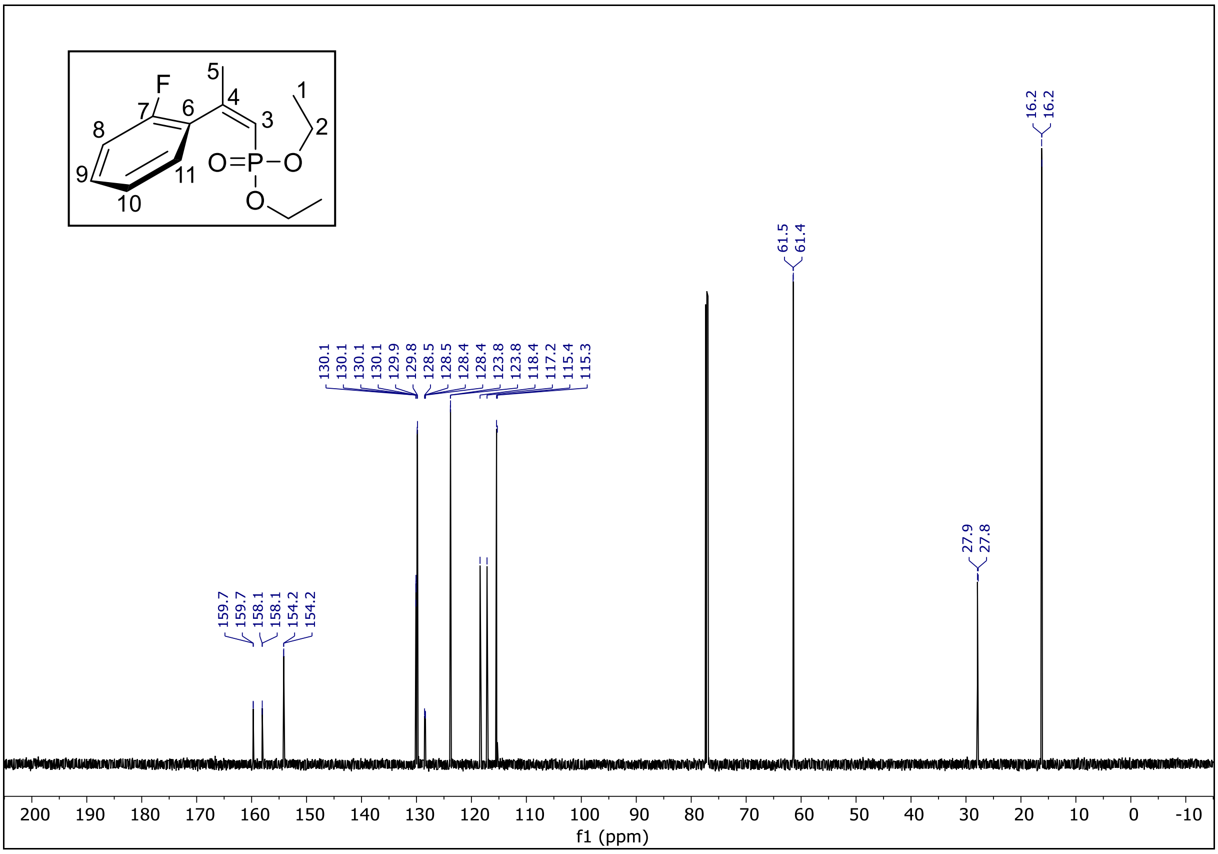

^{19}F NMR (564 MHz, $CDCl_3$): ***Z*-16**

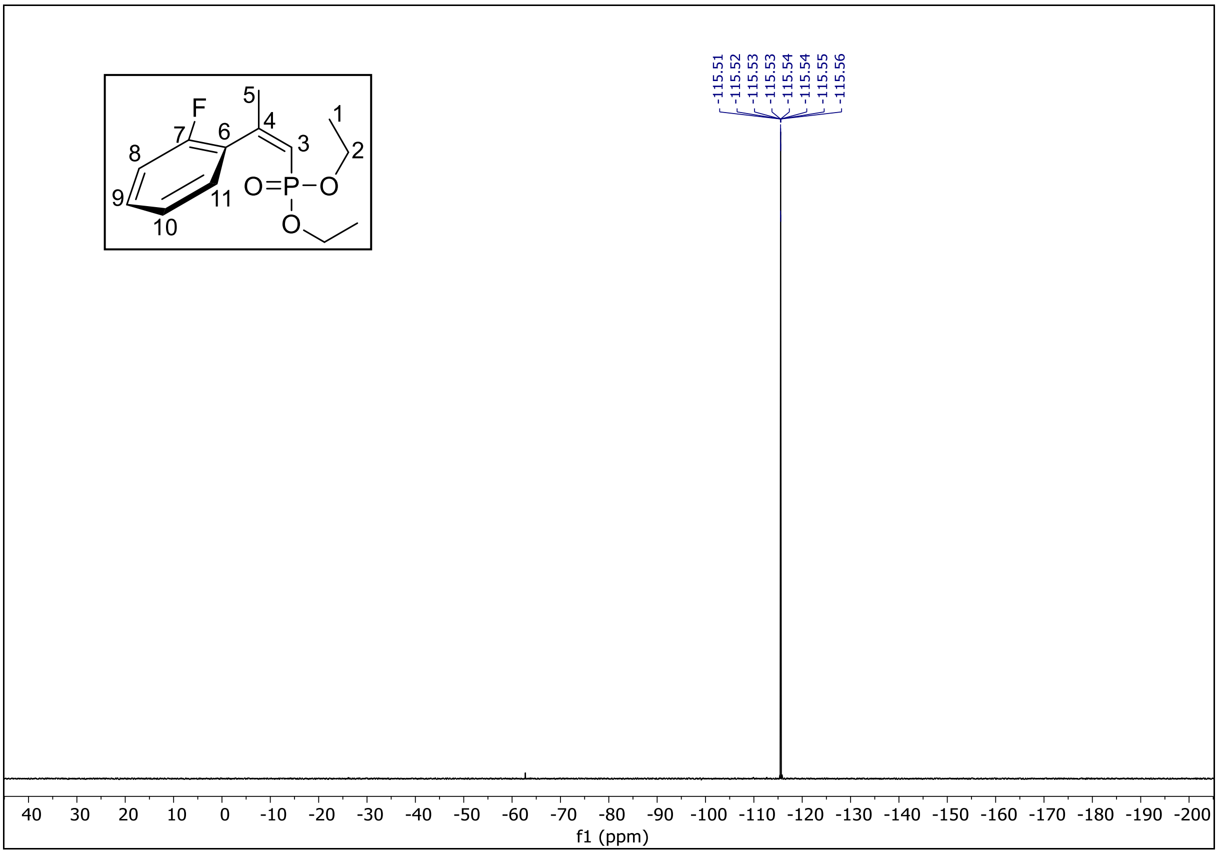

^{31}P NMR (121 MHz, $CDCl_3$): ***Z*-16**

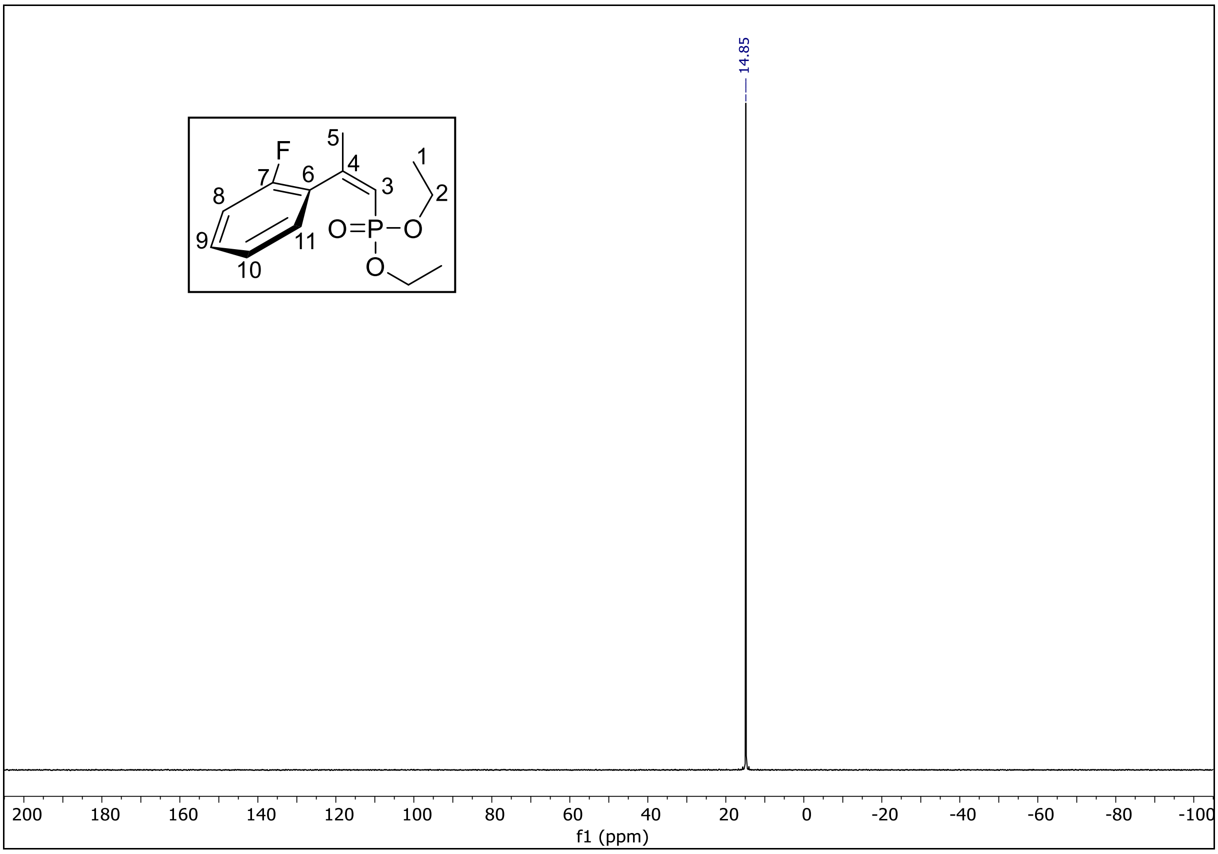

^{1}H NMR (600 MHz, CDCl$_3$): ***Z*-17**

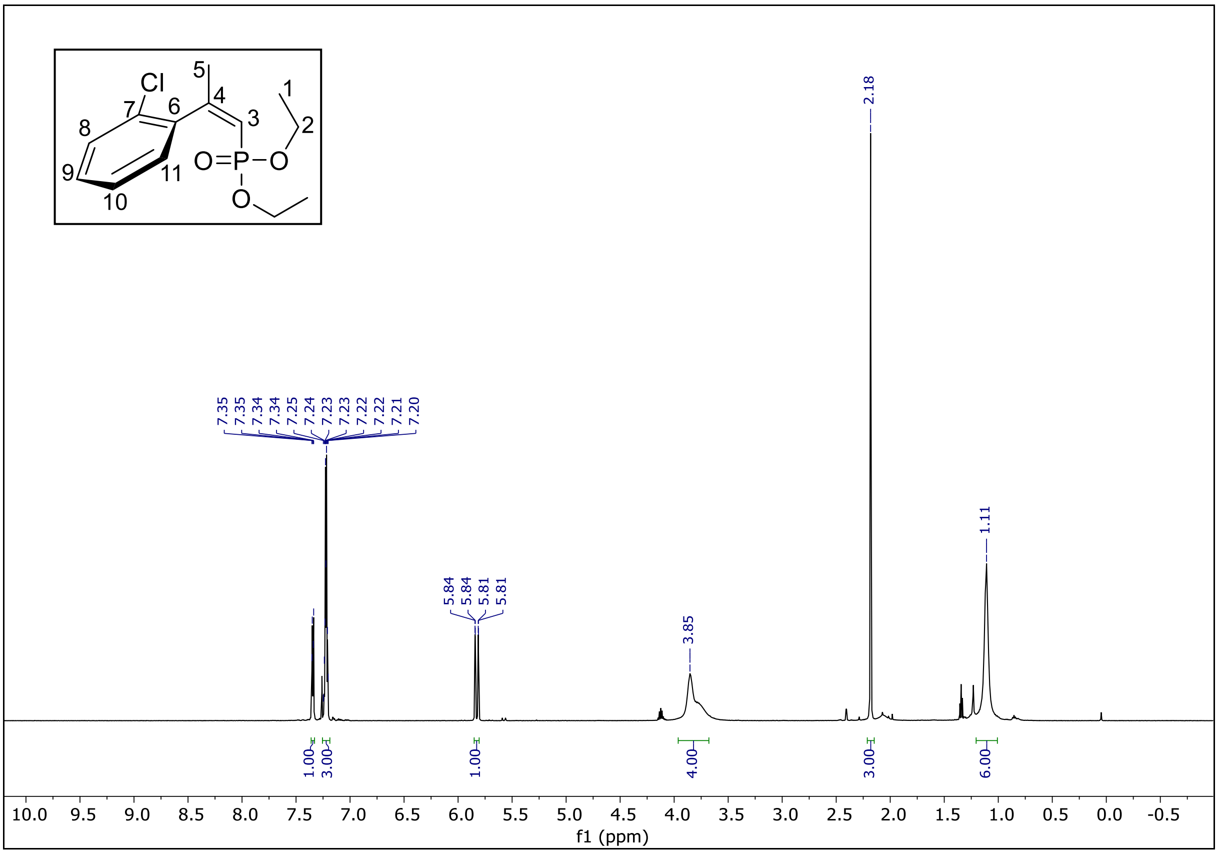

^{13}C NMR (151 MHz, CDCl$_3$): ***Z*-17**

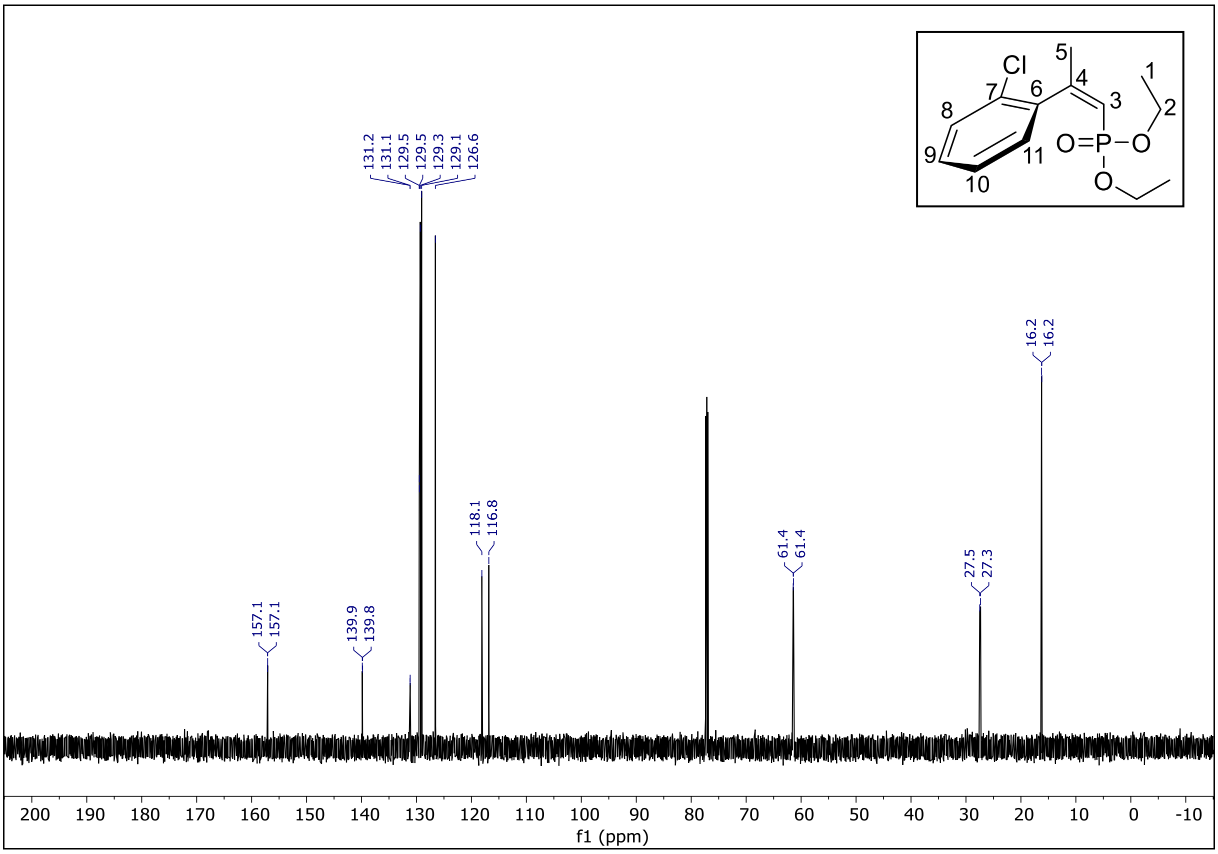

^{31}P NMR (121 MHz, $CDCl_3$): ***Z*-17**

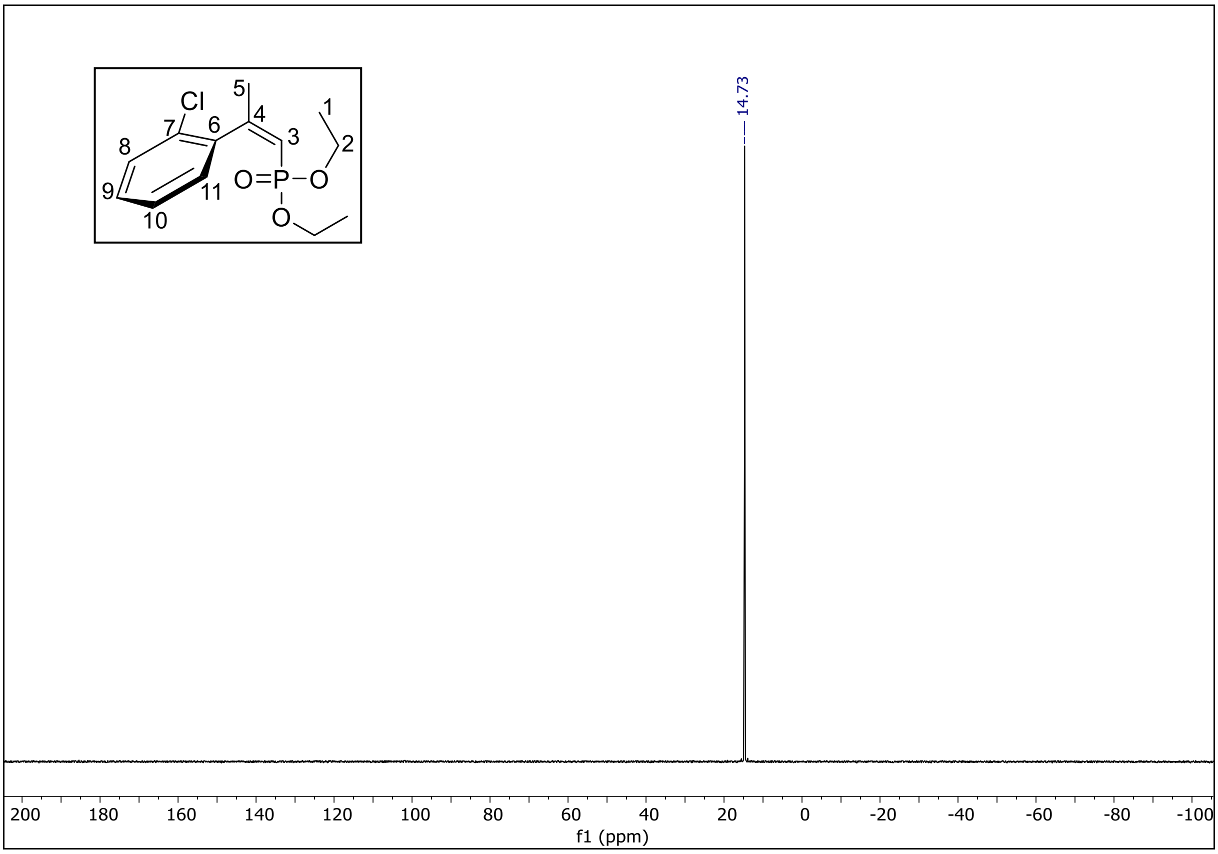

^{1}H NMR (500 MHz, $CDCl_3$): ***Z*-18**

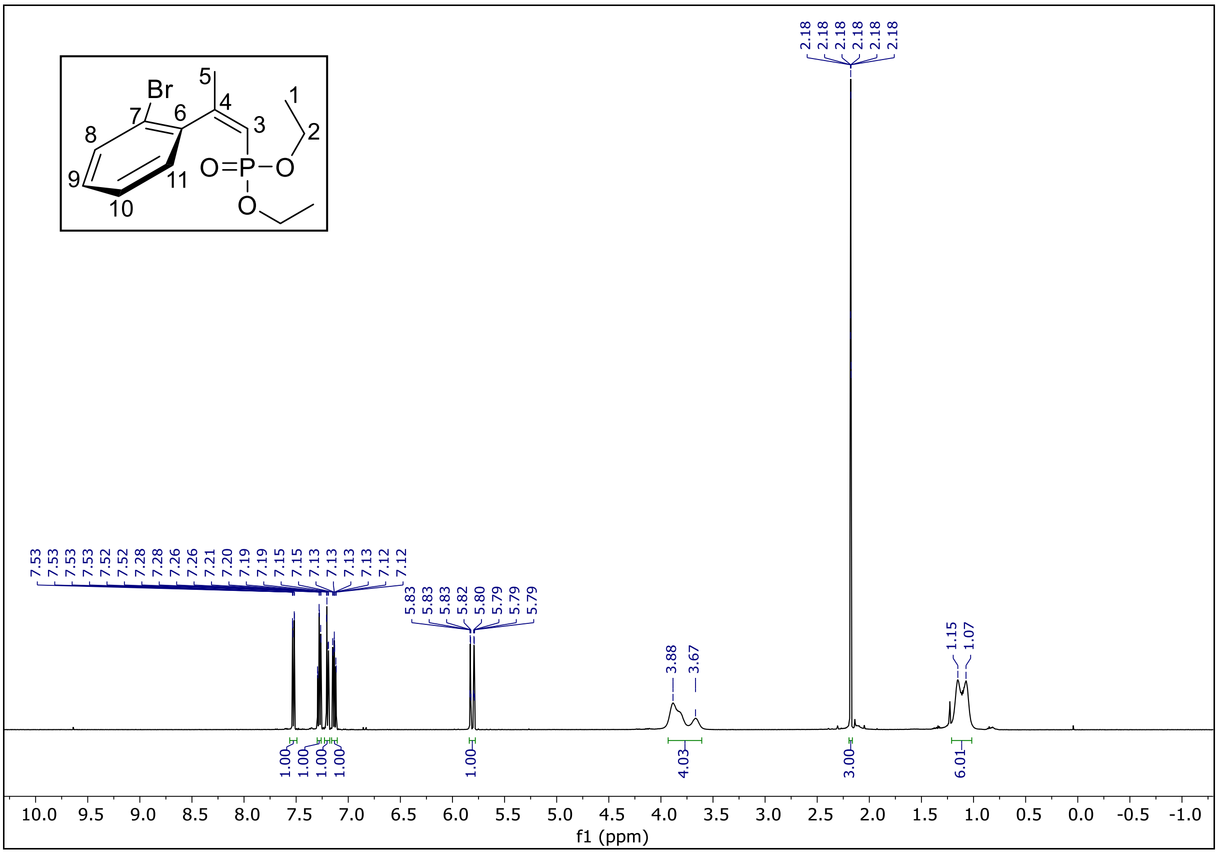

^{13}C NMR (126 MHz, $CDCl_3$): ***Z*-18**

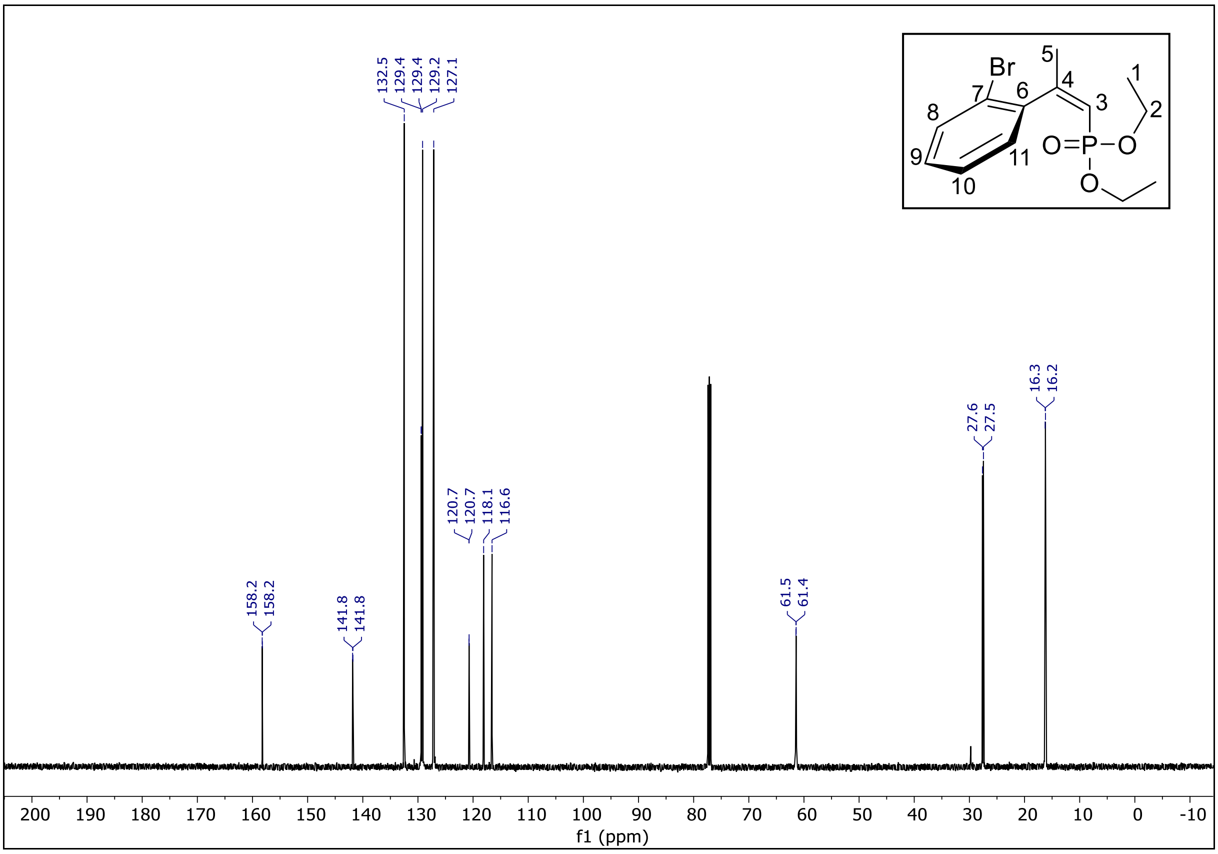

^{31}P NMR (243 MHz, $CDCl_3$): ***Z*-18**

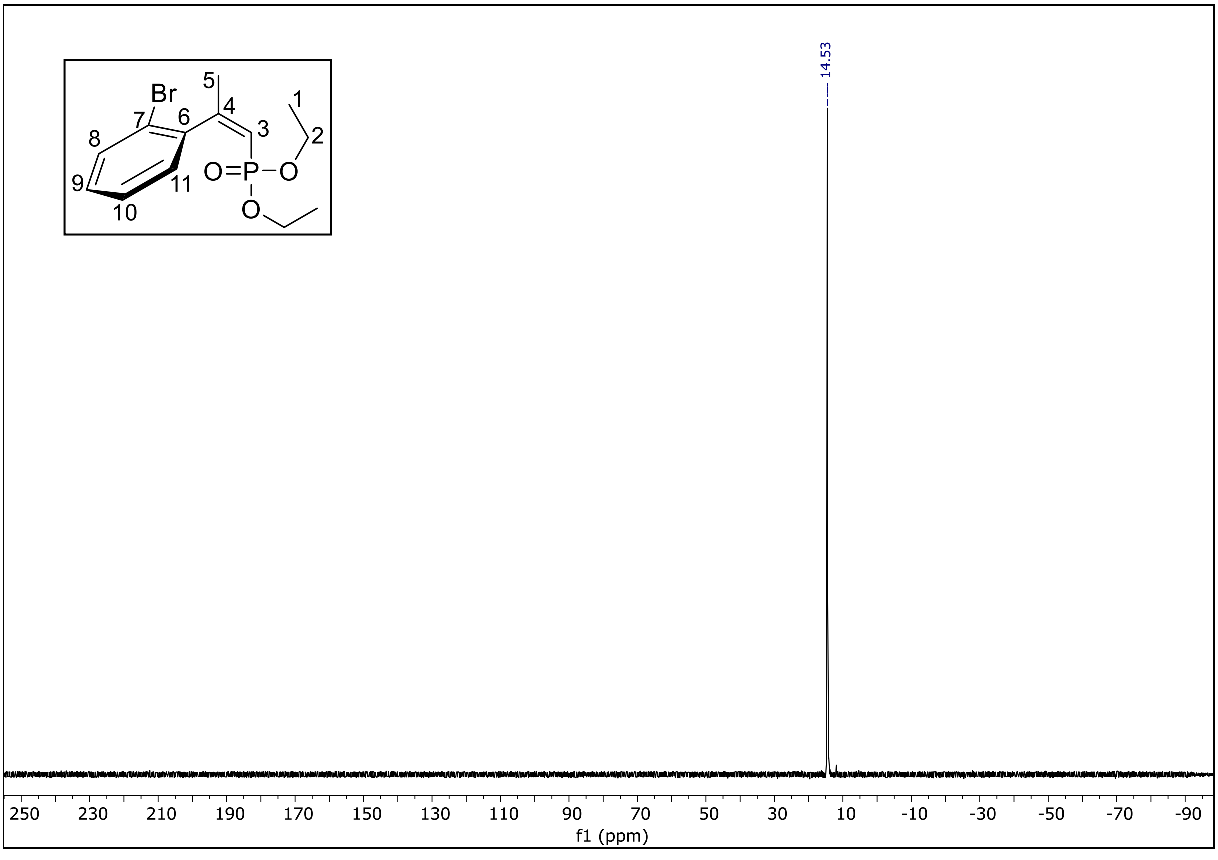

^{1}H NMR (400 MHz, $CDCl_3$): **19**

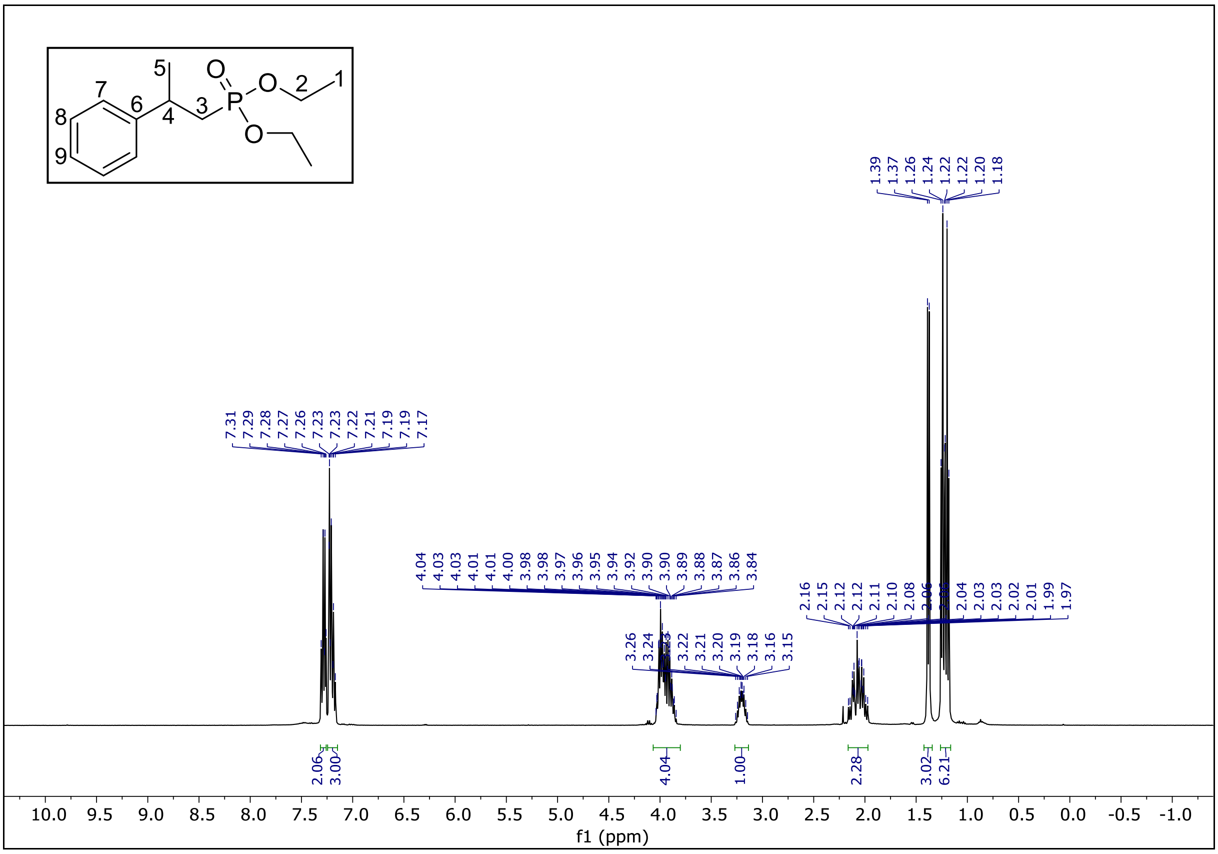

^{13}C NMR (101 MHz, $CDCl_3$): **19**

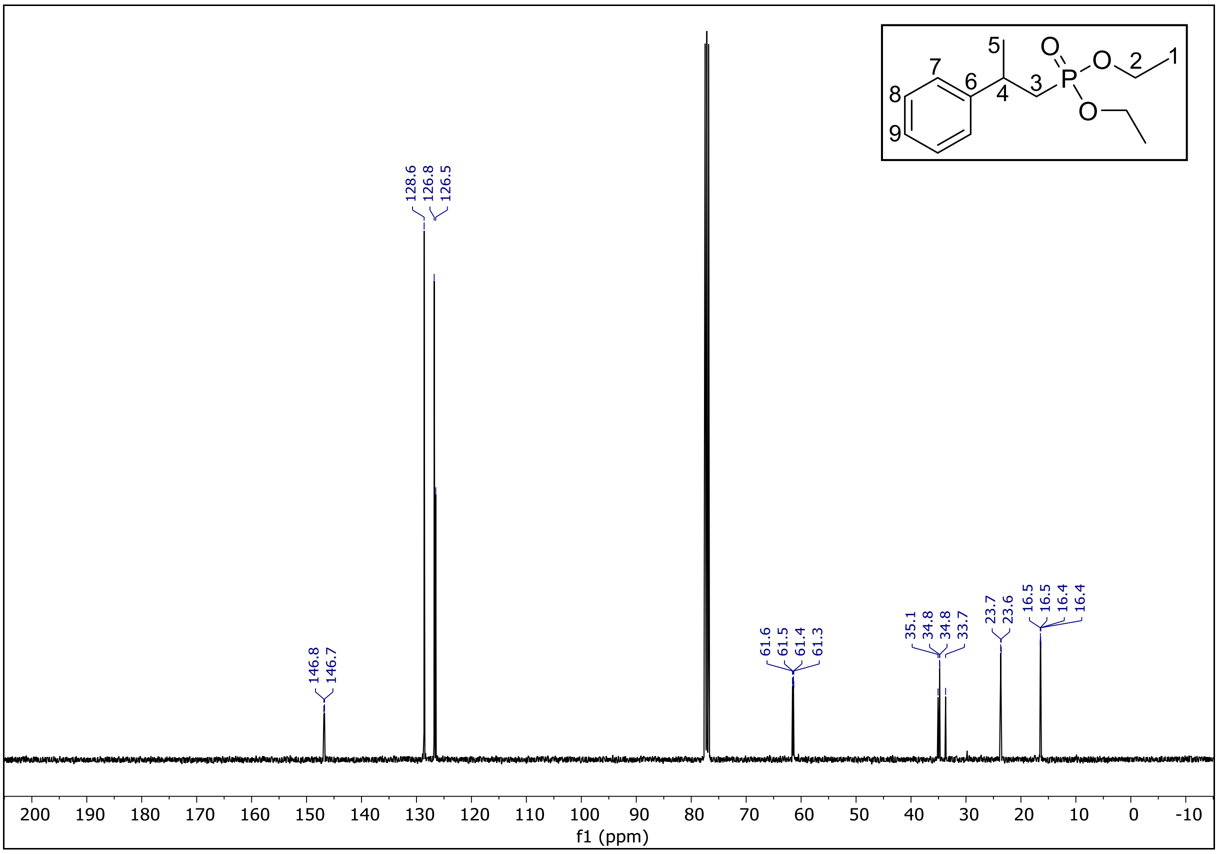

^{31}P NMR (162 MHz, $CDCl_3$): **19**

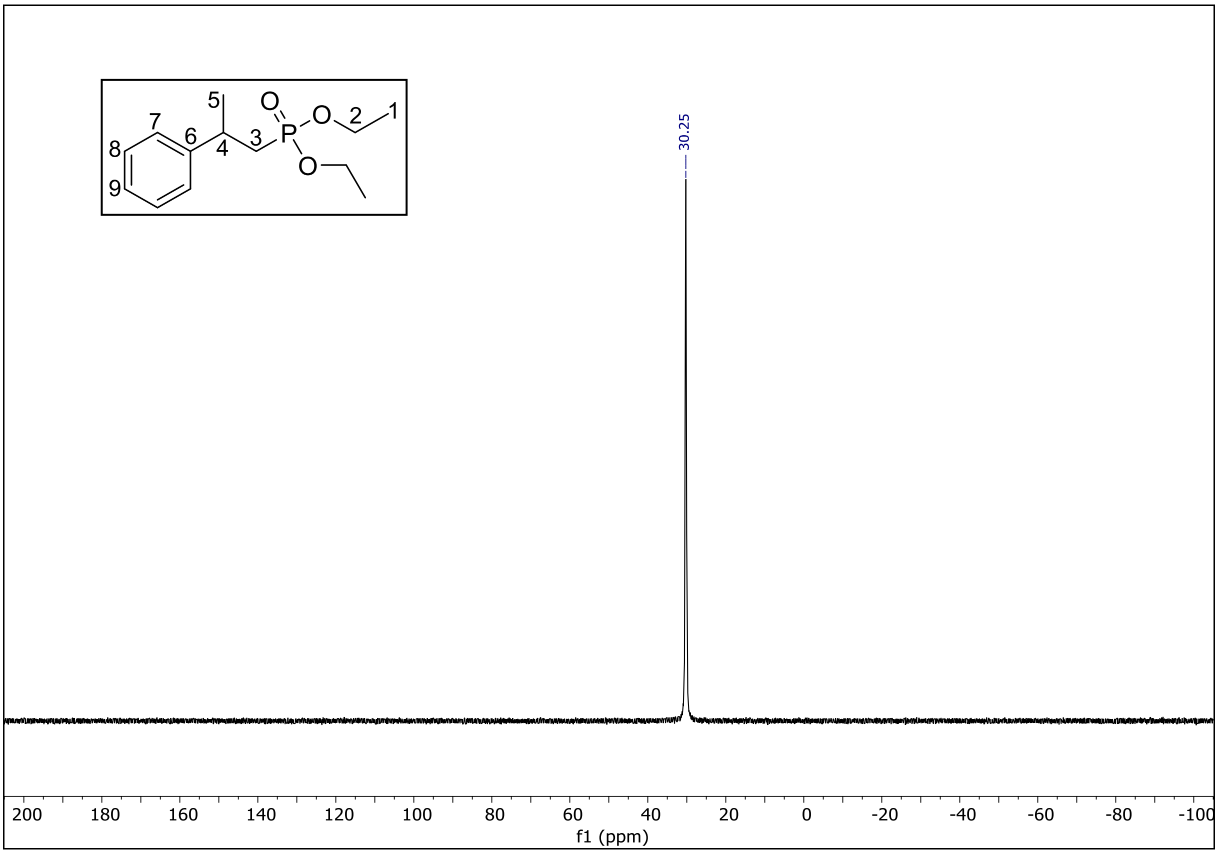

^{1}H NMR (500 MHz, $CDCl_3$): **20**

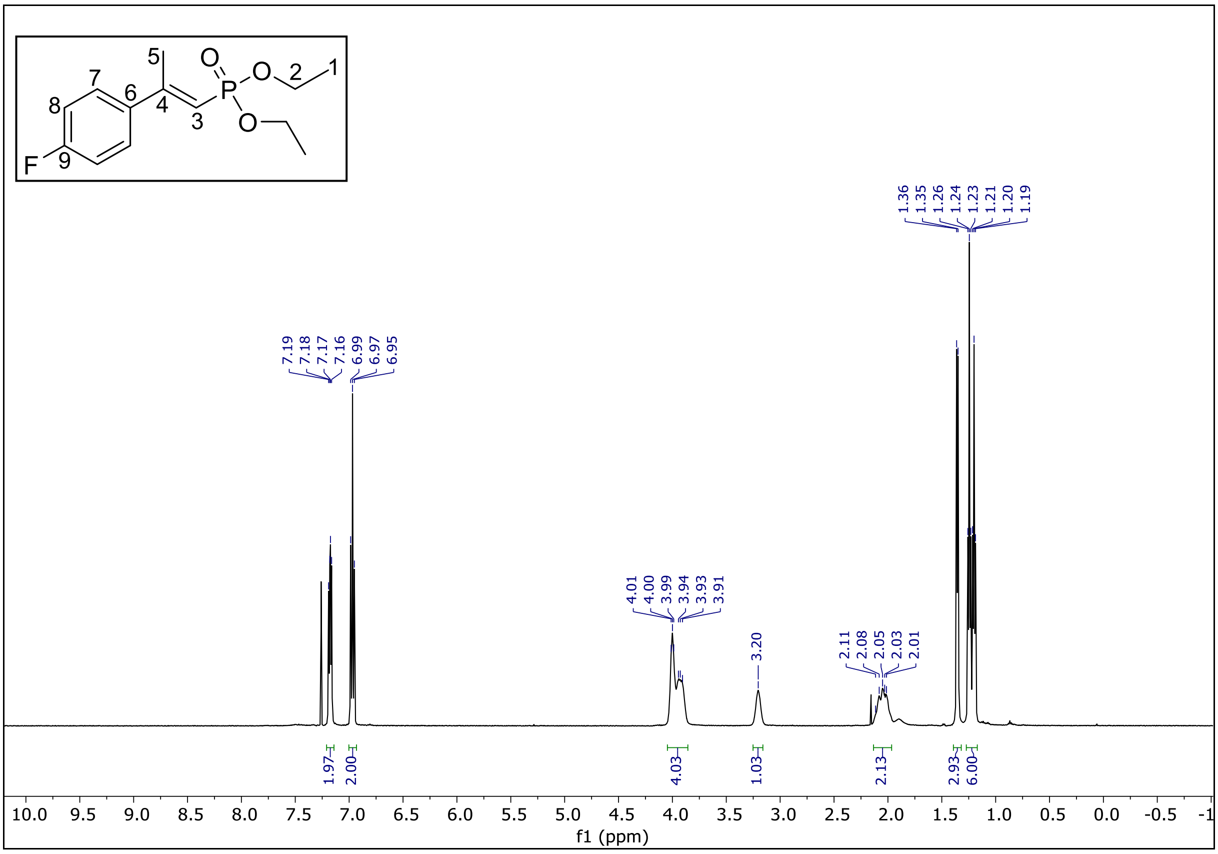

^{13}C NMR (126 MHz, $CDCl_3$): **20**

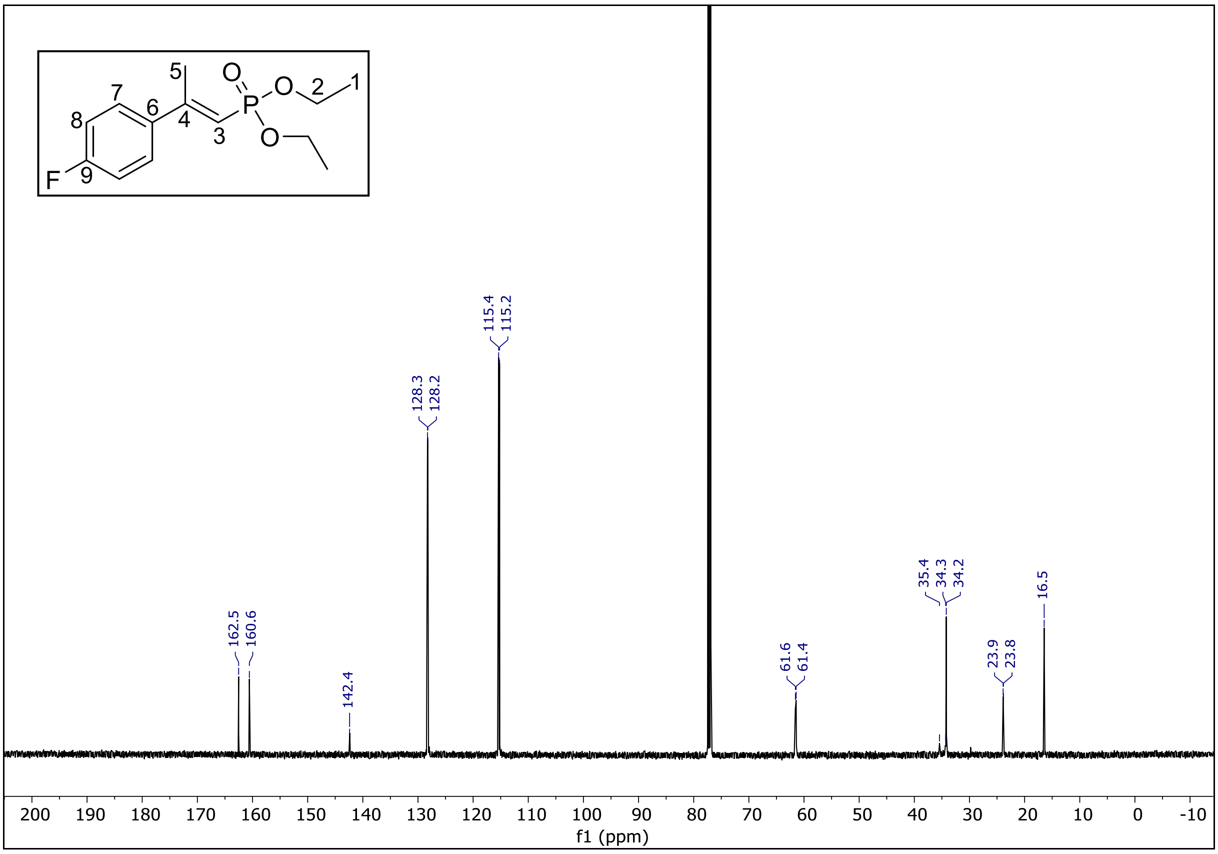

^{19}F NMR (162 MHz, $CDCl_3$): **20**

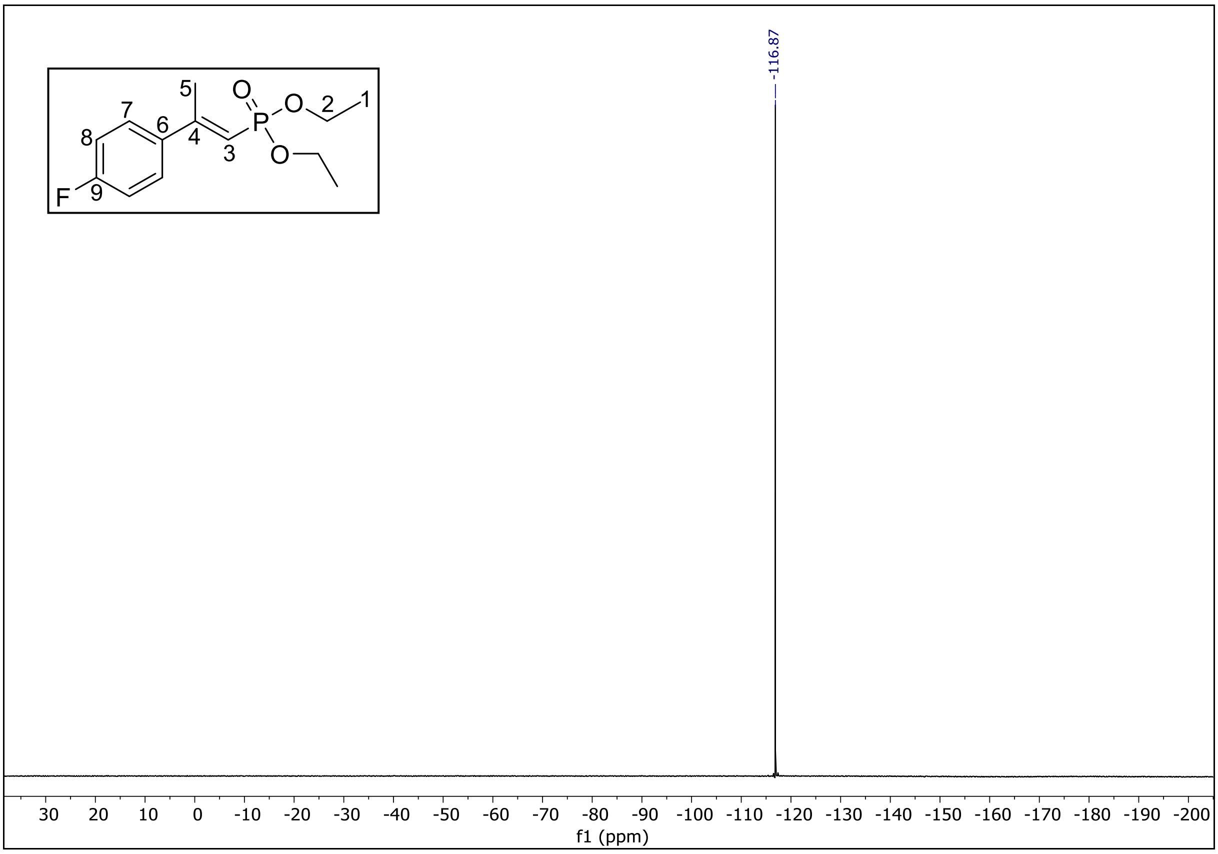

^{31}P NMR (162 MHz, $CDCl_3$): **20**

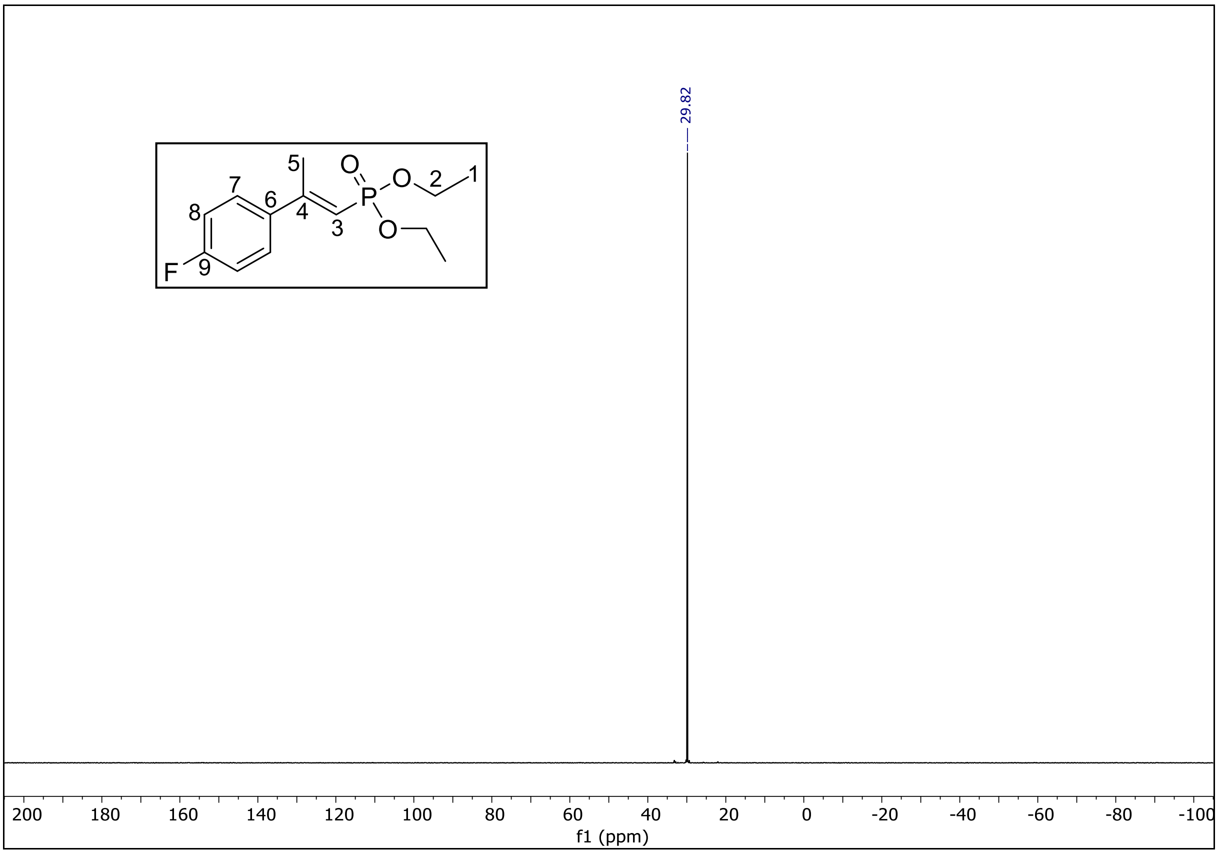

^{1}H NMR (500 MHz, $CDCl_3$): **21**

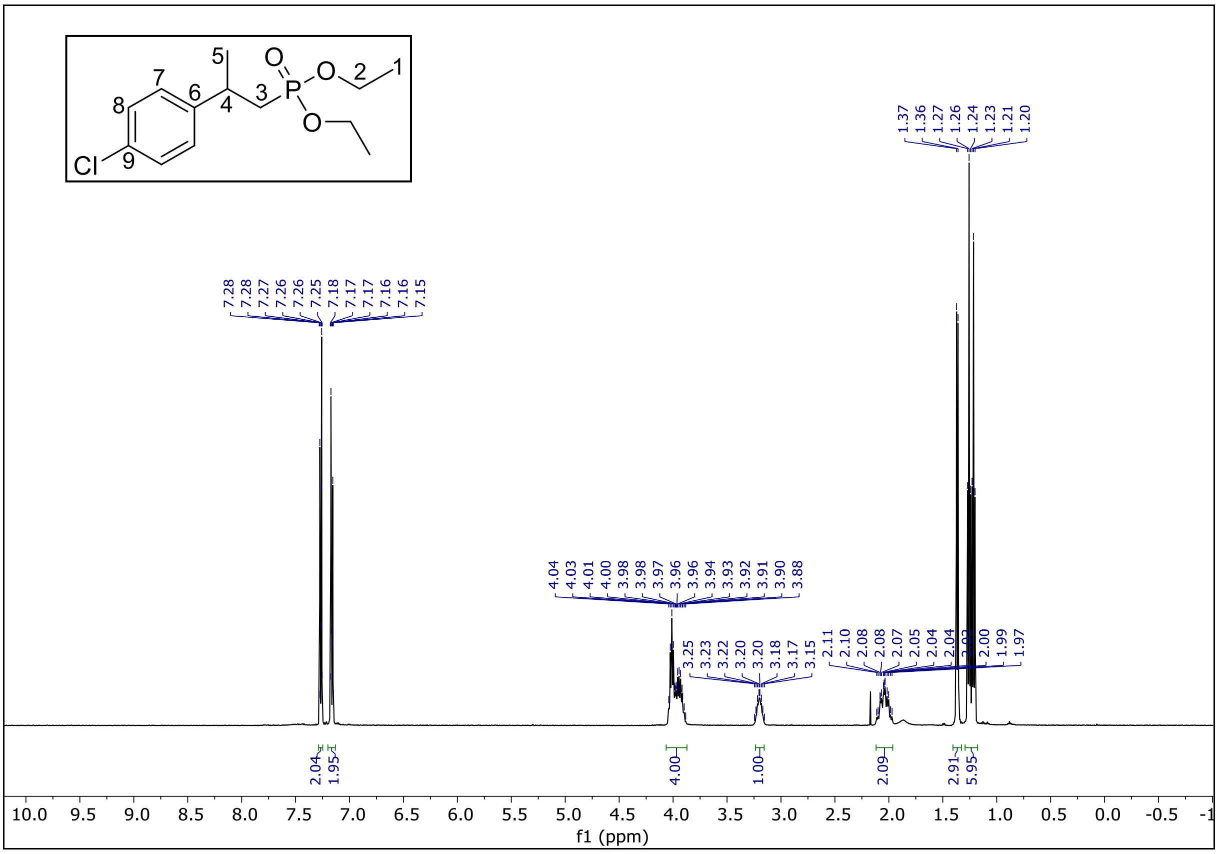

^{13}C NMR (126 MHz, $CDCl_3$): **21**

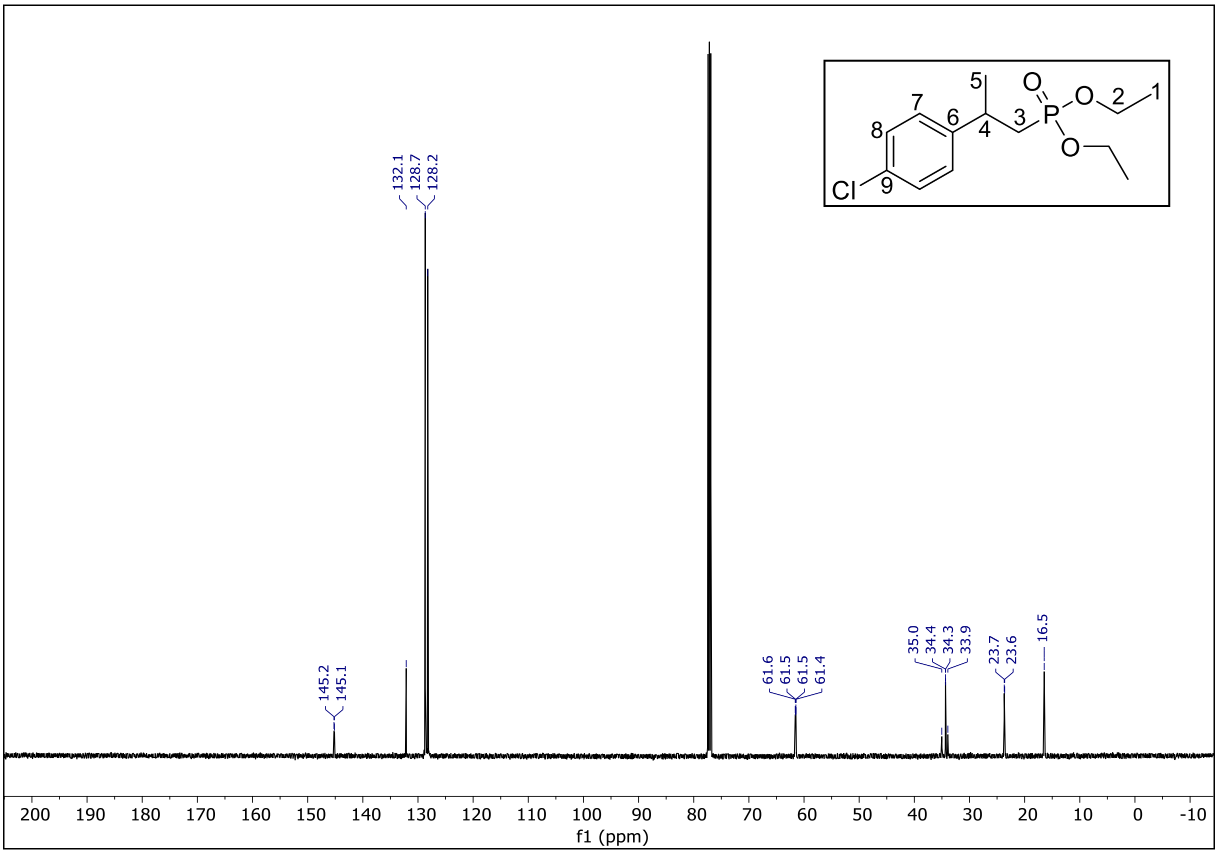

^{31}P NMR (162 MHz, $CDCl_3$): **21**

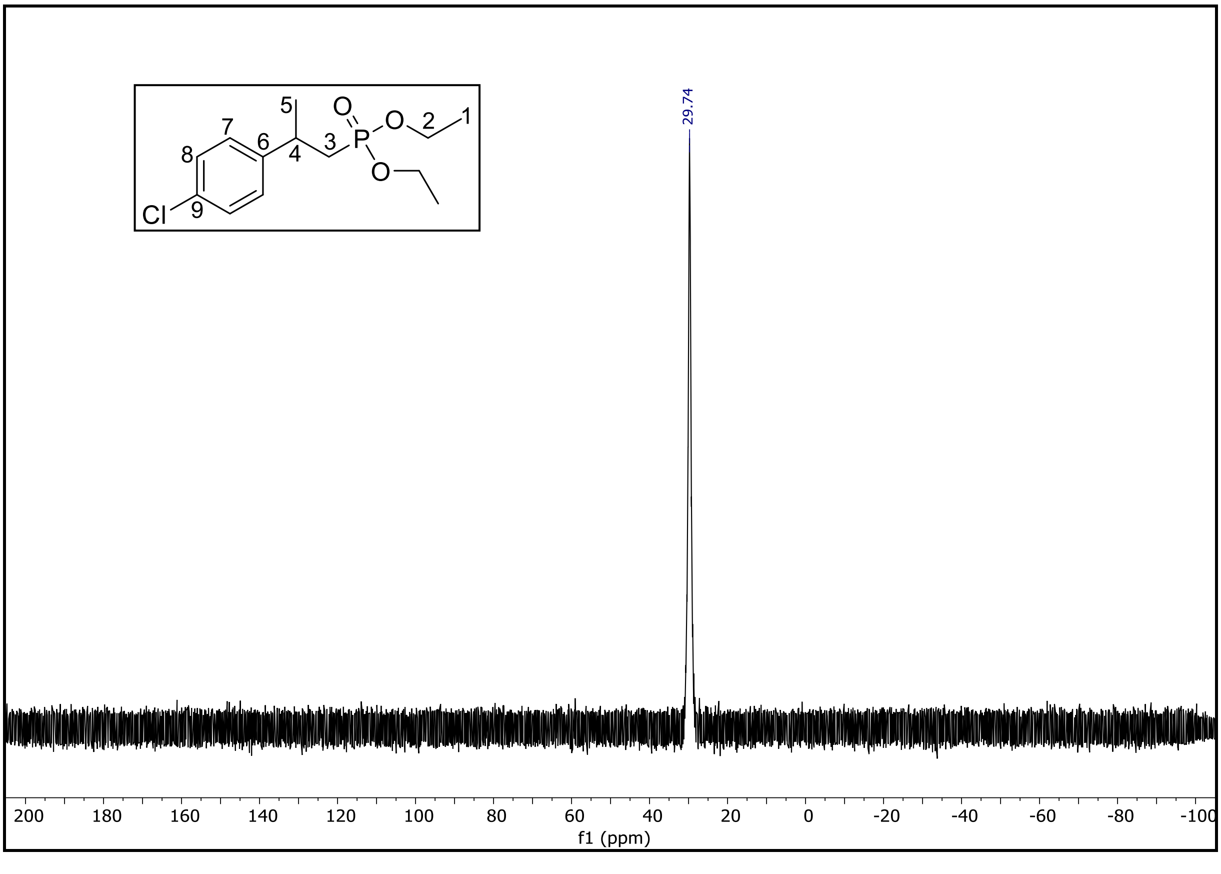

^{1}H NMR (500 MHz, $CDCl_3$): **22**

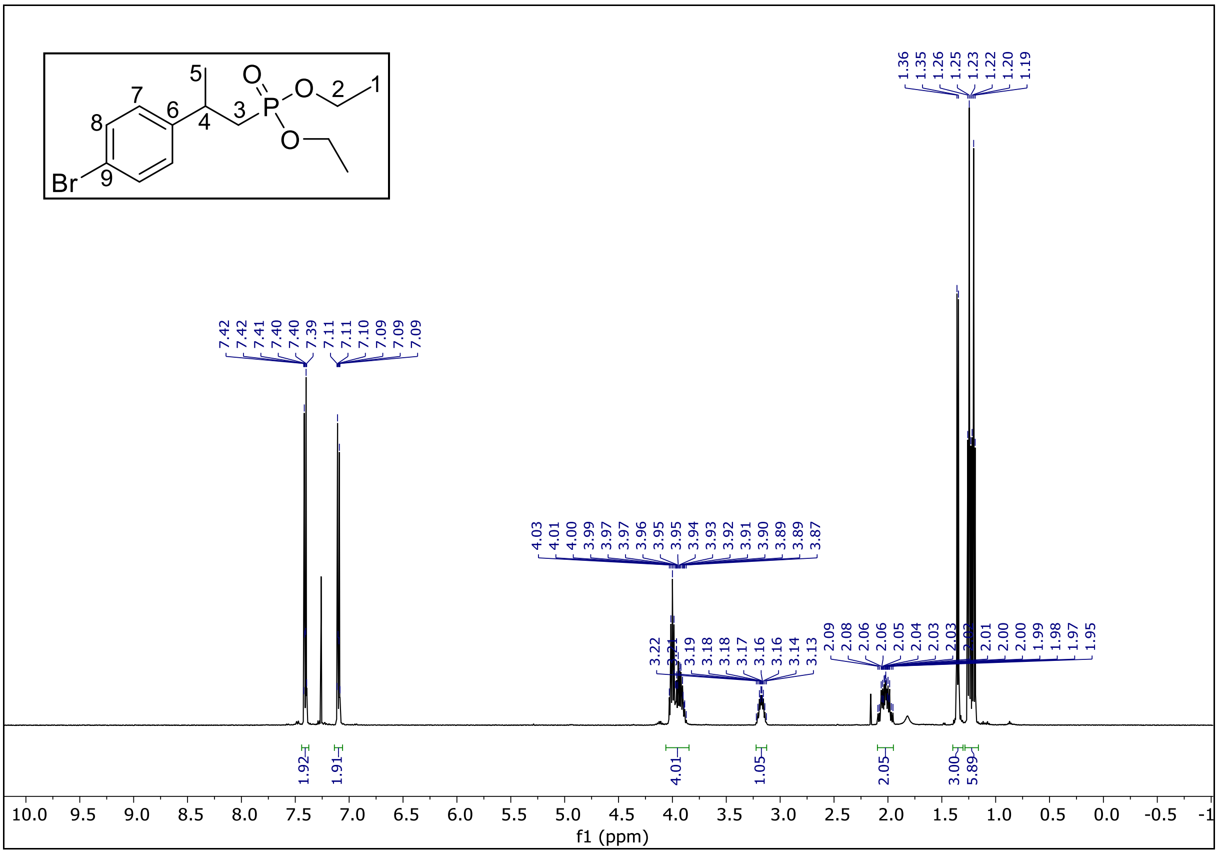

^{13}C NMR (126 MHz, $CDCl_3$): **22**

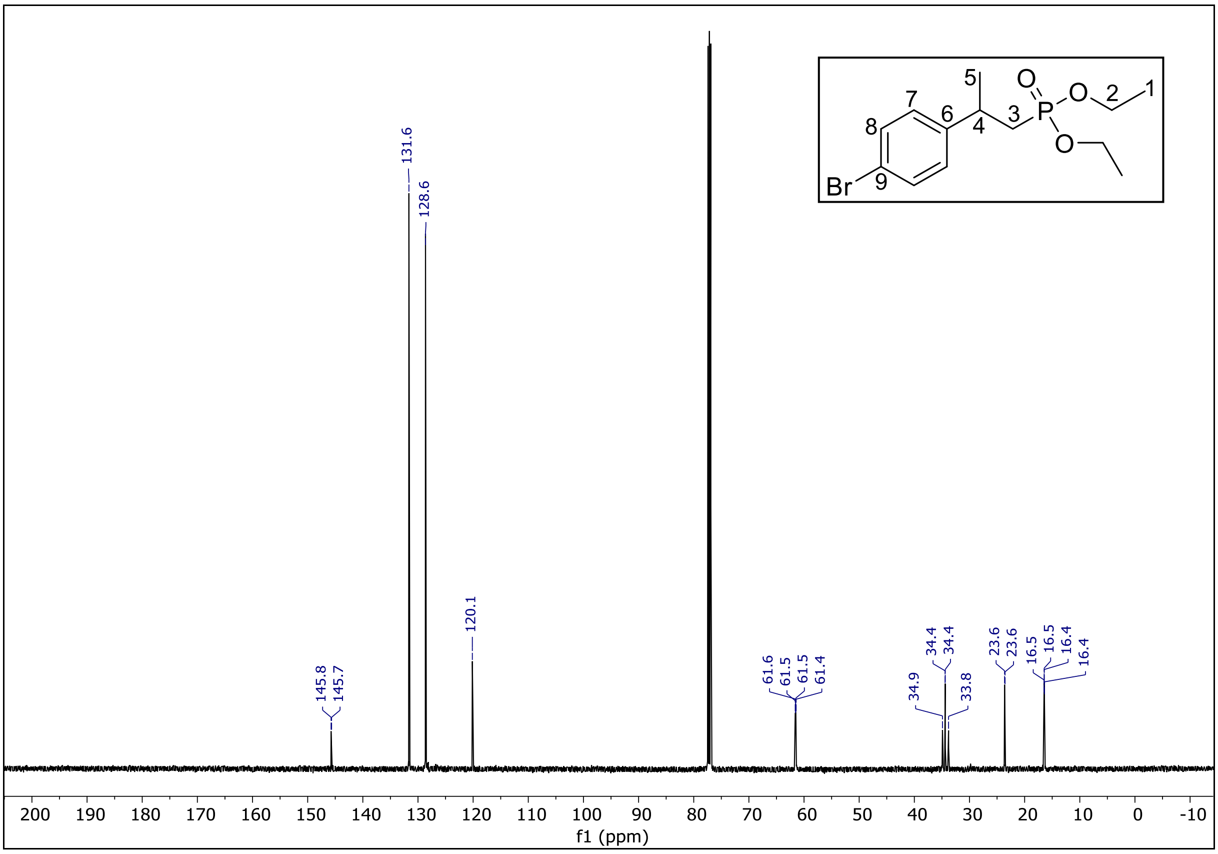

^{31}P NMR (162 MHz, $CDCl_3$): **22**

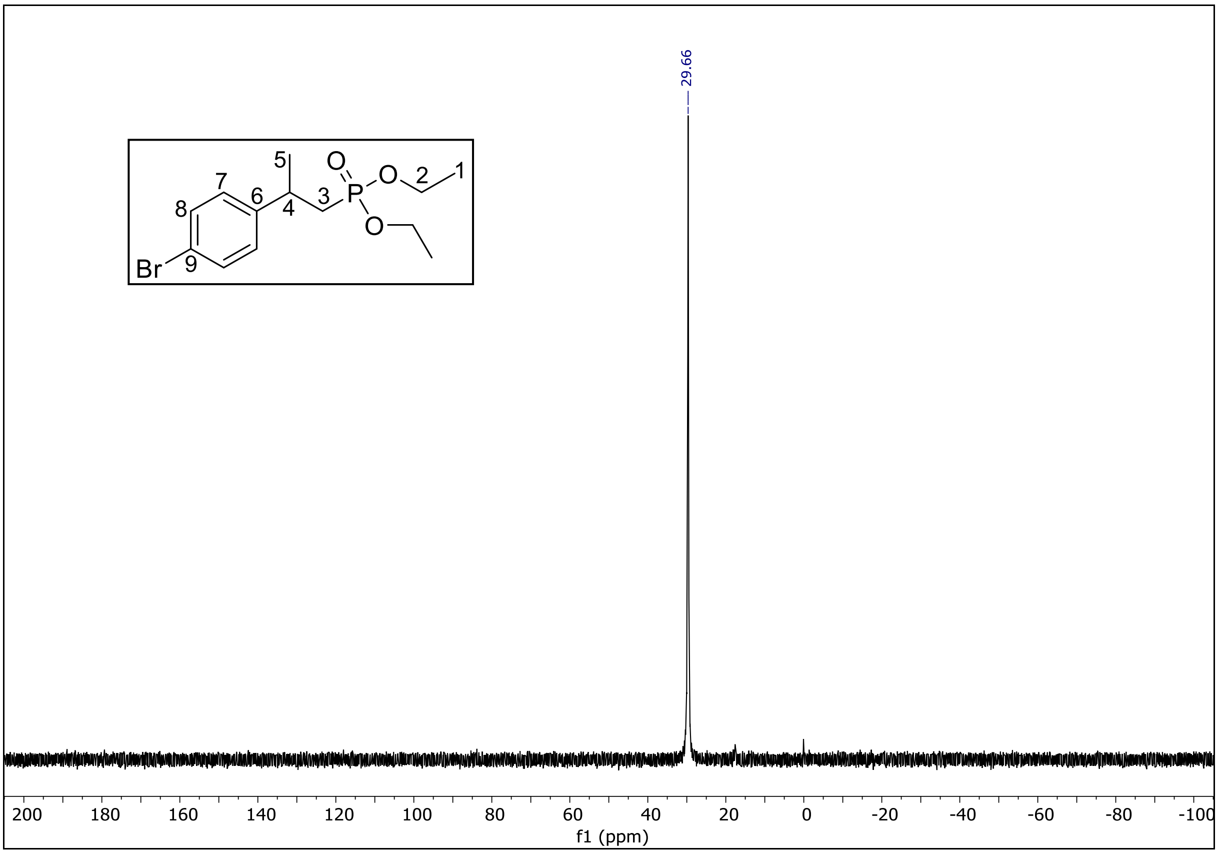

^{1}H NMR (500 MHz, $CDCl_3$): **23**

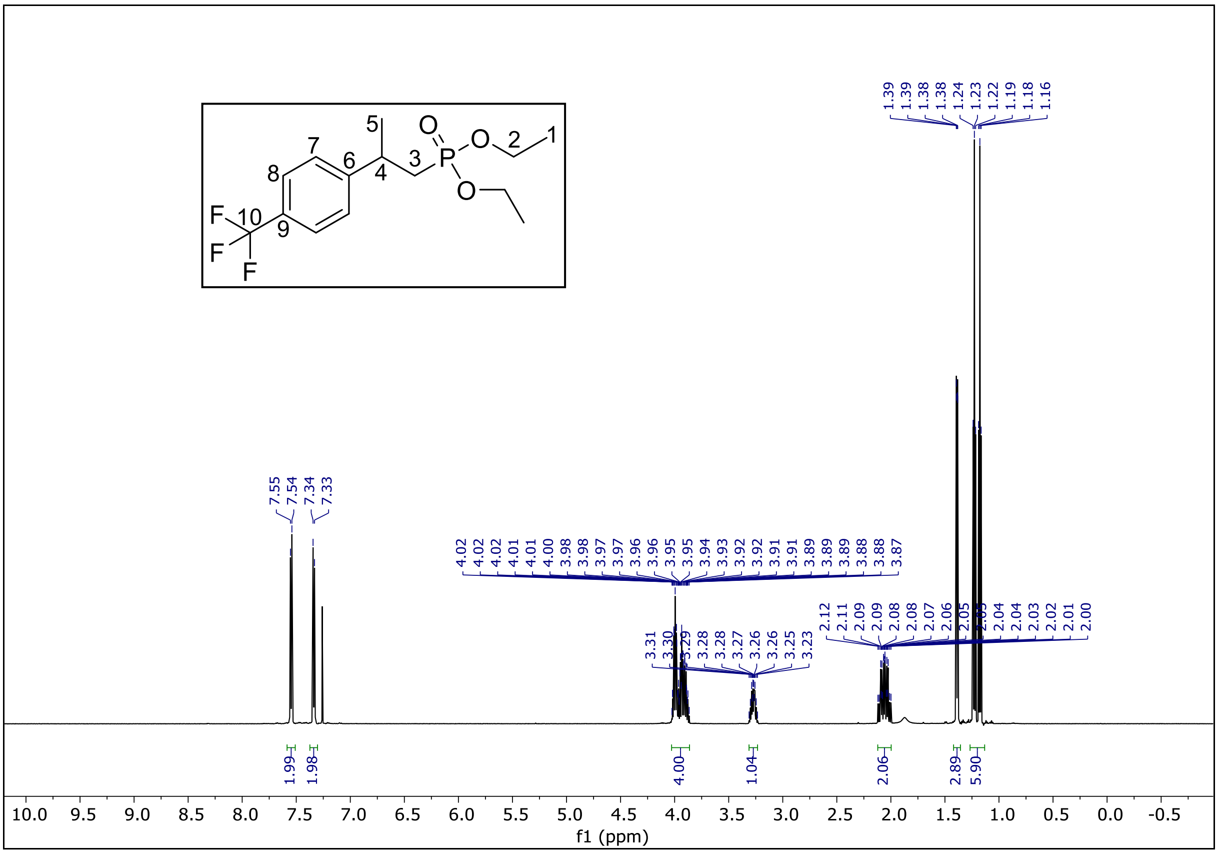

^{13}C NMR (151 MHz, $CDCl_3$): **23**

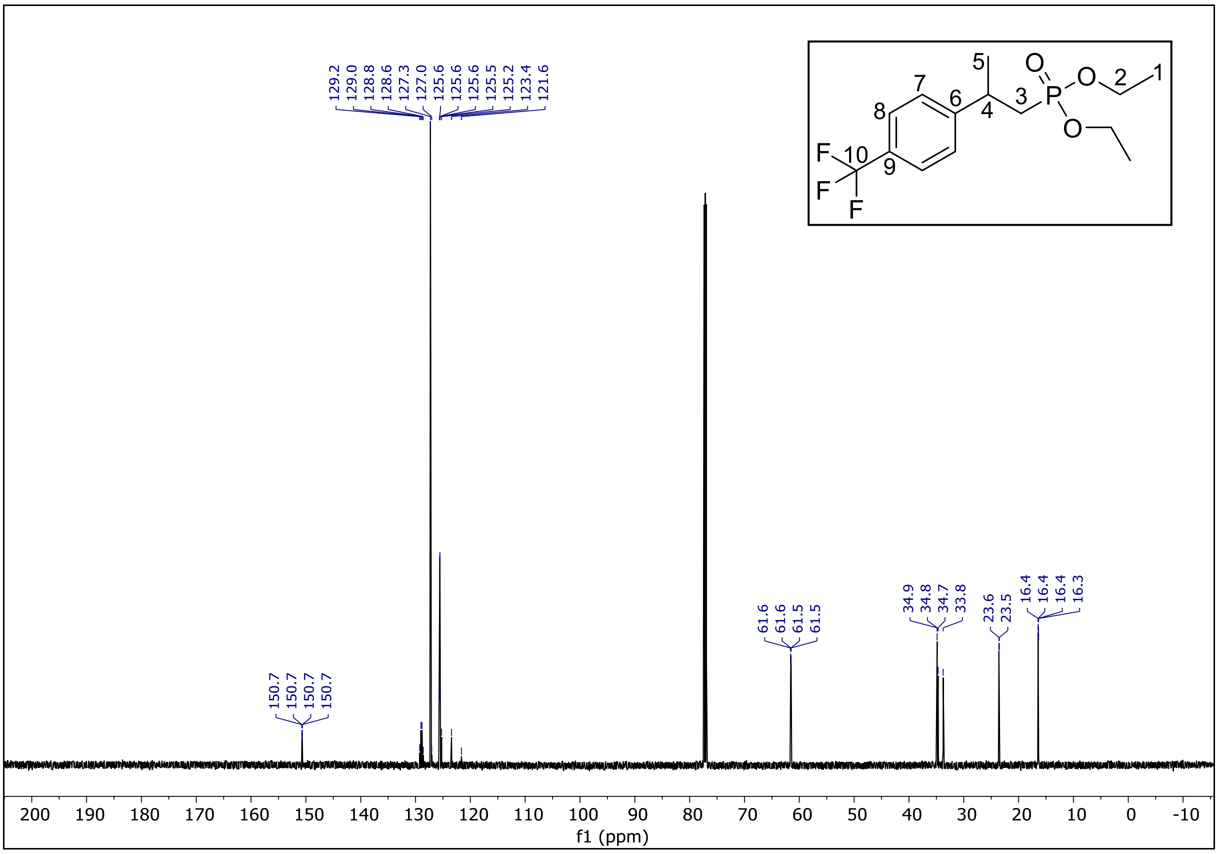

^{19}F NMR (564 MHz, $CDCl_3$): **23**

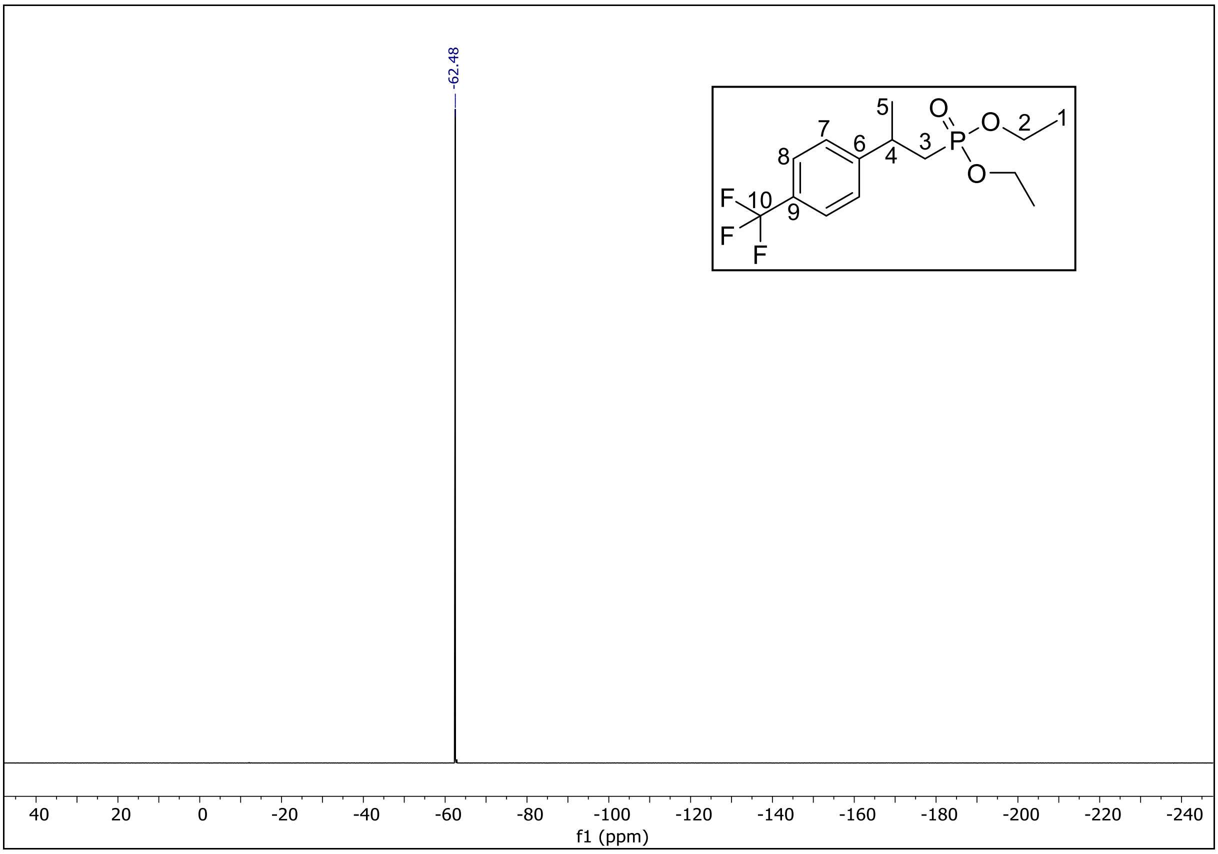

^{31}P NMR (121 MHz, $CDCl_3$): **23**

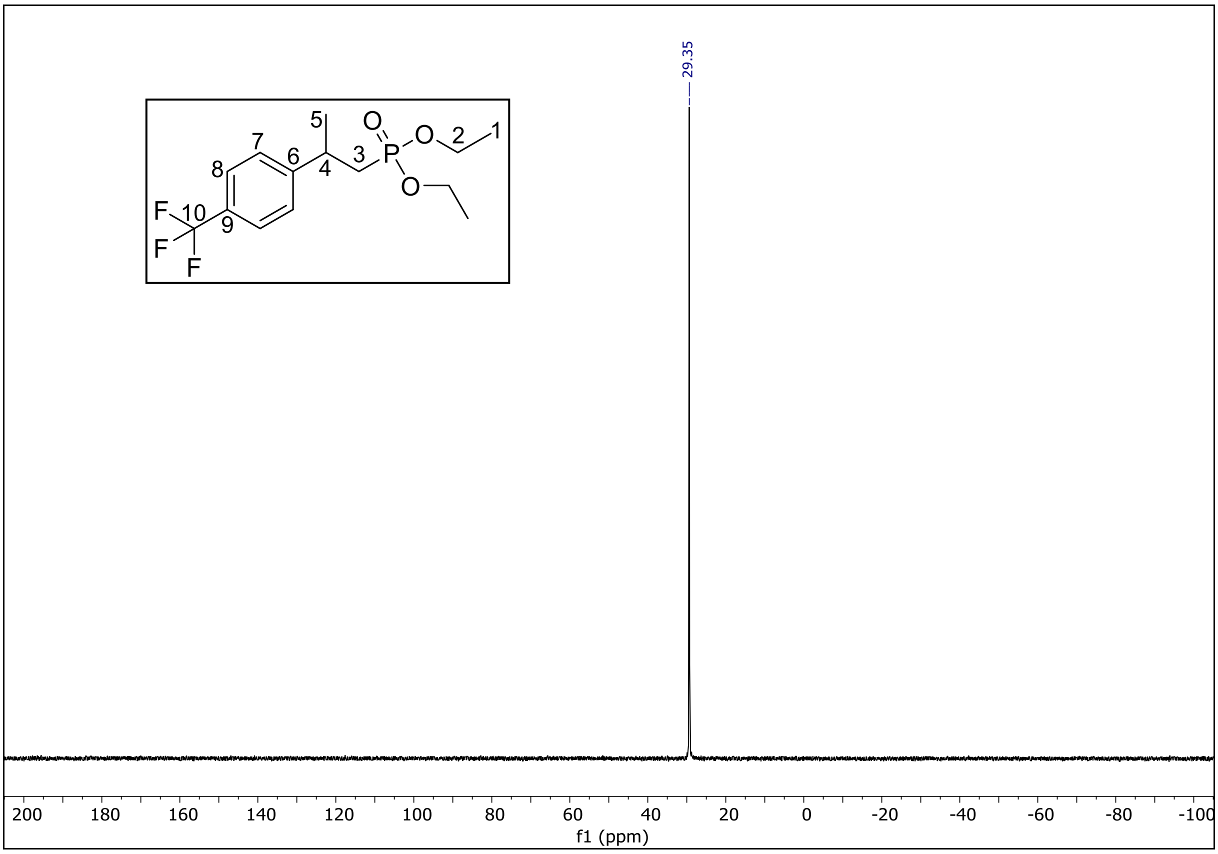

^{1}H NMR (500 MHz, $CDCl_3$): **24**

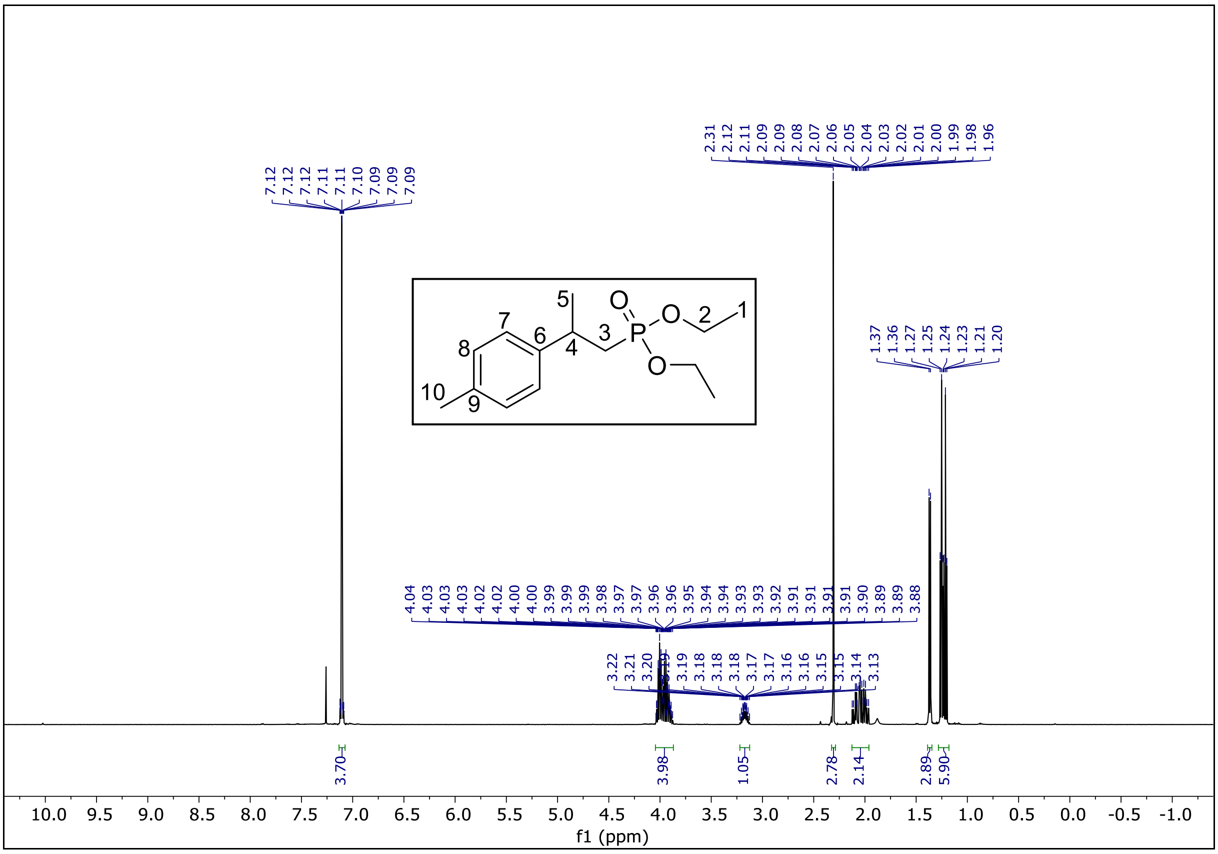

^{13}C NMR (126 MHz, $CDCl_3$): **24**

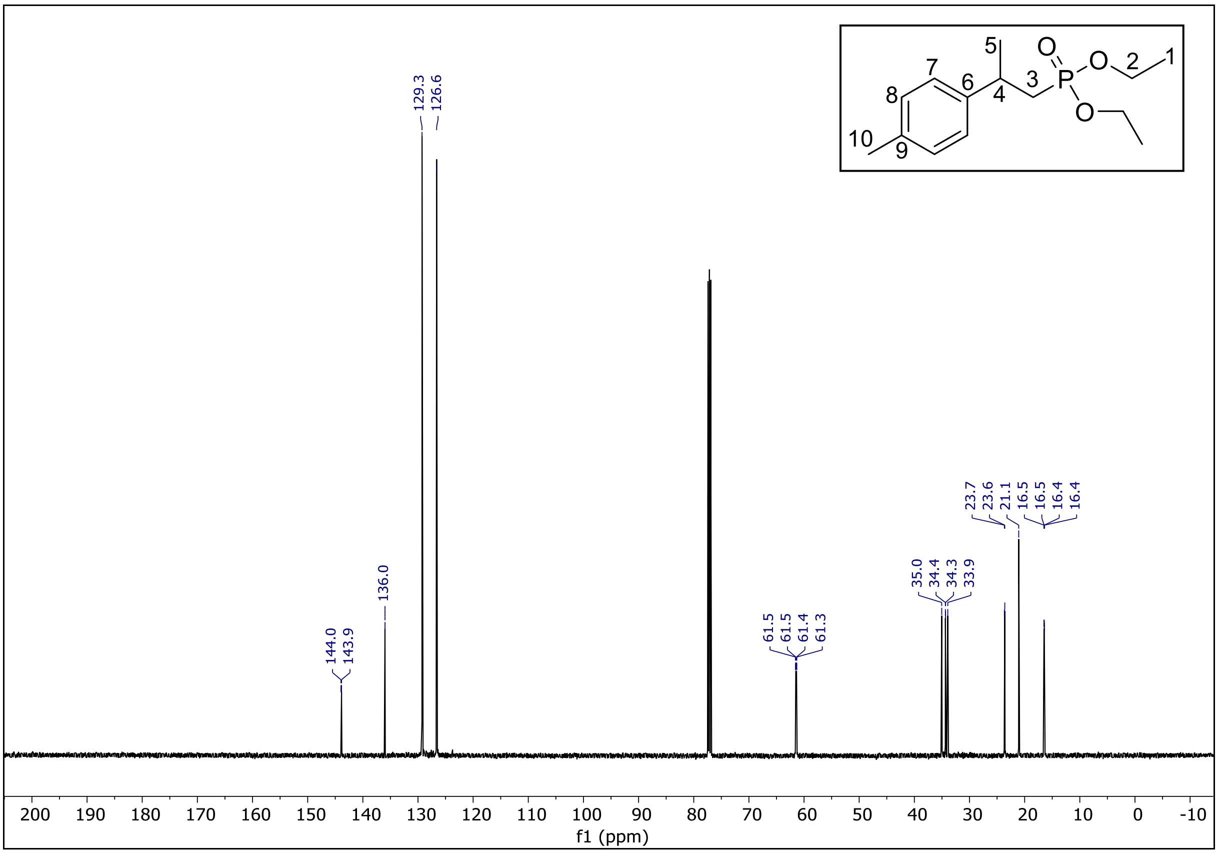

^{31}P NMR (202 MHz, $CDCl_3$): **24**

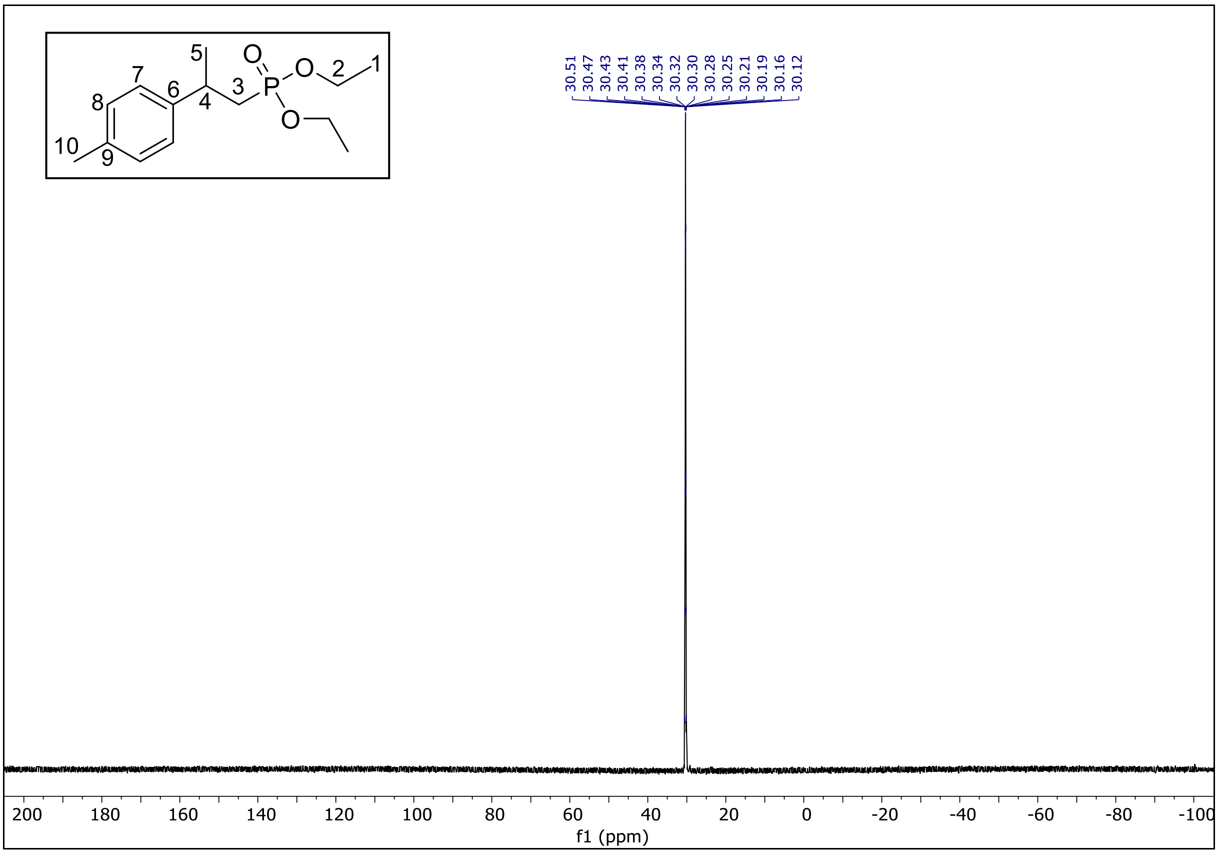

^{1}H NMR (500 MHz, $CDCl_3$): **25**

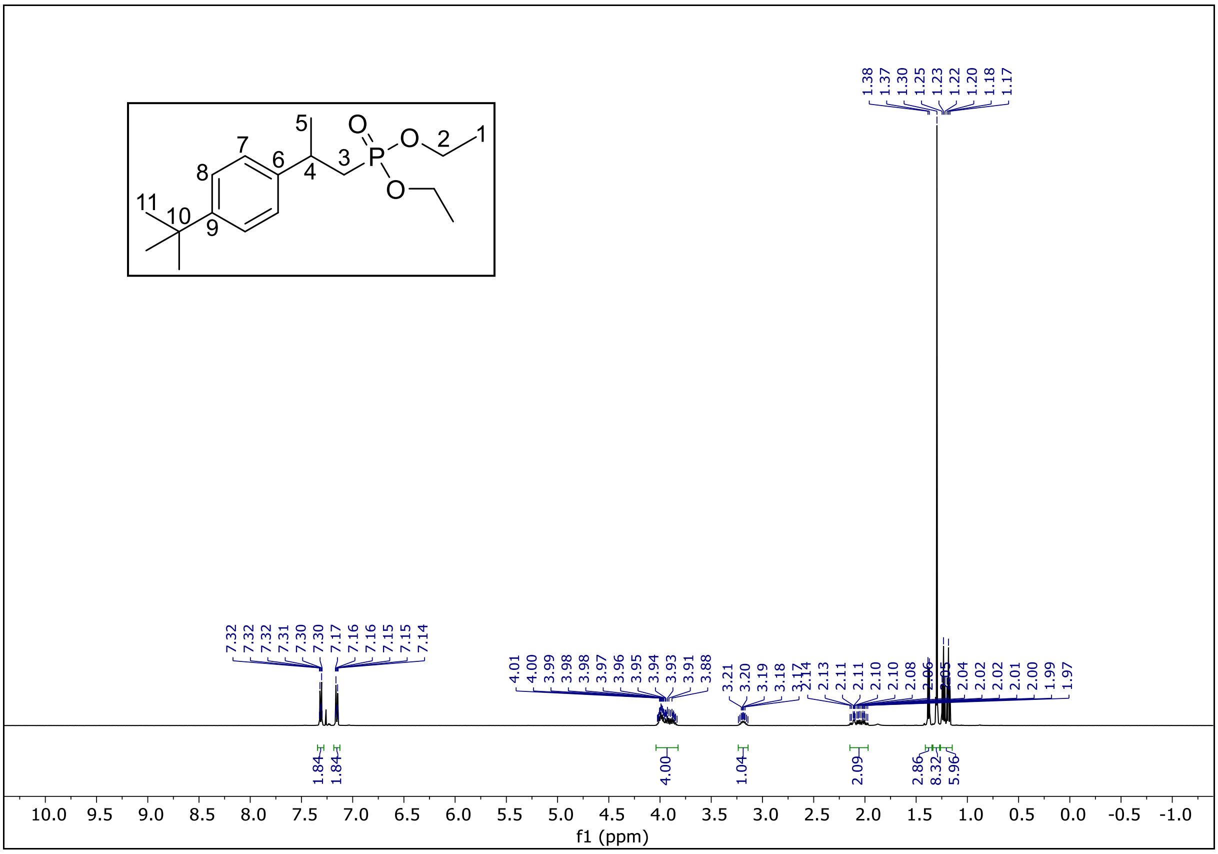

^{13}C NMR (126 MHz, $CDCl_3$): **25**

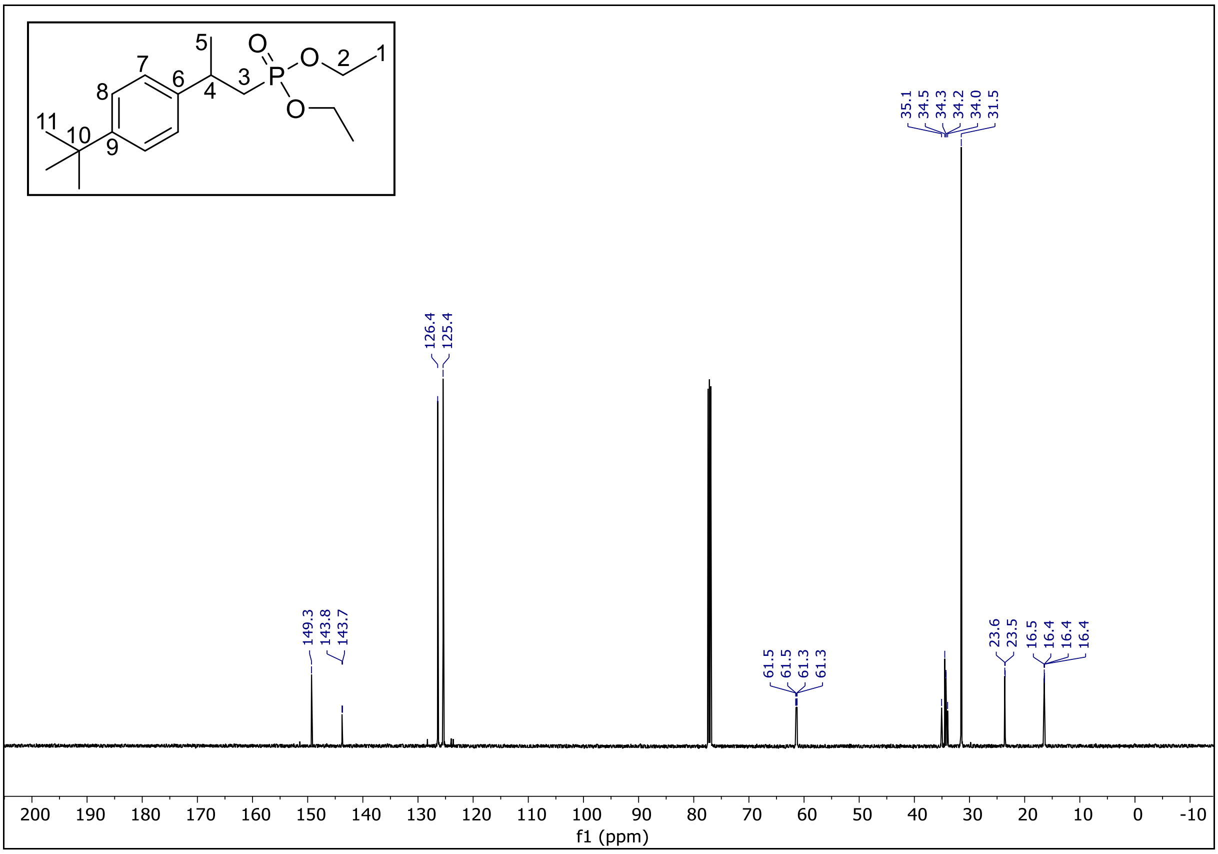

^{31}P NMR (121 MHz, CDCl$_3$): **25**

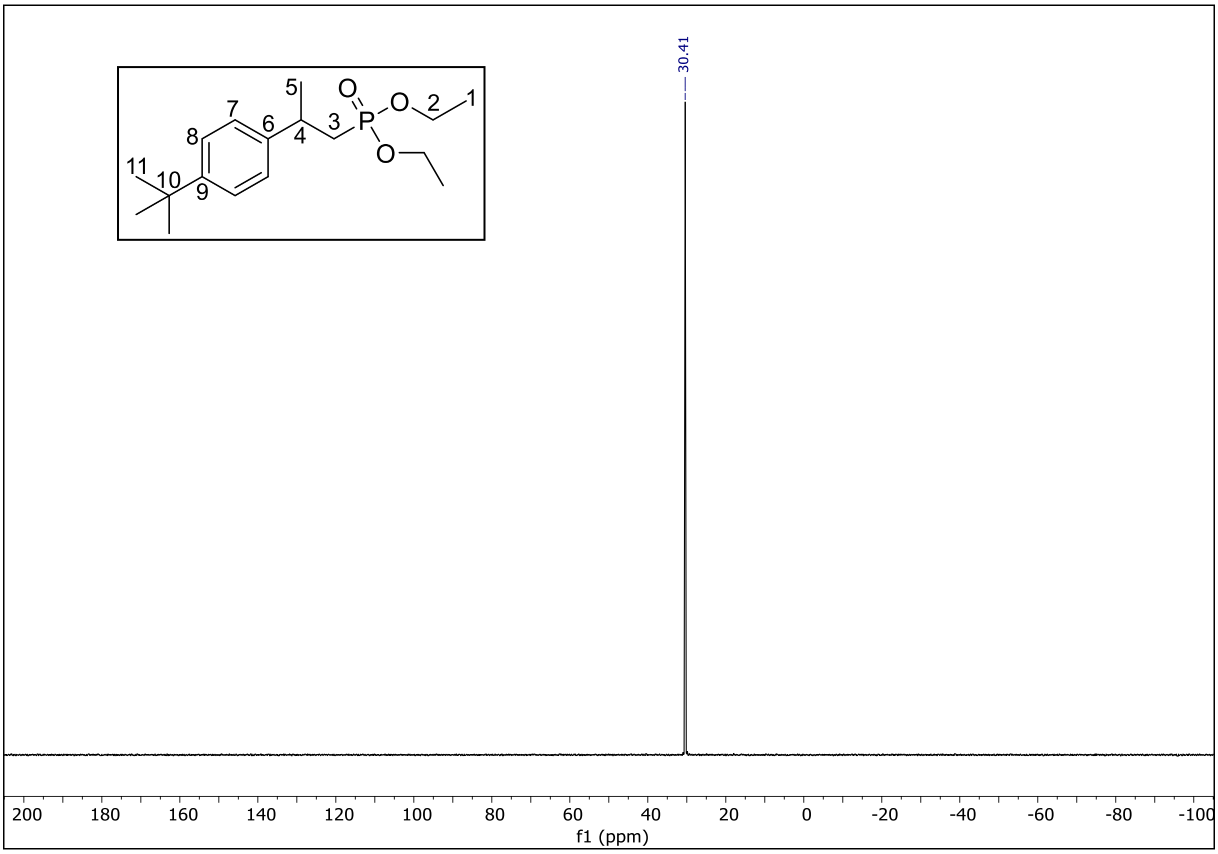

^{1}H NMR (500 MHz, CDCl$_3$): **26**

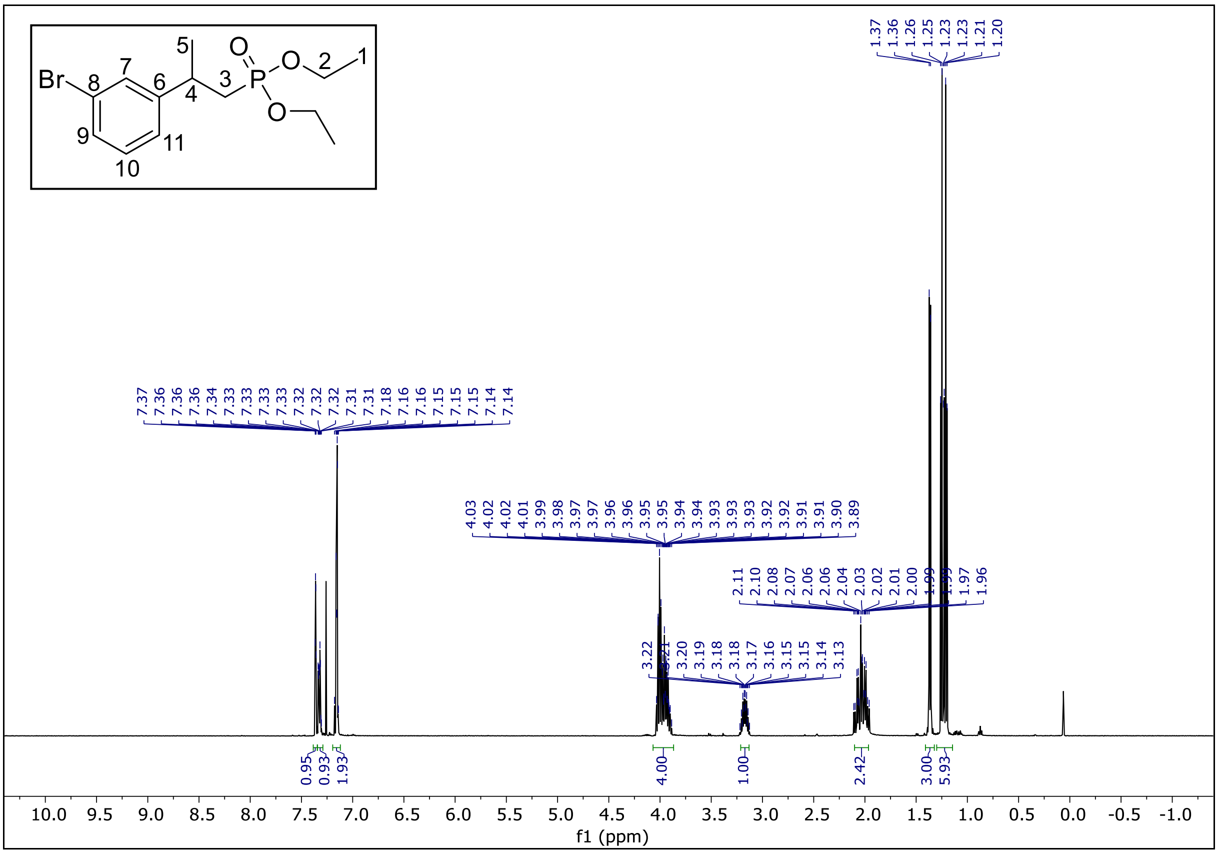

^{13}C NMR (126 MHz, $CDCl_3$): **26**

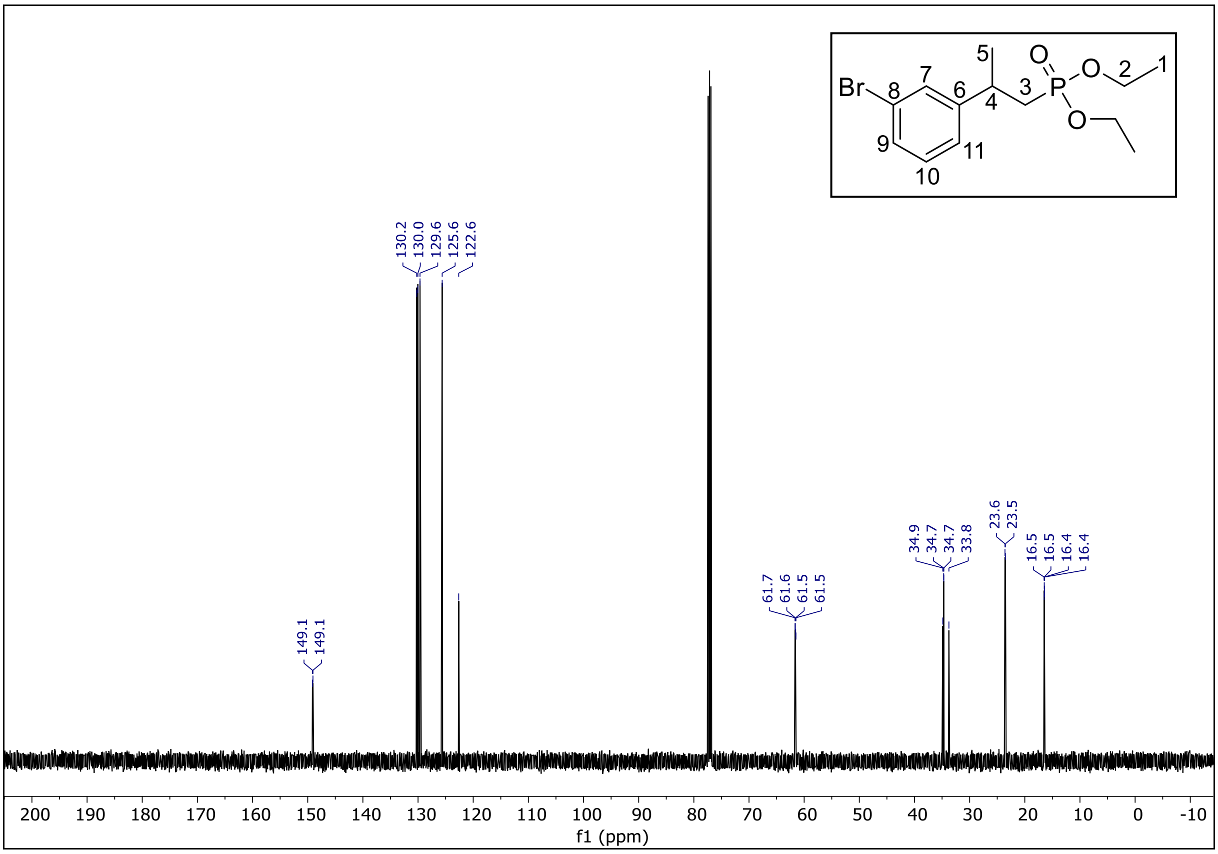

^{31}P NMR (202 MHz, $CDCl_3$): **26**

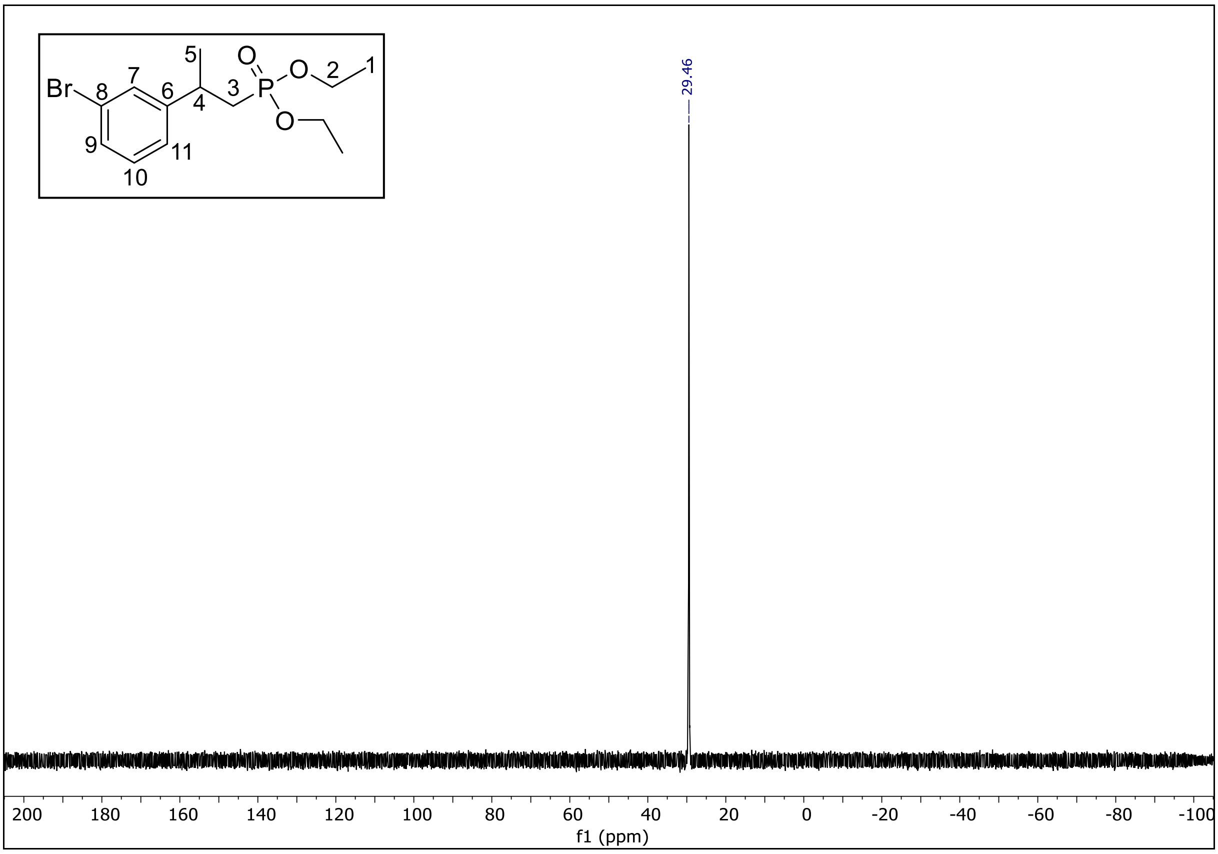

^{1}H NMR (500 MHz, $CDCl_3$): **27**

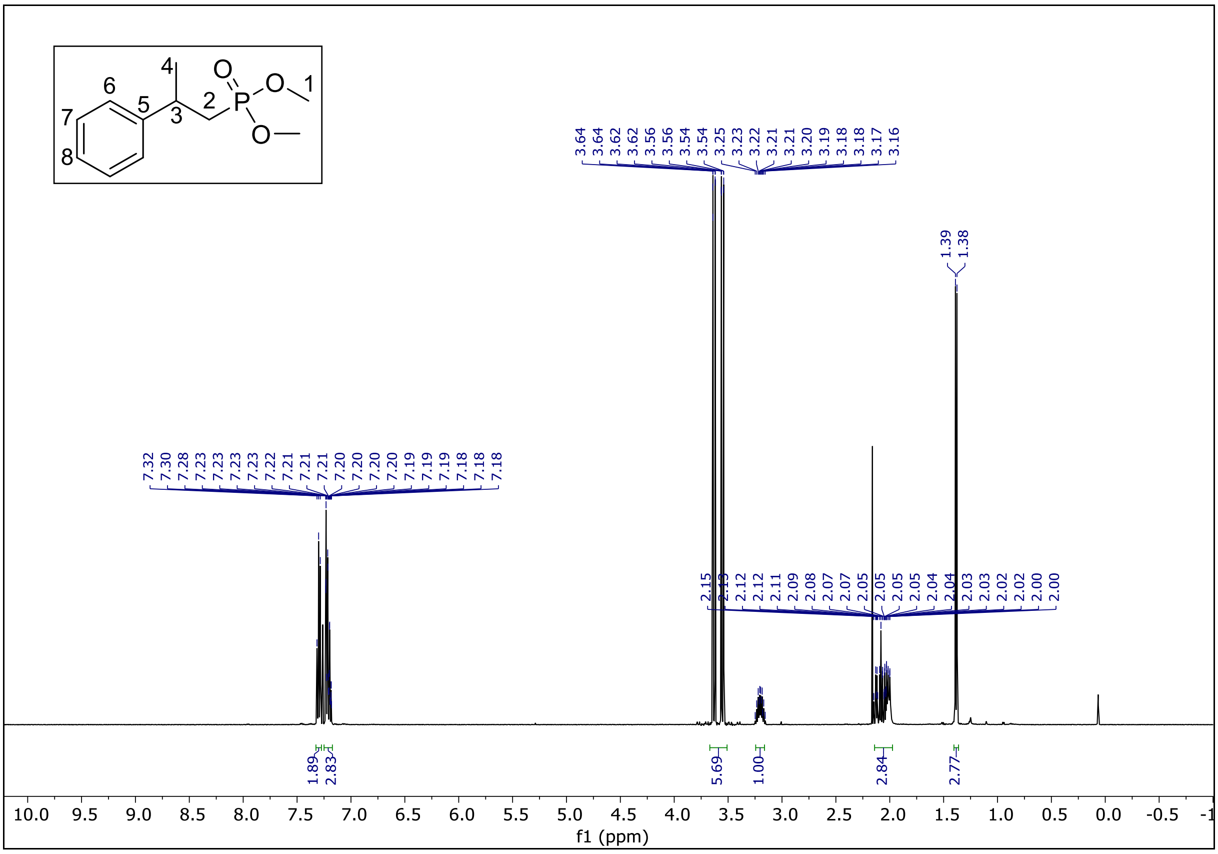

^{13}C NMR (126 MHz, $CDCl_3$): **27**

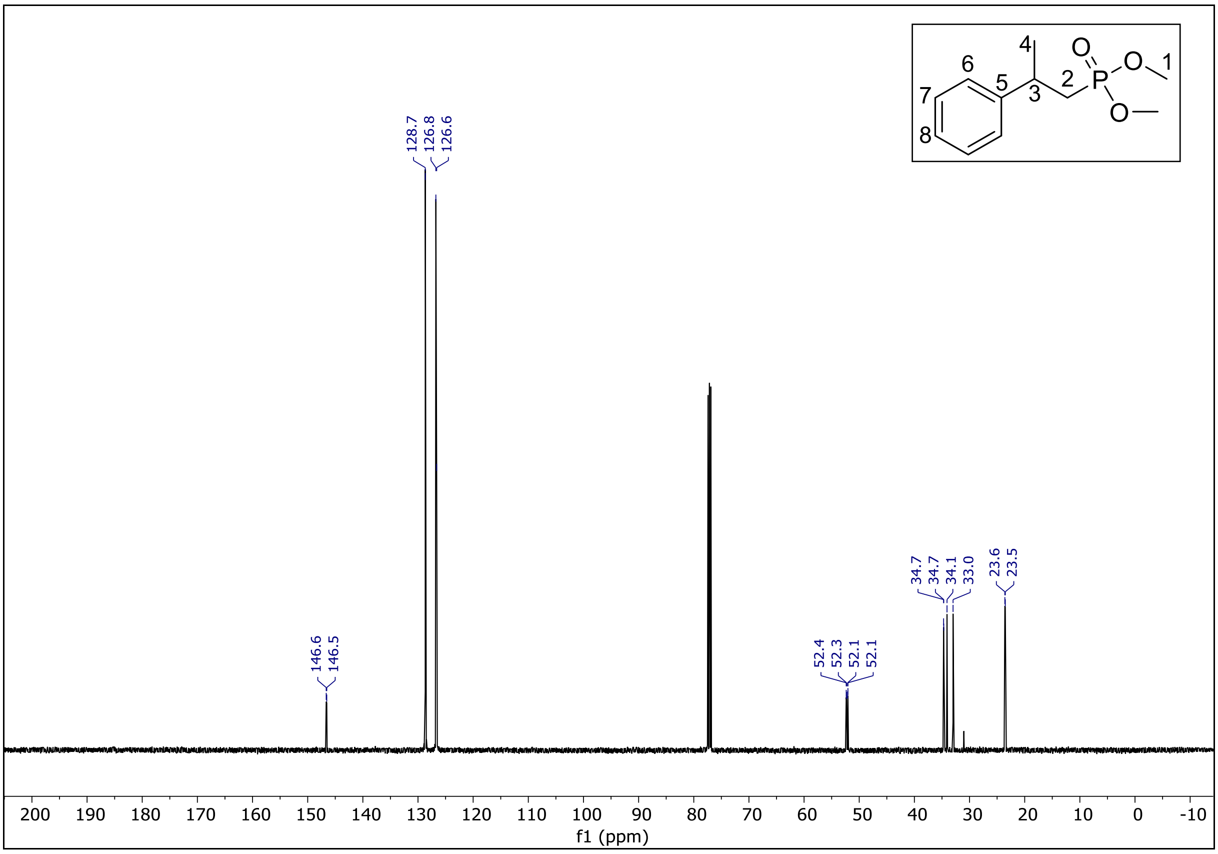

^{31}P NMR (162 MHz, $CDCl_3$): **27**

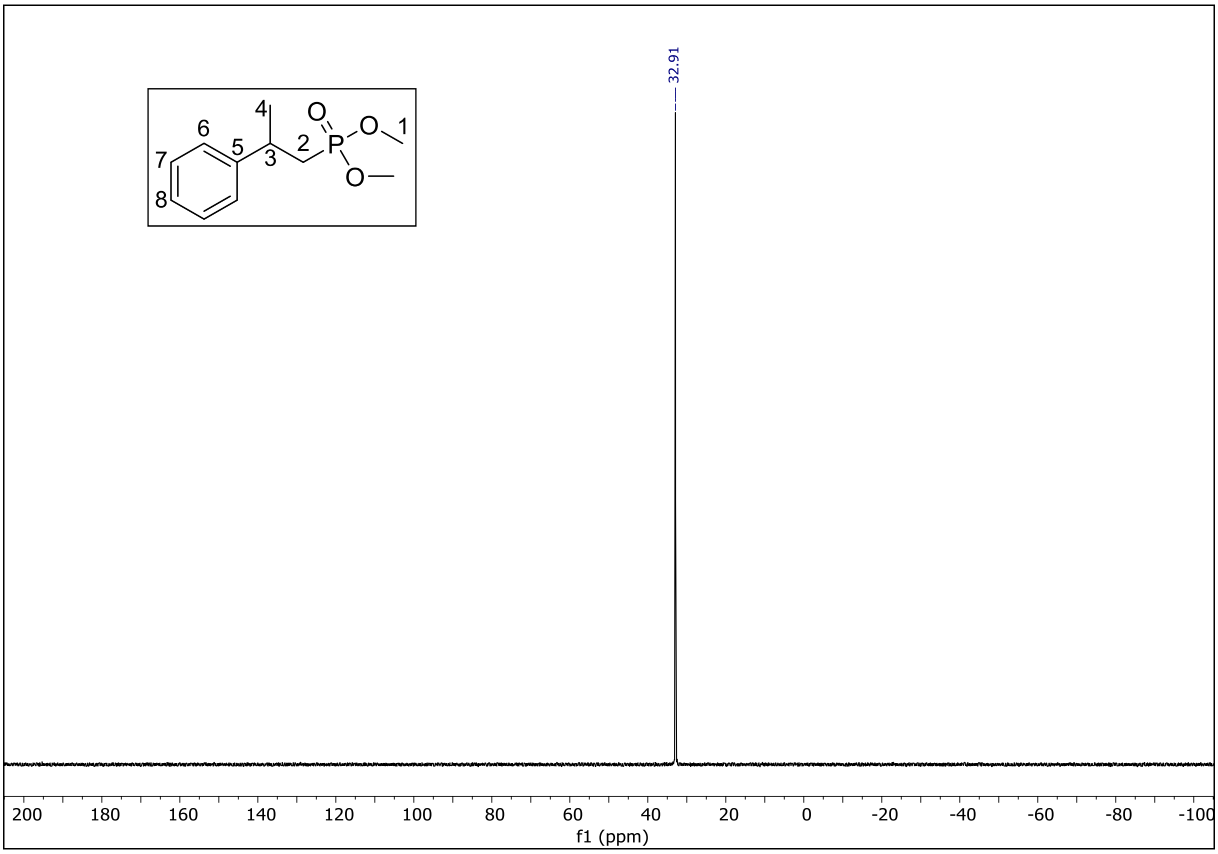

^{1}H NMR (400 MHz, $CDCl_3$): **28**

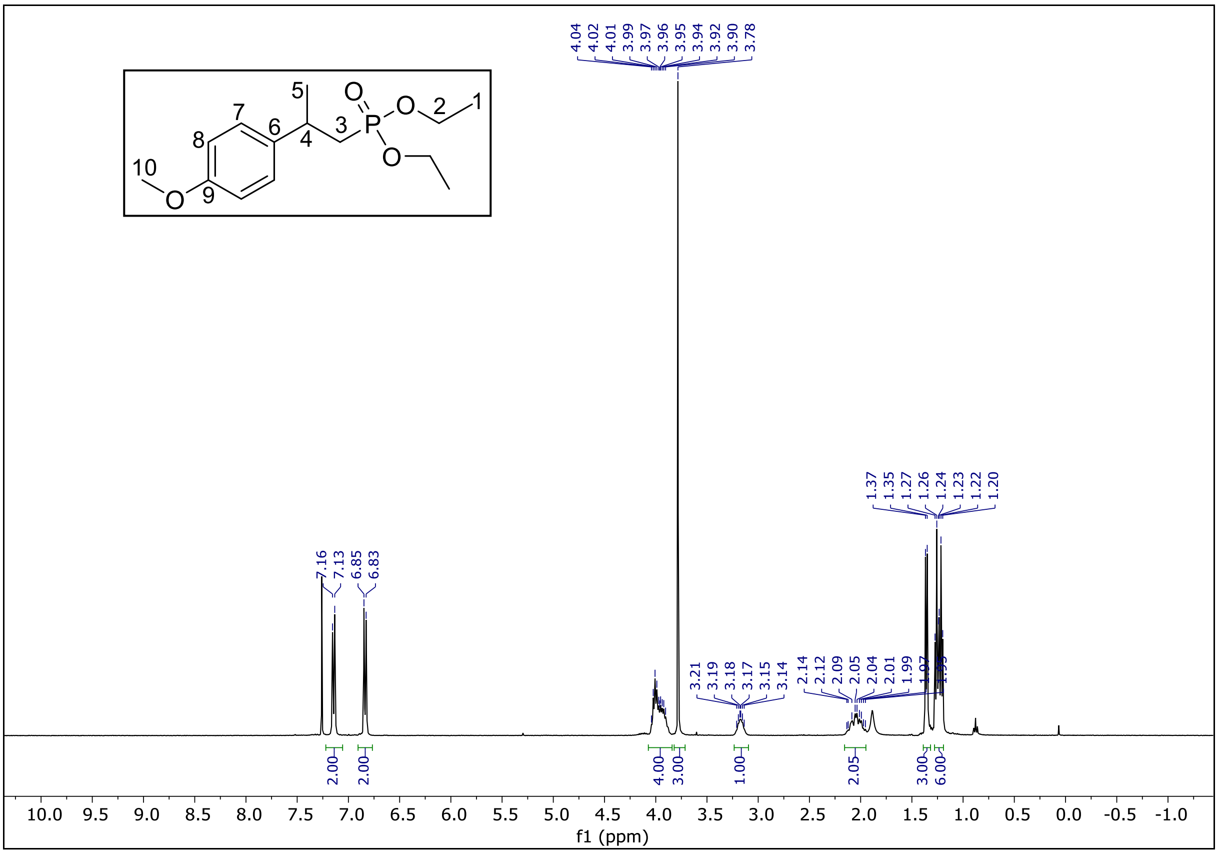

^{13}C NMR (101 MHz, $CDCl_3$): **28**

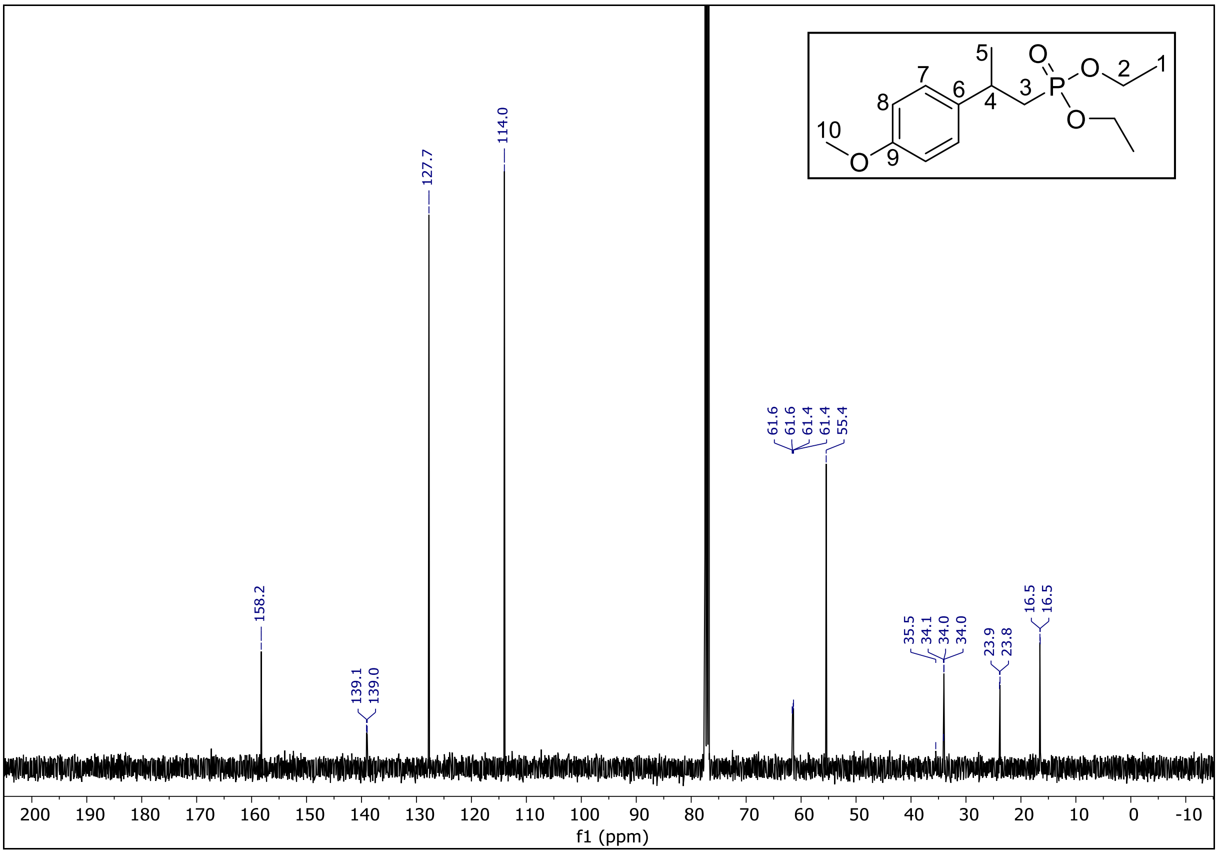

^{31}P NMR (162 MHz, $CDCl_3$): **28**

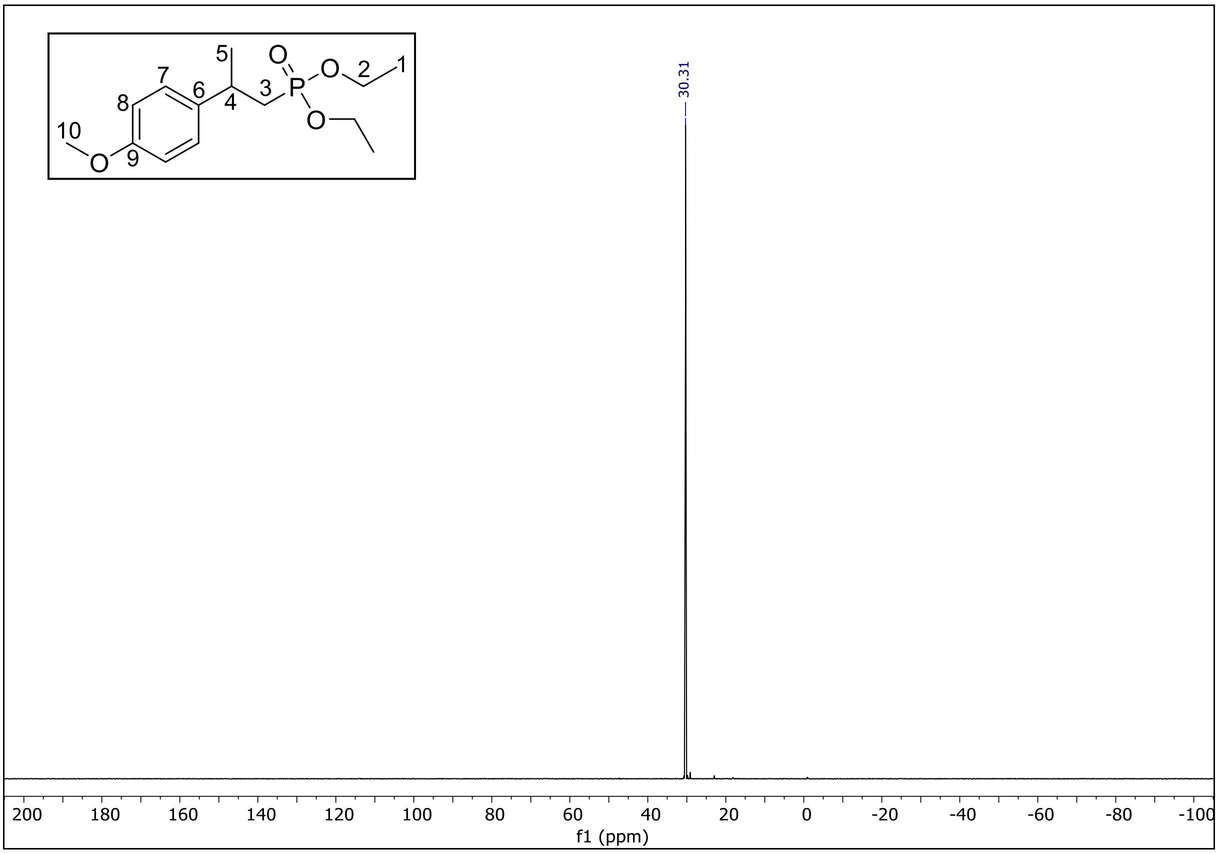

^{1}H NMR (500 MHz, $CDCl_3$): **29**

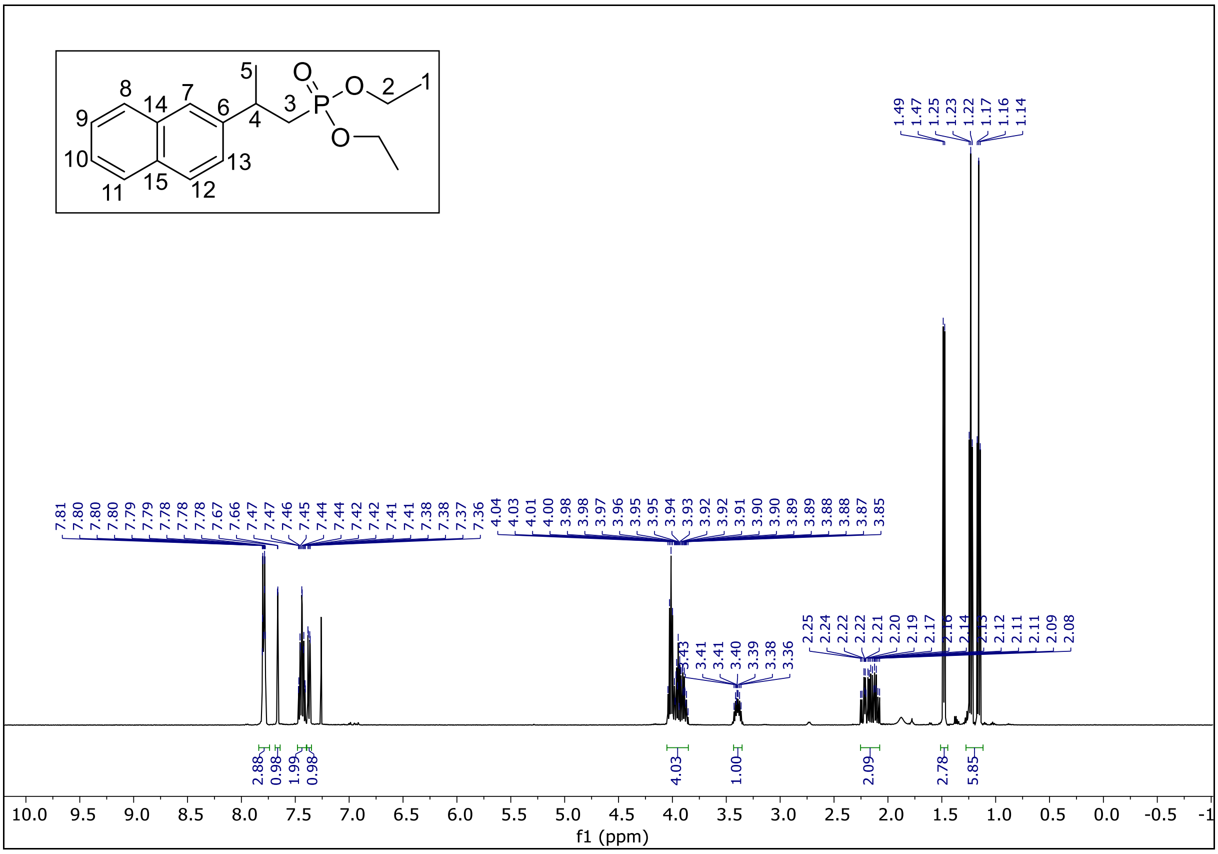

^{13}C NMR (126 MHz, $CDCl_3$): **29**

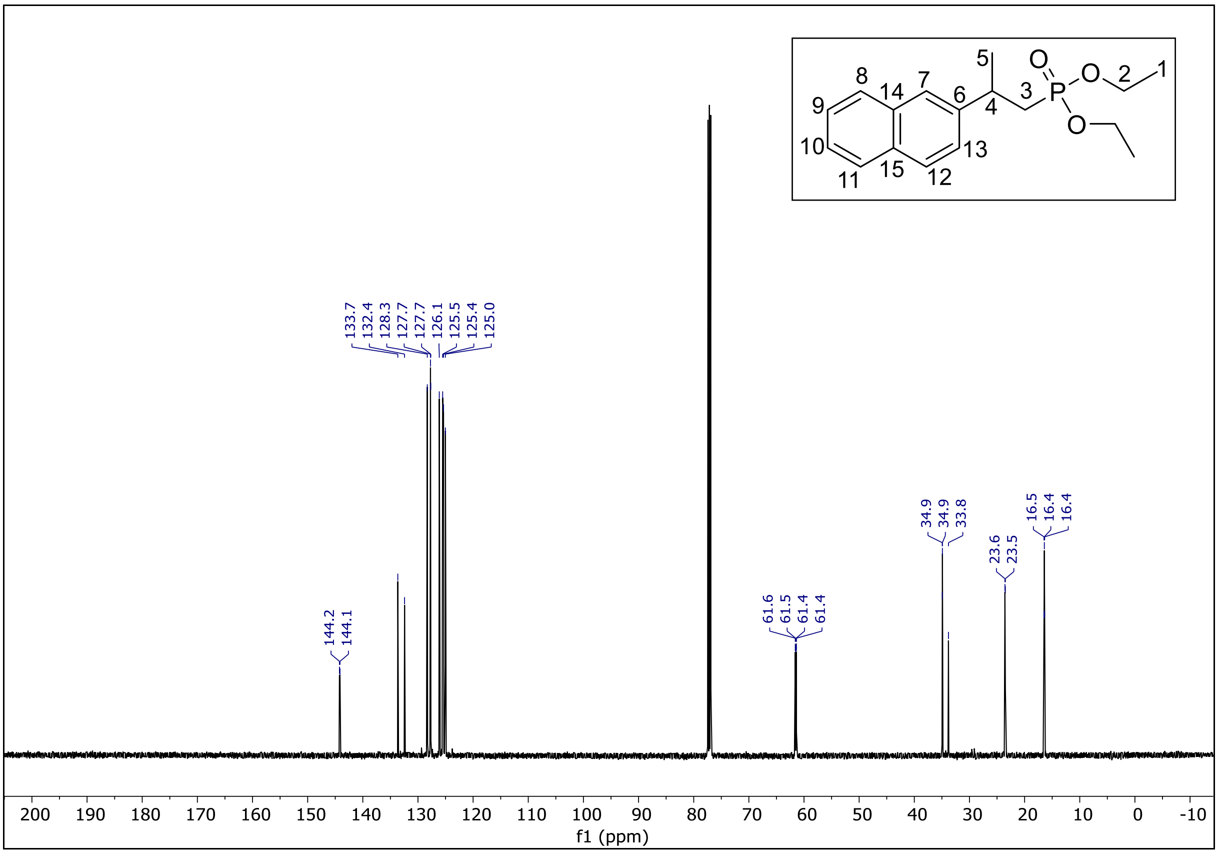

^{31}P NMR (121 MHz, $CDCl_3$): **29**

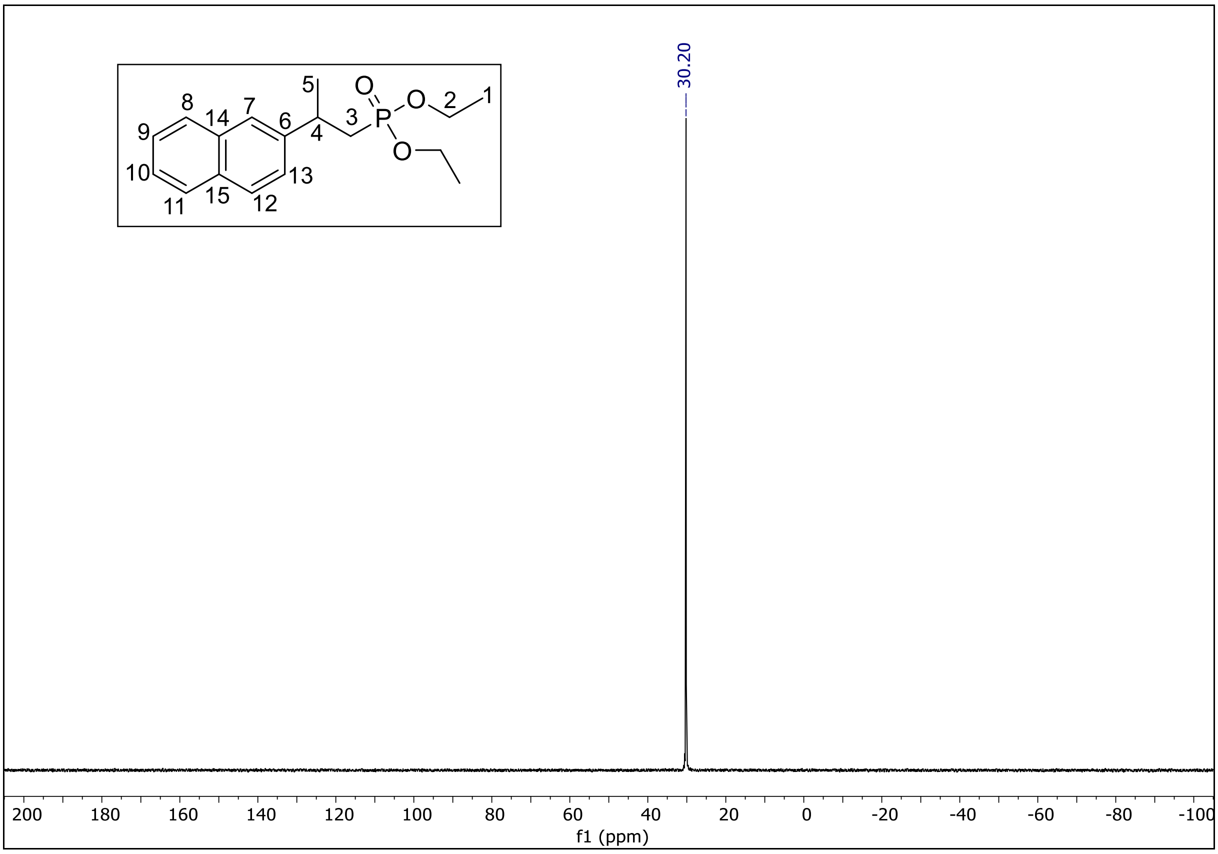

^{1}H NMR (400 MHz, $CDCl_3$): **30**

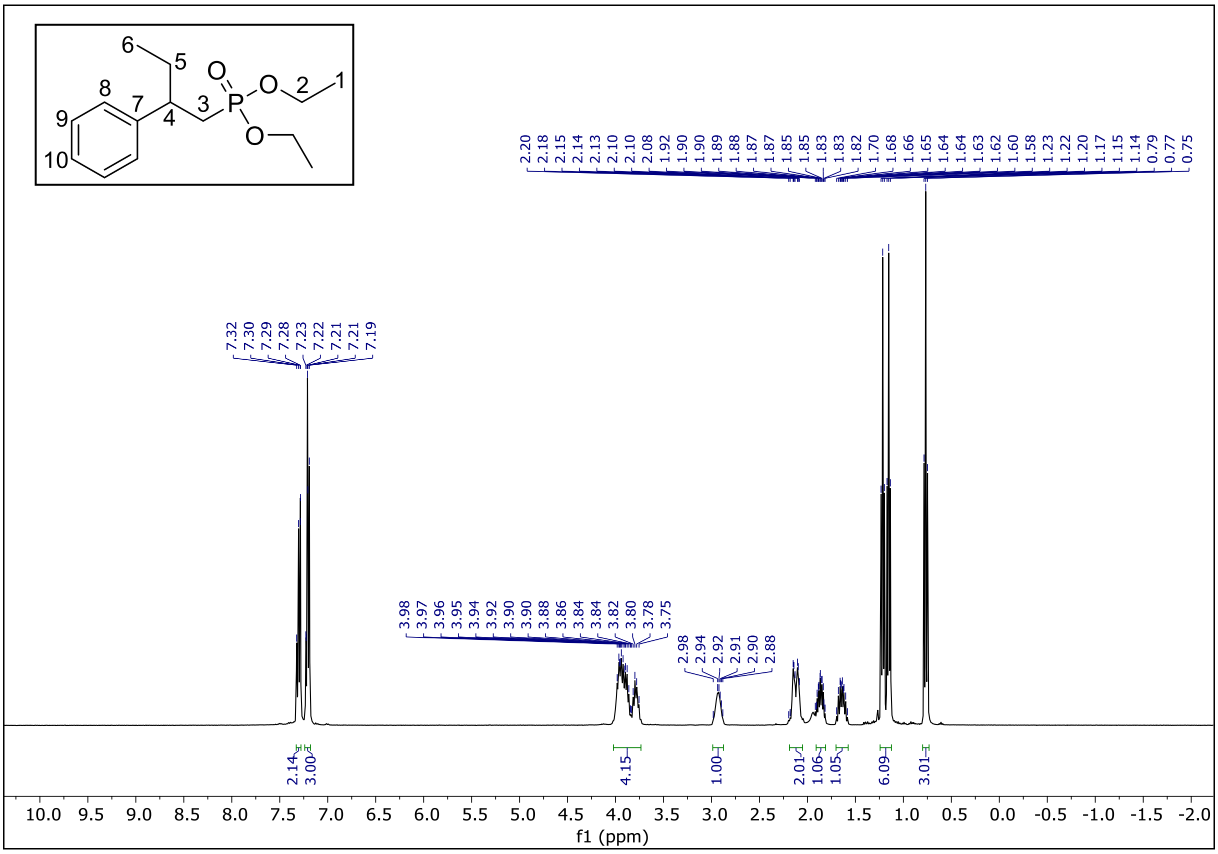

^{13}C NMR (101 MHz, $CDCl_3$): **30**

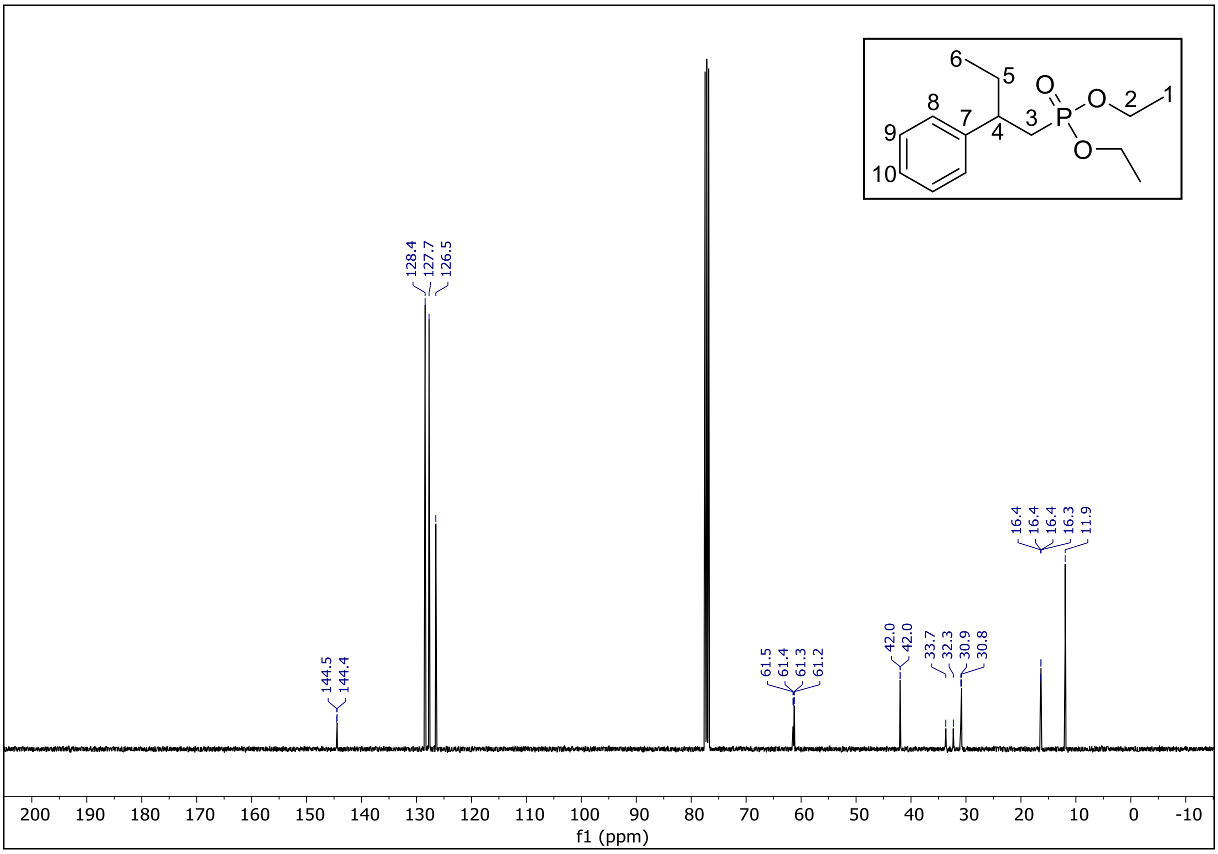

^{31}P NMR (121 MHz, $CDCl_3$): **30**

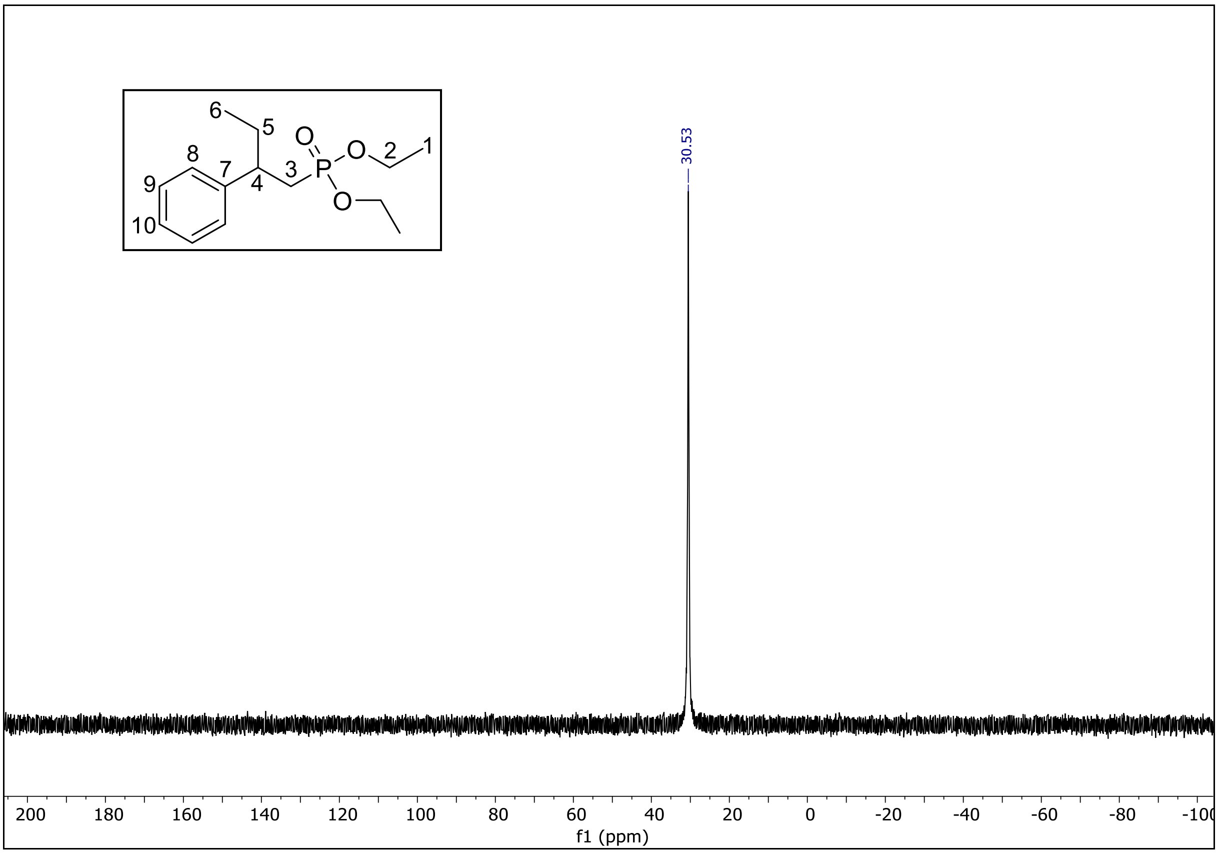

^{1}H NMR (500 MHz, $CDCl_3$): **31**

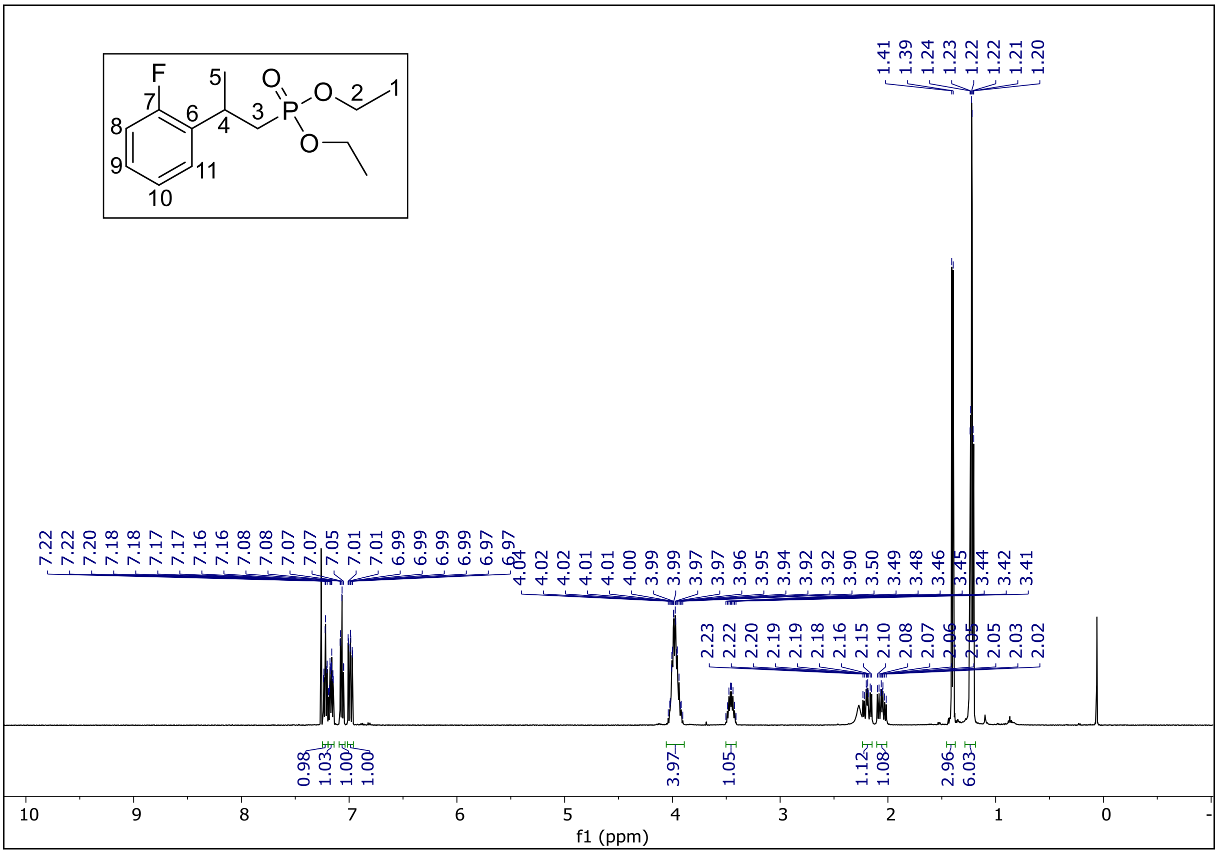

^{13}C NMR (126 MHz, $CDCl_3$): **31**

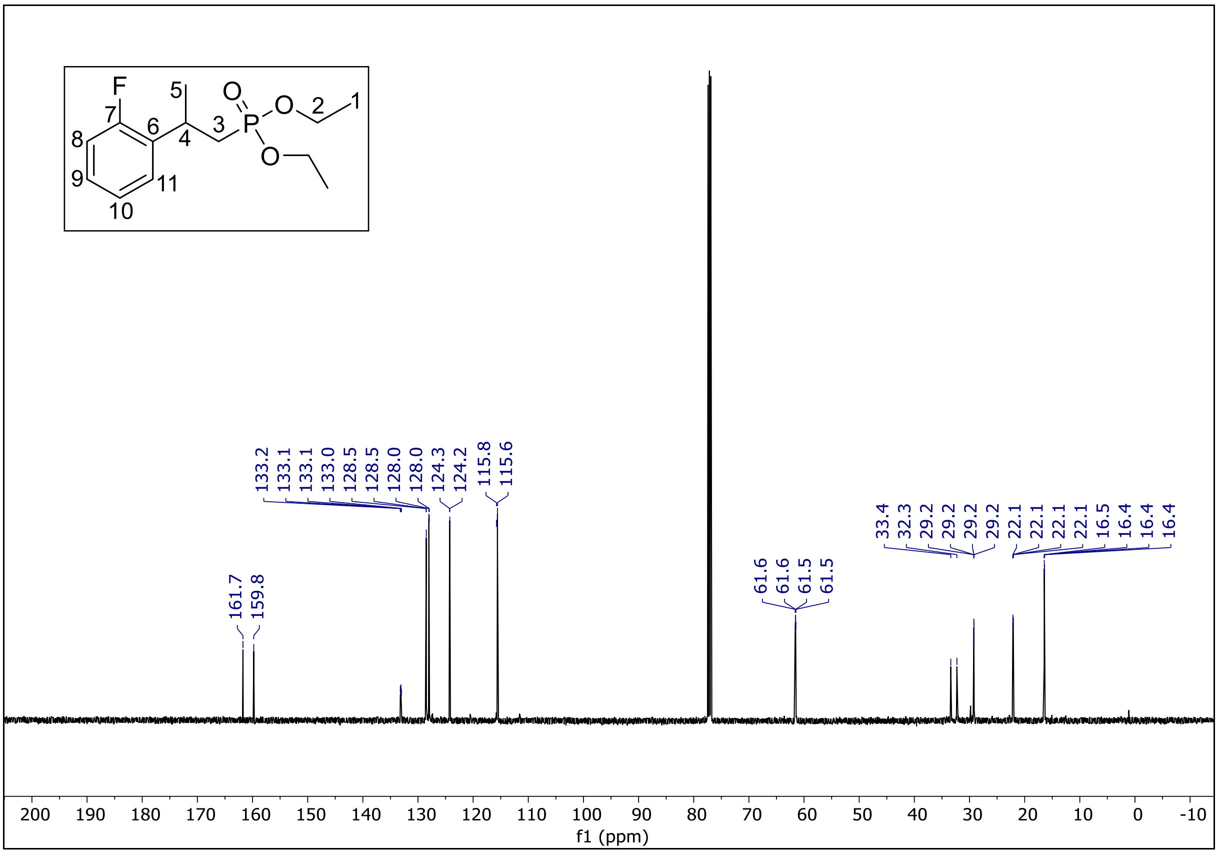

^{19}F NMR (564 MHz, $CDCl_3$): **31**

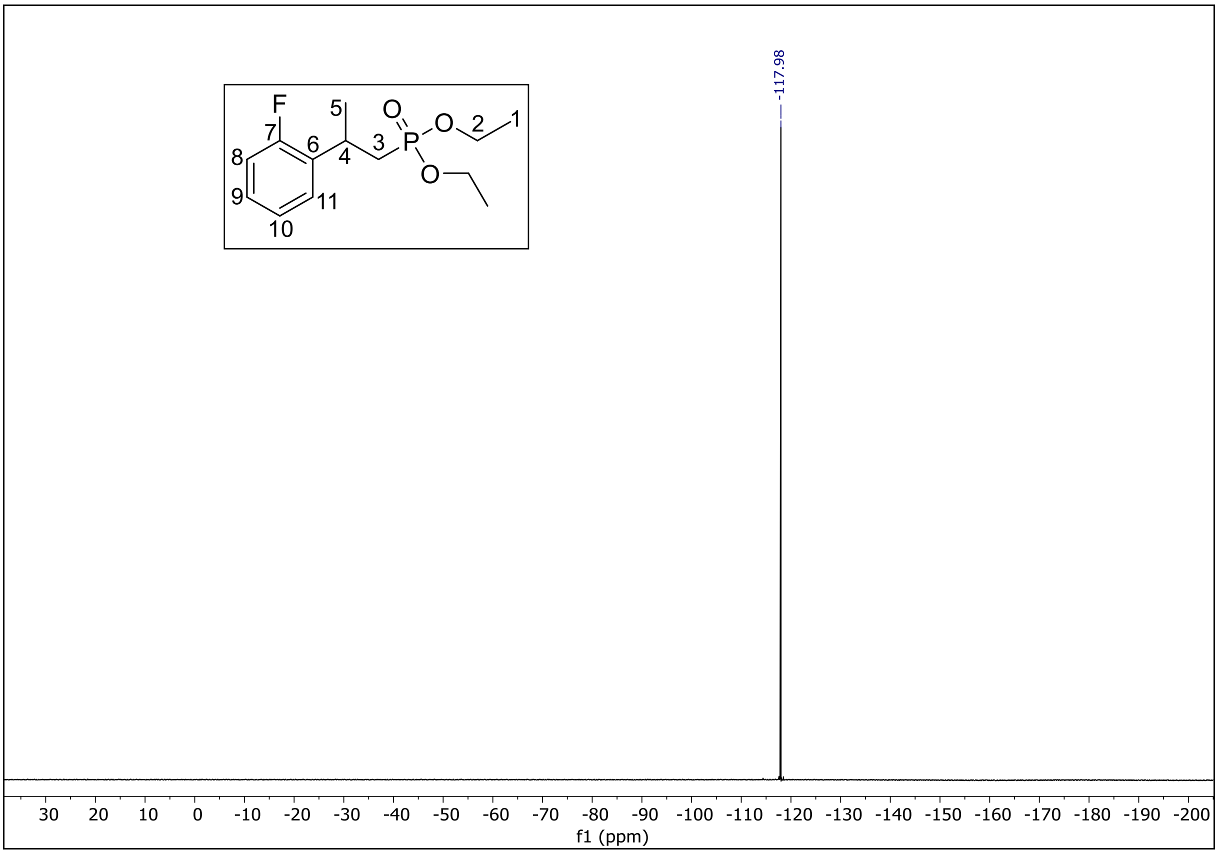

^{31}P NMR (162 MHz, $CDCl_3$): **31**

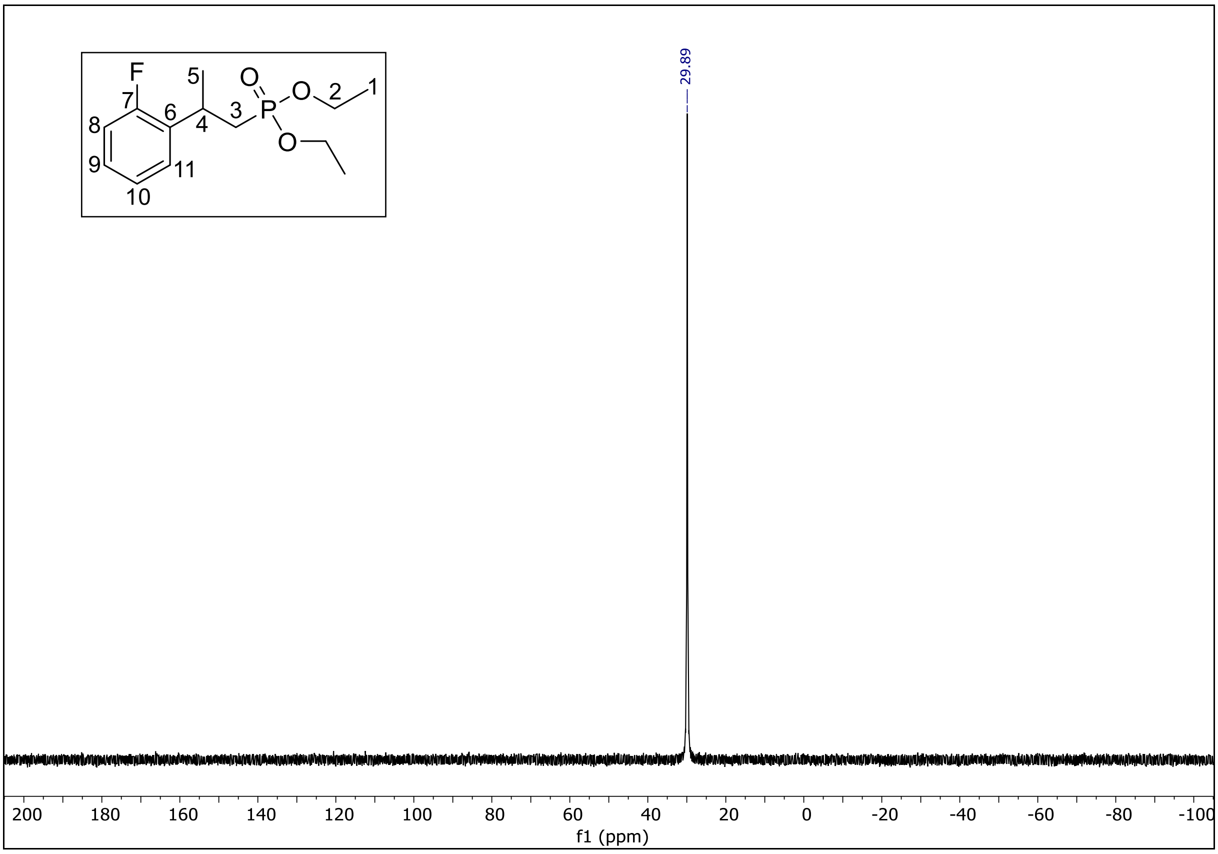

^{1}H NMR (500 MHz, $CDCl_3$): **32**

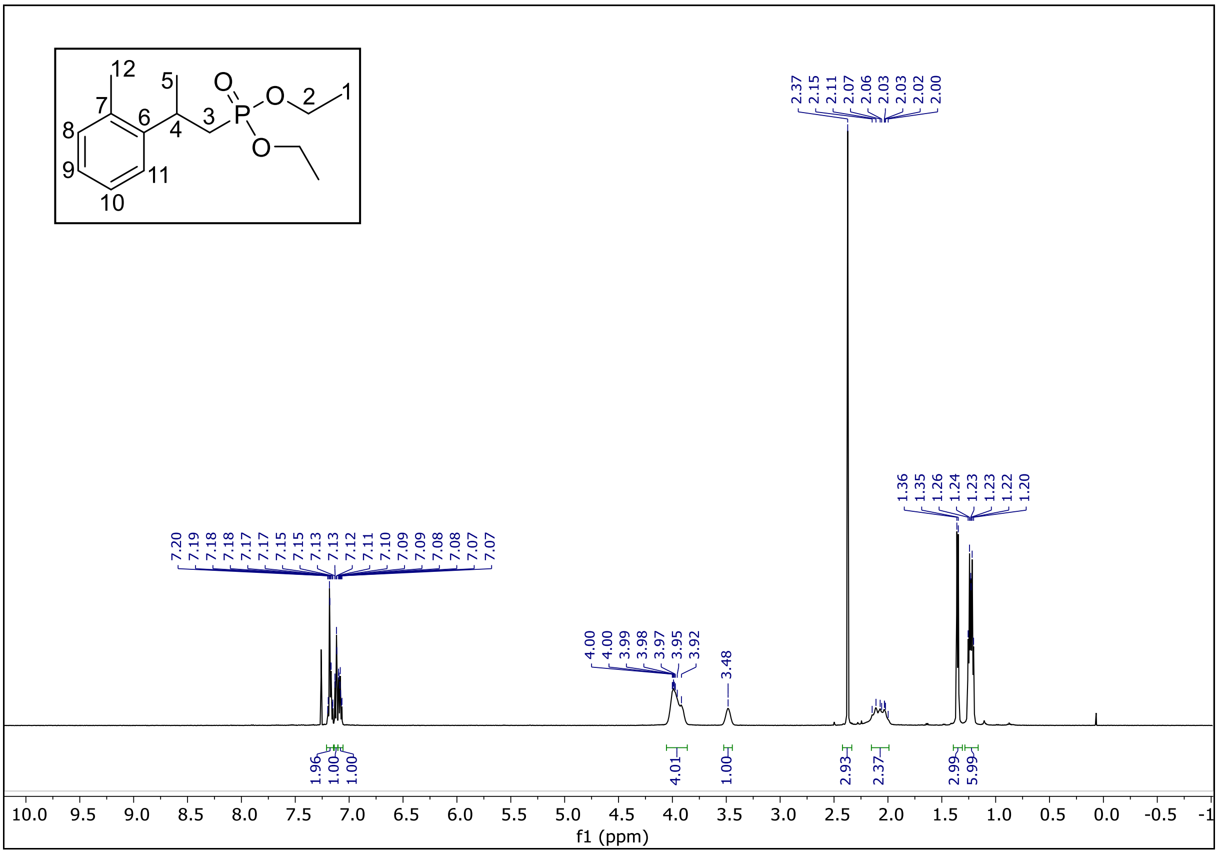

^{13}C NMR (126 MHz, $CDCl_3$): **32**

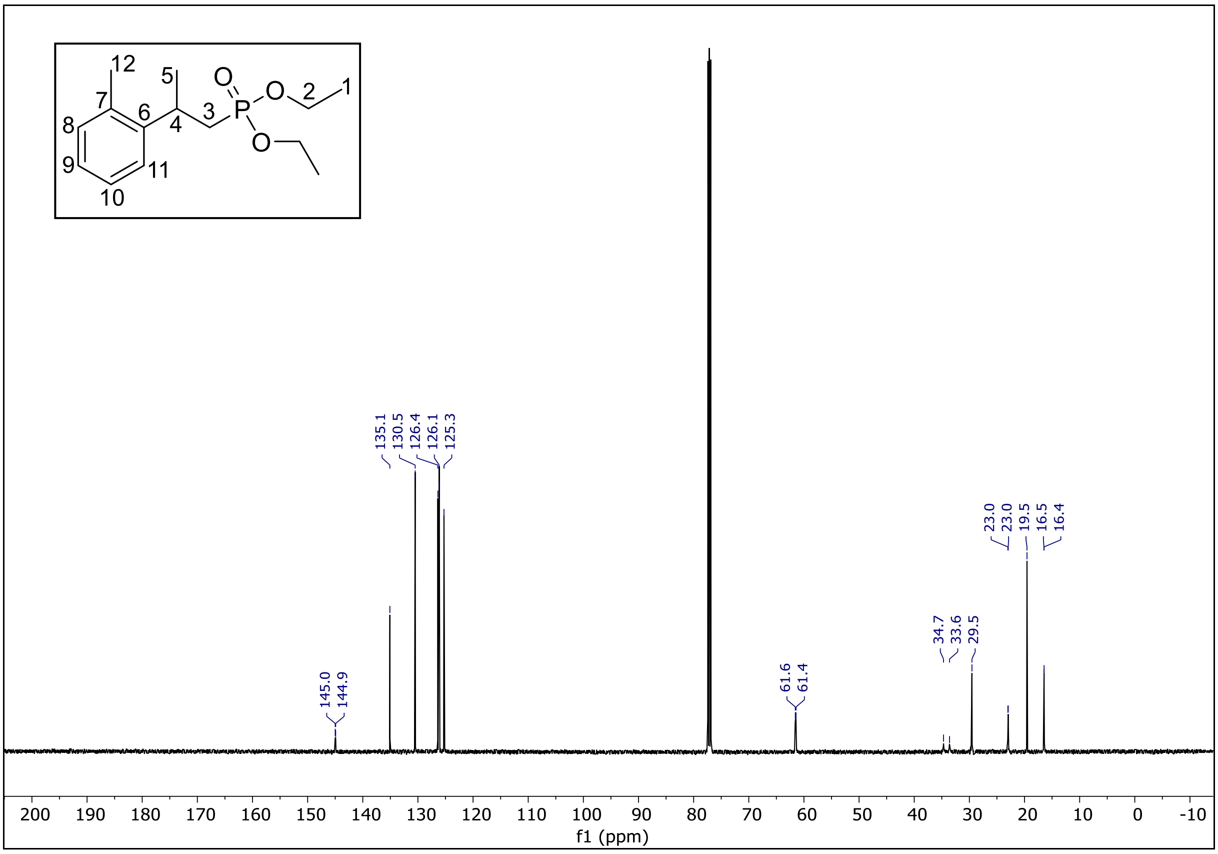

^{31}P NMR (162 MHz, $CDCl_3$): **32**

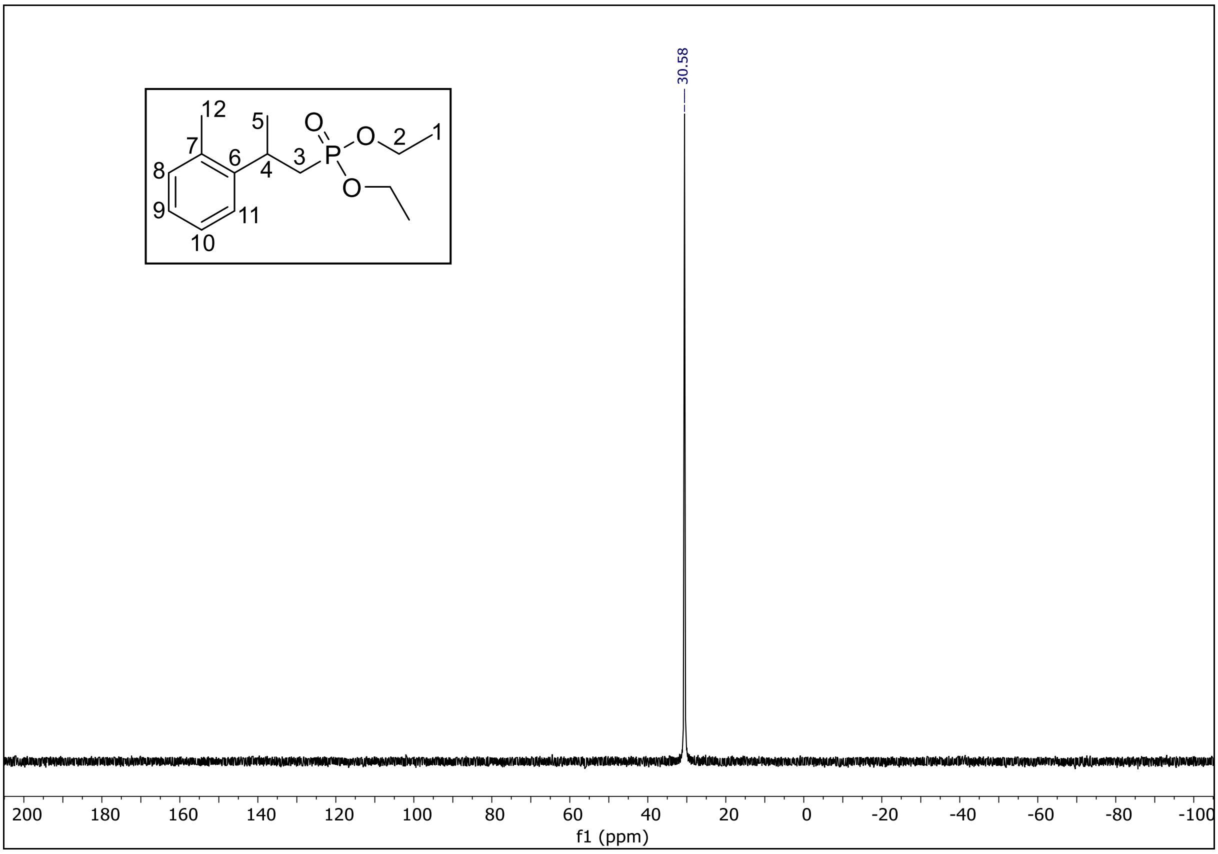

^{1}H NMR (500 MHz, $CDCl_3$): **33**

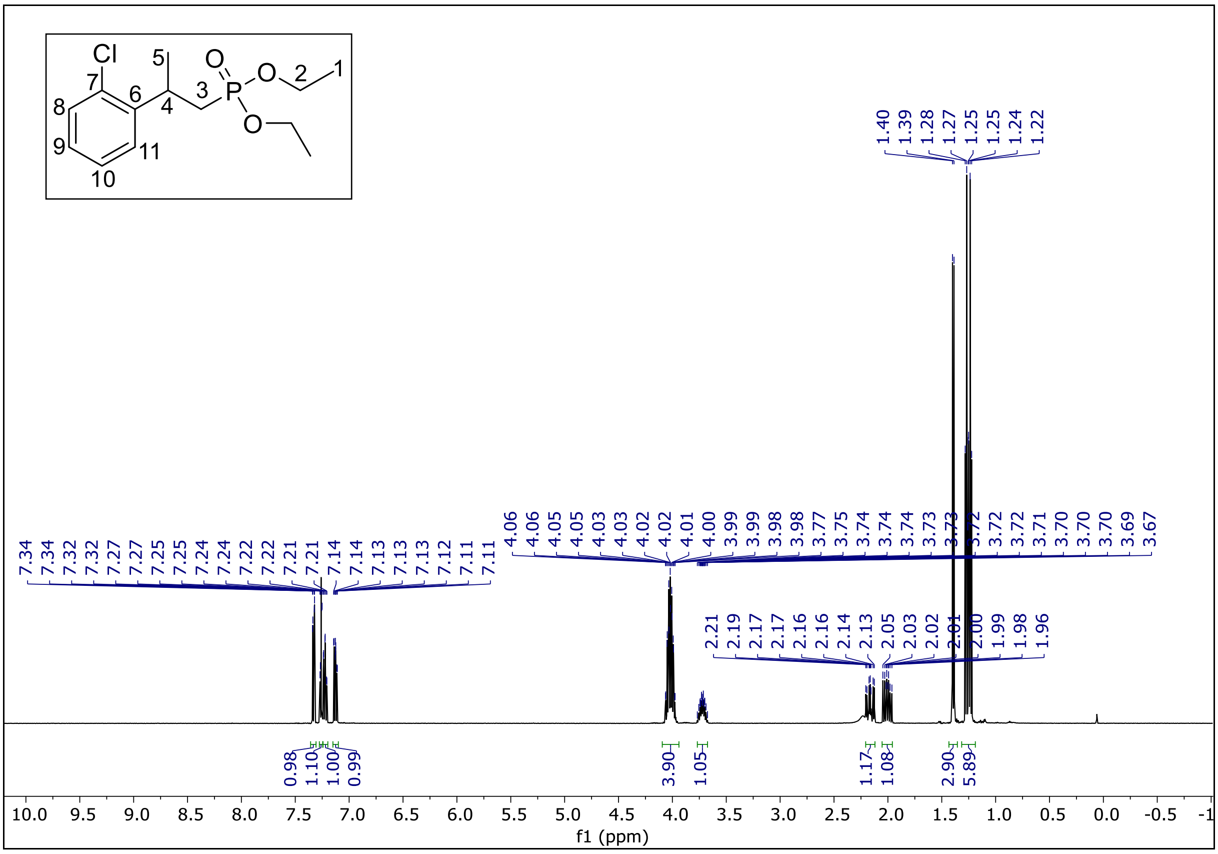

^{13}C NMR (126 MHz, $CDCl_3$): **33**

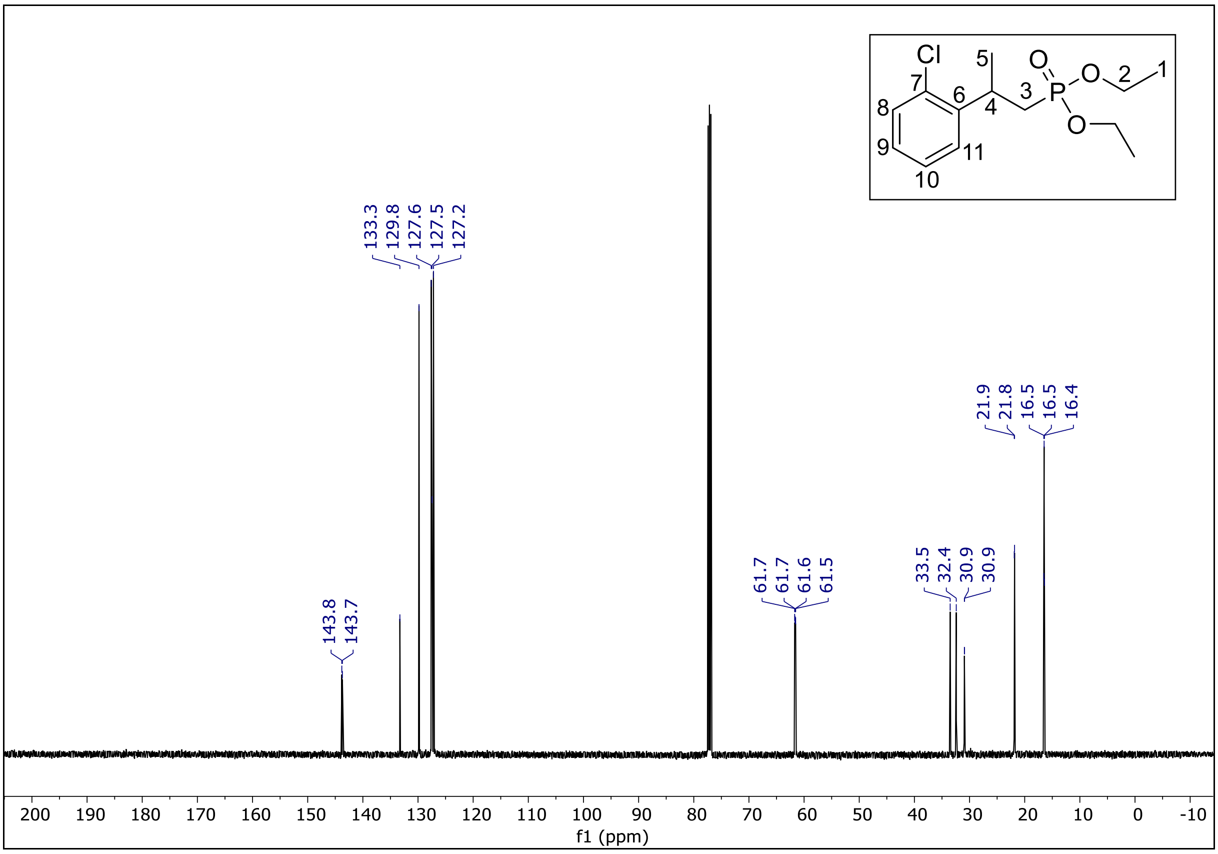

^{31}P NMR (162 MHz, $CDCl_3$): **33**

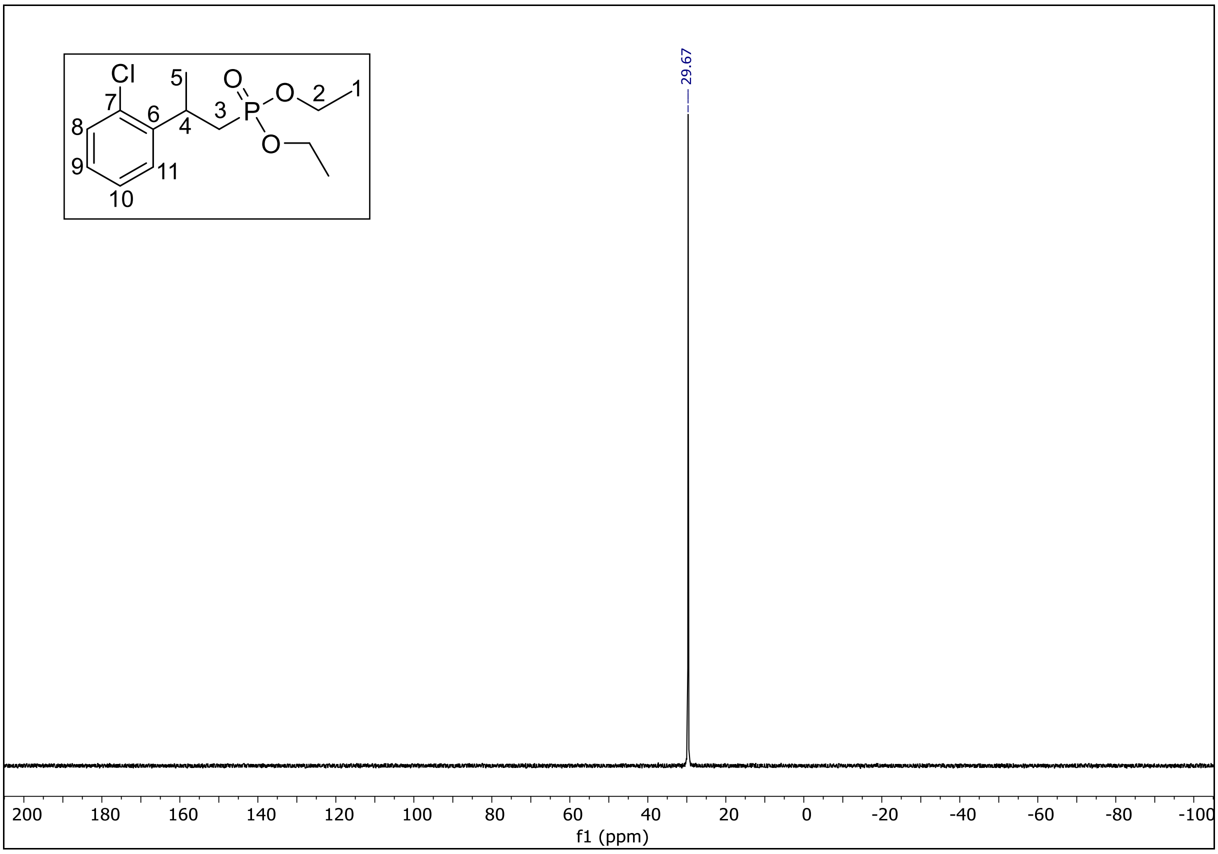

^{1}H NMR (500 MHz, $CDCl_3$): **34**

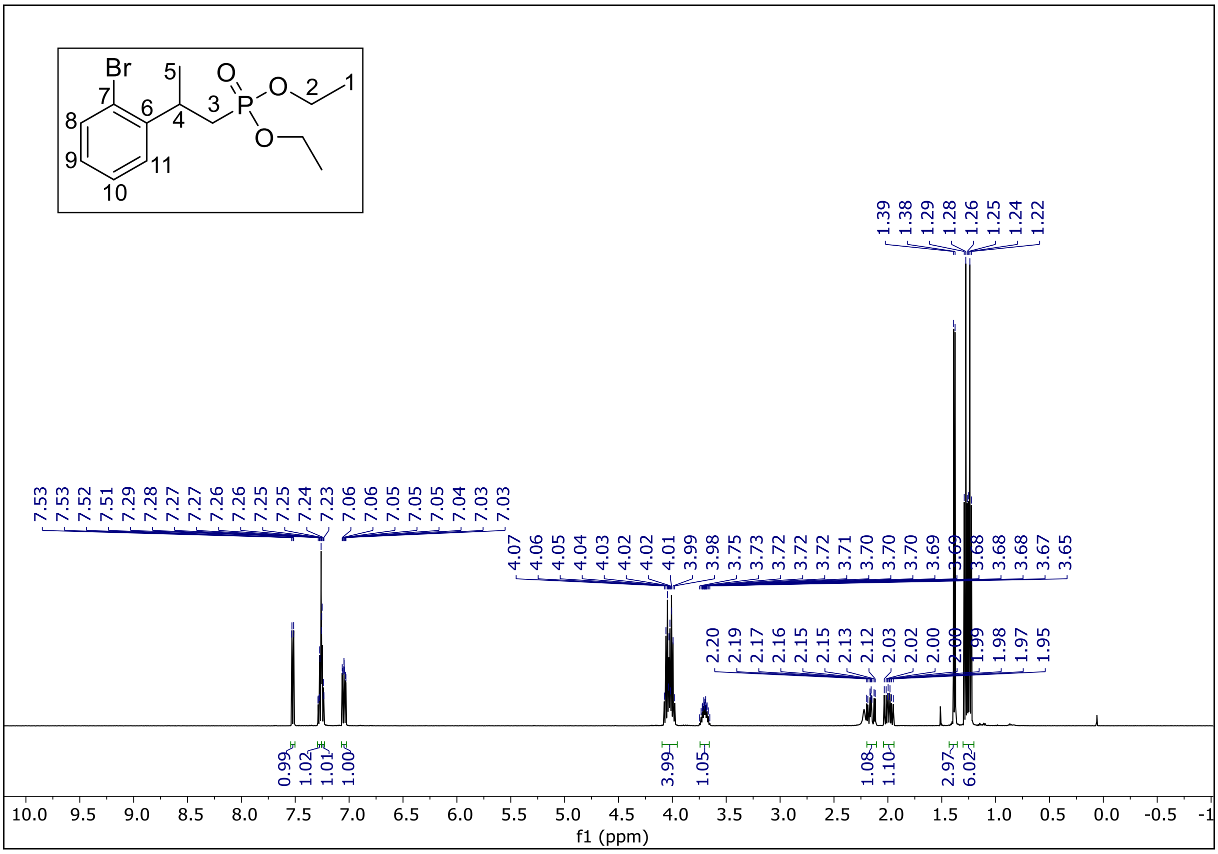

^{13}C NMR (126 MHz, $CDCl_3$): **34**

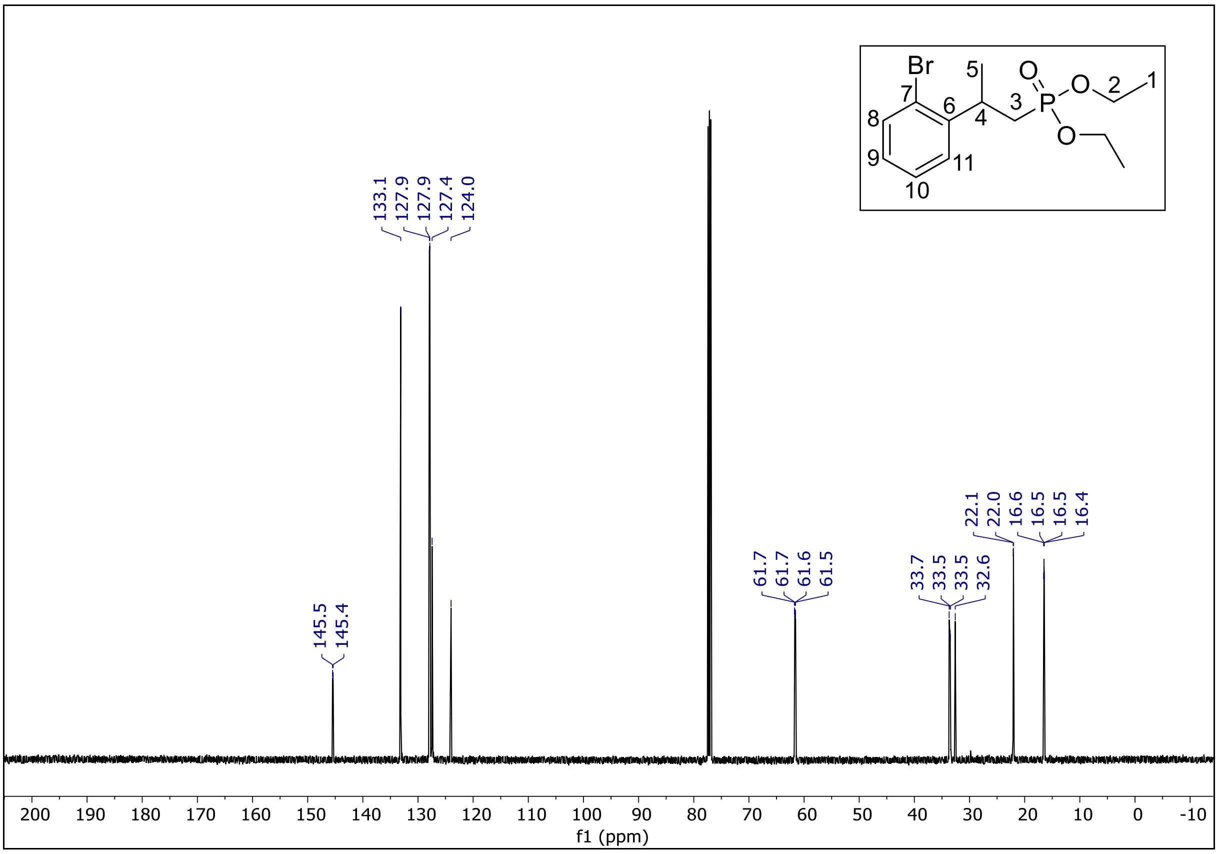

^{31}P NMR (162 MHz, $CDCl_3$): **34**

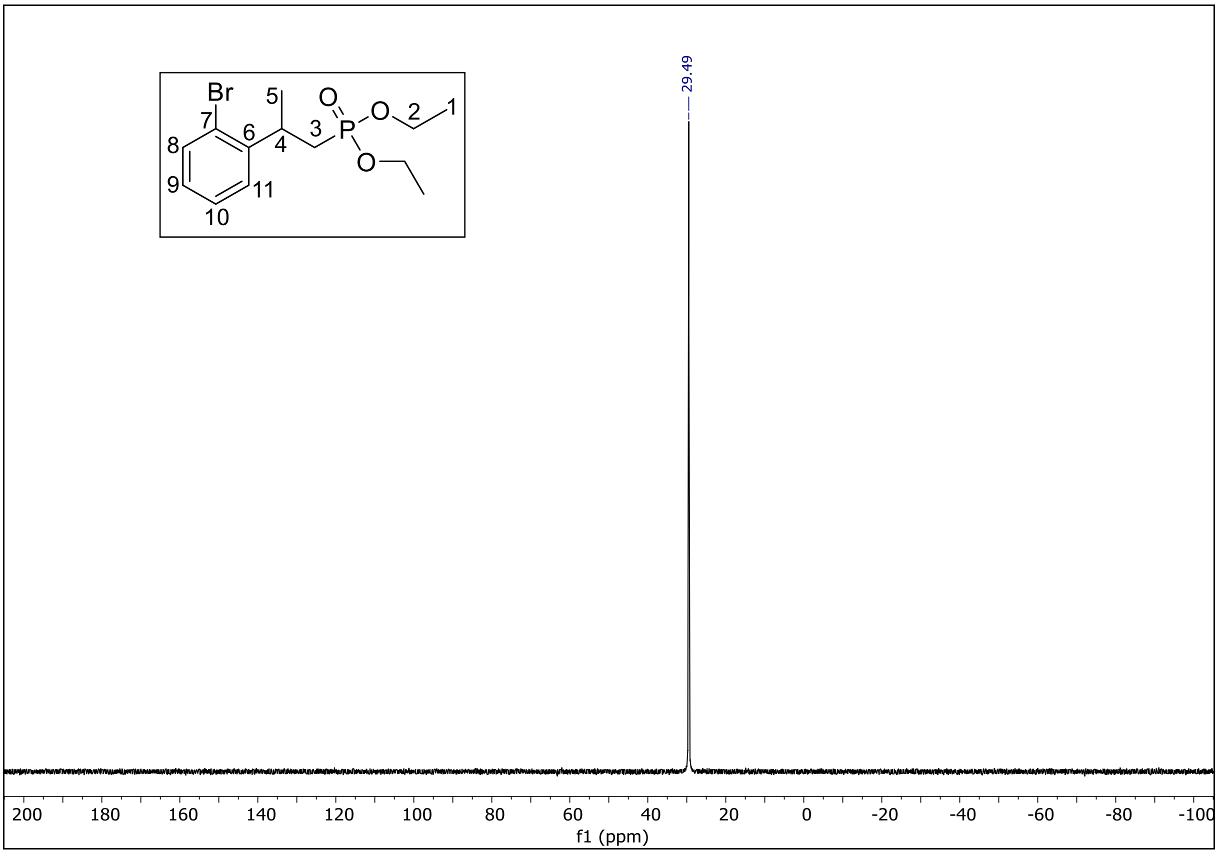

References

[1] Z. Duan, L. Wang, X. Song, Z. Zheng, *Tetrahedron: Asymmetry* **2012**, *23*, 508-514.

[2] G. Evano, K. Tadiparthi, F. Couty, *Chem. Commun.* **2011**, *47*, 179-181.

[3] J. Gu, C. Cai, *Org. Biomol. Chem.* **2017**, *15*, 4226-4230.

[4] G. Axelrad, S. Laosooksathit, R. Engel, *J. Org. Chem.* **1981**, *46*, 5200-5204.

[5] R. Blaszczyk, T. Gajda, *Heteroatom Chemistry* **2007**, *18*, 732-739.

[6] T. Hayashi, T. Senda, Y. Takaya, M. Ogasawara, *J. Am. Chem. Soc.* **1999**, *121*, 11591-11592.
